# Supplementary material for: Photo-induced ring-maintaining hydrosilylation of unactivated alkenes with hydrosilacyclobutanes
Source: Nat Commun. 2025 Mar 12;16:2468. doi: 10.1038/s41467-025-57705-w (PMC11903747; doi:10.1038/s41467-025-57705-w)
Supplement: Supplementary file 1 — Supplementary Information [file 41467_2025_57705_MOESM1_ESM.pdf]

Supplementary Information

Photo-induced ring-maintaining hydrosilylation of unactivated alkenes with hydrosilacyclobutanes

Shaowei Chen, Meiyun Gao, Xiaoqian He and Xiao Shen*

The Institute for Advanced Studies, Engineering Research Center of Organosilicon Compounds & Materials, Ministry of Education, Wuhan University, 299 Bayi Road, Wuhan, Hubei 430072, PR China. *E-mail: xiaoshen@whu.edu.cn

Table of contents

1. **General information……………………………………………………………..2**
2. **Reaction Setup ………………………….…………………………………………..2**
3. **Synthesis of substrates ……………….……………………………………………..3**
4. **Investigation of reaction conditions ……………….………………………………..8**
5. **Synthesis of silacyclobutanes ……….……………..………………………………..12**
6. **Gram scale reaction………………………………………………………………..39**
7. **Synthetic applications…………..……………………………………………….40**
8. **Mechanism studies ……………..……………………………………………….43**
9. **Computational Methods ……………...……………………………..……………52**
10. **NMR spectra for new compounds……………….…………………..……………53**
11. **References………………………..……………………………….……………..152**

1. General information

Chromatography: HaiLang Silica Flash P60 size 40~45 μm (300~400 mesh), TLC: HaiLang silica gel 60 (0.25 mm). Visualization of the chromatogram was performed by UV, phosphomolybdic acid and KMnO4 staining. Mass spectra were recorded on Bruker UltiMate 3000 & Compact, Thermo ISQ LT, LTQ XL and VELOS pro & ORBITRIP mass spectrometers. ^{1}H, ^{13}C were recorded on Bruker 600 and JNM-ECZ 400 using $CDCl_3$ as solvent. Chemical shift values are reported in ppm with the solvent resonance as the internal standard ($CDCl_3$: δ 7.26 for ^{1}H, δ 77.16 for ^{13}C). Data are reported as follows: chemical shifts, multiplicity (s = singlet, bs = broad singlet, d = doublet, dd = doublet of doublets, t = triplet, td = triplet of doublets, m = multiplet), coupling constants (Hz), and integration. Melting point was measured by INESA SGW X-4. All reagents were used as received and solvents were dried and degassed according to standard procedure. If no special description, all reactions were conducted under nitrogen. Silacyclobutanes **1a~1e** were synthesized according to literature precedence.[1] Olefins (**2z**, **2aa**, **2ae**, **2af**) were synthesized according to reported procedure.[2] Other Olefins were purchased from Bidepharm, Adamas, TCI and Energy Chemical.

2. Reaction Setup

Place a 20 ml reaction bottle at the hole of the photoreaction device. Two parallel LED lights (total 24 W, 390–395 nm) are placed perpendicular to the side wall of the reaction bottle (approximately 1 cm away from the light source), so that the reaction bottle can be equally exposed to the LED (approximately 6 W allocated to each hole). During the reaction process, a scissor fan at one end of the factory is constantly working to counteract the heat generated by the LED light and stabilize the reaction temperature for reproducing the results.

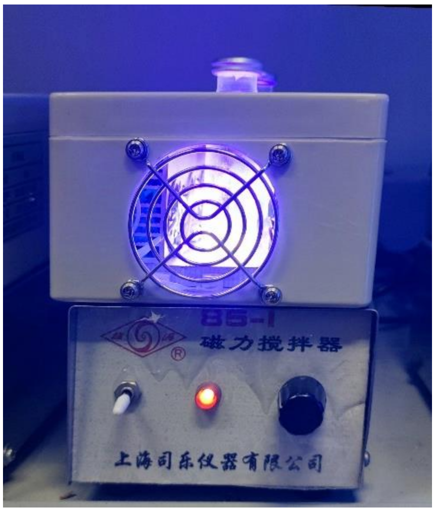

Supplementary Fig. 1. The device of photo-catalysis reaction (6 W)

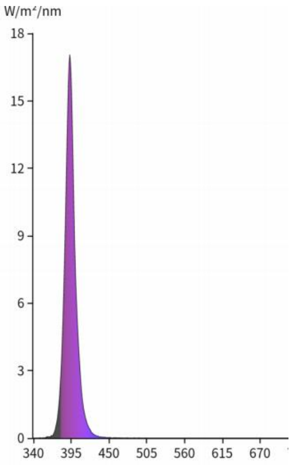

Supplementary Fig. 2. Emission spectra of the LEDs (6W, λmax = 393 nm) used in this work

3. Synthesis of substrates

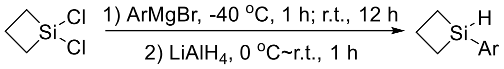

Procedure A: A solution of **S1** (10 mmol, 1 equiv.) in anhydrous THF (20 mL) was cooled to -40 °C. ArMgBr (10 mmol, 0.5 M in THF) was added dropwise at this temperature for 1 h. After warmed to room temperature and stirred for 12 h, the resulting solution was cooled to 0˚C. $LiAlH_4$ powder (430 mg, 10 mmol, 1 equiv.) was added in one portion to the mixture and stirred at room temperature for 1 h. Then H_2O (5 mL) was added dropwise into the mixture and then stirred for 10 minutes at 0 ˚C. The aqueous phase was extracted with diethyl ether and the organic layer was dried over Na_2SO_4, filtered and concentrated under vacuum. The resulting residue was purified by silica gel column chromatography to give the desired product **1a~1e**.

1-(*p*-tolyl)siletane (1a)

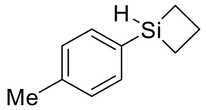

Following procedure **A**, compound **1a** was obtained by flash column chromatography (Pentane) in 68% yield as colorless oil. ^{1}H NMR (400 MHz, $CDCl_3$, 25 ºC) δ 7.57 (d, *J* = 7.5 Hz, 2H), 7.23 (d, *J* = 7.5 Hz, 2H), 5.19–5.15 (m, 1H), 2.38 (s, 3H), 2.35–2.19 (m, 2H), 1.38–1.25 (m, 4H). The spectroscopic data for this product matched the literature data[1].

1-phenylsiletane (1b)

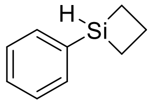

Following procedure **A**, compound **1b** was obtained by flash column chromatography (Pentane) in 62% yield as colorless oil. ^{1}H NMR (600 MHz, $CDCl_3$, 25 ºC) δ 7.70–7.62 (m, 2H), 7.45–7.35 (m, 3H), 5.22–5.14 (m, 1H), 2.37–2.17 (m, 2H), 1.39–1.28 (m, 4H). The spectroscopic data for this product matched the literature data[1].

1-(4-fluorophenyl)siletane (1c)

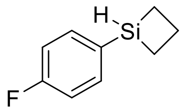

Following procedure **A**, compound **1c** was obtained by flash column chromatography (Pentane) in 45% yield as colorless oil. ^{1}H NMR (400 MHz, $CDCl_3$, 25 ºC) δ 7.69–7.62 (m, 2H), 7.15–7.05 (m, 2H), 5.21–5.15 (m, 1H), 2.40–2.18 (m, 2H), 1.43–1.33 (m, 2H), 1.33–1.25 (m, 2H). The spectroscopic data for this product matched the literature data[1].

1-(4-(tert-butyl)phenyl)siletane (1d)

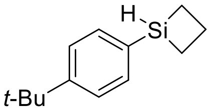

Following procedure **A**, compound **1d** was obtained by flash column chromatography (Pentane) in 67% yield as colorless oil. ^{1}H NMR (400 MHz, $CDCl_3$, 25 ºC) δ 7.64 (d, J = 8.3 Hz, 2H), 7.46 (d, J = 8.2 Hz, 2H), 5.25–5.13 (m, 1H), 2.44–2.15 (m, 2H), 1.43–1.24 (m, 13H). The spectroscopic data for this product matched the literature data[1].

N, N-dimethyl-4-(siletan-1-yl)aniline (1e)

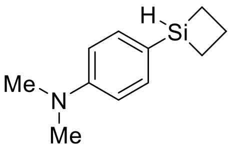

Following procedure **A**, compound **1e** was obtained by flash column chromatography (PE/EA=50/1, v/v) in 41% yield as colorless oil. ^{1}H NMR (400 MHz, $CDCl_3$, 25 ºC) δ 7.59 (d, J = 8.7 Hz, 2H), 6.80 (d, J = 8.7 Hz, 2H), 5.17–5.09 (m, 1H), 3.02 (s, 6H), 2.37–2.15 (m, 2H), 1.40–1.23 (m, 4H). The spectroscopic data for this product matched the literature data[1].

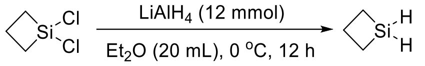

S1 (1.2 mL, 10 mmol, 1 equiv.) was dissolved in anhydrous Et_2O (22 mL) under N_2, then the resulting solution was cooled to 0˚C. $LiAlH_4$ powder (456 mg, 12 mmol, 1.2 equiv.) was added in one portion to the mixture, then the reaction mixture was stirred at 0 ˚C over 18 h. Then H_2O (1 mL) was added dropwise into the mixture and then stirred for 15 minutes at 0 ˚C. The reaction mixture was filtered quickly through a short silica gel plug (2~3 cm) to obtain the **1f** solution (20.3 mL, 0.38 M in Et_2O, 77% yield). The concentration of **1f** was determined by ^{1}H NMR (1,2-dibromoethane as internal standard). ^{1}H NMR (400 MHz, $CDCl_3$, 25 ºC) δ 4.58–4.55 (m, 2H, SiH), 2.28–2.19 (m, 2H, H_1), 3.60 (S, 1,2-dibromoethane), (H_2, $H_{2'}$ overlap with Et_2O). ^{29}Si NMR (119 MHz, $CDCl_3$/Et_2O, 25 ºC) δ -24.2.

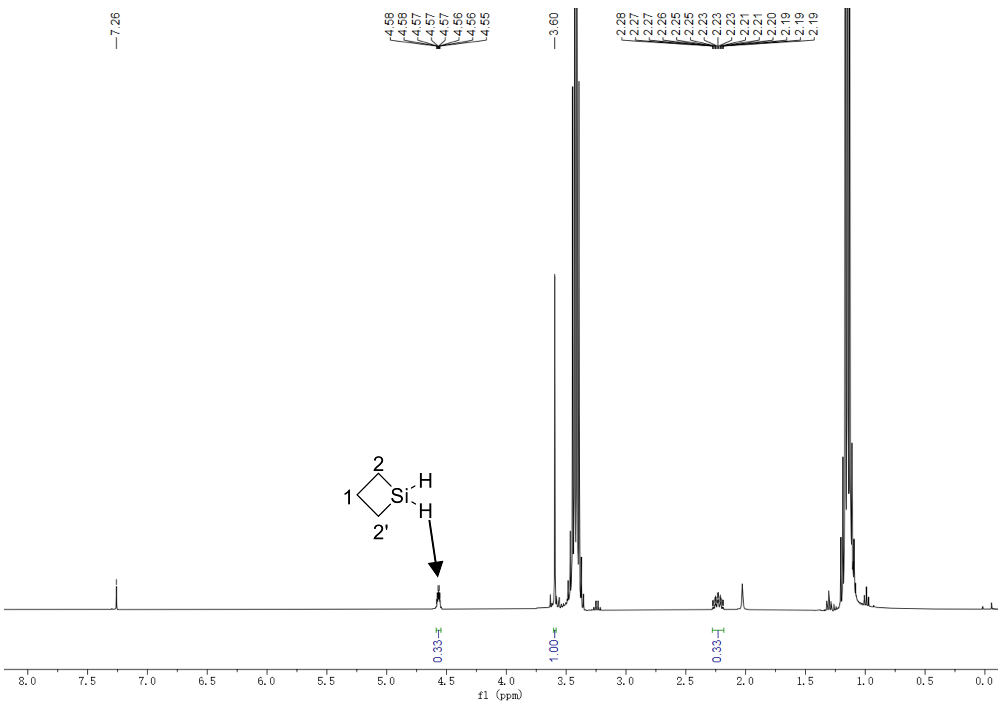

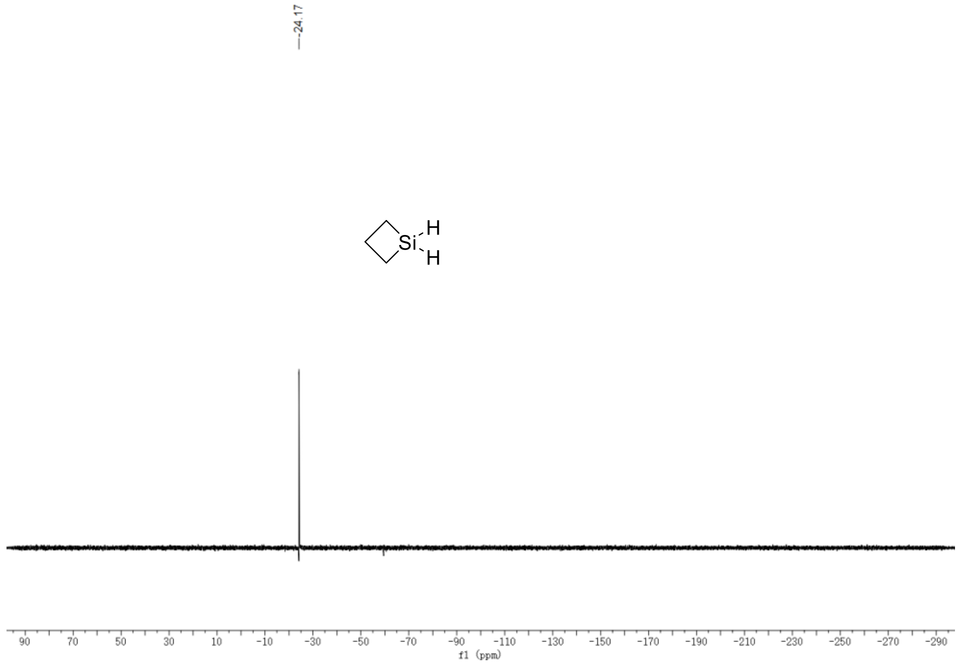

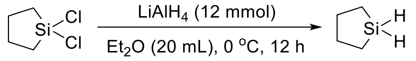

S2 **1g**

S2 (1.55 g, 10 mmol, 1 equiv.) was dissolved in anhydrous Et_2O (22 mL) under N_2, then the resulting solution was cooled to 0˚C. $LiAlH_4$ powder (456 mg, 12 mmol, 1.2 equiv.) was added in one portion to the mixture, then the reaction mixture was stirred at 0 ˚C over 18 h. Then H_2O (1 mL) was added dropwise into the mixture and then stirred for 15 minutes at 0 ˚C. The reaction mixture was filtered quickly through a short silica gel plug (2~3 cm) to obtain the **1g** solution (20 mL, 0.38 M in Et_2O, 76% yield). The concentration of **1g** was determined by ^{1}H NMR (1,2-dibromoethane as internal standard). ^{1}H NMR (400 MHz, $CDCl_3$, 25 °C) δ 3.72–3.68 (m, 2H, SiH), 1.53–1.49 (m, 4H, H_1, $H_{1'}$), 0.73–0.67 (m, 4H, H_2, $H_{2'}$), 3.57 (S, 1,2-dibromoethane). ^{29}Si NMR (119 MHz, $CDCl_3$/Et_2O, 25 ℃) δ -26.2.

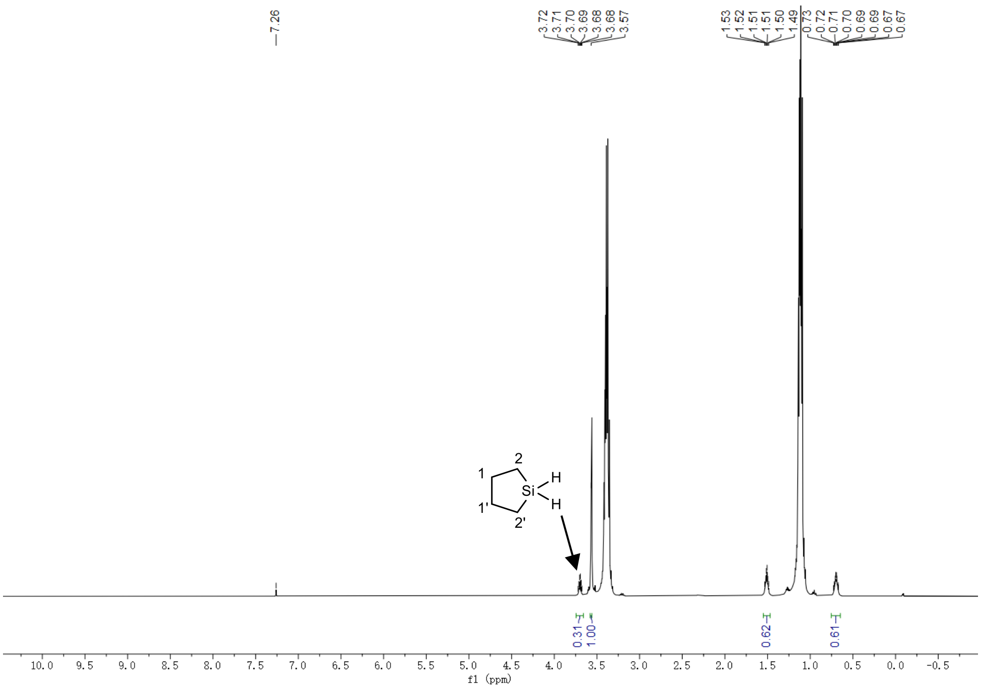

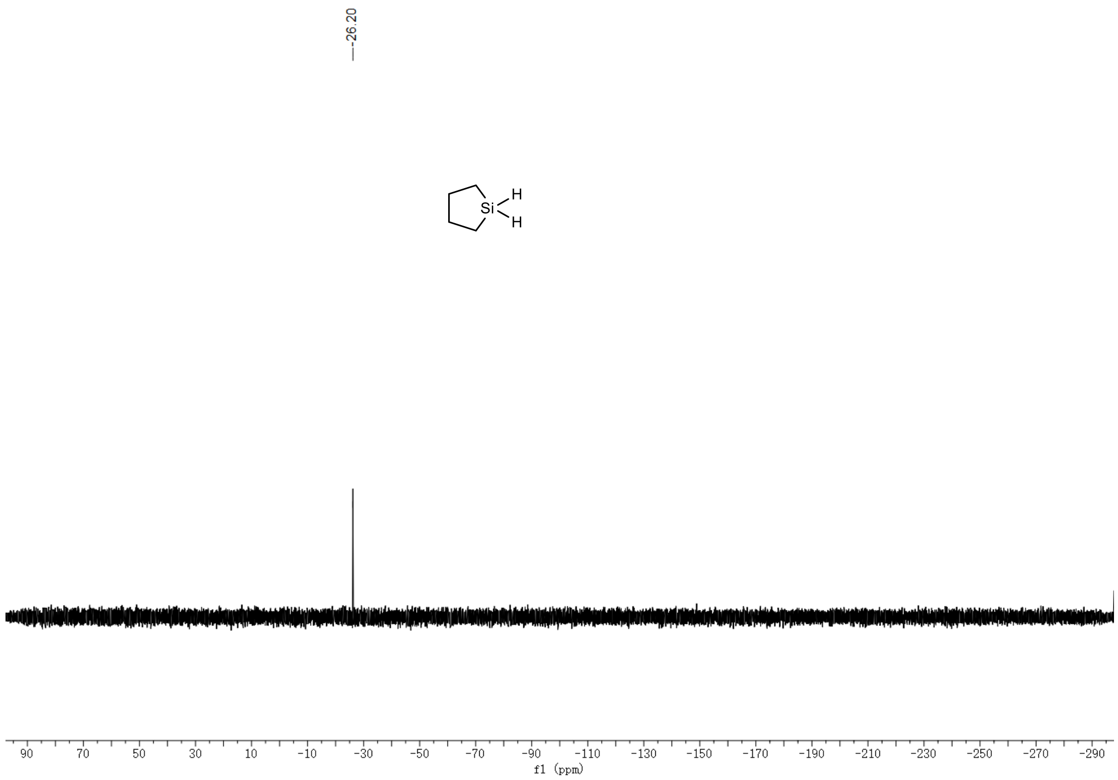

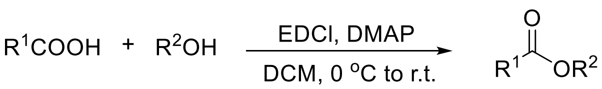

Procedure B: The corresponding acid (10.0 mmol, 1.0 equiv.) was added to a solution of 1-(3 dimethylaminopropyl)-3-ethylcarbodiimide (EDCI, 13.0 mmol, 1.3 equiv.) and DMAP (1.0 mmol 0.1 equiv.) in CH_2Cl_2 (25 mL) at 0 °C. Alcohol (12.0 mmol 1.2 equiv.) was then added and stirred at room temperature for 12 h. The solution was diluted with CH_2Cl_2 (40 mL) and washed with brine (40 mL) sequentially. The organic layer was dried over anhydrous Na_2SO_4. After removal of solvent under

reduced pressure, the crude product was purified by silica gel column chromatography to give the desired product **2z**, **2aa**, **2ae**, **2af**.

(*1R,2S,5R*)-2-isopropyl-5-methylcyclohexyl pent-4-enoate (2z)

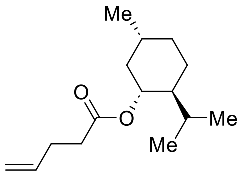

Following procedure **B**, compound **2z** was obtained by flash column chromatography (PE/EA=50/1, v/v) in 81% yield as colorless oil. ^{1}H NMR (600 MHz, $CDCl_3$, 25 ºC) δ 5.90–5.72 (m, 1H), 5.10–4.93 (m, 2H), 4.74–4.61 (m, 1H), 2.41–2.35 (m, 4H), 2.01–1.94 (m, 1H), 1.92–1.80 (m, 1H), 1.73–1.62 (m, 2H), 1.54–1.42 (m, 1H), 1.40–1.32 (m, 1H), 1.10–1.00 (m, 1H), 1.00–0.92 (m, 1H), 0.93-0.81 (m, 7H), 0.75 (d, J = 10.1 Hz, 3H). The spectroscopic data for this product matched the literature data[2b].

(3*S*,8*R*,9*S*,10*R*,13*S*,14*S*)-10,13-dimethyl-17-oxo-2,3,4,7,8,9,10,11,12,13,14,15,16,17-tetradecahydro-1*H*-cyclopenta[a]phenanthren-3-yl pent-4-enoate (2aa)

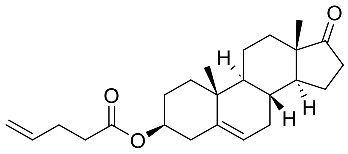

Following procedure **B**, compound **2aa** was obtained by flash column chromatography (PE/EA=10/1, v/v) in 73% yield as white solid. ^{1}H NMR (600 MHz, $CDCl_3$, 25 ºC) δ 5.88–5.76 (m, 1H), 5.41 (d, J = 4.4 Hz, 1H), 5.10–4.98 (m, 2H), 4.68–4.58 (m, 1H), 2.50–2.43 (m, 1H), 2.41–2.29 (m, 5H), 2.16–2.05 (m, 2H), 1.99–1.92 (m, 1H), 1.91–1.81 (m, 3H), 1.72–1.59 (m, 4H), 1.57–1.45 (m, 3H), 1.34–1.24 (m, 2H), 1.19–1.11 (m, 1H), 1.09–0.98 (m, 4H), 0.89 (s, 3H). The spectroscopic data for this product matched the literature data[2c].

3,7-dimethyloct-6-en-1-yl pent-4-enoate (2ae)

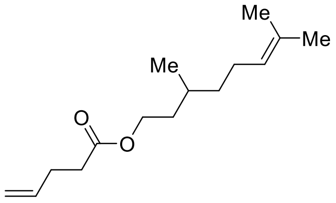

Following procedure **B**, compound **2ae** was obtained by flash column chromatography (PE/EA=50/1, v/v) in 78% yield as colorless oil. ^{1}H NMR (600 MHz, $CDCl_3$, 25 ºC) δ 5.86–5.77 (m, 1H), 5.11–4.96 (m, 3H), 4.16–4.05 (m, 2H), 2.43–2.34 (m, 4H), 2.04–1.89 (m, 2H), 1.69–1.63 (m, 4H), 1.60 (s, 3H), 1.58–1.50 (m, 1H), 1.46–1.40 (m, 1H), 1.38–1.30 (m, 1H), 1.22–1.14 (m, 1H), 0.91 (d, J = 6.6 Hz, 3H). ^{13}C NMR (151 MHz, $CDCl_3$, 25 ºC) δ 173.3, 136.9, 131.5, 124.7, 115.6, 63.1, 37.1, 35.6, 33.8, 29.6, 29.1, 25.9, 25.5, 19.5, 17.8. HRMS (ESI): (m/z) calcd for $C_{15}H_{27}O_2^+$ (M+H$^+$), 239.2006; found, 239.2010.

(3*S*,8*S*,9*S*,10*R*,13*R*,14*S*,17*R*)-10,13-dimethyl-17-((*R*)-6-methylheptan-2-yl)-2,3,4,7,8,9,10,11,12,13,14,15,16,17-tetradecahydro-1*H*-cyclopenta[a]phenanthren-3-yl pent-4-enoate (2af)

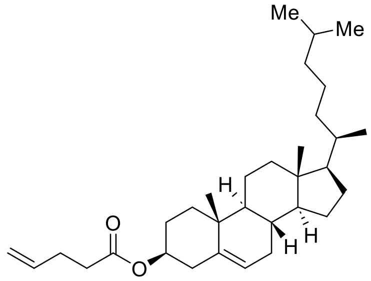

Following procedure **B**, compound **2ae** was obtained by flash column chromatography (PE/EA=50/1, v/v) in 64% yield as white solid. ^{1}H NMR (600 MHz, $CDCl_3$, 25 ºC) δ 5.87–5.76 (m, 1H), 5.36 (d, *J* = 5.0 Hz, 1H), 5.09–4.95 (m, 2H), 4.66–4.56 (m, 1H), 2.38–2.35 (m, 4H), 2.32–2.28 (m, 2H), 2.03–1.93 (m, 2H), 1.87–1.79 (m, 3H), 1.59–1.41 (m, 7H), 1.39–1.30 (m, 3H), 1.27–1.21 (m, 1H), 1.18–1.04 (m, 7H), 1.02–0.94 (m, 6H), 0.91 (d, *J* = 6.5 Hz, 3H), 0.86 (dd, *J* = 6.6, 2.7 Hz, 6H), 0.67 (s, 3H). ^{13}C NMR (151 MHz, $CDCl_3$, 25 ºC) δ 172.6, 139.8, 136.8, 122.7, 115.5, 74.0, 56.8, 56.3, 50.2, 42.4, 39.8, 39.6, 38.3, 37.1, 36.7, 36.3, 35.9, 33.9, 32.0, 31.9, 29.1, 28.3, 28.1, 27.9, 24.4, 23.9, 22.9, 22.7, 21.1, 19.4, 18.8, 11.9. HRMS (ESI): (m/z) calcd for $C_{32}H_{53}O_2^+$ (M+H$^+$), 469.4040; found, 469.4038.

4. Investigation of reaction conditions

4.1 Investigation of photocatalysts.

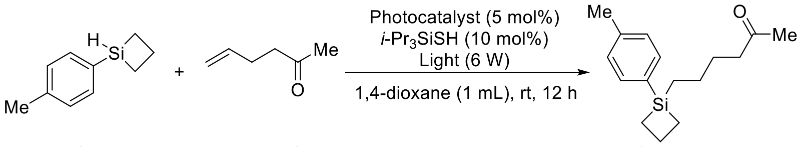

1a **2a** **3a**

Entry	PC	HAT catalyst	Solvent	Light	Yield of **3a**
1	eosin Y	*i*-Pr_3SiSH	1,4-dioxane	460 nm (6 W)	36%
2	eosin Y-Na	*i*-Pr_3SiSH	1,4-dioxane	460 nm (6 W)	38%
3	4CzIPN	*i*-Pr_3SiSH	1,4-dioxane	460 nm (6 W)	81%
4	TBADT	*i*-Pr_3SiSH	1,4-dioxane	390 nm (6 W)	64%
5	Benzophenone	*i*-Pr_3SiSH	1,4-dioxane	390 nm (6 W)	87%
6	-	*i*-Pr_3SiSH	1,4-dioxane	390 nm (6 W)	85%

[a] Reaction conditions: **1a** (0.1 mmol), **2a** (0.1 mmol), Photocatalyst (5 mol%), HAT catalyst (10 mol%), light (6 W), solvent (1 mL), 12 h.

4.2 Investigation of HAT catalysts.

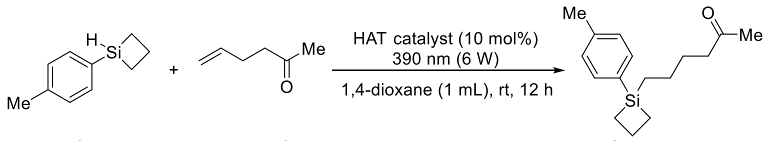

1a **2a** **3a**

Entry	HAT catalyst	Solvent	Light	Yield of **3a**
1	HAT1	1,4-dioxane	390 nm (6 W)	85%
2	HAT2	1,4-dioxane	390 nm (6 W)	19%
3	HAT3	1,4-dioxane	390 nm (6 W)	2%
4	HAT4	1,4-dioxane	390 nm (6 W)	2%
5	HAT5	1,4-dioxane	390 nm (6 W)	trace
6	HAT6	1,4-dioxane	390 nm (6 W)	4%
7	HAT7	1,4-dioxane	390 nm (6 W)	trace
8	HAT8	1,4-dioxane	390 nm (6 W)	trace
9	HAT9	1,4-dioxane	390 nm (6 W)	2%
10	HAT10	1,4-dioxane	390 nm (6 W)	2%
11	HAT11	1,4-dioxane	390 nm (6 W)	7%
12	HAT12	1,4-dioxane	390 nm (6 W)	16%

[a] Reaction conditions: **1a** (0.1 mmol), **2a** (0.1 mmol), HAT catalyst (10 mol%), light (6 W), solvent (1 mL), 12 h.

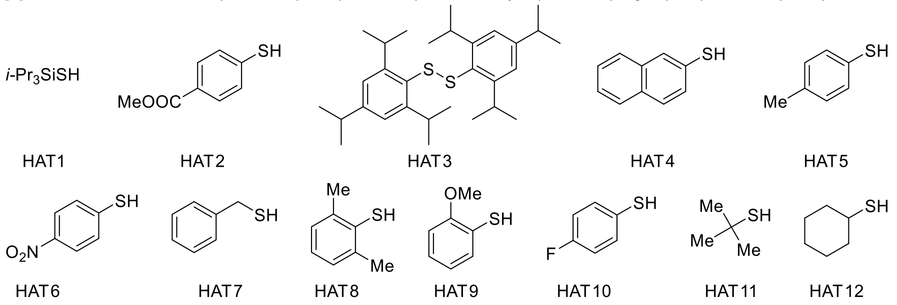

4.3 Investigation of the light (wavelength).

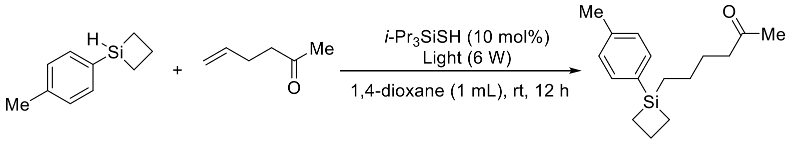

1a **2a** **3a**

Entry	HAT catalyst	Solvent	Light (6 W)	Yield of **3a**
1	*i*-Pr_3SiSH	1,4-dioxane	390-395 nm	85%
2	*i*-Pr_3SiSH	1,4-dioxane	400-410 nm	72%
3	*i*-Pr_3SiSH	1,4-dioxane	410-420 nm	56%
4	*i*-Pr_3SiSH	1,4-dioxane	420-430 nm	57%
5	*i*-Pr_3SiSH	1,4-dioxane	430-440 nm	50%
6	*i*-Pr_3SiSH	1,4-dioxane	440-450 nm	43%
7	*i*-Pr_3SiSH	1,4-dioxane	white LED	18%
8	*i*-Pr_3SiSH	1,4-dioxane	460-465 nm	trace
9	*i*-Pr_3SiSH	1,4-dioxane	under dark	ND

[a] Reaction conditions: **1a** (0.1 mmol), **2a** (0.1 mmol), HAT catalyst (10 mol%), light (6 W), solvent (1 mL), 12 h.

4.4 Investigation of the solvent.

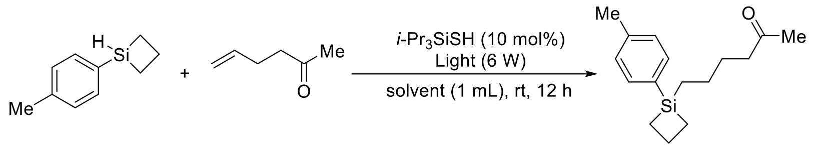

1a **2a** **3a**

Entry	HAT catalyst	Solvent	Light (6 W)	Yield of **3a**
1	*i*-Pr_3SiSH	1,4-dioxane	390-395 nm	85%
2	*i*-Pr_3SiSH	THF	390-395 nm	90%
3	*i*-Pr_3SiSH	Et_2O	390-395 nm	67%
4	*i*-Pr_3SiSH	MTBE	390-395 nm	23%
5	*i*-Pr_3SiSH	EA	390-395 nm	88%
6	*i*-Pr_3SiSH	CH_2Cl_2	390-395 nm	2%
7	*i*-Pr_3SiSH	PhMe	390-395 nm	2%
8	*i*-Pr_3SiSH	DMSO	390-395 nm	trace
9	*i*-Pr_3SiSH	DMF	390-395 nm	3%
10	*i*-Pr_3SiSH	CH_3CN	390-395 nm	4%
11	*i*-Pr_3SiSH	Acetone	390-395 nm	38%

[a] Reaction conditions: **1a** (0.1 mmol), **2a** (0.1 mmol), HAT catalyst (10 mol%), light (6 W), solvent (1 mL), 12 h.

Identifying the chloro derivative of **1a** coming from the reaction with DCM:

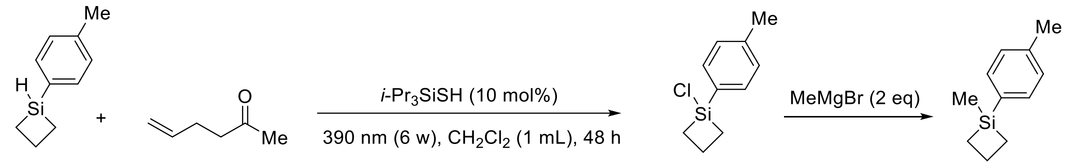

0.2 mmol 0.2 mmol Detected by ^{29}Si NMR **1a-1**, 65% yield

The chloro derivative of **1a** coming from the reaction with dichloromethane was observed by ^{29}Si NMR, the product was quenched by MeMgBr and compound **1a-1** was also isolated in 63% yield. ^{1}H NMR (600 MHz, $CDCl_3$, 25 ºC) δ 7.56–7.50 (m, 2H), 7.22 (d, J = 9.7 Hz, 2H), 2.37 (s, 3H), 2.21–2.13 (m, 2H), 1.32–1.23 (m, 2H), 1.19–1.10 (m, 2H), 0.54 (s, 3H). The spectroscopic data for **1a-1** matched the literature data.[3]

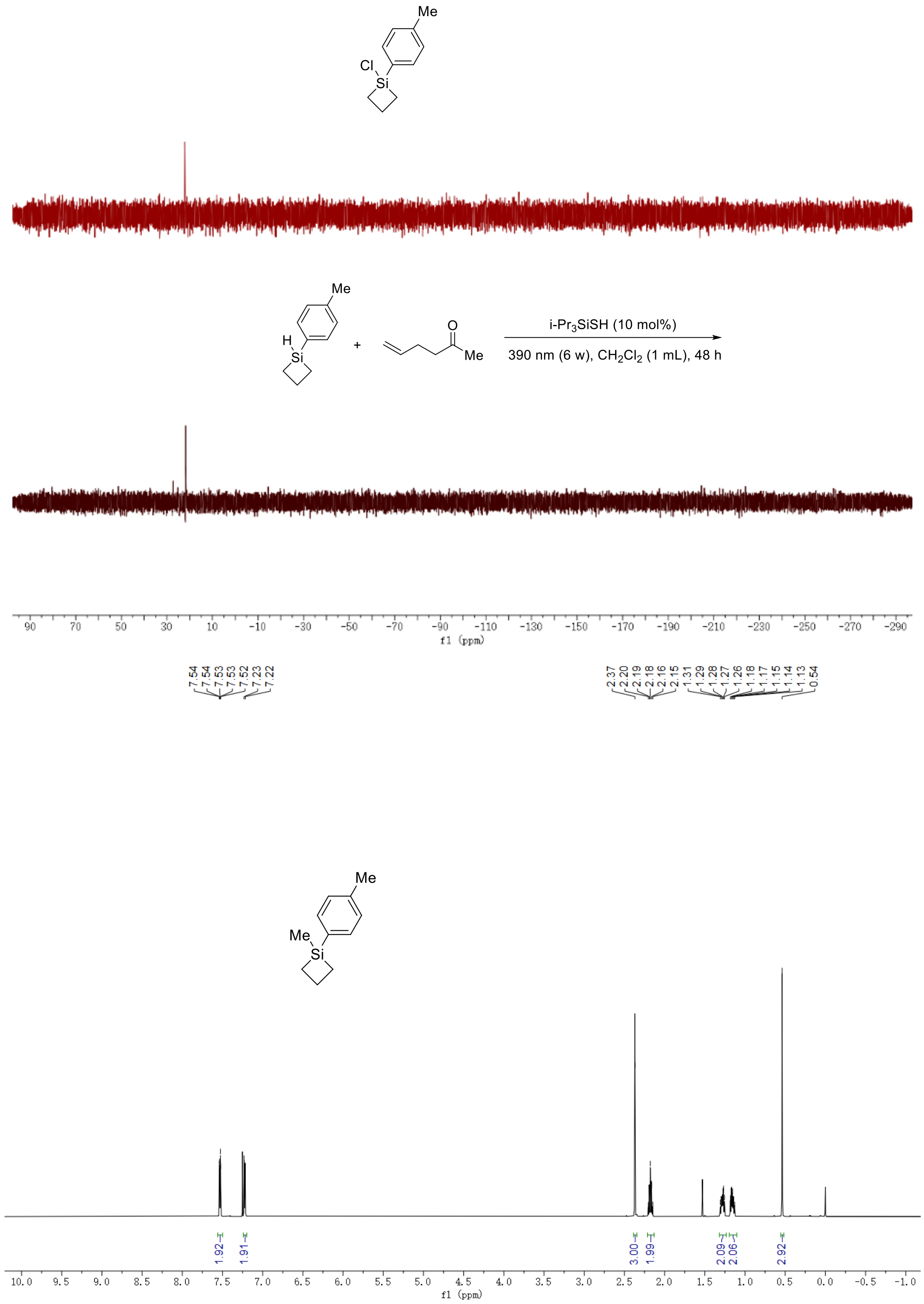

Supplementary Fig. 3. Identifying the chloro derivative of **1a**

4.5 Investigation of reaction time.

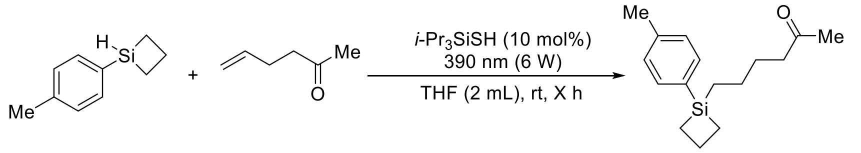

1a **2a** **3a**

Entry	HAT catalyst	X h	Light (6 W)	Yield of **3a**
1	*i*-Pr_3SiSH	24	390-395 nm	68%
2	*i*-Pr_3SiSH	36	390-395 nm	95% (90% isolated)
3	*i*-Pr_3SiSH	48	390-395 nm	95%

[a] Reaction conditions: **1a** (0.6 mmol), **2a** (0.5 mmol), HAT catalyst (10 mol%), light (6 W), solvent (2 mL), X h.

5. Synthesis of silacyclobutanes

6-(1-(*p*-Tolyl)siletan-1-yl)hexan-2-one (3a)

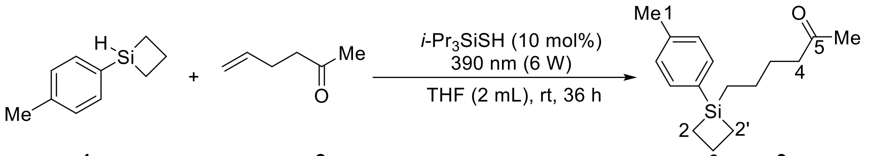

1a **2a** **3a**

In an argon-filled glovebox, to a flame-dried screw-cap reaction tube equipped with a magnetic stir bar were added *i*-Pr_3SiSH (9.5 mg, 0.05 mmol), **1a** (97.2 mg, 0.6 mmol, 1.2 equiv.), **2a** (49 mg, 0.5 mmol, 1 equiv.) and THF (2.0 mL) sequentially. The tube was sealed with a screw cap equipped with a septum, removed from the glovebox. The reaction mixture was stirred at rt for 36 h under 6 W 390 nm LED lamps. The reaction mixture was concentrated under reduced pressure. The crude product was purified with column chromatography on silica gel (200~300 mesh) with PE/EA (20/1, v/v) as eluent to afford 117 mg of the title compound **3a** as a colorless oil (90% yield).

^{1}H NMR (600 MHz, $CDCl_3$, 25 ºC) δ 7.50 (d, *J* = 7.5 Hz, 2H), 7.22 (d, *J* = 7.5 Hz, 2H), 2.43 (t, *J* = 7.4 Hz, 2H, H_4), 2.37 (s, 3H, H_1), 2.20–2.13 (m, 2H, H_3), 2.12 (s, 3H), 1.71–1.60 (m, 2H), 1.51–1.40 (m, 2H), 1.31–1.10 (m, 4H, H_2, $H_{2'}$), 1.08–0.87 (m, 2H). ^{13}C NMR (151 MHz, $CDCl_3$, 25 ºC) δ 209.3 (C_5), 139.4, 134.2, 133.8, 128.9, 43.6 (C_4), 29.9, 27.5, 23.5, 21.6 (C_1), 18.5 (C_3), 15.2, 13.0 (C_2, $C_{2'}$). ^{29}Si NMR (119 MHz, $CDCl_3$, 25 ºC) δ 13.8. HRMS (ESI): (m/z) calcd for $C_{16}H_{25}OSi^+$ ($M+H^+$), 261.1669; found, 261.1665.

5-(1-(*p*-Tolyl)siletan-1-yl)pentan-1-ol (3b)

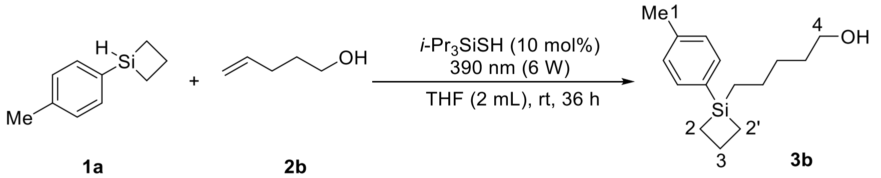

1a **2b** **3b**

In an argon-filled glovebox, to a flame-dried screw-cap reaction tube equipped with a magnetic stir bar were added *i*-Pr_3SiSH (9.5 mg, 0.05 mmol), **1a** (97.2 mg, 0.6 mmol, 1.2 equiv.), **2b** (43 mg, 0.5 mmol, 1 equiv.) and THF (2.0 mL) sequentially. The tube was sealed with a screw cap equipped with a septum, removed from the glovebox. The reaction mixture was stirred at rt for 36 h under 6 W 390 nm LED

lamps. The reaction mixture was concentrated under reduced pressure. The crude product was purified with column chromatography on silica gel (200~300 mesh) with PE/EA (20/1, v/v) as eluent to afford 103 mg of the title compound **3b** as a colorless oil (83% yield).

^{1}H NMR (400 MHz, $CDCl_3$, 25 °C) δ 7.53 (d, *J* = 7.8 Hz, 2H), 7.24 (d, *J* = 7.5 Hz, 2H), 3.63 (t, *J* = 6.6 Hz, 2H, H_4), 2.39 (s, 3H, H_1), 2.26–2.07 (m, 2H, H_3), 1.68–1.39 (m, 6H), 1.35–1.13 (m, 4H, H_2, $H_{2'}$), 1.12–0.91 (m, 2H). ^{13}C NMR (151 MHz, $CDCl_3$, 25 °C) δ 139.4, 134.4, 133.8, 128.9, 63.0 (C_4), 32.5, 29.5, 23.7, 21.6 (C_1), 18.5 (C_3), 15.3, 13.1 (C_2, $C_{2'}$). ^{29}Si NMR (119 MHz, $CDCl_3$, 25 °C) δ 13.8. HRMS (ESI): (m/z) calcd for $C_{15}H_{25}OSi^+$ (M+H$^+$), 249.1669; found, 249.1666.

5-(1-(*p*-Tolyl)siletan-1-yl)pentanoic acid (3c)

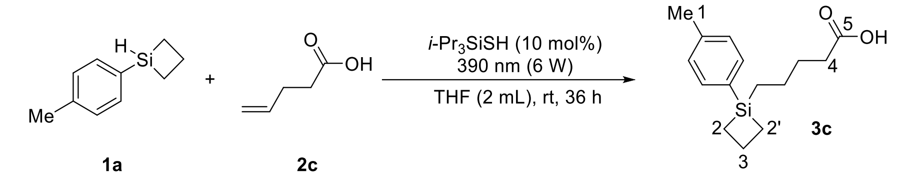

In an argon-filled glovebox, to a flame-dried screw-cap reaction tube equipped with a magnetic stir bar were added *i*-Pr_3SiSH (9.5 mg, 0.05 mmol), **1a** (97.2 mg, 0.6 mmol, 1.2 equiv.), **2c** (49 mg, 0.5 mmol, 1 equiv.) and THF (2.0 mL) sequentially. The tube was sealed with a screw cap equipped with a septum, removed from the glovebox. The reaction mixture was stirred at rt for 36 h under 6 W 390 nm LED lamps. The reaction mixture was concentrated under reduced pressure. The crude product was purified with column chromatography on silica gel (200~300 mesh) with PE/EA (2/1, v/v) as eluent to afford 102 mg of the title compound **3c** as a colorless oil (78% yield).

^{1}H NMR (600 MHz, $CDCl_3$, 25 °C) δ 7.51 (d, *J* = 7.9 Hz, 2H), 7.23 (d, *J* = 8.3 Hz, 2H), 2.41–2.33 (m, 5H, H_1, H_4), 2.24–2.09 (m, 2H, H_3), 1.79–1.68 (m, 2H), 1.59–1.49 (m, 2H), 1.30–1.15 (m, 4H, H_2, $H_{2'}$), 1.08–0.98 (m, 2H). ^{13}C NMR (151 MHz, $CDCl_3$, 25 °C) δ 180.1 (C_5), 139.5, 134.2, 133.8, 128.9, 33.8 (C_4), 28.3, 23.5, 21.6 (C_1), 18.5 (C_3), 15.1, 13.1 (C_2, $C_{2'}$). ^{29}Si NMR (119 MHz, $CDCl_3$, 25 °C) δ 13.8. HRMS (ESI): (m/z) calcd for $C_{15}H_{23}O_2Si^+$ (M+H$^+$), 263.1462; found, 263.1455.

6-(1-(*p*-Tolyl)siletan-1-yl)hexyl acetate (3d)

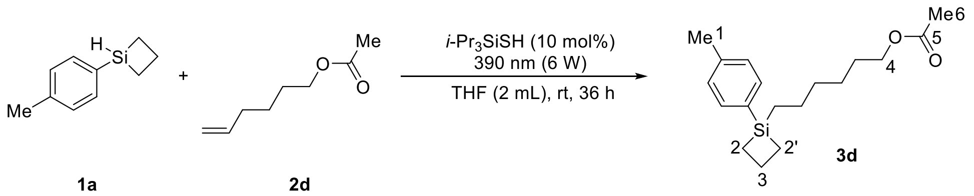

In an argon-filled glovebox, to a flame-dried screw-cap reaction tube equipped with a magnetic stir bar were added *i*-Pr_3SiSH (9.5 mg, 0.05 mmol), **1a** (97.2 mg, 0.6 mmol, 1.2 equiv.), **2d** (71 mg, 0.5 mmol, 1 equiv.) and THF (2.0 mL) sequentially. The tube was sealed with a screw cap equipped with a septum, removed from the glovebox. The reaction mixture was stirred at rt for 36 h under 6 W 390 nm LED lamps. The reaction mixture was concentrated under reduced pressure. The crude product was purified with column chromatography on silica gel (200~300 mesh) with PE/EA (20/1, v/v) as eluent to afford 146 mg of the title compound **3d** as a colorless oil (96% yield).

^{1}H NMR (400 MHz, $CDCl_3$, 25 °C) δ 7.52 (d, *J* = 7.9 Hz, 2H), 7.23 (d, *J* = 7.2 Hz, 2H), 4.06 (t, *J* = 6.7 Hz, 2H, H_4), 2.38 (s, 3H, H_1), 2.23–2.11 (m, 2H, H_3), 2.06 (s, 3H, H_6), 1.68–1.56 (m, 2H), 1.55–1.44 (m, 2H), 1.44–1.32 (m, 4H), 1.30–1.14 (m, 4H, H_2, $H_{2'}$), 1.09–0.92 (m, 2H). ^{13}C NMR (101 MHz, $CDCl_3$, 25

°C) δ 171.4 (C_5), 139.4, 134.4, 133.8, 128.9, 64.7 (C_4), 33.0, 28.6, 25.7, 23.7, 21.6 (C_1), 21.1, 18.5 (C_3), 15.2, 13.1 (C_2, $C_{2'}$). ^{29}Si NMR (119 MHz, $CDCl_3$, 25 °C) δ 13.8. HRMS (ESI): (m/z) calcd for $C_{18}H_{29}O_2Si^+$ ($M+H^+$), 305.1931; found, 305.1929.

11-(1-(*p*-Tolyl)siletan-1-yl)undecanal (3e)

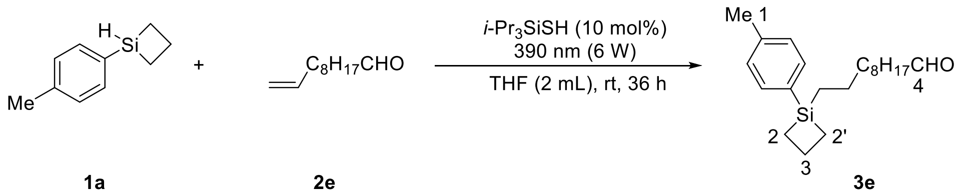

In an argon-filled glovebox, to a flame-dried screw-cap reaction tube equipped with a magnetic stir bar were added *i*-Pr_3SiSH (9.5 mg, 0.05 mmol), **1a** (97.2 mg, 0.6 mmol, 1.2 equiv.), **2e** (84 mg, 0.5 mmol, 1 equiv.) and THF (2.0 mL) sequentially. The tube was sealed with a screw cap equipped with a septum, removed from the glovebox. The reaction mixture was stirred at rt for 36 h under 6 W 390 nm LED lamps. The reaction mixture was concentrated under reduced pressure. The crude product was purified with column chromatography on silica gel (200~300 mesh) with PE/EA (100/1, v/v) as eluent to afford 133 mg of the title compound **3e** as a colorless oil (81% yield).

^{1}H NMR (400 MHz, $CDCl_3$, 25 °C) δ 9.77 (t, *J* = 1.9 Hz, 1H, H_4), 7.52 (d, *J* = 7.8 Hz, 2H), 7.22 (d, *J* = 7.6 Hz, 2H), 2.42 (td, *J* = 7.4, 1.9 Hz, 2H), 2.37 (s, 3H, H_1), 2.24–2.08 (m, 2H, H_3), 1.67–1.57 (m, 2H), 1.51–1.42 (m, 2H), 1.36–1.16 (m, 17H), 1.05–0.93 (m, 2H). ^{13}C NMR (101 MHz, $CDCl_3$, 25 °C) δ 203.1 (C_4), 139.3, 134.6, 133.8, 128.8, 44.1, 33.4, 29.6, 29.5, 29.5, 29.4, 29.3, 23.8, 22.2, 21.6 (C_1), 18.6 (C_3), 15.3, 13.1 (C_2, $C_{2'}$). ^{29}Si NMR (119 MHz, $CDCl_3$, 25 °C) δ 13.9. HRMS (APCI): (m/z) calcd for $C_{21}H_{35}OSi$ (M), 331.2457; found, 331.2455.

4-(1-(*p*-Tolyl)siletan-1-yl)butanenitrile (3f)

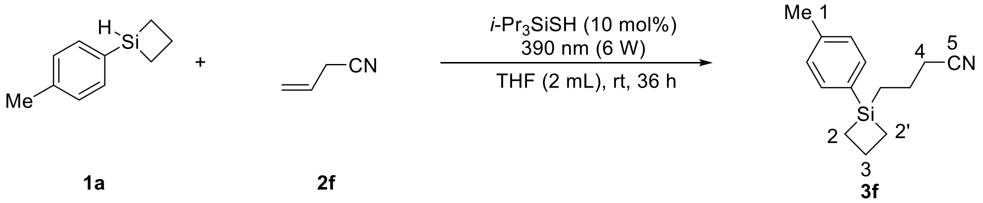

In an argon-filled glovebox, to a flame-dried screw-cap reaction tube equipped with a magnetic stir bar were added *i*-Pr_3SiSH (9.5 mg, 0.05 mmol), **1a** (97.2 mg, 0.6 mmol, 1.2 equiv.), **2f** (33.5 mg, 0.5 mmol, 1 equiv.) and THF (2.0 mL) sequentially. The tube was sealed with a screw cap equipped with a septum, removed from the glovebox. The reaction mixture was stirred at rt for 36 h under 6 W 390 nm LED lamps. The reaction mixture was concentrated under reduced pressure. The crude product was purified with column chromatography on silica gel (200~300 mesh) with PE/EA (50/1, v/v) as eluent to afford 88 mg of the title compound **3f** as a colorless oil (77% yield).

^{1}H NMR (400 MHz, $CDCl_3$, 25 °C) δ 7.50 (d, *J* = 8.0 Hz, 2H), 7.24 (d, *J* = 7.5 Hz, 2H), 2.44–2.34 (m, 5H, H_1, H_4), 2.27–2.10 (m, 2H, H_3), 1.91–1.72 (m, 2H), 1.34–1.15 (m, 6H). ^{13}C NMR (151 MHz, $CDCl_3$, 25 °C) δ 139.9, 133.7, 133.1, 129.1, 119.7 (C_5), 21.6 (C_1), 20.7, 20.5, 18.5 (C_3), 14.9, 12.9 (C_2, $C_{2'}$). ^{29}Si NMR (119 MHz, $CDCl_3$, 25 °C) δ 13.0. HRMS (ESI): (m/z) calcd for $C_{14}H_{20}NSi^+$ ($M+H^+$), 230.1360; found, 230.1357.

1-Hexyl-1-(*p*-tolyl)siletane (3g)

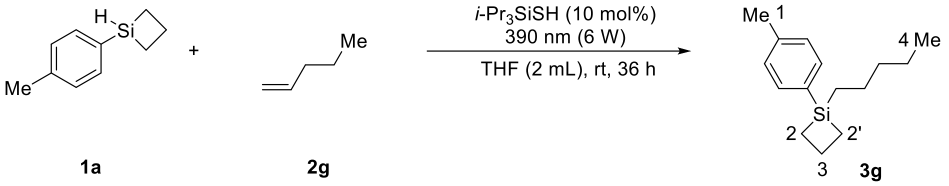

In an argon-filled glovebox, to a flame-dried screw-cap reaction tube equipped with a magnetic stir bar were added *i*-Pr_3SiSH (9.5 mg, 0.05 mmol), **1a** (97.2 mg, 0.6 mmol, 1.2 equiv.), **2g** (35 mg, 0.5 mmol, 1 equiv.) and THF (2.0 mL) sequentially. The tube was sealed with a screw cap equipped with a septum, removed from the glovebox. The reaction mixture was stirred at rt for 36 h under 6 W 390 nm LED lamps. The reaction mixture was concentrated under reduced pressure. The crude product was purified with column chromatography on silica gel (200~300 mesh) with PE as eluent to afford 106 mg of the title compound **3g** as a colorless oil (91% yield).

^{1}H NMR (600 MHz, $CDCl_3$, 25 ºC) δ 7.52 (d, *J* = 7.9 Hz, 2H), 7.22 (d, *J* = 7.5 Hz, 2H), 2.38 (s, 3H, H_1), 2.24–2.07 (m, 2H, H_3), 1.53–1.44 (m, 2H), 1.39–1.29 (m, 4H), 1.26–1.18 (m, 4H, H_2, $H_{2'}$), 1.03–0.96 (m, 2H), 0.89 (t, *J* = 7.1 Hz, 3H, H_4).^{13}C NMR (151 MHz, $CDCl_3$, 25 ºC) δ 139.3, 134.7, 133.8, 128.9, 35.7, 23.51, 22.5, 21.7 (C_1), 18.6 (C_3), 15.3, 14.2 (C_4), 13.1 (C_2, $C_{2'}$). ^{29}Si NMR (119 MHz, $CDCl_3$, 25 ºC) δ 13.9. HRMS (APCI): (m/z) calcd for $C_{15}H_{25}Si^+$ (M+H$^+$), 233.1720; found, 233.1717. Anal. calcd. For $C_{15}H_{24}Si$: C, 77.51; H, 10.41; Found: C, 77.67; H, 10.03.

1-(4-Phenylbutyl)-1-(*p*-tolyl)siletane (3h)

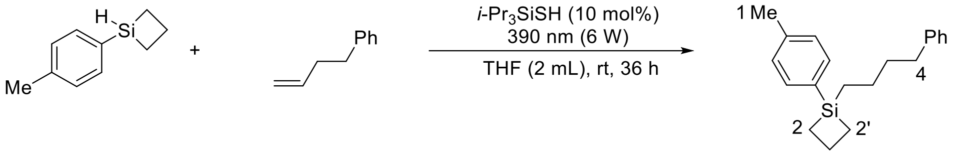

In an argon-filled glovebox, to a flame-dried screw-cap reaction tube equipped with a magnetic stir bar were added *i*-Pr_3SiSH (9.5 mg, 0.05 mmol), **1a** (97.2 mg, 0.6 mmol, 1.2 equiv.), **2h** (66 mg, 0.5 mmol, 1 equiv.) and THF (2.0 mL) sequentially. The tube was sealed with a screw cap equipped with a septum, removed from the glovebox. The reaction mixture was stirred at rt for 36 h under 6 W 390 nm LED lamps. The reaction mixture was concentrated under reduced pressure. The crude product was purified with column chromatography on silica gel (200~300 mesh) with PE as eluent to afford 132 mg of the title compound **3h** as a colorless oil (90% yield).

^{1}H NMR (600 MHz, $CDCl_3$, 25 ºC) δ 7.42 (d, *J* = 7.6 Hz, 2H), 7.20–7.16 (m, 2H), 7.13 (d, *J* = 7.6 Hz, 2H), 7.10–7.05 (m, 3H), 2.53 (t, *J* = 7.9 Hz, 2H, H_4), 2.28 (s, 3H, H_1), 2.15–1.98 (m, 2H, H_3), 1.68–1.56 (m, 2H), 1.53–1.42 (m, 2H), 1.20–1.06 (m, 4H, H_2, $H_{2'}$), 1.01–0.88 (m, 2H). ^{13}C NMR (151 MHz, $CDCl_3$, 25 ºC) δ 142.8, 139.4, 134.4, 133.8, 128.9, 128.5, 128.4, 125.7, 35.8 (C_4), 35.2, 23.6, 21.7 (C_1), 18.6 (C_3), 15.2, 13.1 (C_2, $C_{2'}$). ^{29}Si NMR (119 MHz, $CDCl_3$, 25 ºC) δ 13.8. HRMS (ESI): (m/z) calcd for $C_{20}H_{27}Si^+$ (M+H$^+$), 295.1877; found, 295.1869.

1-(6-Chlorohexyl)-1-(*p*-tolyl)siletane (3i)

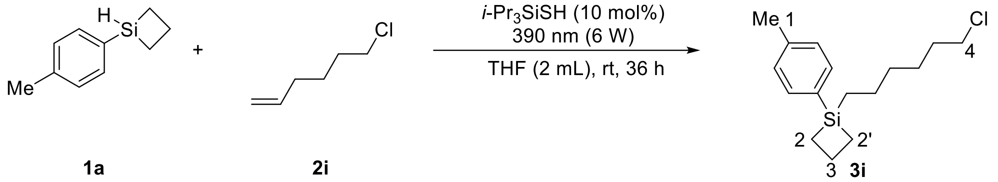

1a **2i** **3i**

In an argon-filled glovebox, to a flame-dried screw-cap reaction tube equipped with a magnetic stir bar were added *i*-Pr_3SiSH (9.5 mg, 0.05 mmol), **1a** (97.2 mg, 0.6 mmol, 1.2 equiv.), **2i** (59 mg, 0.5 mmol, 1 equiv.) and THF (2.0 mL) sequentially. The tube was sealed with a screw cap equipped with a septum, removed from the glovebox. The reaction mixture was stirred at rt for 36 h under 6 W 390 nm LED lamps. The reaction mixture was concentrated under reduced pressure. The crude product was purified with column chromatography on silica gel (200~300 mesh) with PE as eluent to afford 99 mg of the title compound **3i** as a colorless oil (70% yield).

^{1}H NMR (400 MHz, $CDCl_3$, 25 ºC) δ 7.53 (d, *J* = 7.8 Hz, 2H), 7.24 (d, *J* = 7.5 Hz, 2H), 3.54 (t, *J* = 6.8 Hz, 2H, H_4), 2.39 (s, 3H, H_1), 2.25–2.09 (m, 2H, H_3), 1.86–1.67 (m, 2H), 1.54–1.37 (m, 6H), 1.32–1.12 (m, 4H, H_2, $H_{2'}$), 1.07–0.93 (m, 2H). ^{13}C NMR (151 MHz, $CDCl_3$, 25 ºC) δ 139.4, 134.4, 133.8, 128.9, 45.3 (C_4), 32.7, 32.6, 26.7, 23.7, 21.7 (C_1), 18.6 (C_3), 15.2, 13.1 (C_2, $C_{2'}$). ^{29}Si NMR (119 MHz, $CDCl_3$, 25 ºC) δ 13.8. HRMS (APCI: (m/z) calcd for $C_{16}H_{26}ClSi^+$ (M+H^+), 281.1487; found, 281.1486.

1-(*p*-Tolyl)-1-(3-(trimethylsilyl)propyl)siletane (3j)

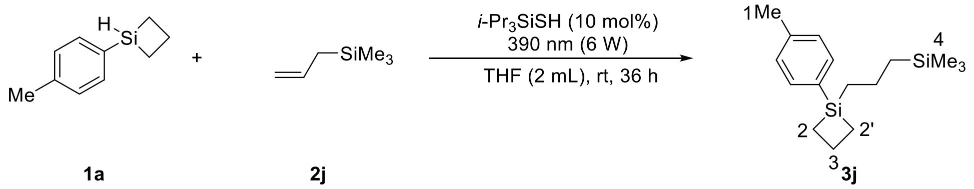

1a **2j** **3j**

In an argon-filled glovebox, to a flame-dried screw-cap reaction tube equipped with a magnetic stir bar were added *i*-Pr_3SiSH (9.5 mg, 0.05 mmol), **1a** (97.2 mg, 0.6 mmol, 1.2 equiv.), **2j** (57 mg, 0.5 mmol, 1 equiv.) and THF (2.0 mL) sequentially. The tube was sealed with a screw cap equipped with a septum, removed from the glovebox. The reaction mixture was stirred at rt for 36 h under 6 W 390 nm LED lamps. The reaction mixture was concentrated under reduced pressure. The crude product was purified with column chromatography on silica gel (200~300 mesh) with PE as eluent to afford 119 mg of the title compound **3j** as a colorless oil (86% yield).

^{1}H NMR (600 MHz, $CDCl_3$, 25 ºC) δ 7.53 (d, *J* = 7.3 Hz, 2H), 7.27–7.21 (m, 2H), 2.39 (s, 3H, H_1), 2.23–2.11 (m, 2H, H_3), 1.59–1.49 (m, 2H), 1.27–1.19 (m, 2H, H_2, $H_{2'}$), 1.13–1.03 (m, 4H), 0.71–0.58 (m, 2H), -0.01 (s, 9H, H_4). ^{13}C NMR (101 MHz, $CDCl_3$, 25 ºC) δ 139.3, 134.7, 133.8, 128.9, 21.7 (C_1), 21.2, 19.7, 18.7 (C_3), 18.5, 13.2 (C_2, $C_{2'}$), -1.4 (C_4). ^{29}Si NMR (119 MHz, $CDCl_3$, 25 ºC) δ 13.3, 0.7. HRMS (ESI): (m/z) calcd for $C_{16}H_{29}Si_2^+$ (M+H^+), 277.1802; found, 277.1803.

1-(3-(4,4,5,5-tetramethyl-1,3,2-dioxaborolan-2-yl)propyl)-1-(*p*-tolyl)siletane (3k)

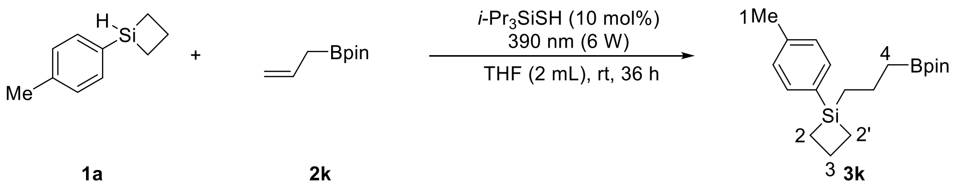

1a **2k** **3k**

In an argon-filled glovebox, to a flame-dried screw-cap reaction tube equipped with a magnetic stir bar were added *i*-Pr_3SiSH (9.5 mg, 0.05 mmol), **1a** (97.2 mg, 0.6 mmol, 1.2 equiv.), **2k** (84 mg, 0.5 mmol, 1 equiv.) and THF (2.0 mL) sequentially. The tube was sealed with a screw cap equipped with a septum, removed from the glovebox. The reaction mixture was stirred at rt for 36 h under 6 W 390 nm LED lamps. The reaction mixture was concentrated under reduced pressure. The crude product was purified with column chromatography on silica gel (200~300 mesh) with PE/EA (100/1, v/v) as eluent to afford 119 mg of the title compound **3k** as a colorless oil (72% yield).

^{1}H NMR (400 MHz, $CDCl_3$, 25 ºC) δ 7.53 (d, *J* = 8.0 Hz, 2H), 7.21 (d, *J* = 8.0 Hz, 2H), 2.37 (s, 3H, H_1), 2.23–2.07 (m, 2H, H_3), 1.70–1.56 (m, 2H), 1.25 (s, 12H), 1.24–1.18 (m, 4H, H_2, $H_{2'}$), 1.09–1.01 (m, 2H), 0.91 (t, *J* = 7.6 Hz, 2H, H_4). ^{13}C NMR (151 MHz, $CDCl_3$, 25 ºC) δ 139.3, 134.6, 133.9, 128.8, 83.0, 24.9, 21.6 (C_1), 18.6 (C_3), 18.6, 18.3, 13.2 (C_2, $C_{2'}$). ^{29}Si NMR (119 MHz, $CDCl_3$, 25 ºC) δ 13.3. HRMS (ESI): (m/z) calcd for $C_{19}H_{32}BO_2Si^+$ ($M+H^+$), 331.2259; found, 331.2259.

1-(3-Phenoxypropyl)-1-(*p*-tolyl)siletane (3l)

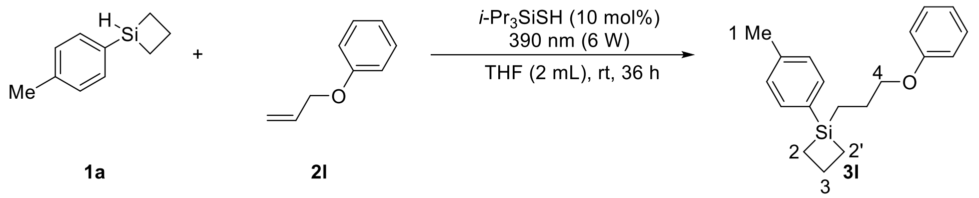

In an argon-filled glovebox, to a flame-dried screw-cap reaction tube equipped with a magnetic stir bar were added *i*-Pr_3SiSH (9.5 mg, 0.05 mmol), **1a** (97.2 mg, 0.6 mmol, 1.2 equiv.), **2l** (68 mg, 0.5 mmol, 1 equiv.) and THF (2.0 mL) sequentially. The tube was sealed with a screw cap equipped with a septum, removed from the glovebox. The reaction mixture was stirred at rt for 36 h under 6 W 390 nm LED lamps. The reaction mixture was concentrated under reduced pressure. The crude product was purified with column chromatography on silica gel (200~300 mesh) with PE as eluent to afford 91 mg of the title compound **3l** as a colorless oil (61% yield).

^{1}H NMR (600 MHz, $CDCl_3$, 25 ºC) δ 7.52 (d, *J* = 7.6 Hz, 2H), 7.29–7.23 (m, 2H), 7.22 (d, *J* = 7.5 Hz, 2H), 6.92 (t, *J* = 7.3 Hz, 1H), 6.87 (d, *J* = 8.2 Hz, 2H), 3.96 (t, *J* = 6.6 Hz, 2H, H_4), 2.37 (s, 3H, H_1), 2.23–2.11 (m, 2H, H_3), 2.02–1.89 (m, 2H), 1.32–1.19 (m, 4H, H_2, $H_{2'}$), 1.17–1.09 (m, 2H). ^{13}C NMR (151 MHz, $CDCl_3$, 25 ºC) δ 159.2, 139.5, 133.9, 133.8, 129.5, 128.9, 120.6, 114.6, 70.2 (C_4), 23.8, 21.7 (C_1), 18.5 (C_3), 13.1 (C_2, $C_{2'}$), 11.5. ^{29}Si NMR (119 MHz, $CDCl_3$, 25 ºC) δ 14.1. HRMS (ESI): (m/z) calcd for $C_{19}H_{25}OSi^+$ ($M+H^+$), 297.1669; found, 297.1667.

1-(4-(Oxiran-2-yl)butyl)-1-(*p*-tolyl)siletane (3m)

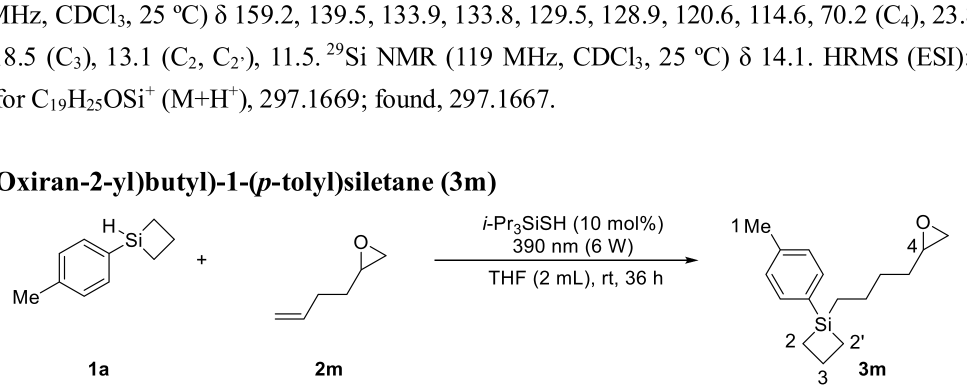

In an argon-filled glovebox, to a flame-dried screw-cap reaction tube equipped with a magnetic stir bar were added *i*-Pr_3SiSH (9.5 mg, 0.05 mmol), **1a** (97.2 mg, 0.6 mmol, 1.2 equiv.), **2m** (49 mg, 0.5 mmol, 1 equiv.) and THF (2.0 mL) sequentially. The tube was sealed with a screw cap equipped with a septum, removed from the glovebox. The reaction mixture was stirred at rt for 36 h under 6 W 390 nm LED lamps. The reaction mixture was concentrated under reduced pressure. The crude product was purified

with column chromatography on silica gel (200~300 mesh) with PE/EA (50/1, v/v) as eluent to afford 113 mg of the title compound **3m** as a colorless oil (87% yield).

^{1}H NMR (400 MHz, $CDCl_3$, 25 °C) δ 7.52 (d, *J* = 7.9 Hz, 2H), 7.24 (d, *J* = 7.6 Hz, 2H), 2.96–2.84 (m, 1H, H_4), 2.77–2.70 (m, 1H), 2.51–2.43 (m, 1H), 2.39 (s, 3H, H_1), 2.26–2.07 (m, 2H, H_3), 1.62–1.47 (m, 6H), 1.34–1.17 (m, 4H, H_2, $H_{2'}$), 1.12–0.96 (m, 2H). ^{13}C NMR (151 MHz, $CDCl_3$, 25 °C) δ 139.4, 134.3, 133.8, 128.8, 52.4 (C_4), 47.2, 32.3, 29.6, 23.7, 21.6 (C_1), 18.5 (C_3), 15.3, 13.1 (C_2, $C_{2'}$). ^{29}Si NMR (119 MHz, $CDCl_3$, 25 °C) δ 13.7. HRMS (ESI): (m/z) calcd for $C_{16}H_{25}OSi^+$ (M+H$^+$), 261.1669; found, 261.1665.

1-(2-(Cyclohex-3-en-1-yl)ethyl)-1-(*p*-tolyl)siletane (3n)

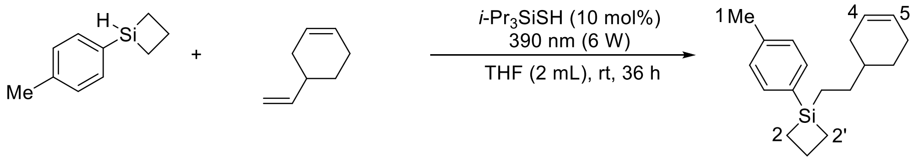

In an argon-filled glovebox, to a flame-dried screw-cap reaction tube equipped with a magnetic stir bar were added *i*-Pr_3SiSH (9.5 mg, 0.05 mmol), **1a** (97.2 mg, 0.6 mmol, 1.2 equiv.), **2n** (54 mg, 0.5 mmol, 1 equiv.) and THF (2.0 mL) sequentially. The tube was sealed with a screw cap equipped with a septum, removed from the glovebox. The reaction mixture was stirred at rt for 36 h under 6 W 390 nm LED lamps. The reaction mixture was concentrated under reduced pressure. The crude product was purified with column chromatography on silica gel (200~300 mesh) with PE as eluent to afford 77 mg of the title compound **3n** as a colorless oil (57% yield).

^{1}H NMR (600 MHz, $CDCl_3$, 25 °C) δ 7.52 (d, *J* = 7.8 Hz, 2H), 7.23 (d, *J* = 7.4 Hz, 2H), 5.70–5.64 (m, 2H, H_4, H_5), 2.38 (s, 3H, H_1), 2.22–2.10 (m, 3H), 2.09–1.95 (m, 2H), 1.79 (d, *J* = 10.9 Hz, 1H), 1.65 (m, 1H), 1.56–1.48 (m, 1H), 1.48–1.39 (m, 2H), 1.29–1.16 (m, 5H), 1.06–0.95 (m, 2H). ^{13}C NMR (151 MHz, $CDCl_3$, 25 °C) δ 139.4, 134.5, 133.8, 128.9, 127.2 (C_5), 126.8 (C_4), 36.5, 31.8, 30.5, 28.6, 25.5, 21.7(C_1), 18.5 (C_3), 13.0, 13.0 (C_2, $C_{2'}$), 12.3. ^{29}Si NMR (119 MHz, $CDCl_3$, 25 °C) δ 14.2. HRMS (ESI): (m/z) calcd for $C_{18}H_{27}Si^+$ (M+H$^+$), 271.1877; found, 271.1874.

1-(Hex-5-en-1-yl)-1-(*p*-tolyl)siletane (3o)

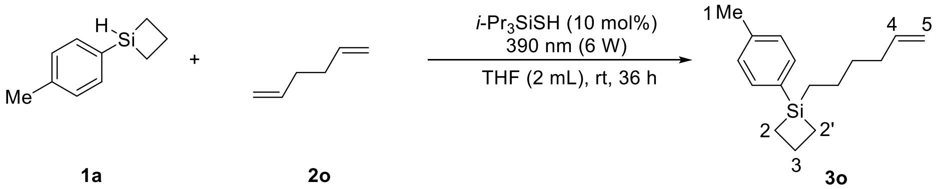

In an argon-filled glovebox, to a flame-dried screw-cap reaction tube equipped with a magnetic stir bar were added *i*-Pr_3SiSH (9.5 mg, 0.05 mmol), **1a** (81 mg, 0.5 mmol, 1 equiv.), **2o** (49 mg, 0.6 mmol, 1.2 equiv.) and THF (2.0 mL) sequentially. The tube was sealed with a screw cap equipped with a septum, removed from the glovebox. The reaction mixture was stirred at rt for 36 h under 6 W 390 nm LED lamps. The reaction mixture was concentrated under reduced pressure. The crude product was purified with column chromatography on silica gel (200~300 mesh) with PE as eluent to afford 78 mg of the title compound **3o** as a colorless oil (64% yield).

^{1}H NMR (400 MHz, $CDCl_3$, 25 °C) δ 7.53 (d, *J* = 7.9 Hz, 2H), 7.24 (d, *J* = 7.3 Hz, 2H), 5.90–5.73 (m, 1H, H_4), 5.08–4.87 (m, 2H, H_5), 2.39 (s, 3H, H_1), 2.24–2.12 (m, 2H, H_3), 2.12–2.03 (m, 2H), 1.55–1.40

(m, 4H), 1.28–1.15 (m, 4H, H_2, $H_{2'}$), 1.09–0.93 (m, 2H). ^{13}C NMR (101 MHz, $CDCl_3$, 25 ºC) δ 139.4, 139.2, 134.5, 133.8, 128.9, 114.4 (C_5), 33.6, 32.7, 23.4, 21.7 (C_1), 18.6 (C_3), 15.2, 13.1 (C_2, $C_{2'}$). ^{29}Si NMR (119 MHz, $CDCl_3$, 25 ºC) δ 13.9. HRMS (APCI): (m/z) calcd for $C_{16}H_{25}Si^+$ (M+H$^+$), 245.1720; found, 245.1725.

1,6-Bis(1-(*p*-tolyl)siletan-1-yl)hexane (3p)

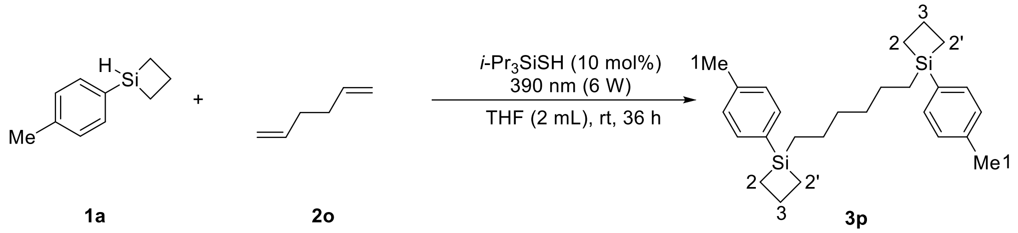

In an argon-filled glovebox, to a flame-dried screw-cap reaction tube equipped with a magnetic stir bar were added *i*-Pr_3SiSH (9.5 mg, 0.05 mmol), **1a** (194 mg, 1.2 mmol, 2.4 equiv.), **2o** (41 mg, 0.5 mmol, 1 equiv.) and THF (2.0 mL) sequentially. The tube was sealed with a screw cap equipped with a septum, removed from the glovebox. The reaction mixture was stirred at rt for 60 h under 6 W 390 nm LED lamps. The reaction mixture was concentrated under reduced pressure. The crude product was purified with column chromatography on silica gel (200~300 mesh) with PE as eluent to afford 150 mg of the title compound **3p** as a colorless oil (74% yield).

^{1}H NMR (400 MHz, $CDCl_3$, 25 ºC) δ 7.52 (d, *J* = 7.8 Hz, 4H), 7.23 (d, *J* = 7.8 Hz, 4H), 2.38 (s, 6H, H_1), 2.24–2.08 (m, 4H, H_3), 1.51–1.35 (m, 8H), 1.28–1.13 (m, 8H, H_2, $H_{2'}$), 1.05–0.89 (m, 4H). ^{13}C NMR (101 MHz, $CDCl_3$, 25 ºC) δ 139.3, 134.6, 133.8, 128.8, 33.1, 23.7, 21.7 (C_1), 18.6 (C_3), 15.3, 13.1 (C_2, $C_{2'}$). ^{29}Si NMR (119 MHz, $CDCl_3$, 25 ºC) δ 13.9. HRMS (APCI): (m/z) calcd for $C_{26}H_{39}Si_2^+$ (M+H$^+$), 407.2585; found, 407.2594.

1-(2-Ethoxyethyl)-1-(*p*-tolyl)siletane (3q)

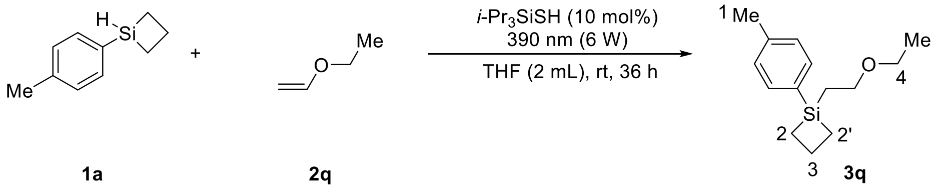

In an argon-filled glovebox, to a flame-dried screw-cap reaction tube equipped with a magnetic stir bar were added *i*-Pr_3SiSH (9.5 mg, 0.05 mmol), **1a** (97.2 mg, 0.6 mmol, 1.2 equiv.), **2q** (36 mg, 0.5 mmol, 1 equiv.) and THF (2.0 mL) sequentially. The tube was sealed with a screw cap equipped with a septum, removed from the glovebox. The reaction mixture was stirred at rt for 36 h under 6 W 390 nm LED lamps. The reaction mixture was concentrated under reduced pressure. The crude product was purified with column chromatography on silica gel (200~300 mesh) with PE as eluent to afford 89 mg of the title compound **3q** as a colorless oil (76% yield).

^{1}H NMR (400 MHz, $CDCl_3$, 25 ºC) δ 7.53 (d, *J* = 7.9 Hz, 2H), 7.23 (d, *J* = 7.5 Hz, 2H), 3.68–3.59 (m, 2H), 3.52–3.35 (m, 2H, H_4), 2.38 (s, 3H, H_1), 2.24–2.09 (m, 2H, H_3), 1.50–1.40 (m, 2H), 1.32–1.13 (m, 7H). ^{13}C NMR (101 MHz, $CDCl_3$, 25 ºC) δ 139.6, 133.8, 133.8, 128.9, 67.2, 65.8 (C_4), 21.6 (C_1), 18.6 (C_3), 17.2, 15.4, 13.3 (C_2, $C_{2'}$). ^{29}Si NMR (119 MHz, $CDCl_3$, 25 ºC) δ 11.7. HRMS (APCI): (m/z) calcd for $C_{14}H_{26}NOSi^+$ (M+NH_4^+), 252.1778; found, 252.1782.

1-(2-(Benzyloxy)ethyl)-1-(*p*-tolyl)siletane (3r)

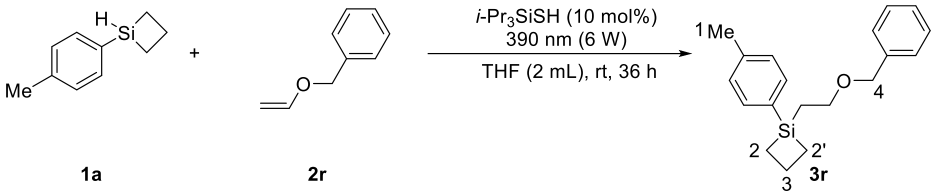

In an argon-filled glovebox, to a flame-dried screw-cap reaction tube equipped with a magnetic stir bar were added *i*-Pr_3SiSH (9.5 mg, 0.05 mmol), **1a** (97.2 mg, 0.6 mmol, 1.2 equiv.), **2r** (67 mg, 0.5 mmol, 1 equiv.) and THF (2.0 mL) sequentially. The tube was sealed with a screw cap equipped with a septum, removed from the glovebox. The reaction mixture was stirred at rt for 36 h under 6 W 390 nm LED lamps. The reaction mixture was concentrated under reduced pressure. The crude product was purified with column chromatography on silica gel (200~300 mesh) with PE as eluent to afford 90 mg of the title compound **3r** as a colorless oil (61% yield).

^{1}H NMR (400 MHz, $CDCl_3$, 25 ºC) δ 7.53 (d, *J* = 8.0 Hz, 2H), 7.39–7.26 (m, 5H), 7.25–7.18 (m, 2H), 4.51 (s, 2H, H_4), 3.84–3.57 (m, 2H), 2.39 (s, 3H, H_1), 2.25–2.08 (m, 2H, H_3), 1.55–1.43 (m, 2H), 1.36–1.09 (m, 4H, H_2, $H_{2'}$). ^{13}C NMR (151 MHz, $CDCl_3$, 25 ºC) δ 139.6, 138.7, 133.8, 133.8, 128.9, 128.5, 127.7, 127.6, 72.7 (C_4), 67.3, 21.7 (C_1), 18.6 (C_3), 17.2, 13.4 (C_2, $C_{2'}$). ^{29}Si NMR (119 MHz, $CDCl_3$, 25 ºC) δ 11.7. HRMS (APCI): (m/z) calcd for $C_{19}H_{28}NOSi^+$ ($M+NH_4^+$), 314.1935; found, 314.1933.

2-(1-(*p*-Tolyl)siletan-1-yl)ethyl propionate (3s)

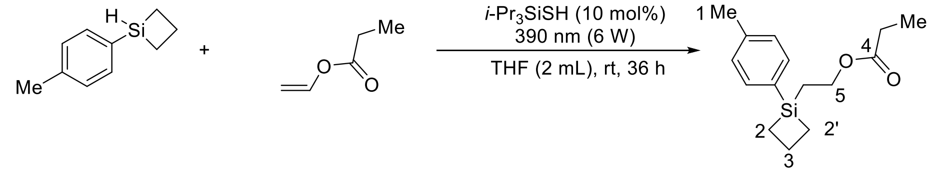

In an argon-filled glovebox, to a flame-dried screw-cap reaction tube equipped with a magnetic stir bar were added *i*-Pr_3SiSH (9.5 mg, 0.05 mmol), **1a** (97.2 mg, 0.6 mmol, 1.2 equiv.), **2s** (50 mg, 0.5 mmol, 1 equiv.) and THF (2.0 mL) sequentially. The tube was sealed with a screw cap equipped with a septum, removed from the glovebox. The reaction mixture was stirred at rt for 36 h under 6 W 390 nm LED lamps. The reaction mixture was concentrated under reduced pressure. The crude product was purified with column chromatography on silica gel (200~300 mesh) with PE/EA (20/1, v/v) as eluent to afford 124 mg of the title compound **3s** as a colorless oil (95% yield).

^{1}H NMR (400 MHz, $CDCl_3$, 25 ºC) δ 7.52 (d, *J* = 7.9 Hz, 2H), 7.24 (d, *J* = 7.5 Hz, 2H), 4.37–4.27 (m, 2H, H_5), 2.38 (s, 3H, H_1), 2.33–2.25 (m, 2H), 2.25–2.12 (m, 2H, H_3), 1.56–1.44 (m, 2H), 1.37–1.20 (m, 4H, H_2, $H_{2'}$), 1.13 (t, *J* = 7.6 Hz, 3H). ^{13}C NMR (151 MHz, $CDCl_3$, 25 ºC) δ 174.7 (C_4), 139.8, 133.7, 133.1, 129.0, 61.9 (C_5), 27.8, 21.6 (C_1), 18.5 (C_3), 16.2, 13.2 (C_2, $C_{2'}$), 9.2. ^{29}Si NMR (119 MHz, $CDCl_3$, 25 ºC) δ 11.7. HRMS (ESI): (m/z) calcd for $C_{15}H_{23}O_2Si^+$ ($M+H^+$), 263.1462; found, 263.1462.

1-(2-(Ethylthio)ethyl)-1-(*p*-tolyl)siletane (3t)

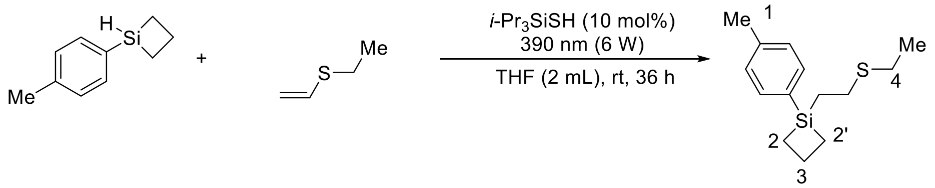

In an argon-filled glovebox, to a flame-dried screw-cap reaction tube equipped with a magnetic stir bar were added *i*-Pr_3SiSH (9.5 mg, 0.05 mmol), **1a** (97.2 mg, 0.6 mmol, 1.2 equiv.), **2t** (47 mg, 0.5 mmol, 1 equiv.) and THF (2.0 mL) sequentially. The tube was sealed with a screw cap equipped with a septum, removed from the glovebox. The reaction mixture was stirred at rt for 36 h under 6 W 390 nm LED lamps. The reaction mixture was concentrated under reduced pressure. The crude product was purified with column chromatography on silica gel (200~300 mesh) with PE as eluent to afford 104 mg of the title compound **3t** as a colorless oil (83% yield).

^{1}H NMR (400 MHz, $CDCl_3$, 25 °C) δ 7.51 (d, J = 7.9 Hz, 2H), 7.23 (d, J = 7.5 Hz, 2H), 2.75–2.66 (m, 2H), 2.56 (q, J = 7.4 Hz, 2H, H_4), 2.37 (s, 3H, H_1), 2.25–2.10 (m, 2H, H_3), 1.41–1.33 (m, 2H), 1.31–1.18 (m, 7H). ^{13}C NMR (151 MHz, $CDCl_3$, 25 °C) δ 139.7, 133.8, 133.4, 128.9, 26.8, 25.8 (C_4), 21.6 (C_1), 18.5 (C_3), 15.9, 14.8, 13.2 (C_2, $C_{2'}$). ^{29}Si NMR (119 MHz, $CDCl_3$, 25 °C) δ 12.6. HRMS (APCI): (m/z) calcd for $C_{14}H_{23}SSi^+$ (M+H$^+$), 251.1284; found, 251.1293.

1-(2-(1-(*p*-Tolyl)siletan-1-yl)ethyl)pyrrolidin-2-one (3u)

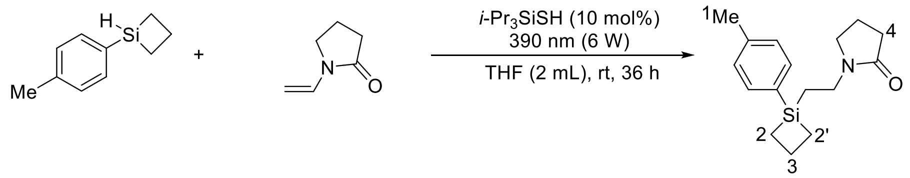

In an argon-filled glovebox, to a flame-dried screw-cap reaction tube equipped with a magnetic stir bar were added *i*-Pr_3SiSH (9.5 mg, 0.05 mmol), **1a** (97.2 mg, 0.6 mmol, 1.2 equiv.), **2u** (55.5 mg, 0.5 mmol, 1 equiv.) and THF (2.0 mL) sequentially. The tube was sealed with a screw cap equipped with a septum, removed from the glovebox. The reaction mixture was stirred at rt for 36 h under 6 W 390 nm LED lamps. The reaction mixture was concentrated under reduced pressure. The crude product was purified with column chromatography on silica gel (200~300 mesh) with PE/EA (10/1, v/v) as eluent to afford 125 mg of the title compound **3u** as a colorless oil (92% yield).

^{1}H NMR (400 MHz, $CDCl_3$, 25 °C) δ 7.50 (d, J = 8.0 Hz, 2H), 7.22 (d, J = 7.4 Hz, 2H), 3.54–3.43 (m, 2H), 3.40–3.25 (m, 2H), 2.36 (s, 3H, H_1), 2.31 (t, J = 8.1 Hz, 2H, H_4), 2.23–2.10 (m, 2H, H_3), 1.98–1.85 (m, 2H), 1.32–1.22 (m, 6H). ^{13}C NMR (151 MHz, $CDCl_3$, 25 °C) δ 174.5, 139.7, 133.6, 133.2, 128.9, 46.5, 38.4, 31.3 (C_4), 21.6 (C_1), 18.5 (C_3), 17.7, 14.1, 12.9 (C_2, $C_{2'}$). ^{29}Si NMR (119 MHz, $CDCl_3$, 25 °C) δ 11.9. HRMS (ESI): (m/z) calcd for $C_{16}H_{24}NOSi^+$ (M+H$^+$), 274.1622; found, 274.1617.

N-(2-(1-(*p*-tolyl)siletan-1-yl)ethyl)acetamide (3v)

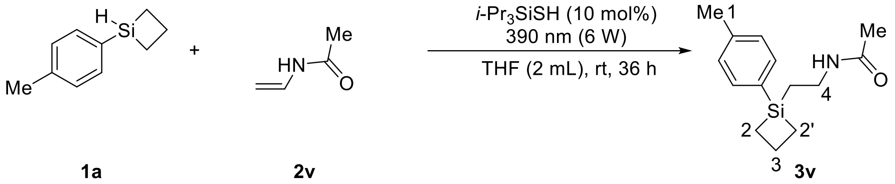

In an argon-filled glovebox, to a flame-dried screw-cap reaction tube equipped with a magnetic stir bar were added *i*-Pr_3SiSH (9.5 mg, 0.05 mmol), **1a** (97.2 mg, 0.6 mmol, 1.2 equiv.), **2v** (42.5 mg, 0.5 mmol, 1 equiv.) and THF (2.0 mL) sequentially. The tube was sealed with a screw cap equipped with a septum, removed from the glovebox. The reaction mixture was stirred at rt for 36 h under 6 W 390 nm LED lamps. The reaction mixture was concentrated under reduced pressure. The crude product was purified with column chromatography on silica gel (200~300 mesh) with PE/EA (2/1, v/v) as eluent to afford 95 mg of the title compound **3v** as a colorless oil (77% yield).

^{1}H NMR (400 MHz, $CDCl_3$, 25 ºC) δ 7.50 (d, *J* = 8.0 Hz, 2H), 7.22 (d, *J* = 8.3 Hz, 2H), 5.56 (s, 1H), 3.53–3.32 (m, 2H, H_4), 2.36 (s, 3H, H_1), 2.27–2.06 (m, 2H, H_3), 1.89 (s, 3H), 1.44–1.03 (m, 6H). ^{13}C NMR (151 MHz, $CDCl_3$, 25 ºC) δ 169.9, 139.8, 133.7, 133.3, 129.1, 35.8 (C_4), 23.4, 21.6 (C_1), 18.5 (C_3), 16.4, 13.0 (C_2, $C_{2'}$). ^{29}Si NMR (119 MHz, $CDCl_3$, 25 ºC) δ 12.2. HRMS (ESI): (m/z) calcd for $C_{14}H_{22}NOSi^+$ (M+H$^+$), 248.1465; found, 248.1461.

N-methyl-N-(2-(1-(*p*-tolyl)siletan-1-yl)ethyl)acetamide (3w)

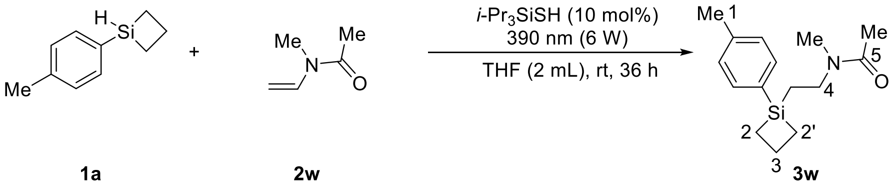

In an argon-filled glovebox, to a flame-dried screw-cap reaction tube equipped with a magnetic stir bar were added *i*-Pr_3SiSH (9.5 mg, 0.05 mmol), **1a** (97.2 mg, 0.6 mmol, 1.2 equiv.), **2w** (49.5 mg, 0.5 mmol, 1 equiv.) and THF (2.0 mL) sequentially. The tube was sealed with a screw cap equipped with a septum, removed from the glovebox. The reaction mixture was stirred at rt for 36 h under 6 W 390 nm LED lamps. The reaction mixture was concentrated under reduced pressure. The crude product was purified with column chromatography on silica gel (200~300 mesh) with PE/EA (10/1, v/v) as eluent to afford 120 mg of the title compound **3s** as a colorless oil (92% yield).

^{1}H NMR (400 MHz, $CDCl_3$, 25 ºC) δ 7.56–7.45 (m, 2H), 7.27–7.18 (m, 2H), 3.60–3.51 (m, 2H, H, H_4), 3.47–3.37 (m, 2H, H' , H_4), 2.92 (s, 3H, H), 2.90 (s, 3H, H'), 2.37 (s, 3H, H' , H_1), 2.36 (s, 3H, H, H_1), 2.25–2.12 (m, 2H, H_3), 2.02 (s, 3H, H'), 2.00 (s, 3H, H), 1.40–1.19 (m, 6H). ^{13}C NMR (151 MHz, $CDCl_3$, 25 ºC) δ 170.1 (C_5), 170.0 (C_5), 140.1, 139.6, 133.7, 133.5, 133.4, 132.5, 129.1, 128.9, 46.6 (C_4), 43.5 (C_4), 35.5, 32.5, 22.0, 21.6 (C_1), 21.6 (C_1), 21.2, 18.6 (C_3), 18.5 (C_3), 15.3, 13.9, 13.0 (C_2, $C_{2'}$), 12.9 (C_2, $C_{2'}$). ^{29}Si NMR (119 MHz, $CDCl_3$, 25 ºC) δ 11.9, 11.4. HRMS (ESI): (m/z) calcd for $C_{15}H_{24}NOSi^+$ (M+H$^+$), 262.1622; found, 262.1618.

9-(2-(1-(*p*-Tolyl)siletan-1-yl)ethyl)-9H-carbazole (3x)

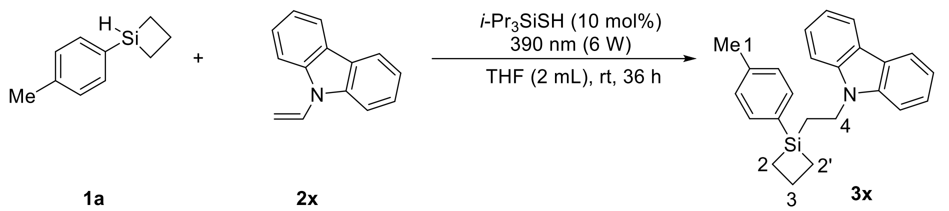

In an argon-filled glovebox, to a flame-dried screw-cap reaction tube equipped with a magnetic stir bar were added *i*-Pr_3SiSH (9.5 mg, 0.05 mmol), **1a** (97.2 mg, 0.6 mmol, 1.2 equiv.), **2x** (96.5 mg, 0.5 mmol, 1 equiv.) and THF (2.0 mL) sequentially. The tube was sealed with a screw cap equipped with a septum, removed from the glovebox. The reaction mixture was stirred at rt for 36 h under 6 W 390 nm LED lamps. The reaction mixture was concentrated under reduced pressure. The crude product was purified with column chromatography on silica gel (200~300 mesh) with PE/EA (20/1, v/v) as eluent to afford 163 mg of the title compound **3x** as a colorless oil (92% yield).

M.p. 78.1–80.2 °C. ^{1}H NMR (400 MHz, $CDCl_3$, 25 °C) δ 8.12 (d, J = 7.8 Hz, 2H), 7.56 (d, J = 7.6 Hz, 2H), 7.52–7.43 (m, 2H), 7.36 (d, J = 8.2 Hz, 2H), 7.32–7.21 (m, 4H), 4.60–4.31 (m, 2H, H_4), 2.43 (s, 3H, H_1), 2.31–2.18 (m, 2H, H_3), 1.77–1.62 (m, 2H), 1.44–1.17 (m, 4H, H_2, $H_{2'}$). ^{13}C NMR (151 MHz, $CDCl_3$, 25 °C) δ 140.0, 139.8, 133.7, 132.8, 129.2, 125.6, 123.2, 120.5, 118.9, 108.7, 38.6 (C_4), 21.7 (C_1), 18.7 (C_3), 15.5, 13.1(C_2, $C_{2'}$). ^{29}Si NMR (119 MHz, $CDCl_3$, 25 °C) δ 11.6. HRMS (APCI): (m/z) calcd for $C_{24}H_{26}NSi^+$ (M+H^+), 356.1829; found, 356.1817.

1-Hexyl-1-phenylsiletane (3y)

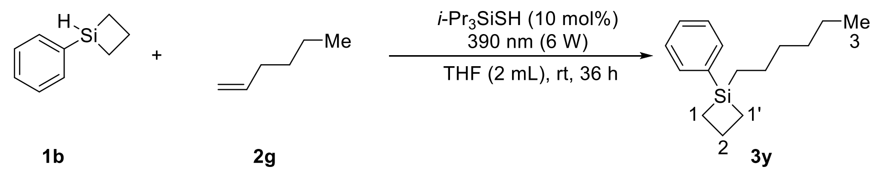

In an argon-filled glovebox, to a flame-dried screw-cap reaction tube equipped with a magnetic stir bar were added *i*-Pr_3SiSH (9.5 mg, 0.05 mmol), **1b** (97.2 mg, 0.6 mmol, 1.2 equiv.), **2g** (42 mg, 0.5 mmol, 1 equiv.) and THF (2.0 mL) sequentially. The tube was sealed with a screw cap equipped with a septum, removed from the glovebox. The reaction mixture was stirred at rt for 36 h under 6 W 390 nm LED lamps. The reaction mixture was concentrated under reduced pressure. The crude product was purified with column chromatography on silica gel (200~300 mesh) with PE as eluent to afford 108 mg of the title compound **3y** as a colorless oil (93% yield).

^{1}H NMR (400 MHz, $CDCl_3$, 25 °C) δ 7.67–7.59 (m, 2H), 7.44–7.35 (m, 3H), 2.27–2.08 (m, 2H, H_2), 1.55–1.44 (m, 2H), 1.43–1.34 (m, 2H), 1.33–1.18 (m, 8H), 1.06–0.99 (m, 2H), 0.93–0.85 (m, 3H, H_3). ^{13}C NMR (151 MHz, $CDCl_3$, 25 °C) δ 138.3, 133.8, 129.4, 128.0, 33.2, 31.7, 23.8, 22.7, 18.6 (C_2), 15.3, 14.3 (C_3), 13.1(C_1, $C_{1'}$). ^{29}Si NMR (119 MHz, $CDCl_3$, 25 °C) δ 14.1. HRMS (APCI): (m/z) calcd for $C_{15}H_{25}Si^+$ (M+H^+), 233.1720; found, 233.1719.

1-(4-Fluorophenyl)-1-hexylsiletane (3z)

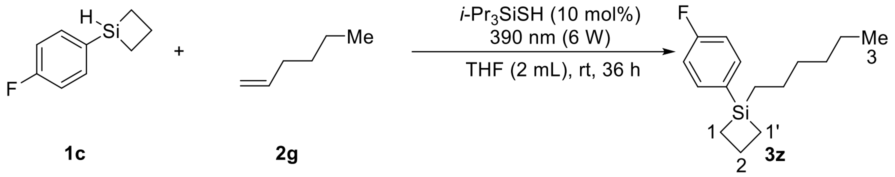

In an argon-filled glovebox, to a flame-dried screw-cap reaction tube equipped with a magnetic stir bar were added *i*-Pr_3SiSH (9.5 mg, 0.05 mmol), **1c** (99.6 mg, 0.6 mmol, 1.2 equiv.), **2g** (42 mg, 0.5 mmol, 1 equiv.) and THF (2.0 mL) sequentially. The tube was sealed with a screw cap equipped with a septum, removed from the glovebox. The reaction mixture was stirred at rt for 36 h under 6 W 390 nm LED lamps. The reaction mixture was concentrated under reduced pressure. The crude product was purified with column chromatography on silica gel (200~300 mesh) with PE as eluent to afford 110 mg of the title compound **3z** as a colorless oil (88% yield).

^{1}H NMR (400 MHz, $CDCl_3$, 25 °C) δ 7.65–7.54 (m, 2H), 7.16–7.01 (m, 2H), 2.27–2.06 (m, 2H, H_2), 1.52–1.42 (m, 2H), 1.42–1.34 (m, 2H), 1.33–1.26 (m, 4H), 1.27–1.19 (m, 4H), 1.05–0.96 (m, 2H), 0.94–0.84 (m, 3H, H_3). ^{13}C NMR (101 MHz, $CDCl_3$, 25 °C) δ 163.8 (d, J = 296.8 Hz), 135.7 (d, J = 7.4 Hz), 133.7, 115.2 (d, J = 19.6 Hz), 33.1, 31.7, 23.7, 22.7, 18.5 (C_2), 15.4, 14.3 (C_3), 13.2 (C_1, $C_{1'}$). ^{29}Si NMR (119 MHz, $CDCl_3$, 25 °C) δ 14.1. HRMS (APCI): (m/z) calcd for $C_{15}H_{24}FSi^+$ (M+H$^+$), 251.1626; found, 251.1628.

1-(4-(tert-butyl)phenyl)-1-hexylsiletane (3aa)

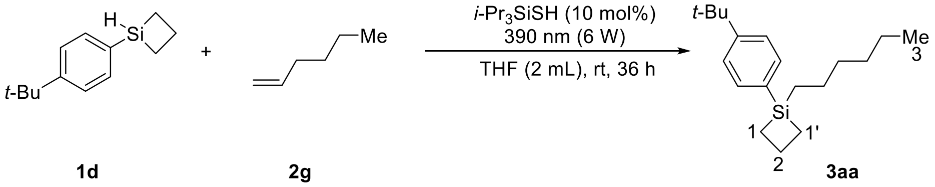

In an argon-filled glovebox, to a flame-dried screw-cap reaction tube equipped with a magnetic stir bar were added *i*-Pr_3SiSH (9.5 mg, 0.05 mmol), **1d** (122 mg, 0.6 mmol, 1.2 equiv.), **2g** (42 mg, 0.5 mmol, 1 equiv.) and THF (2.0 mL) sequentially. The tube was sealed with a screw cap equipped with a septum, removed from the glovebox. The reaction mixture was stirred at rt for 36 h under 6 W 390 nm LED lamps. The reaction mixture was concentrated under reduced pressure. The crude product was purified with column chromatography on silica gel (200~300 mesh) with PE as eluent to afford 116 mg of the title compound **3aa** as a colorless oil (81% yield).

^{1}H NMR (400 MHz, $CDCl_3$, 25 °C) δ 7.62–7.54 (m, 2H), 7.48–7.41 (m, 2H), 2.26–2.12 (m, 2H, H_2), 1.55–1.47 (m, 2H), 1.44–1.38 (m, 2H), 1.36 (s, 9H), 1.34–1.29 (m, 4H), 1.28–1.19 (m, 4H), 1.07–0.98 (m, 2H), 0.97–0.86 (m, 3H, H_3). ^{13}C NMR (151 MHz, $CDCl_3$, 25 °C) δ 152.4, 134.8, 133.7, 125.0, 34.8, 33.2, 31.7, 31.4, 23.8, 22.8, 18.6 (C_2), 15.2, 14.3 (C_3), 13.1 (C_1, $C_{1'}$). ^{29}Si NMR (119 MHz, $CDCl_3$, 25 °C) δ 13.9. HRMS (APCI): (m/z) calcd for $C_{19}H_{33}Si^+$ (M+H$^+$), 289.2346; found, 289.2338.

4-(1-Hexylsiletan-1-yl)-N,N-dimethylaniline (3ab)

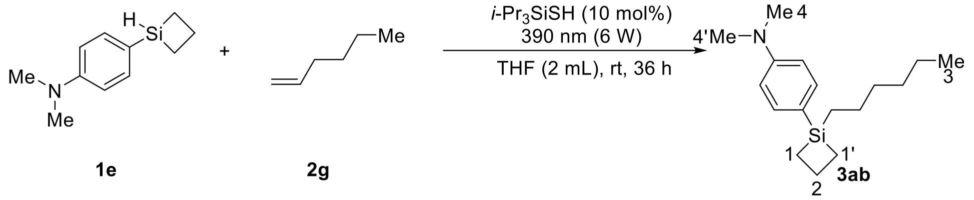

In an argon-filled glovebox, to a flame-dried screw-cap reaction tube equipped with a magnetic stir bar were added *i*-Pr_3SiSH (9.5 mg, 0.05 mmol), **1e** (115 mg, 0.6 mmol, 1.2 equiv.), **2g** (42 mg, 0.5 mmol, 1 equiv.) and THF (2.0 mL) sequentially. The tube was sealed with a screw cap equipped with a septum, removed from the glovebox. The reaction mixture was stirred at rt for 36 h under 6 W 390 nm LED lamps. The reaction mixture was concentrated under reduced pressure. The crude product was purified with column chromatography on silica gel (200~300 mesh) with PE as eluent to afford 106 mg of the title compound **3ab** as a colorless oil (77% yield).

^{1}H NMR (600 MHz, $CDCl_3$, 25 °C) δ 7.50 (d, *J* = 8.9 Hz, 2H), 6.78 (d, *J* = 8.8 Hz, 2H), 2.99 (s, 6H, H_4, $H_{4'}$), 2.22–2.08 (m, 2H, H_2), 1.54–1.45 (m, 2H), 1.43–1.35 (m, 2H), 1.35–1.26 (m, 4H), 1.26–1.14 (m, 4H), 1.00 (t, *J* = 9.8 Hz, 2H), 0.93–0.86 (m, 3H, H_3). ^{13}C NMR (151 MHz, $CDCl_3$, 25 °C) δ 151.4, 134.9, 123.2, 112.1, 40.3 (C_4, $C_{4'}$), 33.3, 31.7, 23.9, 22.8, 18.5 (C_2), 15.2, 14.3 (C_3), 13.4 (C_1, $C_{1'}$). ^{29}Si NMR (119 MHz, $CDCl_3$, 25 °C) δ 8.4. HRMS (APCI): (m/z) calcd for $C_{17}H_{30}NSi^+$ (M+H$^+$), 276.2142; found, 276.2133.

3-((1-(4-(dimethylamino)phenyl)siletan-1-yl)methyl)cyclobutane-1-carbonitrile (3ac)

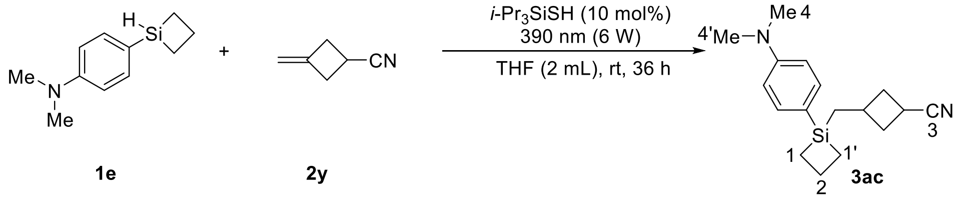

In an argon-filled glovebox, to a flame-dried screw-cap reaction tube equipped with a magnetic stir bar were added *i*-Pr_3SiSH (9.5 mg, 0.05 mmol), **1e** (115 mg, 0.6 mmol, 1.2 equiv.), **2y** (46 mg, 0.5 mmol, 1 equiv.) and THF (2.0 mL) sequentially. The tube was sealed with a screw cap equipped with a septum, removed from the glovebox. The reaction mixture was stirred at rt for 36 h under 6 W 390 nm LED lamps. The reaction mixture was concentrated under reduced pressure. The crude product was purified with column chromatography on silica gel (200~300 mesh) with PE/EA (20/1, v/v) as eluent to afford 109 mg of the title compound **3ac** as a colorless oil (77% yield).

^{1}H NMR (600 MHz, $CDCl_3$, 25 °C) δ 7.45 (d, *J* = 7.2 Hz, 2H), 6.76 (d, *J* = 8.4 Hz, 2H), 2.99 (s, 6H, H_4, $H_{4'}$), 2.90–2.77 (m, 1H), 2.58–2.43 (m, 3H), 2.20–2.02 (m, 4H), 1.31–1.20 (m, 4H), 1.18–1.06 (m, 2H). ^{13}C NMR (151 MHz, $CDCl_3$, 25 °C) δ 151.5, 134.7, 122.4, 121.9 (C_3), 112.0, 40.2 (C_4, $C_{4'}$), 36.5, 35.1, 30.6, 24.0, 18.6, 18.5 (C_2), 13.6 (C_1, $C_{1'}$). ^{29}Si NMR (119 MHz, $CDCl_3$, 25 °C) δ 10.7. HRMS (APCI): (m/z) calcd for $C_{17}H_{25}N_2Si^+$ (M+H$^+$), 285.1782; found, 285.1781.

(*1R,2S,5R*)-2-isopropyl-5-methylcyclohexyl 5-(1-(p-tolyl)siletan-1-yl)pentanoate (3ad)

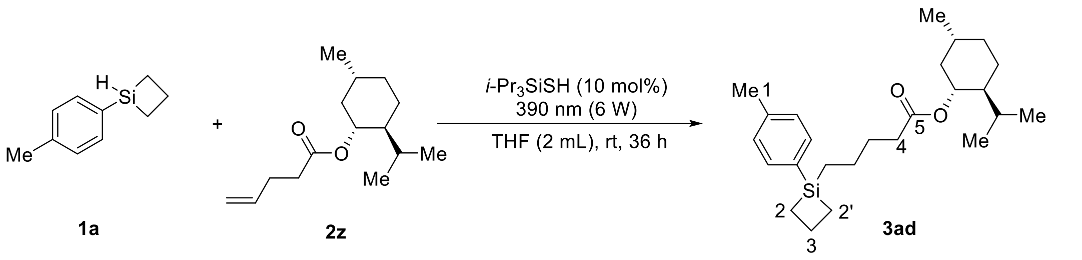

In an argon-filled glovebox, to a flame-dried screw-cap reaction tube equipped with a magnetic stir bar were added *i*-Pr_3SiSH (9.5 mg, 0.05 mmol), **1a** (97 mg, 0.6 mmol, 1.2 equiv.), **2z** (119 mg, 0.5 mmol, 1 equiv.) and THF (2.0 mL) sequentially. The tube was sealed with a screw cap equipped with a septum, removed from the glovebox. The reaction mixture was stirred at rt for 36 h under 6 W 390 nm LED lamps. The reaction mixture was concentrated under reduced pressure. The crude product was purified with column chromatography on silica gel (200~300 mesh) with PE/EA (20/1, v/v) as eluent to afford 150 mg of the title compound **3ad** as a colorless oil (75% yield).

^{1}H NMR (400 MHz, $CDCl_3$, 25 ºC) δ 7.51 (d, J = 7.7 Hz, 2H), 7.23 (d, J = 7.5 Hz, 2H), 4.78–4.55 (m, 1H), 2.38 (s, 3H, H_1), 2.31 (t, J = 7.4 Hz, 2H, H_4), 2.23–2.08 (m, 2H, H_3), 2.02–1.93 (m, 1H), 1.91–1.80 (m, 1H), 1.77–1.63 (m, 4H), 1.57–1.44 (m, 3H), 1.42–1.32 (m, 1H), 1.28–1.17 (m, 4H), 1.06–0.98 (m, 4H), 0.94–0.85 (m, 7H), 0.76 (d, J = 7.0 Hz, 3H). ^{13}C NMR (101 MHz, $CDCl_3$, 25 ºC) δ 173.5 (C_5), 139.4, 134.2, 133.8, 128.9, 74.0, 47.2, 41.1, 34.6, 34.4, 31.5, 28.8, 26.4, 23.5, 23.5, 22.2, 21.6 (C_1), 20.9, 18.5 (C_3), 16.4, 15.1, 13.0 (C_2, $C_{2'}$). ^{29}Si NMR (119 MHz, $CDCl_3$, 25 ºC) δ 13.7. HRMS (APCI): (m/z) calcd for $C_{25}H_{41}O_2Si^+$ (M+H^+), 401.2870; found, 401.2871.

(*3S,8R,9S,10R,13S,14S*)-10,13-dimethyl-17-oxo-2,3,4,7,8,9,10,11,12,13,14,15,16,17-tetradecahydro-1H-cyclopenta[a]phenanthren-3-yl 5-(1-(4-(dimethylamino)phenyl)siletan-1-yl)pentanoate (3ae)

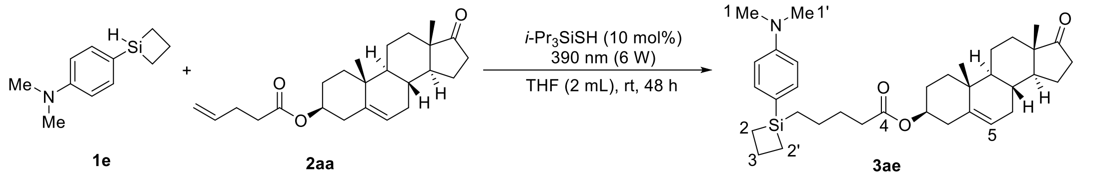

In an argon-filled glovebox, to a flame-dried screw-cap reaction tube equipped with a magnetic stir bar were added *i*-Pr_3SiSH (9.5 mg, 0.05 mmol), **1e** (172.5 mg, 0.75 mmol, 1.5 equiv.), **2aa** (185 mg, 0.5 mmol, 1 equiv.) and THF (2.0 mL) sequentially. The tube was sealed with a screw cap equipped with a septum, removed from the glovebox. The reaction mixture was stirred at rt for 48 h under 6 W 390 nm LED lamps. The reaction mixture was concentrated under reduced pressure. The crude product was purified with column chromatography on silica gel (200~300 mesh) with PE/EA (10/1, v/v) as eluent to afford 207 mg of the title compound **3ae** as a colorless oil (74% yield).

^{1}H NMR (400 MHz, $CDCl_3$, 25 ºC) δ 7.47 (d, *J* = 6.9 Hz, 2H), 6.75 (d, *J* = 8.7 Hz, 2H), 5.50–5.23 (m, 1H, H_5), 4.68–4.50 (m, 1H), 2.97 (s, 6H, H_1, $H_{1'}$), 2.54–2.40 (m, 1H), 2.36–2.24 (m, 4H), 2.19–2.03 (m, 4H), 2.00–1.91 (m, 1H), 1.90–1.79 (m, 3H), 1.75–1.60 (m, 6H), 1.58–1.44 (m, 4H), 1.36–1.24 (m, 3H), 1.23–1.13 (m, 4H, H_2, $H_{2'}$), 1.08–0.97 (m, 6H), 0.88 (s, 3H). ^{13}C NMR (151 MHz, $CDCl_3$, 25 ºC) δ 221.3, 173.4 (C_4), 151.4, 140.1, 134.9, 122.7, 121.9 (C_5), 112.1, 73.6, 51.8, 50.3, 47.7, 40.3 (C_1), 38.2, 37.1, 36.8, 35.9, 34.5, 31.6, 31.5, 30.9, 28.8, 27.8, 23.6, 22.0, 20.4, 19.5, 18.4 (C_3), 14.9, 13.7, 13.4 (C_2, $C_{2'}$).

^{29}Si NMR (119 MHz, $CDCl_3$, 25 ºC) δ 9.7. HRMS (APCI): (m/z) calcd for $C_{35}H_{52}NO_3Si^+$ (M+H$^+$), 562.3711; found, 562.3708.

1-(4-Phenylbutyl)siletane (3af)

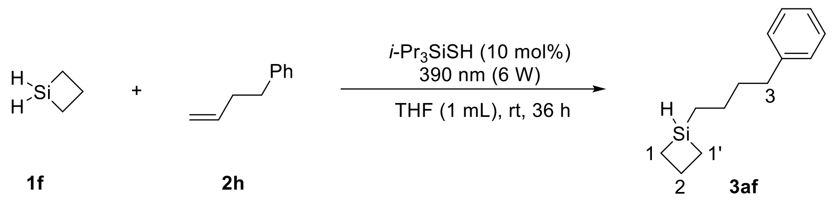

In an argon-filled glovebox, to a flame-dried screw-cap reaction tube equipped with a magnetic stir bar were added *i*-Pr_3SiSH (9.5 mg, 0.05 mmol), **2h** (66 mg, 0.5 mmol, 1 equiv.), THF (1 mL) and **1f** (2 mL, 0.38 M, 1.5 equiv.) sequentially. The tube was sealed with a screw cap equipped with a septum, removed from the glovebox. The reaction mixture was stirred at rt for 36 h under 6 W 390 nm LED lamps. The reaction mixture was concentrated under reduced pressure. The crude product was purified with column chromatography on silica gel (100~200 mesh) with PE as eluent to afford 92 mg of the title compound **3af** as a colorless oil (90% yield).

^{1}H NMR (400 MHz, $CDCl_3$, 25 ºC) δ 7.37–7.26 (m, 2H), 7.24–7.13 (m, 3H), 4.75–4.56 (m, 1H, SiH), 2.65 (t, J = 8 Hz, 2H, H_3), 2.28–2.05 (m, 2H, H_2), 1.78–1.63 (m, 2H), 1.60–1.44 (m, 2H,), 1.20–1.08 (m, 2H, H_1), 1.08–0.96 (m, 2H, $H_{1'}$), 0.96–0.88 (m, 2H). ^{13}C NMR (151 MHz, $CDCl_3$, 25 ºC) δ 142.8, 128.5, 128.4, 125.7, 35.8 (C_3), 34.8, 24.0, 20.0 (C_2), 14.2, 11.6 (C_1, $C_{1'}$). ^{29}Si NMR (119 MHz, $CDCl_3$, 25 ºC) δ 3.5. HRMS (APCI): (m/z) calcd for $C_{13}H_{21}Si^+$ (M+H$^+$), 205.1407; found, 205.1410.

5-(Siletan-1-yl)pentanoic acid (3ag)

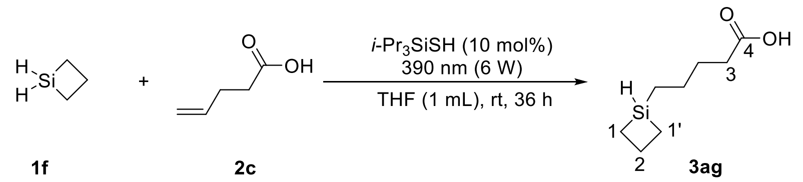

In an argon-filled glovebox, to a flame-dried screw-cap reaction tube equipped with a magnetic stir bar were added *i*-Pr_3SiSH (9.5 mg, 0.05 mmol), **2c** (50 mg, 0.5 mmol, 1 equiv.), THF (1 mL) and **1f** (2 mL, 0.38 M, 1.5 equiv.) sequentially. The tube was sealed with a screw cap equipped with a septum, removed from the glovebox. The reaction mixture was stirred at rt for 36 h under 6 W 390 nm LED lamps. The reaction mixture was concentrated under reduced pressure. The crude product was purified with column chromatography on silica gel (100~200 mesh) with CH_2Cl_2/EA (1/1, v/v) as eluent to afford 78 mg of the title compound **3ag** as a colorless oil (91% yield).

^{1}H NMR (600 MHz, $CDCl_3$, 25 ºC) δ 4.70–4.58 (m, 1H, SiH), 2.37 (t, J = 7.5 Hz, 2H, H_3), 2.23–2.05 (m, 2H, H_2), 1.74–1.64 (m, 2H), 1.57–1.44 (m, 2H), 1.16–1.07 (m, 2H, H_1), 1.05–0.95 (m, 2H, $H_{1'}$), 0.92–0.84 (m, 2H). ^{13}C NMR (151 MHz, $CDCl_3$, 25 ºC) δ 180.3 (C_4), 33.9 (C_3), 27.9, 23.8, 20.0 (C_2), 14.0, 11.5 (C_1, $C_{1'}$). ^{29}Si NMR (119 MHz, $CDCl_3$, 25 ºC) δ 3.3. HRMS (APCI): (m/z) calcd for $C_8H_{17}O_2Si^+$ (M+H$^+$), 173.0992; found, 173.0988.

1-(6-Chlorohexyl)siletane (3ah)

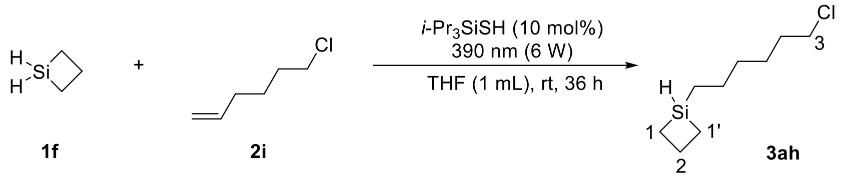

In an argon-filled glovebox, to a flame-dried screw-cap reaction tube equipped with a magnetic stir bar were added *i*-Pr_3SiSH (9.5 mg, 0.05 mmol), **2i** (59 mg, 0.5 mmol, 1 equiv.), THF (1 mL) and **1f** (2 mL, 0.38 M, 1.5 equiv.) sequentially. The tube was sealed with a screw cap equipped with a septum, removed from the glovebox. The reaction mixture was stirred at rt for 36 h under 6 W 390 nm LED lamps. The reaction mixture was concentrated under reduced pressure. The crude product was purified with column chromatography on silica gel (100~200 mesh) with PE as eluent to afford 69 mg of the title compound **3ah** as a colorless oil (73% yield).

^{1}H NMR (600 MHz, $CDCl_3$, 25 ℃) δ 4.69–4.58 (m, 1H, SiH), 3.54 (t, *J* = 6.8 Hz, 2H, H_3), 2.24–2.02 (m, 2H, H_2), 1.83–1.72 (m, 2H), 1.51–1.42 (m, 4H), 1.42–1.34 (m, 2H), 1.15–1.08 (m, 2H, H_1), 1.06–0.96 (m, 2H, $H_{1'}$), 0.92–0.84 (m, 2H). ^{13}C NMR (151 MHz, $CDCl_3$, 25 ℃) δ 45.3 (C_3), 32.7, 32.2, 26.7, 24.1, 20.0 (C_2), 14.3, 11.6 (C_1, $C_{1'}$). ^{29}Si NMR (119 MHz, $CDCl_3$, 25 ℃) δ 3.5. HRMS (APCI): (m/z) calcd for $C_9H_{23}ClNSi^+$ (M+NH_4^+), 208.1283; found, 208.1288.

6-(Siletan-1-yl)hexan-1-ol (3ai)

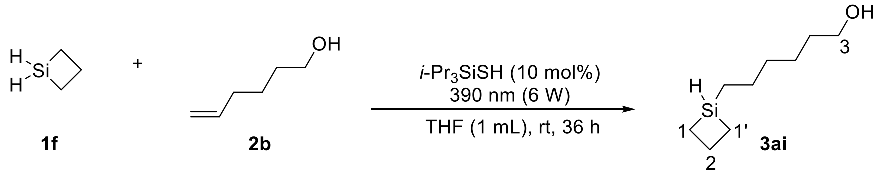

In an argon-filled glovebox, to a flame-dried screw-cap reaction tube equipped with a magnetic stir bar were added *i*-Pr_3SiSH (9.5 mg, 0.05 mmol), **2b** (50 mg, 0.5 mmol, 1 equiv.), THF (1 mL) and **1f** (2 mL, 0.38 M, 1.5 equiv.) sequentially. The tube was sealed with a screw cap equipped with a septum, removed from the glovebox. The reaction mixture was stirred at rt for 36 h under 6 W 390 nm LED lamps. The reaction mixture was concentrated under reduced pressure. The crude product was purified with column chromatography on silica gel (100~200 mesh) with PE/EA (5/1, v/v) as eluent to afford 72 mg of the title compound **3ai** as a colorless oil (84% yield).

^{1}H NMR (400 MHz, $CDCl_3$, 25 ℃) δ 4.73–4.56 (m, 1H, SiH), 3.63 (t, *J* = 6.6 Hz, 2H, H_3), 2.26–1.96 (m, 2H, H_2), 1.61–1.52 (m, 2H), 1.51–1.42 (m, 2H), 1.42–1.31 (m, 4H), 1.15–0.94 (m, 4H, H_1, $H_{1'}$), 0.90–0.80 (m, 2H). ^{13}C NMR (151 MHz, $CDCl_3$, 25 ℃) δ 63.1 (C_3), 32.8, 32.7, 25.6, 24.2, 19.9 (C_2), 14.3, 11.5 (C_1, $C_{1'}$). ^{29}Si NMR (119 MHz, $CDCl_3$, 25 ℃) δ 3.5. HRMS (APCI): (m/z) calcd for $C_9H_{21}OSi^+$ (M+H^+), 173.1356; found, 173.1355.

6-(Siletan-1-yl)hexyl acetate (3aj)

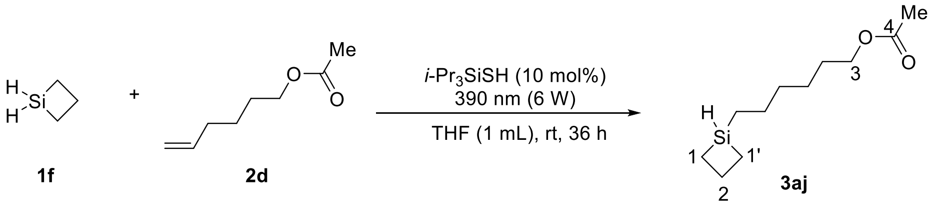

In an argon-filled glovebox, to a flame-dried screw-cap reaction tube equipped with a magnetic stir bar were added *i*-Pr_3SiSH (9.5 mg, 0.05 mmol), **2d** (71 mg, 0.5 mmol, 1 equiv.), THF (1 mL) and **1f** (2 mL, 0.38 M, 1.5 equiv.) sequentially. The tube was sealed with a screw cap equipped with a septum, removed from the glovebox. The reaction mixture was stirred at rt for 36 h under 6 W 390 nm LED lamps. The reaction mixture was concentrated under reduced pressure. The crude product was purified with column chromatography on silica gel (100~200 mesh) with PE/EA (20/1, v/v) as eluent to afford 86 mg of the title compound **3aj** as a colorless oil (81% yield).

^{1}H NMR (400 MHz, $CDCl_3$, 25 ºC) δ 4.76–4.54 (m, 1H, SiH), 4.05 (t, J = 6.8 Hz, 2H, H_3), 2.26–2.09 (m, 2H, H_2), 2.04 (s, 3H), 1.69–1.58 (m, 2H), 1.51–1.43 (m, 2H), 1.42–1.31 (m, 4H), 1.16–1.06 (m, 2H, H_1), 1.05–0.94 (m, 2H, $H_{1'}$), 0.90–0.81 (m, 2H). ^{13}C NMR (151 MHz, $CDCl_3$, 25 ºC) δ 171.4 (C_4), 64.8 (C_3), 32.6, 28.6, 25.8, 24.2, 21.3, 20.0 (C_2), 14.3, 11.6 (C_1, $C_{1'}$). ^{29}Si NMR (119 MHz, $CDCl_3$, 25 ºC) δ 3.6. HRMS (APCI): (m/z) calcd for $C_{11}H_{23}O_2Si^+$ (M+H$^+$), 215.1462; found, 215.1459.

4-(Siletan-1-yl)butyl benzoate (3ak)

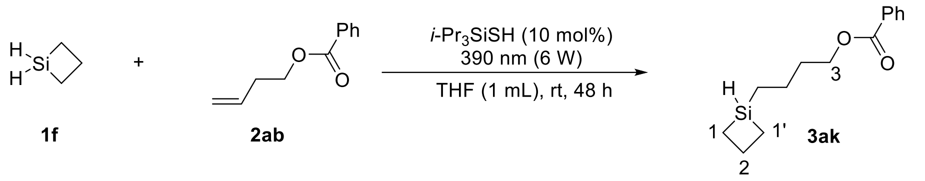

In an argon-filled glovebox, to a flame-dried screw-cap reaction tube equipped with a magnetic stir bar were added *i*-Pr_3SiSH (9.5 mg, 0.05 mmol), **2ab** (88 mg, 0.5 mmol, 1 equiv.), THF (1 mL) and **1f** (2 mL, 0.38 M, 1.5 equiv.) sequentially. The tube was sealed with a screw cap equipped with a septum, removed from the glovebox. The reaction mixture was stirred at rt for 48 h under 6 W 390 nm LED lamps. The reaction mixture was concentrated under reduced pressure. The crude product was purified with column chromatography on silica gel (100~200 mesh) with PE/EA (20/1, v/v) as eluent to afford 89 mg of the title compound **3ak** as a colorless oil (72% yield).

^{1}H NMR (400 MHz, $CDCl_3$, 25 ºC) δ 8.07–8.01 (m, 2H), 7.59–7.52 (m, 1H), 7.48–7.39 (m, 2H), 4.72–4.62 (m, 1H, SiH), 4.34 (t, J = 6.5 Hz, 2H, H_3), 2.26–2.04 (m, 2H, H_2), 1.92–1.76 (m, 2H), 1.68–1.60 (m, 2H), 1.19–1.09 (m, 2H, H_1), 1.08–0.97 (m, 2H, $H_{1'}$), 0.99–0.89 (m, 2H). ^{13}C NMR (151 MHz, $CDCl_3$, 25 ºC) δ 166.8, 132.9, 130.6, 129.7, 128.5, 64.8 (C_3), 31.9, 20.9, 20.0 (C_2), 13.9, 11.5 (C_1, $C_{1'}$). ^{29}Si NMR (119 MHz, $CDCl_3$, 25 ºC) δ 3.5. HRMS (ESI): (m/z) calcd for $C_{14}H_{21}O_2Si^+$ (M+H$^+$), 249.1305; found, 249.1298.

1-(3-(4,4,5,5-tetramethyl-1,3,2-dioxaborolan-2-yl)propyl)siletane (3al)

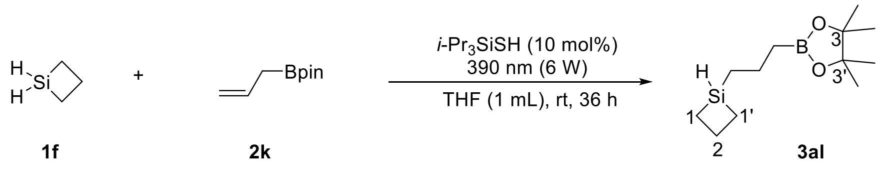

In an argon-filled glovebox, to a flame-dried screw-cap reaction tube equipped with a magnetic stir bar were added *i*-Pr_3SiSH (9.5 mg, 0.05 mmol), **2k** (84 mg, 0.5 mmol, 1 equiv.), THF (1 mL) and **1f** (2 mL, 0.38 M, 1.5 equiv.) sequentially. The tube was sealed with a screw cap equipped with a septum, removed from the glovebox. The reaction mixture was stirred at rt for 36 h under 6 W 390 nm LED lamps. The reaction mixture was concentrated under reduced pressure. The crude product was purified with column

chromatography on silica gel (100~200 mesh) with PE/EA (100/1, v/v) as eluent to afford 102 mg of the title compound **3al** as a colorless oil (85% yield).

^{1}H NMR (600 MHz, $CDCl_3$, 25 ºC) δ 4.67–4.58 (m, 1H, SiH), 2.23–2.02 (m, 2H, H_2), 1.63–1.54 (m, 2H), 1.24 (s, 12H), 1.12–1.05 (m, 2H, H_1), 1.04–0.96 (m, 2H, $H_{1'}$), 0.92–0.82 (m, 4H). ^{13}C NMR (151 MHz, $CDCl_3$, 25 ºC) δ 83.1 (C_3), 24.9, 20.1 (C_2), 18.9, 17.5, 11.6 (C_1, $C_{1'}$). ^{29}Si NMR (119 MHz, $CDCl_3$, 25 ºC) δ 3.4. HRMS (APCI): (m/z) calcd for $C_{12}H_{26}BO_2Si^+$ (M+H$^+$), 241.1790; found, 241.1791.

9-(2-(Siletan-1-yl)ethyl)-9H-carbazole (3am)

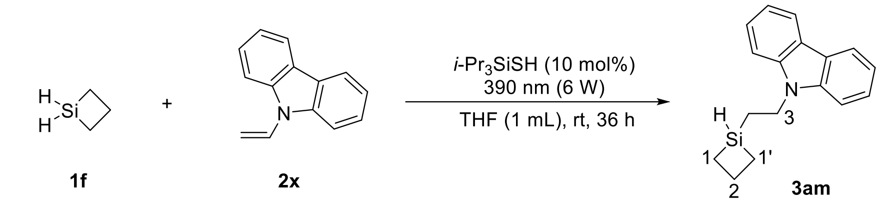

In an argon-filled glovebox, to a flame-dried screw-cap reaction tube equipped with a magnetic stir bar were added *i*-Pr$_3$SiSH (9.5 mg, 0.05 mmol), **2x** (96 mg, 0.5 mmol, 1 equiv.), THF (1 mL) and **1f** (2 mL, 0.38 M, 1.5 equiv.) sequentially. The tube was sealed with a screw cap equipped with a septum, removed from the glovebox. The reaction mixture was stirred at rt for 36 h under 6 W 390 nm LED lamps. The reaction mixture was concentrated under reduced pressure. The crude product was purified with column chromatography on silica gel (100~200 mesh) with PE/EA (20/1, v/v) as eluent to afford 99 mg of the title compound **3am** as a colorless oil (75% yield).

^{1}H NMR (600 MHz, $CDCl_3$, 25 ºC) δ 8.11 (d, *J* = 7.8 Hz, 2H), 7.51–7.45 (m, 2H), 7.41 (d, *J* = 9.1 Hz, 2H), 7.28–7.20 (m, 2H), 4.78–4.72 (m, 1H, SiH), 4.55–4.47 (m, 2H, H_3), 2.22–2.11 (m, 2H, H_2), 1.57–1.52 (m, 2H), 1.16–1.08 (m, 2H, H_1), 1.03–0.92 (m, 2H, $H_{1'}$). ^{13}C NMR (151 MHz, $CDCl_3$, 25 ºC) δ 139.9, 125.7, 123.2, 120.6, 118.9, 108.7, 38.8 (C_3), 20.2 (C_2), 15.0, 11.3 (C_1, $C_{1'}$). ^{29}Si NMR (119 MHz, $CDCl_3$, 25 ºC) δ 1.9. HRMS (APCI): (m/z) calcd for $C_{17}H_{20}NSi^+$ (M+H$^+$), 266.1360; found, 266.1355.

(*1R,2S,5R*)-2-isopropyl-5-methylcyclohexyl 5-(siletan-1-yl)pentanoate (3an)

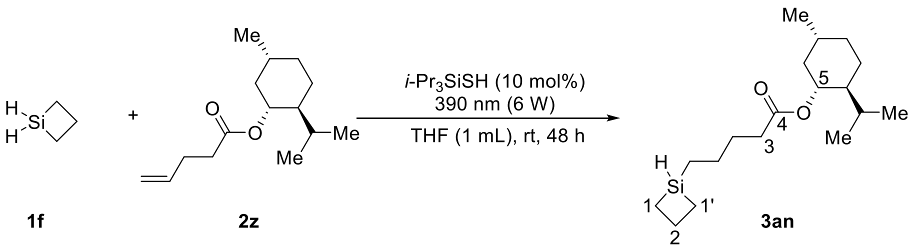

In an argon-filled glovebox, to a flame-dried screw-cap reaction tube equipped with a magnetic stir bar were added *i*-Pr$_3$SiSH (9.5 mg, 0.05 mmol), **2z** (119 mg, 0.5 mmol, 1 equiv.), THF (1 mL) and **1f** (2 mL, 0.38 M, 1.5 equiv.) sequentially. The tube was sealed with a screw cap equipped with a septum, removed from the glovebox. The reaction mixture was stirred at rt for 48 h under 6 W 390 nm LED lamps. The reaction mixture was concentrated under reduced pressure. The crude product was purified with column chromatography on silica gel (100~200 mesh) with PE/EA (20/1, v/v) as eluent to afford 133 mg of the title compound **3an** as a colorless oil (86% yield).

^{1}H NMR (400 MHz, $CDCl_3$, 25 ºC) δ 4.74–4.55 (m, 2H, SiH, H_5), 2.30 (t, *J* = 7.4 Hz, 2H, H_3), 2.21–2.07 (m, 2H, H_2), 2.02–1.93 (m, 1H), 1.90–1.81 (m, 1H), 1.75–1.62 (m, 4H), 1.54–1.42 (m, 3H), 1.40–1.31 (m, 1H), 1.17–1.05 (m, 3H), 1.04–0.94 (m, 3H), 0.92–0.84 (m, 9H), 0.75 (d, *J* = 7.0 Hz, 3H). ^{13}C NMR

(151 MHz, $CDCl_3$, 25 ºC) δ 173.4 (C_4), 74.1 (C_5), 47.2, 41.1, 34.6, 34.4, 31.5, 28.4, 26.4, 23.9, 23.5, 22.2, 20.9, 20.0, 16.4, 14.1, 11.5 (C_1, $C_{1'}$). ^{29}Si NMR (119 MHz, $CDCl_3$, 25 ºC) δ 3.5. HRMS (APCI): (m/z) calcd for $C_{18}H_{35}O_2Si^+$ (M+H$^+$), 311.2401; found, 311.2405.

1,6-di(Siletan-1-yl)hexane (3ao)

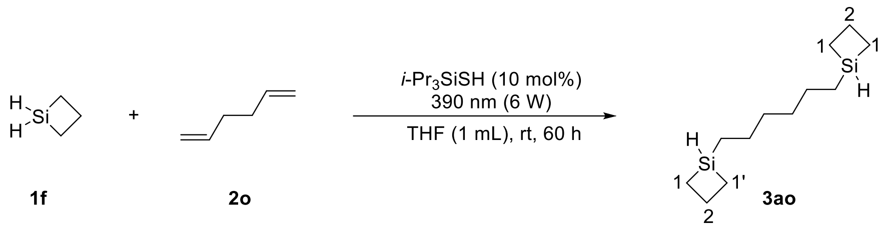

In an argon-filled glovebox, to a flame-dried screw-cap reaction tube equipped with a magnetic stir bar were added *i*-Pr_3SiSH (9.5 mg, 0.05 mmol), **2o** (41 mg, 0.5 mmol, 1 equiv.), THF (1 mL) and **1f** (4 mL, 0.38 M, 3 equiv.) sequentially. The tube was sealed with a screw cap equipped with a septum, removed from the glovebox. The reaction mixture was stirred at rt for 60 h under 6 W 390 nm LED lamps. The reaction mixture was concentrated under reduced pressure. The crude product was purified with column chromatography on silica gel (100~200 mesh) with PE as eluent to afford 87 mg of the title compound **3ao** as a colorless oil (77% yield).

^{1}H NMR (600 MHz, $CDCl_3$, 25 ºC) δ 4.69–4.57 (m, 2H, SiH), 2.30–1.93 (m, 4H, H_2), 1.49–1.40 (m, 4H), 1.39–1.31 (m, 4H), 1.15–1.07 (m, 4H, H_1), 1.04–0.93 (m, 4H, $H_{1'}$), 0.90–0.82 (m, 4H). ^{13}C NMR (151 MHz, $CDCl_3$, 25 ºC) δ 32.7, 24.2, 20.0 (C_2), 14.4, 11.6 (C_1, $C_{1'}$). ^{29}Si NMR (119 MHz, $CDCl_3$, 25 ºC) δ −1.7. HRMS (APCI): (m/z) calcd for $C_{12}H_{27}Si_2^+$ (M+H$^+$), 227.1646; found, 227.1647.

1-Cyclooctylsiletane (3ap)

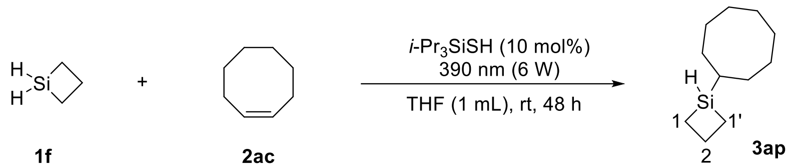

In an argon-filled glovebox, to a flame-dried screw-cap reaction tube equipped with a magnetic stir bar were added *i*-Pr_3SiSH (9.5 mg, 0.05 mmol), **2ac** (55 mg, 0.5 mmol, 1 equiv.), THF (1 mL) and **1f** (3 mL, 0.38 M, 2.28 equiv.) sequentially. The tube was sealed with a screw cap equipped with a septum, removed from the glovebox. The reaction mixture was stirred at rt for 48 h under 6 W 390 nm LED lamps. The reaction mixture was concentrated under reduced pressure. The crude product was purified with column chromatography on silica gel (100~200 mesh) with PE as eluent to afford 45 mg of the title compound **3ap** as a colorless oil (50% yield).

^{1}H NMR (400 MHz, $CDCl_3$, 25 ºC) δ 4.59–4.35 (m, 1H, SiH), 2.24–2.01 (m, 2H, H_2), 1.85–1.66 (m, 4H), 1.66–1.39 (m, 10H), 1.18–0.94 (m, 5H). ^{13}C NMR (151 MHz, $CDCl_3$, 25 ºC) δ 27.6, 27.5, 27.1, 26.8, 22.9, 19.5(C_2), 10.8 (C_1, $C_{1'}$). ^{29}Si NMR (119 MHz, $CDCl_3$, 25 ºC) δ 1.7. HRMS (APCI): (m/z) calcd for $C_{11}H_{23}Si^+$ (M+H$^+$), 183.1564; found, 183.1568.

1-(2-methyl-4-phenylbutyl)siletane (3aq)

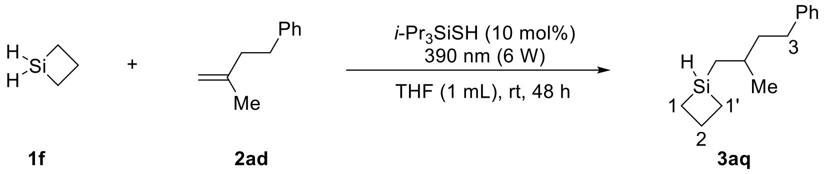

In an argon-filled glovebox, to a flame-dried screw-cap reaction tube equipped with a magnetic stir bar were added *i*-Pr_3SiSH (9.5 mg, 0.05 mmol), **2ad** (73 mg, 0.5 mmol, 1 equiv.), THF (1 mL) and **1f** (3 mL, 0.38 M, 2.28 equiv.) sequentially. The tube was sealed with a screw cap equipped with a septum, removed from the glovebox. The reaction mixture was stirred at rt for 48 h under 6 W 390 nm LED lamps. The reaction mixture was concentrated under reduced pressure. The crude product was purified with column chromatography on silica gel (100~200 mesh) with PE as eluent to afford 73 mg of the title compound **3aq** as a colorless oil (67% yield).

^{1}H NMR (600 MHz, $CDCl_3$, 25 ℃) δ 7.30–7.24 (m, 2H), 7.17 (d, *J* = 7.0 Hz, 3H), 4.74–4.55 (m, 1H, SiH), 2.72–2.51 (m, 2H, H_3), 2.27–2.15 (m, 1H, H_2), 2.15–2.04 (m, 1H, H_2), 1.84–1.72 (m, 1H), 1.70–1.61 (m, 1H), 1.59–1.51 (m, 1H), 1.17–1.09 (m, 2H), 1.07–0.95 (m, 6H), 0.89–0.80 (m, 1H). ^{13}C NMR (151 MHz, $CDCl_3$, 25 ℃) δ 142.9, 128.5, 128.4, 125.7, 41.9, 33.7 (C_3), 30.1, 22.9, 22.6, 20.1, 12.5(C_1), 12.3($C_{1'}$). ^{29}Si NMR (119 MHz, $CDCl_3$, 25 ℃) δ 1.5. HRMS (APCI): (m/z) calcd for $C_{14}H_{23}Si^+$ (M+H$^+$), 219.1564; found, 219.1570.

2-(1-(4-Phenylbutyl)siletan-1-yl)ethyl propionate (4aa)

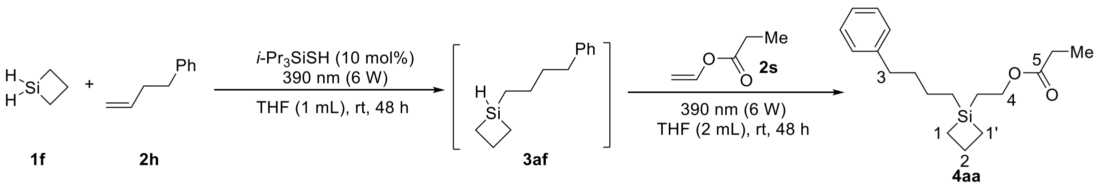

In an argon-filled glovebox, to a flame-dried screw-cap reaction tube equipped with a magnetic stir bar were added *i*-Pr_3SiSH (14.2 mg, 0.075 mmol), **2h** (99 mg, 0.75 mmol), THF (1 mL) and **1f** (3 mL, 0.38 M) sequentially. The tube was sealed with a screw cap equipped with a septum, removed from the glovebox. The reaction mixture was stirred at rt for 48 h under 6 W 390 nm LED lamps to ensure **2n** was consumed. The reaction mixture was concentrated under reduced pressure and pumped under the oil pump for 10 minutes to make sure **1f** was removed. Then the reaction tube containing **3af** was transferred to the glove box and **2s** (50 mg, 0.5 mmol) and THF (2 mL) were added to the tube. The tube was sealed with a screw cap equipped with a septum, removed from the glovebox. The reaction mixture was stirred at rt for 48 h under 6 W 390 nm LED lamps. The reaction mixture was concentrated under reduced pressure. The crude product was purified with column chromatography on silica gel (200~300 mesh) with PE/EA (20/1, v/v) as eluent to afford 131 mg of the title compound **4aa** as a colorless oil (86% yield).

^{1}H NMR (600 MHz, $CDCl_3$, 25 ℃) δ 7.24–7.16 (m, 2H), 7.13–7.06 (m, 3H), 4.24–4.20 (t, *J* = 8.3 Hz, 2H, H_4), 2.55 (t, *J* = 7.8 Hz, 2H, H_3), 2.23 (q, *J* = 7.6 Hz, 2H), 2.09–1.87 (m, 2H, H_2), 1.66–1.57 (m, 2H), 1.46–1.35 (m, 2H), 1.14 (t, *J* = 8.3 Hz, 2H), 1.06 (t, *J* = 7.6 Hz, 3H), 0.94 (t, *J* = 8.3 Hz, 4H, H_1, $H_{1'}$), 0.74 (t, *J* = 8.9 Hz, 2H). ^{13}C NMR (151 MHz, $CDCl_3$, 25 ℃) δ 174.7 (C_5), 142.7, 128.5, 128.4, 125.7, 62.2 (C_4), 35.8 (C_3), 35.2, 27.8, 23.4, 18.6 (C_2), 16.0, 15.3, 12.3 (C_1, $C_{1'}$), 9.3. ^{29}Si NMR (119 MHz, $CDCl_3$, 25 ℃) δ 19.7. HRMS (APCI): (m/z) calcd for $C_{18}H_{29}O_2Si^+$ (M+H$^+$), 305.1931; found, 305.1925.

1-(6-Chlorohexyl)-1-(4-phenylbutyl)siletane (4ab)

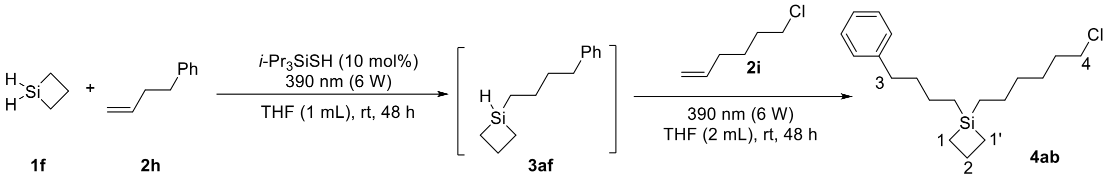

In an argon-filled glovebox, to a flame-dried screw-cap reaction tube equipped with a magnetic stir bar were added *i*-Pr_3SiSH (14.2 mg, 0.075 mmol), **2h** (99 mg, 0.75 mmol), THF (1 mL) and **1f** (3 mL, 0.38 M) sequentially. The tube was sealed with a screw cap equipped with a septum, removed from the glovebox. The reaction mixture was stirred at rt for 48 h under 6 W 390 nm LED lamps to ensure **2n** was consumed. The reaction mixture was concentrated under reduced pressure and pumped under the oil pump for 10 minutes to make sure **1f** was removed. Then the reaction tube containing **3af** was transferred to the glove box and **2i** (59 mg, 0.5 mmol) and THF (2 mL) were added to the tube. The tube was sealed with a screw cap equipped with a septum, removed from the glovebox. The reaction mixture was stirred at rt for 48 h under 6 W 390 nm LED lamps. The reaction mixture was concentrated under reduced pressure. The crude product was purified with column chromatography on silica gel (200~300 mesh) with PE as eluent to afford 114 mg of the title compound **4ab** as a colorless oil (71% yield).

^{1}H NMR (600 MHz, $CDCl_3$, 25 °C) δ 7.32–7.23 (m, 2H), 7.22–7.15 (m, 3H), 3.54 (t, J = 6.7 Hz, 2H, H_4), 2.64 (t, J = 7.8 Hz, 2H, H_3), 2.13–2.00 (m, 2H, H_2), 1.84–1.73 (m, 2H), 1.73–1.62 (m, 2H), 1.53–1.40 (m, 6H), 1.41–1.32 (m, 2H), 0.96 (t, J = 8.3 Hz, 4H, H_1, $H_{1'}$), 0.82–0.67 (m, 4H). ^{13}C NMR (151 MHz, $CDCl_3$, 25 °C) δ 142.9, 128.5, 128.4, 125.7, 45.3 (C_4), 35.8 (C_3), 35.3, 32.7, 32.7, 26.7, 23.7, 23.6, 18.6 (C_2), 15.2, 15.1, 12.3 (C_1, $C_{1'}$). ^{29}Si NMR (119 MHz, $CDCl_3$, 25 °C) δ 22.0. HRMS (APCI): (m/z) calcd for $C_{19}H_{32}ClSi^+$ (M+H$^+$), 323.1956; found, 323.1956.

6-(1-(4-Phenylbutyl)siletan-1-yl)hexan-1-ol (4ac)

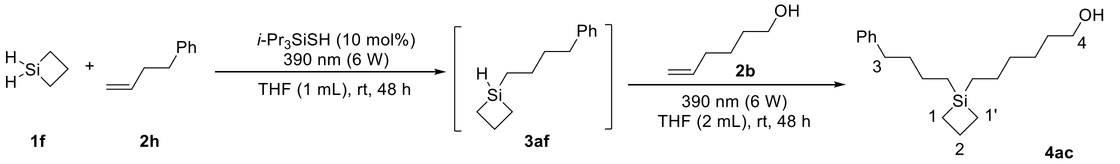

In an argon-filled glovebox, to a flame-dried screw-cap reaction tube equipped with a magnetic stir bar were added *i*-Pr_3SiSH (14.2 mg, 0.075 mmol), **2h** (99 mg, 0.75 mmol), THF (1 mL) and **1f** (3 mL, 0.38 M) sequentially. The tube was sealed with a screw cap equipped with a septum, removed from the glovebox. The reaction mixture was stirred at rt for 48 h under 6 W 390 nm LED lamps to ensure **2n** was consumed. The reaction mixture was concentrated under reduced pressure and pumped under the oil pump for 10 minutes to make sure **1f** was removed. Then the reaction tube containing **3af** was transferred to the glove box and **2b** (50 mg, 0.5 mmol) and THF (2 mL) were added to the tube. The tube was sealed with a screw cap equipped with a septum, removed from the glovebox. The reaction mixture was stirred at rt for 48 h under 6 W 390 nm LED lamps. The reaction mixture was concentrated under reduced pressure. The crude product was purified with column chromatography on silica gel (200~300 mesh) with PE/EA (5/1, v/v) as eluent to afford 112 mg of the title compound **4ac** as a colorless oil (74% yield).

^{1}H NMR (400 MHz, $CDCl_3$, 25 °C) δ 7.31–7.22 (m, 2H), 7.21–7.14 (m, 3H), 3.64 (t, J = 6.6 Hz, 2H, H_4), 2.64 (t, J = 7.8 Hz, 2H, H_3), 2.18–1.91 (m, 2H, H_2), 1.75–1.62 (m, 2H), 1.62–1.52 (m, 2H), 1.50–1.31 (m, 8H), 1.03–0.86 (m, 4H, H_1, $H_{1'}$), 0.85–0.63 (m, 4H). ^{13}C NMR (151 MHz, $CDCl_3$, 25 °C) δ 142.9,

128.5, 128.4, 125.7, 63.2 (C_4), 35.8 (C_3), 35.3, 33.2, 32.8, 25.6, 23.8, 23.6, 18.6 (C_2), 15.2, 15.1, 12.3 (C_1, $C_{1'}$). ^{29}Si NMR (119 MHz, $CDCl_3$, 25 °C) δ 22.0. HRMS (ESI): (m/z) calcd for $C_{19}H_{33}OSi^+$ (M+H$^+$), 305.2295; found, 305.2290.

1-(4-Phenylbutyl)-1-(3-(4,4,5,5-tetramethyl-1,3,2-dioxaborolan-2-yl)propyl)siletane (4ad)

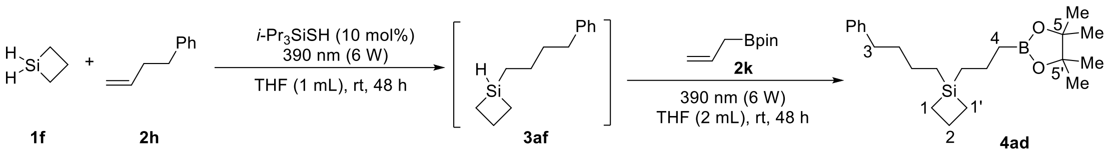

In an argon-filled glovebox, to a flame-dried screw-cap reaction tube equipped with a magnetic stir bar were added *i*-Pr_3SiSH (14.2 mg, 0.075 mmol), **2h** (99 mg, 0.75 mmol), THF (1 mL) and **1f** (3 mL, 0.38 M) sequentially. The tube was sealed with a screw cap equipped with a septum, removed from the glovebox. The reaction mixture was stirred at rt for 48 h under 6 W 390 nm LED lamps to ensure **2n** was consumed. The reaction mixture was concentrated under reduced pressure and pumped under the oil pump for 10 minutes to make sure **1f** was removed. Then the reaction tube containing **3af** was transferred to the glove box and **2k** (84 mg, 0.5 mmol) and THF (2 mL) were added to the tube. The tube was sealed with a screw cap equipped with a septum, removed from the glovebox. The reaction mixture was stirred at rt for 48 h under 6 W 390 nm LED lamps. The reaction mixture was concentrated under reduced pressure. The crude product was purified with column chromatography on silica gel (200~300 mesh) with PE/EA (50/1, v/v) as eluent to afford 150 mg of the title compound **4ad** as a colorless oil (81% yield).

^{1}H NMR (600 MHz, $CDCl_3$, 25 °C) δ 7.27–7.16 (m, 2H), 7.11 (d, *J* = 7.7 Hz, 3H), 2.55 (t, *J* = 7.8 Hz, 2H, H_3), 2.00–1.94 (m, 2H, H_2), 1.64–1.55 (m, 2H), 1.50–1.44 (m, 2H), 1.44–1.37 (m, 2H), 1.18 (s, 12H), 0.93–0.84 (m, 4H, H_1, $H_{1'}$), 0.80 (t, *J* = 7.7 Hz, 2H, H_4), 0.73–0.65 (m, 4H). ^{13}C NMR (151 MHz, $CDCl_3$, 25 °C) δ 142.9, 128.5, 128.4, 125.7, 83.0 (C_5), 35.9 (C_3), 35.3, 24.9, 23.6, 18.7, 18.6 (C_2), 18.4, 15.1, 12.3 (C_1, $C_{1'}$). ^{29}Si NMR (119 MHz, $CDCl_3$, 25 °C) δ 21.6. HRMS (ESI): (m/z) calcd for $C_{19}H_{33}OSi^+$ (M+H$^+$), 373.2729; found, 373.2723.

4-(1-(4-Phenylbutyl)siletan-1-yl)butanenitrile (4ae)

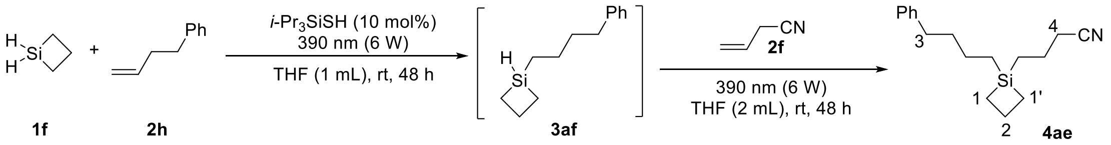

In an argon-filled glovebox, to a flame-dried screw-cap reaction tube equipped with a magnetic stir bar were added *i*-Pr_3SiSH (14.2 mg, 0.075 mmol), **2h** (99 mg, 0.75 mmol), THF (1 mL) and **1f** (3 mL, 0.38 M) sequentially. The tube was sealed with a screw cap equipped with a septum, removed from the glovebox. The reaction mixture was stirred at rt for 48 h under 6 W 390 nm LED lamps to ensure **2n** was consumed. The reaction mixture was concentrated under reduced pressure and pumped under the oil pump for 10 minutes to make sure **1f** was removed. Then the reaction tube containing **3af** was transferred to the glove box and **2f** (33.5 mg, 0.5 mmol) and THF (2 mL) were added to the tube. The tube was sealed with a screw cap equipped with a septum, removed from the glovebox. The reaction mixture was stirred at rt for 48 h under 6 W 390 nm LED lamps. The reaction mixture was concentrated under reduced pressure. The crude product was purified with column chromatography on silica gel (200~300 mesh) with PE/EA (100/1, v/v) as eluent to afford 103 mg of the title compound **4ae** as a colorless oil (76%

yield).

^{1}H NMR (400 MHz, $CDCl_3$, 25 ºC) δ 7.31–7.24 (m, 2H), 7.20–7.11 (m, 3H), 2.62 (t, J = 7.8 Hz, 2H, H_3), 2.38 (t, J = 7.0 Hz, 2H, H_4), 2.14–1.97 (m, 2H, H_2), 1.82–1.72 (m, 2H), 1.72–1.62 (m, 2H), 1.52–1.41 (m, 2H), 1.02–0.93 (m, 4H, H_1, $H_{1'}$), 0.91–0.84 (m, 2H), 0.82–0.74 (m, 2H). ^{13}C NMR (151 MHz, $CDCl_3$, 25 ºC) δ 142.7, 128.5, 128.4, 125.8, 119.8 (CN), 35.7 (C_3), 35.1, 23.4, 20.8, 20.7, 18.6 (C_2), 14.9, 14.8, 12.0 (C_1, $C_{1'}$). ^{29}Si NMR (119 MHz, $CDCl_3$, 25 ºC) δ 21.5. HRMS (ESI): (m/z) calcd for $C_{17}H_{26}NSi^+$ ($M+H^+$), 272.1829; found, 272.1827.

9-(2-(1-(4-Phenylbutyl)siletan-1-yl)ethyl)-9H-carbazole (4af)

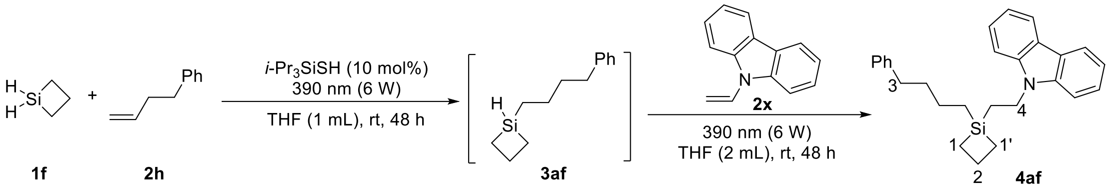

In an argon-filled glovebox, to a flame-dried screw-cap reaction tube equipped with a magnetic stir bar were added i-Pr_3SiSH (14.2 mg, 0.075 mmol), **2h** (99 mg, 0.75 mmol), THF (1 mL) and **1f** (3 mL, 0.38 M) sequentially. The tube was sealed with a screw cap equipped with a septum, removed from the glovebox. The reaction mixture was stirred at rt for 48 h under 6 W 390 nm LED lamps to ensure **2n** was consumed. The reaction mixture was concentrated under reduced pressure and pumped under the oil pump for 10 minutes to make sure **1f** was removed. Then the reaction tube containing **3af** was transferred to the glove box and **2x** (96.5 mg, 0.5 mmol) and THF (2 mL) were added to the tube. The tube was sealed with a screw cap equipped with a septum, removed from the glovebox. The reaction mixture was stirred at rt for 48 h under 6 W 390 nm LED lamps. The reaction mixture was concentrated under reduced pressure. The crude product was purified with column chromatography on silica gel (200~300 mesh) with PE as eluent to afford 135 mg of the title compound **4af** as a colorless oil (68% yield).

^{1}H NMR (400 MHz, $CDCl_3$, 25 ºC) δ 8.15–8.09 (m, 2H), 7.51–7.45 (m, 2H), 7.39 (d, J = 8.1 Hz, 2H), 7.32–7.19 (m, 5H), 7.17 (d, J = 6.3 Hz, 2H), 4.51–4.42 (m, 2H, H_4), 2.64–2.54 (m, 2H, H_3), 2.21–2.10 (m, 2H, H_2), 1.71–1.60 (m, 2H), 1.50–1.43 (m, 2H), 1.43–1.37 (m, 2H), 1.12–1.05 (m, 4H, H_1, $H_{1'}$), 0.82–0.72 (m, 2H). ^{13}C NMR (151 MHz, $CDCl_3$, 25 ºC) δ 142.7, 139.9, 128.5, 128.4, 125.8, 125.7, 123.2, 120.6, 118.9, 108.6, 38.8 (C_4), 35.7 (C_3), 35.1, 23.4, 18.8 (C_2), 15.4, 15.1, 12.2 (C_1, $C_{1'}$). ^{29}Si NMR (119 MHz, $CDCl_3$, 25 ºC) δ 20.2. HRMS (ESI): (m/z) calcd for $C_{27}H_{32}NSi^+$ ($M+H^+$), 398.2299; found, 398.2294.

1-(2-(1-(4-Phenylbutyl)siletan-1-yl)ethyl)pyrrolidin-2-one (4ag)

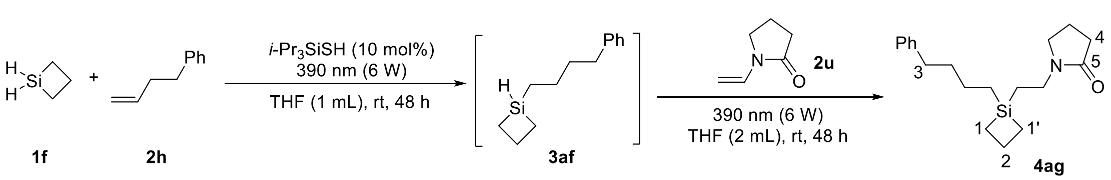

In an argon-filled glovebox, to a flame-dried screw-cap reaction tube equipped with a magnetic stir bar were added i-Pr_3SiSH (14.2 mg, 0.075 mmol), **2h** (99 mg, 0.75 mmol), THF (1 mL) and **1f** (3 mL, 0.38 M) sequentially. The tube was sealed with a screw cap equipped with a septum, removed from the glovebox. The reaction mixture was stirred at rt for 48 h under 6 W 390 nm LED lamps to ensure **2n** was consumed. The reaction mixture was concentrated under reduced pressure and pumped under the oil

pump for 10 minutes to make sure **1f** was removed. Then the reaction tube containing **3af** was transferred to the glove box and **2u** (55.5 mg, 0.5 mmol) and THF (2 mL) were added to the tube. The tube was sealed with a screw cap equipped with a septum, removed from the glovebox. The reaction mixture was stirred at rt for 48 h under 6 W 390 nm LED lamps. The reaction mixture was concentrated under reduced pressure. The crude product was purified with column chromatography on silica gel (200~300 mesh) with PE/EA (2/1, v/v) as eluent to afford 127 mg of the title compound **4ag** as a colorless oil (81% yield). ^{1}H NMR (400 MHz, $CDCl_3$, 25 °C) δ 7.30–7.24 (m, 2H), 7.20–7.12 (m, 3H), 3.47–3.33 (m, 4H), 2.67–2.58 (m, 2H, H_3), 2.37 (t, J = 8.1 Hz, 2H, H_4), 2.13–1.94 (m, 4H), 1.71–1.64 (m, 2H), 1.54–1.42 (m, 2H), 1.01 (td, J = 8.6, 5.4 Hz, 6H), 0.88–0.75 (m, 2H). ^{13}C NMR (151 MHz, $CDCl_3$, 25 °C) δ 174.5 (C_5), 142.8, 128.5, 128.4, 125.7, 46.4, 38.5, 35.8 (C_3), 35.1, 31.4, 23.4, 18.7 (C_2), 17.9, 15.0, 13.9, 12.1 (C_1, $C_{1'}$). ^{29}Si NMR (119 MHz, $CDCl_3$, 25 °C) δ 21.5. HRMS (ESI): (m/z) calcd for $C_{19}H_{30}NOSi^+$ (M+H$^+$), 316.2091; found, 316.2085.

(*1R,2S,5R*)-2-isopropyl-5-methylcyclohexyl 5-(1-(4-phenylbutyl)siletan-1-yl)pentanoate (4ah)

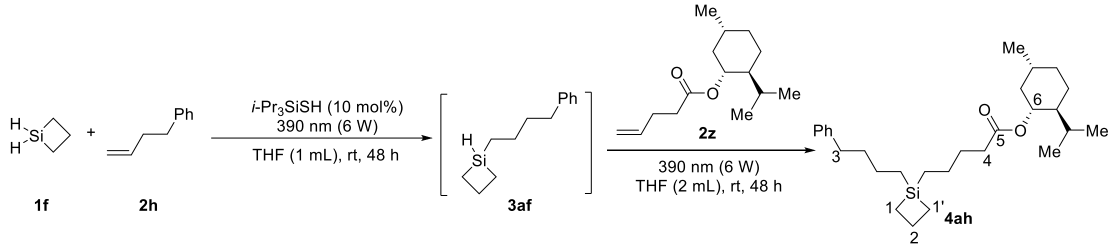

In an argon-filled glovebox, to a flame-dried screw-cap reaction tube equipped with a magnetic stir bar were added *i*-Pr_3SiSH (14.2 mg, 0.075 mmol), **2h** (99 mg, 0.75 mmol), THF (1 mL) and **1f** (3 mL, 0.38 M) sequentially. The tube was sealed with a screw cap equipped with a septum, removed from the glovebox. The reaction mixture was stirred at rt for 48 h under 6 W 390 nm LED lamps to ensure **2n** was consumed. The reaction mixture was concentrated under reduced pressure and pumped under the oil pump for 10 minutes to make sure **1f** was removed. Then the reaction tube containing **3af** was transferred to the glove box and **2z** (119 mg, 0.5 mmol) and THF (2 mL) were added to the tube. The tube was sealed with a screw cap equipped with a septum, removed from the glovebox. The reaction mixture was stirred at rt for 48 h under 6 W 390 nm LED lamps. The reaction mixture was concentrated under reduced pressure. The crude product was purified with column chromatography on silica gel (200~300 mesh) with PE/EA (50/1, v/v) as eluent to afford 166 mg of the title compound **4ah** as a colorless oil (75% yield).

^{1}H NMR (600 MHz, $CDCl_3$, 25 °C) δ 7.30–7.24 (m, 2H), 7.18 (d, J = 7.3 Hz, 3H), 4.69 (td, J = 10.9, 4.3 Hz, 1H, H_6), 2.63 (t, J = 7.8 Hz, 2H, H_3), 2.30 (t, J = 7.4 Hz, 2H, H_4), 2.07–1.99 (m, 2H, H_2), 2.01–1.96 (m, 1H), 1.91–1.83 (m, 1H), 1.70–1.66 (m, 6H), 1.46 (td, J = 17.1, 7.6 Hz, 5H), 1.39–1.34 (m, 1H), 1.12–0.99 (m, 2H), 0.99–0.92 (m, 5H), 0.91–0.82 (m, 7H), 0.78–0.72 (m, 6H). ^{13}C NMR (151 MHz, $CDCl_3$, 25 °C) δ 173.5 (C_5), 142.9, 128.5, 128.4, 125.7, 74.1 (C_6), 47.2, 41.1, 35.8 (C_3), 35.3, 34.7, 34.4, 31.5, 28.9, 26.4, 23.6, 23.6, 22.2, 20.9, 18.6 (C_2), 16.4, 15.1, 14.9, 12.2 (C_1, $C_{1'}$). ^{29}Si NMR (119 MHz, $CDCl_3$, 25 °C) δ 21.9. HRMS (ESI): (m/z) calcd for $C_{28}H_{47}O_2Si+$ (M+H$^+$), 443.3340; found, 443.3343.

3,6-dimethylhept-5-en-1-yl 5-(1-(6-acetoxyhexyl)siletan-1-yl)pentanoate (4ai)

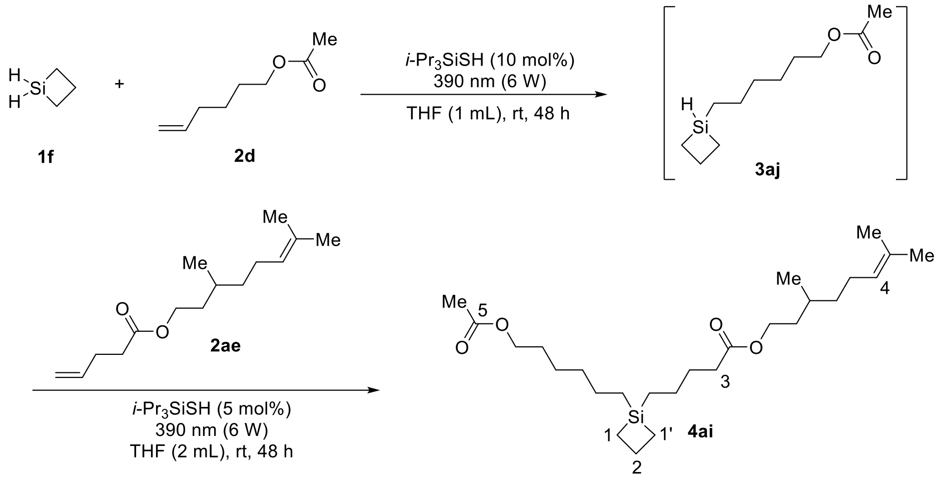

In an argon-filled glovebox, to a flame-dried screw-cap reaction tube equipped with a magnetic stir bar were added *i*-Pr_3SiSH (19.1 mg, 0.1 mmol), **2d** (142 mg, 1 mmol), THF (1 mL) and **1f** (4 mL, 0.38 M) sequentially. The tube was sealed with a screw cap equipped with a septum, removed from the glovebox. The reaction mixture was stirred at rt for 48 h under 6 W 390 nm LED lamps to ensure **2d** was consumed. The reaction mixture was concentrated under reduced pressure and pumped under the oil pump for 10 minutes to make sure **1f** was removed. Then the reaction tube containing **3aj** was transferred to the glove box and **2ae** (119 mg, 0.5 mmol), *i*-Pr_3SiSH (5 mg, 0.025 mmol, 5 mol%) and THF (2 mL) were added to the tube. The tube was sealed with a screw cap equipped with a septum, removed from the glovebox. The reaction mixture was stirred at rt for 48 h under 6 W 390 nm LED lamps. The reaction mixture was concentrated under reduced pressure. The crude product was purified with column chromatography on silica gel (200~300 mesh) with PE/EA (50/1, v/v) as eluent to afford 124 mg of the title compound **4ai** as a colorless oil (55% yield).

^{1}H NMR (600 MHz, $CDCl_3$, 25 ºC) δ 5.12–5.06 (m, 1H, H_4), 4.15–4.08 (m, 2H), 4.06 (t, *J* = 6.7 Hz, 2H), 2.32 (t, *J* = 7.5 Hz, 2H, H_3), 2.06–2.04 (m, 4H), 1.97 (m, 2H), 1.69 (d, *J* = 22.6 Hz, 5H), 1.60 (s, 5H), 1.59 (s, 3H), 1.54 (m, 1H), 1.48–1.41 (m, 5H), 1.39–1.34 (m, 4H), 1.22–1.14 (m, 1H), 0.97–0.94 (m, 4H, H_1, $H_{1'}$), 0.91 (d, *J* = 6.7 Hz, 3H), 0.78–0.67 (m, 4H). ^{13}C NMR (151 MHz, $CDCl_3$, 25 ºC) δ 174.1, 171.4 (C_5), 131.5, 124.7 (C_4), 64.8, 62.9, 37.1, 35.6, 34.3, 33.1, 29.6, 28.7, 28.7, 25.8, 25.7, 25.5, 23.8, 23.6, 21.2, 19.5, 18.6 (C_2), 17.8, 15.2, 14.9, 12.2 (C_1, $C_{1'}$). ^{29}Si NMR (119 MHz, $CDCl_3$, 25 ºC) δ 21.9. HRMS (ESI): (m/z) calcd for $C_{26}H_{49}O_4Si^+$ (M+H$^+$), 453.3395; found, 453.3391.

4-(1-(5-(((*3S,8S,9S,10R,13R,14S,17R*)-10,13-dimethyl-17-((*R*)-6-methylheptan-2-yl)-2,3,4,7,8,9,10,11,12,13,14,15,16,17-tetradecahydro-1H-cyclopenta[a]phenanthren-3-yl)oxy)-5-oxopentyl)siletan-1-yl)butyl benzoate (4aj)

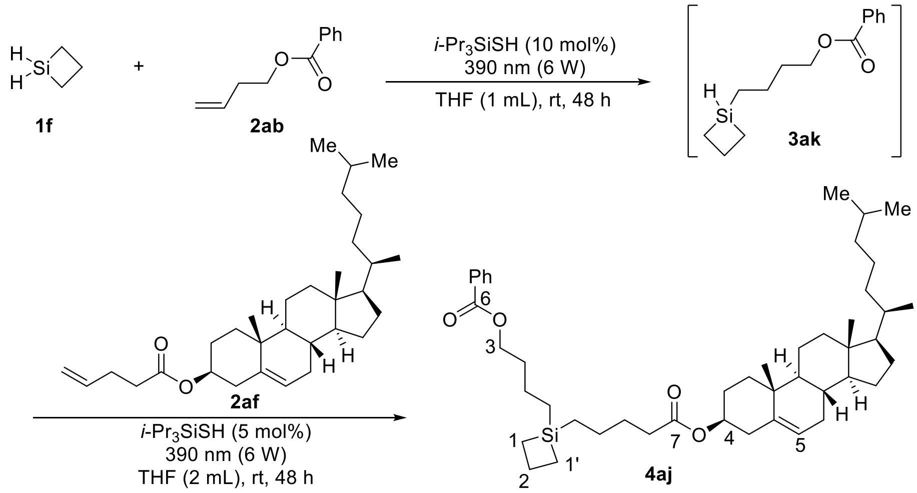

In an argon-filled glovebox, to a flame-dried screw-cap reaction tube equipped with a magnetic stir bar were added *i*-Pr_3SiSH (19.1 mg, 0.1 mmol), **2ab** (176 mg, 1 mmol), THF (1 mL) and **1f** (4 mL, 0.38 M) sequentially. The tube was sealed with a screw cap equipped with a septum, removed from the glovebox. The reaction mixture was stirred at rt for 48 h under 6 W 390 nm LED lamps to ensure **2ab** was consumed. The reaction mixture was concentrated under reduced pressure and pumped under the oil pump for 10 minutes to make sure **1f** was removed. Then the reaction tube containing **3ak** was transferred to the glove box and **2af** (234 mg, 0.5 mmol), *i*-Pr_3SiSH (5 mg, 0.025 mmol, 5 mol%) and THF (2 mL) were added to the tube. The tube was sealed with a screw cap equipped with a septum, removed from the glovebox. The reaction mixture was stirred at rt for 48 h under 6 W 390 nm LED lamps. The reaction mixture was concentrated under reduced pressure. The crude product was purified with column chromatography on silica gel (200~300 mesh) with PE/EA (20/1, v/v) as eluent to afford 154 mg of the title compound **4aj** as a colorless oil (43% yield).

^1H NMR (600 MHz, $CDCl_3$, 25 °C) δ 8.05–8.02 (m, 2H), 7.58–7.52 (m, 1H), 7.45–7.43 (m, 2H), 5.39–5.34 (m, 1H, H_5), 4.65–4.58 (m, 1H, H_4), 4.45 (t, J = 6.4 Hz, 2H, H_3), 2.35–2.25 (m, 4H), 2.10–1.94 (m, 5H), 1.87–1.81 (m, 4H), 1.71–1.63 (m, 3H), 1.51–1.41 (m, 6H), 1.35–1.23 (m, 7H), 1.18–1.05 (m, 8H), 1.03–0.85 (m, 19H), 0.83–0.73 (m, 4H), 0.69–0.65 (m, 3H). ^{13}C NMR (151 MHz, $CDCl_3$, 25 °C) δ 173.4 (C_7), 166.8 (C_6), 139.8, 132.9, 130.6, 129.7, 128.5, 122.7 (C_5), 73.9 (C_4), 64.8 (C_3), 56.8, 56.3, 50.2, 42.4, 39.8, 39.6, 38.3, 37.1, 36.7, 36.3, 35.9, 34.6, 32.3, 32.0, 32.0, 28.7, 28.4, 28.2, 27.9, 24.4, 23.9, 23.5, 22.9, 22.7, 21.2, 20.5, 19.5, 18.8, 18.6 (C_2), 14.9, 14.8, 12.2 (C_1, $C_{1'}$), 12.0. ^{29}Si NMR (119 MHz, $CDCl_3$, 25 °C) δ 21.7. HRMS (ESI): (m/z) calcd for $C_{46}H_{73}O_4Si^+$ ($M+H^+$), 717.5273; found, 717.5278.

4-(1-(5-(((*3S,8R,9S,10R,13S,14S*)-10,13-dimethyl-17-oxo-2,3,4,7,8,9,10,11,12,13,14,15,16,17-tetradecahydro-1H-cyclopenta[a]phenanthren-3-yl)oxy)-5-oxopentyl)siletan-1-yl)butyl benzoate (4ak)

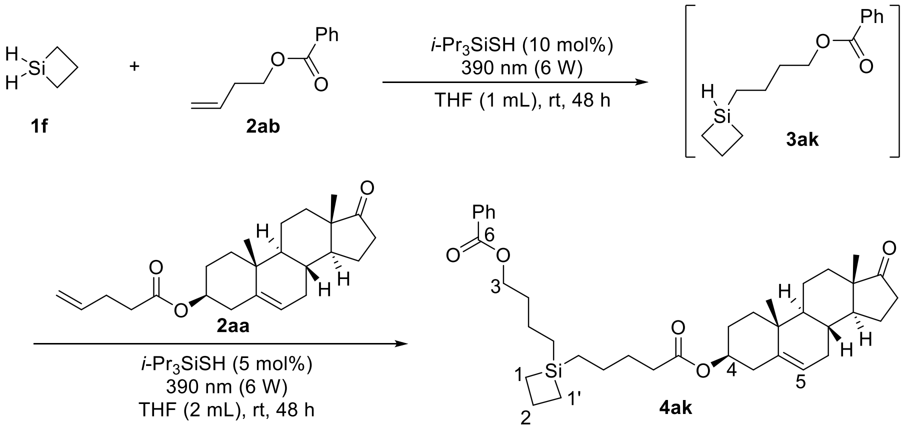

In an argon-filled glovebox, to a flame-dried screw-cap reaction tube equipped with a magnetic stir bar were added *i*-Pr_3SiSH (19.1 mg, 0.1 mmol), **2ab** (176 mg, 1 mmol), THF (1 mL) and **1f** (4 mL, 0.38 M) sequentially. The tube was sealed with a screw cap equipped with a septum, removed from the glovebox. The reaction mixture was stirred at rt for 48 h under 6 W 390 nm LED lamps to ensure **2ab** was consumed. The reaction mixture was concentrated under reduced pressure and pumped under the oil pump for 10 minutes to make sure **1f** was removed. Then the reaction tube containing **3ak** was transferred to the glove box and **2aa** (185 mg, 0.5 mmol), *i*-Pr_3SiSH (5 mg, 0.025 mmol, 5 mol%) and THF (2 mL) were added to the tube. The tube was sealed with a screw cap equipped with a septum, removed from the glovebox. The reaction mixture was stirred at rt for 48 h under 6 W 390 nm LED lamps. The reaction mixture was concentrated under reduced pressure. The crude product was purified with column chromatography on silica gel (200~300 mesh) with PE/EA (5/1, v/v) as eluent to afford 161 mg of the title compound **4ak** as a colorless oil (52% yield).

^{1}H NMR (600 MHz, $CDCl_3$, 25 ºC) δ 8.05 (d, *J* = 7.8 Hz, 2H), 7.56 (m, 1H), 7.44 (m, 2H), 5.44–5.37 (m, 1H, H_5), 4.68–4.55 (m, 1H, H_4), 4.34 (t, *J* = 6.4 Hz, 2H, H_3), 2.46 (m, 1H), 2.38–2.26 (m, 4H), 2.14–2.02 (m, 4H), 1.98–1.92 (m, 1H), 1.91–1.81 (m, 5H), 1.70–1.62 (m, 5H), 1.60–1.41 (m, 6H), 1.35–1.23 (m, 3H), 1.20–1.08 (m, 2H), 0.98 (t, *J* = 8.4 Hz, 7H), 0.88 (s, 3H), 0.82–0.74 (m, 4H). ^{13}C NMR (151 MHz, $CDCl_3$, 25 ºC) δ 221.3, 173.3, 166.8 (C_6), 140.1, 132.9, 130.6, 129.7, 128.5, 121.9 (C_5), 73.6 (C_4), 64.8 (C_3), 51.8, 50.3, 47.6, 38.3, 37.1, 36.8, 35.9, 34.5, 32.3, 31.6, 31.5, 30.9, 28.7, 27.8, 23.5, 22.0, 20.4, 19.5, 18.6 (C_2), 14.9, 14.8, 13.7, 12.2 (C_1, $C_{1'}$). ^{29}Si NMR (119 MHz, $CDCl_3$, 25 ºC) δ 21.9. HRMS (ESI): (m/z) calcd for $C_{38}H_{55}O_5Si^+$ (M+H$^+$), 619.3813; found, 619.3818.

6. Gram scale reaction

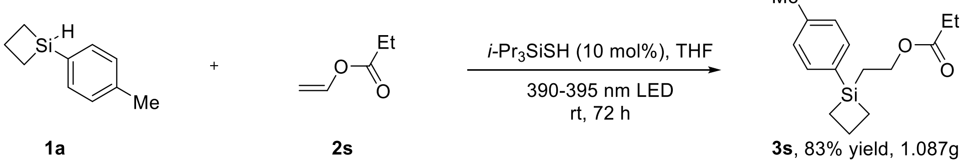

In an argon-filled glovebox, to a flame-dried screw-cap reaction tube equipped with a magnetic stir bar were added *i*-Pr_3SiSH (95 mg, 0.5 mmol), **1a** (1.2 g, 7.5 mmol, 1.5 equiv.), **2s** (500 mg, 5 mmol, 1 equiv.) and THF (10 mL) sequentially. The tube was sealed with a screw cap equipped with a septum, removed from the glovebox. The reaction mixture was stirred at rt for 72 h under 40 W 390 nm LED lamps. The

reaction mixture was concentrated under reduced pressure. The crude product was purified with column chromatography on silica gel (200~300 mesh) with PE/EA (20/1, v/v) as eluent to afford 1.087 g of the title compound **3s** as a colorless oil (83% yield).

7. Synthetic applications

(*E*)-2-(propyl(styryl)(p-tolyl)silyl)ethyl propionate (5)

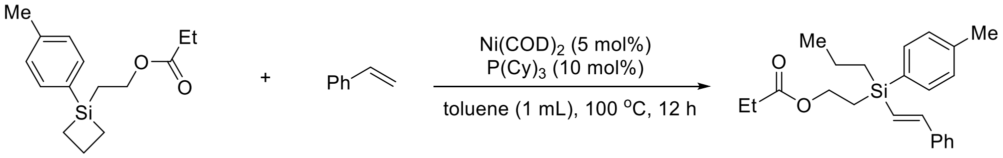

3s **5**

In a glovebox, to an oven-dried 10 mL Schlenk tube equipped with a magnetic stir bar was added $Ni(COD)_2$ (2.75 mg, 0.01 mmol, 5 mol%), $(Cy)_3P$ (5.6 mg, 0.02 mmol, 10 mol%), dry toluene (1 mL), **3s** (52 mg, 0.2 mmol), **styrene** (31.2 mg, 0.3 mmol). The tube was then sealed, and the resulting mixture was stirred at 100 °C in a heating block for 12 h, after which the mixture was cooled to room temperature. The reaction mixture was filtered through a thin silica gel plug with CH_2Cl_2 (30 mL) as the eluent. The organic phase was concentrated under reduced pressure. The crude product was purified with column chromatography on silica gel (200~300 mesh) with PE/EA (50/1, v/v) as eluent to afford **5** as a colorless oil (68 mg, 86% yield).

^{1}H NMR (400 MHz, $CDCl_3$, 25 °C) δ 7.46–7.43 (m, 4H), 7.36–7.25 (m, 3H), 7.22–7.16 (m, 2H), 6.98 (d, *J* = 19.2 Hz, 1H), 6.54 (d, *J* = 19.3 Hz, 1H), 4.26–4.14 (m, 2H), 2.35 (s, 3H), 2.25 (q, *J* = 7.6 Hz, 2H), 1.50–1.38 (m, 4H), 1.09 (t, *J* = 7.6 Hz, 3H), 1.01–0.94 (m, 5H). ^{13}C NMR (151 MHz, $CDCl_3$, 25 °C) δ 174.7, 146.9, 139.5, 138.1, 134.6, 131.8, 128.9, 128.7, 128.5, 126.7, 123.9, 62.3, 27.8, 21.6, 18.5, 17.4, 15.9, 14.3, 9.2. ^{29}Si NMR (119 MHz, $CDCl_3$, 25 °C) δ -11.3. HRMS (ESI): (m/z) calcd for $C_{23}H_{34}NO_2Si^+$ ($M+NH_4^+$), 384.2353; found, 384.2351.

(*E*)-2-(2-(2-ethoxy-2-oxoethylidene)-1-(p-tolyl)silinan-1-yl)ethyl propionate (6)

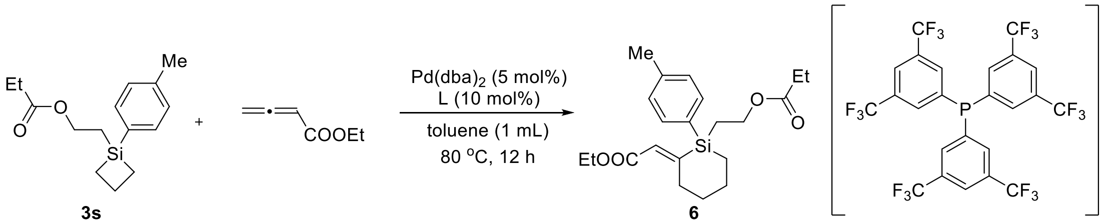

3s **6** L

In a glovebox, to an oven-dried 10 mL Schlenk tube equipped with a magnetic stir bar was added $Pd(dba)_2$ (5.6 mg, 0.01 mmol, 5 mol%), L (14 mg, 0.02 mmol, 10 mol%), dry toluene (1 mL), **3s** (52 mg, 0.2 mmol), **ethyl buta-2,3-dienoate** (44.8 mg, 0.4 mmol). The tube was then sealed, and the resulting mixture was stirred at 80 °C in a heating block for 12 h, after which the mixture was cooled to room temperature. The reaction mixture was filtered through a thin silica gel plug with CH_2Cl_2 (30 mL) as the eluent. The organic phase was concentrated under reduced pressure. The crude product was purified with column chromatography on silica gel (200~300 mesh) with PE/EA (20/1, v/v) as eluent to afford **6** as a colorless oil (63 mg, 85% yield).

^{1}H NMR (400 MHz, $CDCl_3$, 25 °C) δ 7.41 (d, *J* = 7.4 Hz, 2H), 7.20 (d, *J* = 7.5 Hz, 2H), 5.99 (s, 1H), 4.20–4.10 (m, 4H), 3.25–3.13 (m, 1H), 2.89–2.76 (m, 1H), 2.36 (s, 3H), 2.29–2.20 (m, 2H), 1.93–1.73 (m, 2H), 1.64–1.57 (m, 2H), 1.43–1.34 (m, 2H), 1.30–1.22 (m, 4H), 1.09 (t, *J* = 7.5 Hz, 3H), 1.03–0.93 (m, 1H).^{13}C NMR (151 MHz, $CDCl_3$, 25 °C) δ 174.6, 165.5, 162.4, 140.0, 134.6, 129.5, 129.2, 126.4,

61.6, 59.9, 32.5, 29.9, 27.7, 23.9, 21.6, 14.4, 13.3, 12.2, 9.2. ^{29}Si NMR (119 MHz, $CDCl_3$, 25 °C) δ -12.5. HRMS (ESI): (m/z) calcd for $C_{21}H_{31}O_4Si^+$ (M+H$^+$), 375.1986; found, 375.1984.

2-(Propyl(*p*-tolyl)((triisopropylsilyl)ethynyl)silyl)ethyl propionate (7)

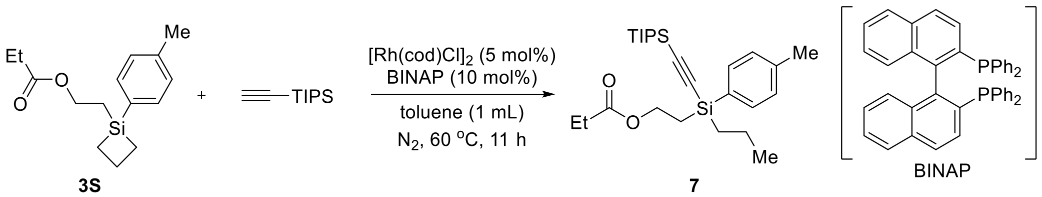

In a glovebox, to an oven-dried 10 mL Schlenk tube equipped with a magnetic stir bar was added $[Rh(cod)Cl]_2$ (2.5 mg, 0.005 mmol, 5 mol%), BINAP (6.23 mg, 0.01 mmol, 10 mol%), dry toluene (0.6 mL), **3s** (26.2 mg, 0.1 mmol), **ethynyltriisopropylsilane** (21.9 mg, 0.12 mmol). The tube was then sealed, and the resulting mixture was stirred at 60 °C in a heating block for 11 h, after which the mixture was cooled to room temperature. The reaction mixture was filtered through a thin silica gel plug with CH_2Cl_2 (30 mL) as the eluent. The organic phase was concentrated under reduced pressure. The crude product was purified with column chromatography on silica gel (200~300 mesh) with PE/EA (20/1, v/v) as eluent to afford **7** as a colorless oil (35.5 mg, 85% yield).

^{1}H NMR (400 MHz, $CDCl_3$, 25 °C) δ 7.54 (d, *J* = 8.0 Hz, 2H), 7.20 (d, *J* = 7.4 Hz, 2H), 4.33–4.14 (m, 2H), 2.36 (s, 3H), 2.28–2.20 (m, 2H), 1.55–1.40 (m, 2H), 1.35–1.29 (m, 2H), 1.14–1.08 (m, 24H), 0.98 (t, *J* = 7.3 Hz, 3H), 0.91–0.85 (m, 2H). ^{13}C NMR (151 MHz, $CDCl_3$, 25 °C) δ 174.6, 139.6, 134.4, 130.7, 128.9, 114.7, 110.6, 62.2, 27.7, 21.6, 18.7, 18.1, 17.5, 17.0, 15.5, 11.2, 9.2. ^{29}Si NMR (119 MHz, $CDCl_3$, 25 °C) δ -2.2, -21.9. HRMS (ESI): (m/z) calcd for $C_{26}H_{48}NO_2Si_2^+$ (M+NH$_4^+$), 462.3218; found, 462.3213.

2-(5,6-diphenyl-1-(p-tolyl)-1,2,3,4-tetrahydrosilin-1-yl)ethyl propionate (8)

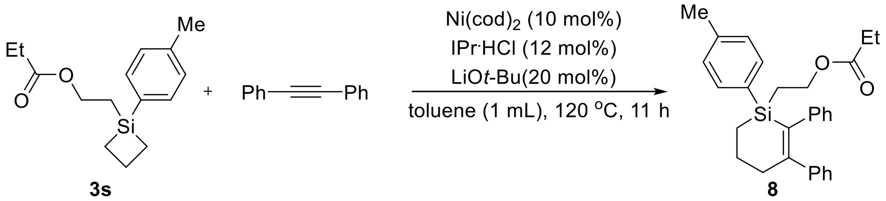

In a glovebox, to an oven-dried 10 mL Schlenk tube equipped with a magnetic stir bar was added $Ni(cod)_2$ (2.75 mg, 0.01 mmol, 10 mol%), IPr·HCl (5.1 mg, 0.012 mmol, 12 mol%), dry toluene (0.5 mL), **3s** (52.4 mg, 0.2 mmol), **1,2-diphenylethyne** (17.8 mg, 0.1 mmol). The tube was then sealed, and the resulting mixture was stirred at 120 °C in a heating block for 11 h, after which the mixture was cooled to room temperature. The reaction mixture was filtered through a thin silica gel plug with CH_2Cl_2 (30 mL) as the eluent. The organic phase was concentrated under reduced pressure. The crude product was purified with column chromatography on silica gel (200~300 mesh) with PE/EA (20/1, v/v) as eluent to afford **8** as a colorless oil (32 mg, 73% yield).

^{1}H NMR (600 MHz, $CDCl_3$, 25 °C) δ 7.33 (d, *J* = 7.4 Hz, 2H), 7.09 (d, *J* = 7.5 Hz, 2H), 7.01–6.96 (m, 2H), 6.95–6.91 (m, 1H), 6.90–6.81 (m, 5H), 6.58 (d, *J* = 7.4 Hz, 2H), 4.10–3.98 (m, 2H), 2.67–2.49 (m, 2H), 2.27 (s, 3H), 2.20–2.14 (m, 2H), 2.06–1.96 (m, 2H), 1.28–1.18 (m, 4H), 1.02 (t, *J* = 7.5 Hz, 3H). ^{13}C NMR (151 MHz, $CDCl_3$, 25 °C) δ 174.7, 156.6, 144.6, 142.6, 139.4, 134.7, 134.3, 132.6, 129.4, 128.8, 128.4, 127.6, 127.6, 126.2, 124.9, 62.1, 37.2, 27.8, 21.9, 21.6, 13.7, 10.3, 9.2. ^{29}Si NMR (119 MHz, $CDCl_3$, 25 °C) δ -18.5. HRMS (ESI): (m/z) calcd for $C_{29}H_{33}O_2Si^+$ (M+H$^+$), 441.2244; found, 441.2252.

1-methoxy-1-(4-phenylbutyl)siletane (9)

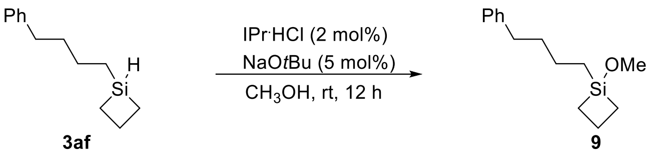

In a glovebox, to an oven-dried 10 mL Schlenk tube equipped with a magnetic stir bar was added IPr·HCl (1.7 mg, 0.004 mmol, 12 mol%), NaO*t*-Bu (1 mg, 0.01 mmol, 5 mol%), dry MeOH (2 mL), **3af** (41 mg, 0.2 mmol). The tube was then sealed, and the resulting mixture was stirred at rt for 12 h. The reaction mixture was filtered through a thin silica gel plug with CH_2Cl_2 (30 mL) as the eluent. The organic phase was concentrated under reduced pressure. The crude product was purified with column chromatography on silica gel (200~300 mesh) with PE/EA (50/1, v/v) as eluent to afford **9** as a colorless oil (34 mg, 73% yield).

^{1}H NMR (400 MHz, $CDCl_3$, 25 °C) δ 7.30–7.22 (m, 2H), 7.19–7.10 (m, 3H), 3.50 (s, 3H), 2.63–2.54 (m, 2H), 1.71–1.59 (m, 2H), 1.47–1.34 (m, 4H), 0.96 (t, *J* = 7.3 Hz, 2H), 0.71–0.52 (m, 4H). ^{13}C NMR (151 MHz, $CDCl_3$, 25 °C) δ 142.8, 128.5, 128.4, 125.7, 50.4, 35.7, 35.3, 22.5, 18.2, 16.4, 14.5, 11.9. ^{29}Si NMR (119 MHz, $CDCl_3$, 25 ℃) δ -2.9. HRMS (APCI): (m/z) calcd for $C_{14}H_{23}OSi^+$ (M+H$^+$), 235.1513; found, 235.1515.

4-(1-(3-(benzyloxy)-3-oxopropyl)siletan-1-yl)butyl benzoate (10a)

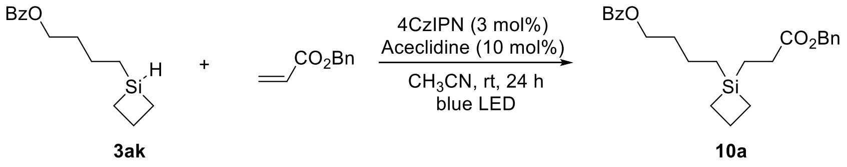

In an argon-filled glovebox, to a flame-dried screw-cap reaction tube equipped with a magnetic stir bar were added aceclidine (1.7 mg, 0.01 mmol, 10 mol%), 4CzIPN (2.35 mg, 0.003 mmol, 3 mol%), **3ak** (24.8 mg, 0.1 mmol), **alkene** (16.3 mg, 0.1 mmol) and CH_3CN (1.0 mL) sequentially. The tube was sealed with a screw cap equipped with a septum, removed from the glovebox. The reaction mixture was stirred at rt for 24 h under 6 W 460 nm LED lamps. The reaction mixture was concentrated under reduced pressure. The crude product was purified with column chromatography on silica gel (200~300 mesh) with PE/EA (20/1, v/v) as eluent to afford 33 mg of the title compound **10a** as a colorless oil (81% yield).

^{1}H NMR (400 MHz, $CDCl_3$, 25 °C) δ 8.07–8.01 (m, 2H), 7.58–7.52 (m, 1H), 7.46–7.40 (m, 2H), 7.38–7.30 (m, 5H), 5.12 (s, 2H), 4.34 (t, *J* = 6.6 Hz, 2H), 2.52–2.44 (m, 2H), 2.13–1.98 (m, 2H), 1.86–1.78 (m, 2H), 1.59 (d, *J* = 3.6 Hz, 2H), 1.13–1.07 (m, 2H), 1.03–0.96 (m, 4H), 0.86–0.79 (m, 2H). ^{13}C NMR (151 MHz, $CDCl_3$, 25 °C) δ 174.7, 166.8, 136.1, 132.9, 130.6, 129.6, 128.7, 128.4, 128.4, 128.4, 66.5, 64.7, 32.2, 28.8, 20.3, 18.5, 14.7, 12.0, 10.2. ^{29}Si NMR (119 MHz, $CDCl_3$, 25 ℃) δ 22.4. HRMS (APCI): (m/z) calcd for $C_{24}H_{31}O_4Si^+$ (M+H$^+$), 411.1986; found, 411.1984.

4-(1-(2-cyanoethyl)siletan-1-yl)butyl benzoate (10b)

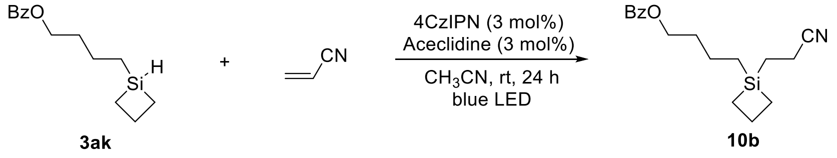

In an argon-filled glovebox, to a flame-dried screw-cap reaction tube equipped with a magnetic stir bar were added aceclidine (1.7 mg, 0.01 mmol, 10 mol%), 4CzIPN (2.35 mg, 0.003 mmol, 3 mol%), **3ak**

(24.8 mg, 0.1 mmol), **alkene** (5.3 mg, 0.1 mmol) and CH_3CN (1.0 mL) sequentially. The tube was sealed with a screw cap equipped with a septum, removed from the glovebox. The reaction mixture was stirred at rt for 24 h under 6 W 460 nm LED lamps. The reaction mixture was concentrated under reduced pressure. The crude product was purified with column chromatography on silica gel (200~300 mesh) with PE/EA (20/1, v/v) as eluent to afford 17 mg of the title compound **10b** as a colorless oil (55% yield). ^{1}H NMR (600 MHz, $CDCl_3$, 25 °C) δ 8.04 (d, *J* = 7.7 Hz, 2H), 7.59–7.53 (m, 1H), 7.48–7.40 (m, 2H), 4.35 (t, *J* = 6.6 Hz, 2H), 2.46 (t, *J* = 8.0 Hz, 2H), 2.18–2.03 (m, 2H), 1.88–1.79 (m, 2H), 1.61 (d, *J* = 8.4 Hz, 2H), 1.16 (d, *J* = 7.9 Hz, 2H), 1.07 (t, *J* = 8.5 Hz, 4H), 0.88 (t, *J* = 8.4 Hz, 2H). ^{13}C NMR (151 MHz, $CDCl_3$, 25 °C) δ 166.8, 133.0, 130.5, 129.6, 128.5, 121.0, 64.5, 32.2, 20.2, 18.6, 14.6, 12.1, 11.8, 11.2. ^{29}Si NMR (119 MHz, $CDCl_3$, 25 °C) δ 22.3. HRMS (ESI): (m/z) calcd for $C_{17}H_{24}NO_2Si^+$ (M+H$^+$), 302.1571; found, 302.1564.

5-(1-(3-methoxy-3-oxopropyl)siletan-1-yl)pentyl benzoate (10c)

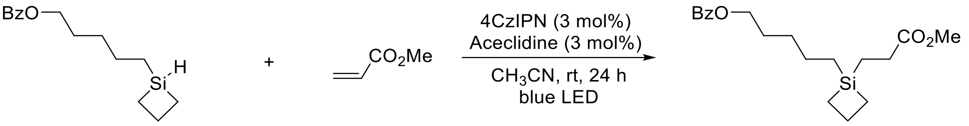

3ar **10c**

In an argon-filled glovebox, to a flame-dried screw-cap reaction tube equipped with a magnetic stir bar were added aceclidine (1.7 mg, 0.01 mmol, 10 mol%), 4CzIPN (2.35 mg, 0.003 mmol, 3 mol%), **3ar** (26.2 mg, 0.1 mmol), **alkene** (8.6 mg, 0.1 mmol) and CH_3CN (1.0 mL) sequentially. The tube was sealed with a screw cap equipped with a septum, removed from the glovebox. The reaction mixture was stirred at rt for 24 h under 6 W 460 nm LED lamps. The reaction mixture was concentrated under reduced pressure. The crude product was purified with column chromatography on silica gel (200~300 mesh) with PE/EA (20/1, v/v) as eluent to afford 25 mg of the title compound **10c** as a colorless oil (71% yield). ^{1}H NMR (600 MHz, $CDCl_3$, 25 °C) δ 8.06–8.02 (m, 2H), 7.58–7.52 (m, 1H), 7.45–7.42 (m, 2H), 4.32 (t, *J* = 6.6 Hz, 2H), 3.67 (s, 3H), 2.44–2.38 (m, 2H), 2.12–1.99 (m, 2H), 1.81–1.77 (m, 2H), 1.56–1.46 (m, 4H), 1.09–1.03 (m, 2H), 1.03–0.93 (m, 4H), 0.80–0.77 (m, 2H). ^{13}C NMR (151 MHz, $CDCl_3$, 25 °C) δ 175.4, 166.8, 132.9, 130.6, 129.6, 128.4, 65.2, 51.8, 29.8, 28.6, 28.6, 23.5, 18.5, 15.1, 12.1, 10.3. ^{29}Si NMR (119 MHz, $CDCl_3$, 25 °C) δ 22.3. HRMS (ESI): (m/z) calcd for $C_{19}H_{29}O_4Si^+$ (M+H$^+$), 349.1830; found, 349.1823.

8. Mechanism studies

8.1 Control experiment: non-strained cyclic and acyclic silanes under the standard conditions.

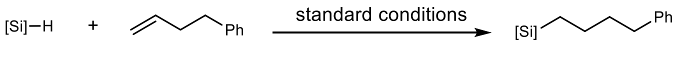

2h **11-13**[a]

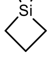	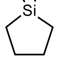	Et_2SiH_2	Et_3SiH
**1f**	**1g**		
100% c. of **2h**<br>90% y. of **3af**	70% c. of **2h**<br>66% y. of **11**	45% c. of **2h**<br>41% y. of **12**	6% c. of **2h**<br>5% y. of **13**

a): "c.": conversion; "y.": yield.

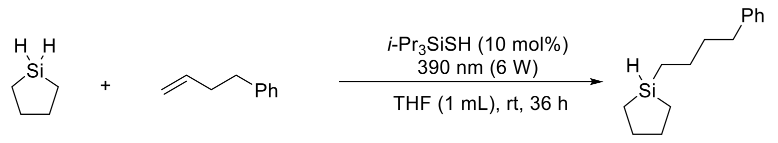

1g **2h** **11**

In an argon-filled glovebox, to a flame-dried screw-cap reaction tube equipped with a magnetic stir bar were added *i*-Pr_3SiSH (9.5 mg, 0.05 mmol), **2h** (66 mg, 0.5 mmol, 1 equiv.), THF (1 mL) and **1g** (2 mL, 0.38 M, 1.5 equiv.) sequentially. The tube was sealed with a screw cap equipped with a septum, removed from the glovebox. The reaction mixture was stirred at rt for 36 h under 6 W 390 nm LED lamps. The reaction mixture was concentrated under reduced pressure. The crude product was purified with column chromatography on silica gel (100~200 mesh) with PE as eluent to afford 72 mg of compound **11** as a colorless oil (66% yield). ^{1}H NMR (600 MHz, $CDCl_3$, 25 ℃) δ 7.29–7.25 (m, 2H), 7.20–7.14 (m, 3H), 3.97–3.91 (m, 1H), 2.60 (t, *J* = 9 Hz, 2H), 1.69–1.62 (m, 2H), 1.58–1.54 (m, 4H), 1.46–1.39 (m, 2H), 0.79–0.72 (m, 2H), 0.72–0.67 (m, 2H), 0.57–0.50 (m, 2H). ^{13}C NMR (151 MHz, $CDCl_3$, 25 ℃) δ 142.9, 128.5, 128.4, 125.7, 35.8, 35.0, 27.5, 24.7, 12.4, 9.1. ^{29}Si NMR (119 MHz, $CDCl_3$, 25 ℃) δ 1.8. HRMS (APCI): (m/z) calcd for $C_{14}H_{23}Si^+$ ($M+H^+$), 219.1564; found, 219.1560.

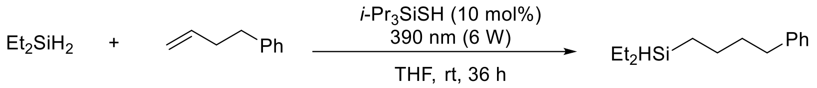

2h **12**

In an argon-filled glovebox, to a flame-dried screw-cap reaction tube equipped with a magnetic stir bar were added *i*-Pr_3SiSH (9.5 mg, 0.05 mmol), **2h** (66 mg, 0.5 mmol, 1 equiv.), THF (3 mL) and **Et_2SiH_2** (66 mg, 0.75 mmol, 1.5 equiv.) sequentially. The tube was sealed with a screw cap equipped with a septum, removed from the glovebox. The reaction mixture was stirred at rt for 36 h under 6 W 390 nm LED lamps. The reaction mixture was concentrated under reduced pressure. The crude product was purified with column chromatography on silica gel (100~200 mesh) with PE as eluent to afford 45 mg of compound **12** as a colorless oil (41% yield). ^{1}H NMR (400 MHz, $CDCl_3$, 25 ℃) δ 7.30–7.25 (m, 2H), 7.21–7.12 (m, 3H), 3.69–3.58 (m, 1H), 2.61 (t, *J* = 8 Hz, 2H), 1.70–1.61 (m, 2H), 1.46–1.36 (m, 2H), 0.97 (t, *J* = 7.9 Hz, 6H), 0.67–0.54 (m, 6H). ^{13}C NMR (151 MHz, $CDCl_3$, 25 ℃) δ 142.9, 128.5, 128.4, 125.7, 35.8, 35.3, 24.5, 10.6, 8.4, 2.9. ^{29}Si NMR (119 MHz, $CDCl_3$, 25 ℃) δ -2.0. HRMS (APCI): (m/z) calcd for $C_{14}H_{25}Si^+$ ($M+H^+$), 221.1720; found, 221.1726.

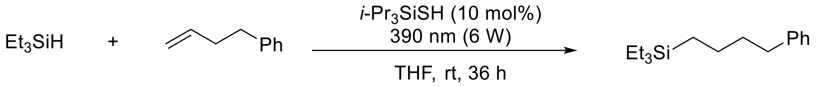

2h **13**

In an argon-filled glovebox, to a flame-dried screw-cap reaction tube equipped with a magnetic stir bar were added *i*-Pr_3SiSH (9.5 mg, 0.05 mmol), **2h** (66 mg, 0.5 mmol, 1 equiv.), THF (3 mL) and **Et_3SiH** (87 mg, 0.75 mmol, 1.5 equiv.) sequentially. The tube was sealed with a screw cap equipped with a septum, removed from the glovebox. The reaction mixture was stirred at rt for 36 h under 6 W 390 nm LED lamps. The reaction mixture was concentrated under reduced pressure. The crude product was purified with column chromatography on silica gel (100~200 mesh) with PE as eluent to afford 6 mg of compound **13** as a colorless oil (5% yield). The spectroscopic data for this product matched the literature data.[4] ^{1}H NMR (400 MHz, $CDCl_3$, 25 ℃) δ 7.29–7.24 (m, 2H), 7.18–7.14 (m, 3H), 2.60 (t, *J* = 8.0 Hz, 2H), 1.67–1.60 (m, 2H), 1.39–1.33 (m, 2H), 0.92 (t, *J* = 8.0 Hz, 9H), 0.57–0.53 (m, 2H), 0.49 (q, *J* = 8.0

Hz, 6H).

8.2 Control experiment: without *i*-Pr_3SiSH under standard conditions.

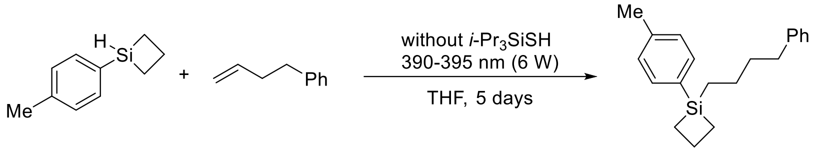

In an argon-filled glovebox, to a flame-dried screw-cap reaction tube equipped with a magnetic stir bar were added **1a** (97.2 mg, 0.6 mmol, 1.2 equiv.), **2h** (66 mg, 0.5 mmol, 1 equiv.) and THF (2.0 mL) sequentially. The tube was sealed with a screw cap equipped with a septum, removed from the glovebox. The reaction mixture was stirred at rt for 5 days under 6 W 390 nm LED lamps. The yield of **3h** (11% yield) was determined by GC. This result proves that **1a** could produce silicon radical under 390 nm irradiation.

8.3 Radical clock experiments.

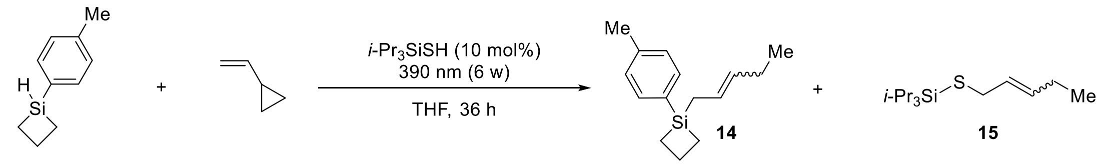

In an argon-filled glovebox, to a flame-dried screw-cap reaction tube equipped with a magnetic stir bar were added *i*-Pr_3SiSH (9.5 mg, 0.05 mmol), **1a** (97.2 mg, 0.6 mmol, 1.2 equiv.), **2ah** (34 mg, 0.5 mmol, 1 equiv.) and THF (2.0 mL) sequentially. The tube was sealed with a screw cap equipped with a septum, removed from the glovebox. The reaction mixture was stirred at rt for 36 h under 6 W 390 nm LED lamps. The reaction mixture was concentrated under reduced pressure. The crude product was purified with column chromatography on silica gel (200~300 mesh) with PE as eluent to afford 47 mg of the compound **14** as a colorless oil (41% yield) and 10 mg of the compound **15** as a colorless oil (81% yield).
Spectra date of **14**: ^{1}H NMR (600 MHz, $CDCl_3$, 25 ºC) δ 7.55–7.48 (m, 2H), 7.23–7.18 (m, 2H), 5.53–5.32 (m, 2H), 2.36 (s, 3H), 2.17–2.09 (m, 2H), 2.06–1.97 (m, 2H), 1.97–1.87 (m, 2H), 1.23 (t, J = 7.4 Hz, 4H), 0.98–0.92 (m, 3H). ^{13}C NMR (151 MHz, $CDCl_3$, 25 ºC) δ 139.6, 139.5, 134.1, 134.1, 133.9, 133.8, 132.2, 130.9, 128.9, 128.8, 123.5, 123.3, 26.0, 21.7, 20.8, 20.6, 18.5, 18.3, 17.1, 14.5, 14.4, 13.2, 12.9. ^{29}Si NMR (119 MHz, $CDCl_3$, 25 ºC) δ 11.1, 10.9. HRMS (APCI): (m/z) calcd for $C_{15}H_{23}Si^+$ (M+H$^+$), 231.1564; found, 231.1562.
Spectra date of **15**: ^{1}H NMR (600 MHz, $CDCl_3$, 25 ºC) δ 5.66–5.47 (m, 2H), 3.16 (d, J = 5.9 Hz, 2H), 2.08–1.99 (m, 2H), 1.28–1.23 (m, 3H), 1.12 (d, J = 7.4 Hz, 18H), 0.98 (t, J = 7.4 Hz, 3H). ^{13}C NMR (151 MHz, $CDCl_3$, 25 ºC) δ 134.1, 127.6, 28.2, 25.4, 18.7, 13.6, 12.9. HRMS (APCI): (m/z) calcd for $C_{14}H_{31}SSi^+$ (M+H$^+$), 259.1910; found, 259.1908.

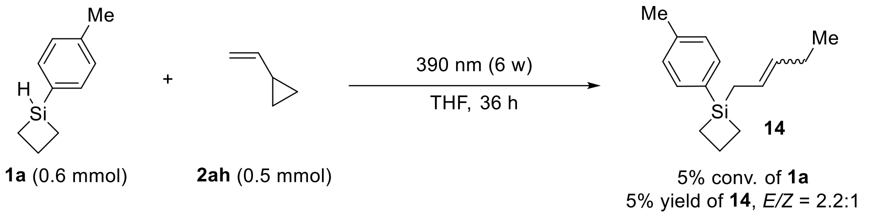

In an argon-filled glovebox, to a flame-dried screw-cap reaction tube equipped with a magnetic stir bar were added **1a** (97.2 mg, 0.6 mmol, 1.2 equiv.), **2ah** (34 mg, 0.5 mmol, 1 equiv.) and THF (2.0 mL) sequentially. The tube was sealed with a screw cap equipped with a septum, removed from the glovebox. The reaction mixture was stirred at rt for 36 h under 6 W 390 nm LED lamps. The reaction mixture was concentrated under reduced pressure. The crude product was purified with column chromatography on silica gel (200~300 mesh) with PE as eluent to afford 6 mg of the compound **14** as a colorless oil (5% yield).

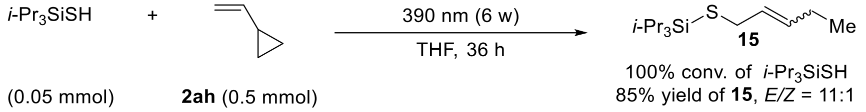

In an argon-filled glovebox, to a flame-dried screw-cap reaction tube equipped with a magnetic stir bar were added *i*-Pr_3SiSH (9.5 mg, 0.05 mmol), **2ah** (34 mg, 0.5 mmol, 1 equiv.) and THF (2.0 mL) sequentially. The tube was sealed with a screw cap equipped with a septum, removed from the glovebox. The reaction mixture was stirred at rt for 36 h under 6 W 390 nm LED lamps. The reaction mixture was concentrated under reduced pressure. The crude product was purified with column chromatography on silica gel (200~300 mesh) with PE as eluent to afford 11 mg of the compound **15** as a colorless oil (85% yield).

8.4 EPR experiments.

EPR spectra was recorded at 298 K on EPR spectrometer operated at 9.820 GHz. Typical spectrometer parameters are shown as follows, scan range: 100 G; center field set: 3503 G; time constant: 163.84 ms; S21 scan time: 30.72 s; modulation amplitude: 1.0 G; modulation frequency: 100 kHz; receiver gain: 1.00×10^4; microwave power: 20.90 mW.

A. EPR studies of 1a: In an argon-filled glovebox, to a flame-dried screw-cap reaction tube equipped with a magnetic stir bar were added **1a** (97.2 mg, 0.6 mmol) and THF (2.0 mL) sequentially. The tube was sealed with a screw cap equipped with a septum, removed from the glovebox. The reaction mixture was stirred at room temperature under 6 W 390 nm LED lamps. After 6 h, DMPO (30 μL) was added and stirred for 4 min. Then, the solution sample was taken out into a small tube and analyzed by EPR. A mixture signal of **radical 1** (g = 2.0076, A_N = 14.30 G, A_H =20.60 G), **carbon radical** (g = 2.0076, A_N = 13.90 G, A_H =18.0 G) and **hydrogenated DMPO** (g = 2.0076, A_N = 14.50 G, A_{H1} = 18.80 G, A_{H2} = 18.80 G) were identified. The simulation data for **carbon** and **hydrogen radicals** are consistent with reference (*Free Radical Bio. Med.* **1987**, *3*, 259–303. Page 264, line 40; Page 261).[5] Based on radical clock experiments and EPR studies of *i*-Pr_3SiSH, We speculated that **radical 1** is a **silyl radical. Carbon radical** may be formed from the HAT process of **silyl radical** or **hydrogen radical** with THF.

B. EPR studies of *i*-Pr_3SiSH: In an argon-filled glovebox, to a flame-dried screw-cap reaction tube equipped with a magnetic stir bar were added *i*-Pr_3SiSH (9.5 mg, 0.05 mmol) and THF (2.0 mL) sequentially. The tube was sealed with a screw cap equipped with a septum, removed from the glovebox. The reaction mixture was stirred at room temperature under 6 W 390 nm LED lamps. After 6 h, DMPO

(30 μL) was added and stirred for 4 min. Then, the solution sample was taken out into a small tube and analyzed by EPR. A mixture signal of thiyl radicals (g = 2.0083, A_N = 13.20 G, A_H =11.60 G), carbon radicals (g = 2.0078, A_N = 13.90 G, A_H =18.0 G) was identified. The simulation data for **thiyl radical** and **carbon radical** are consistent with reference (*Free Radical Bio. Med.* **1987**, *3*, 259–303. Page 272, line 21-23; Page 264, line 40). **Carbon radical** may be formed from the HAT process of **thiyl radical** with THF.

C. EPR studies of the reaction system: In an argon-filled glovebox, to a flame-dried screw-cap reaction tube equipped with a magnetic stir bar were added *i*-Pr_3SiSH (9.5 mg, 0.05 mmol), **1a** (97.2 mg, 0.6 mmol, 1.2 equiv.), **2h** (66 mg, 0.5 mmol, 1 equiv.) and THF (2.0 mL) sequentially. The tube was sealed with a screw cap equipped with a septum, removed from the glovebox. The reaction mixture was stirred at room temperature under 6 W 390 nm LED lamps. After 6 h, DMPO (30 μL) was added and stirred for 4 min. Then, the solution sample was taken out into a small tube and analyzed by EPR. A mixture signal of **radical 1** (g = 2.0079, A_N = 14.30 G, A_H =20.60 G), **thiyl radicals** (g = 2.0083, A_N = 13.20 G, A_H =11.60 G), and **carbon radicals** (g = 2.0079, A_N = 13.90 G, A_H =18.0 G) was identified. The experimental result provided strong evidence for the radical process of the reaction.

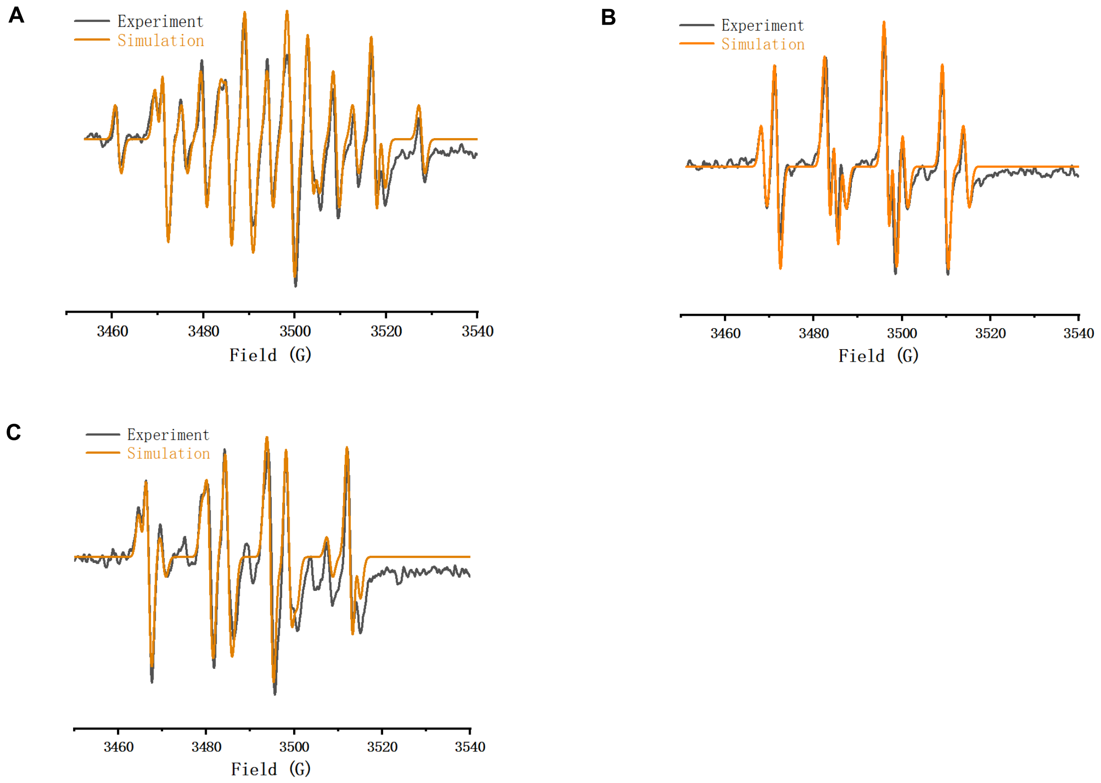

Supplementary Fig. 4. **A** EPR studies of **1a**. **B** EPR studies of *i*-Pr_3SiSH. **C** EPR studies of the reaction system**.**

The simulations process for EPR data: taking the simulations process of the EPR date of **1a** as an example:

1. Use the "eprfileconverter.exe" software to normalize the raw data to obtain the ordinate and save it as a text file.

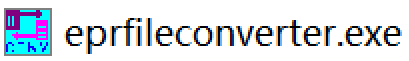

2. Simulate normalized data using "isotropicradicals.exe":

isotropicradicals.exe

Software operation interface as follow:

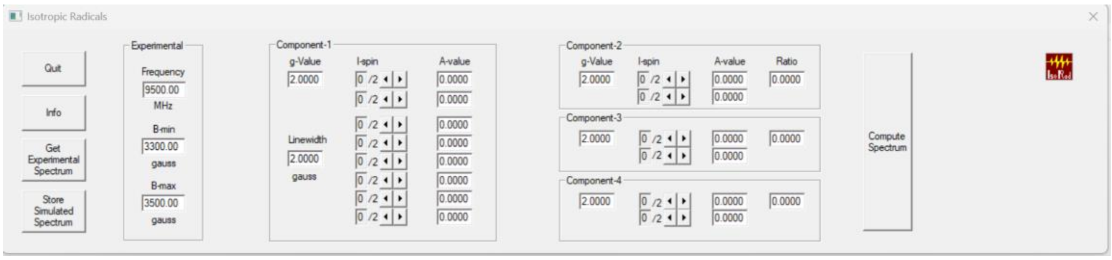

a. Click the "Get Experimental Spectrum" button to import the normalized data.

b. Enter the microwave frequency of 9819.544 used in the experimental spectrum test into Frequency, enter the minimum value of 3454.021484 on the abscissa of the experimental spectrum into B-min, and enter the maximum value of 3553.997070 on the abscissa of the experimental spectrum into B-max.

c. Enter 2.0067 for g-Value first, and 0.8 for Linewidth. Then click "Compute Spectrum" to get the EPR spectrum of a single electron as follows (green line is the experimental spectrum, red line is the simulation spectrum):

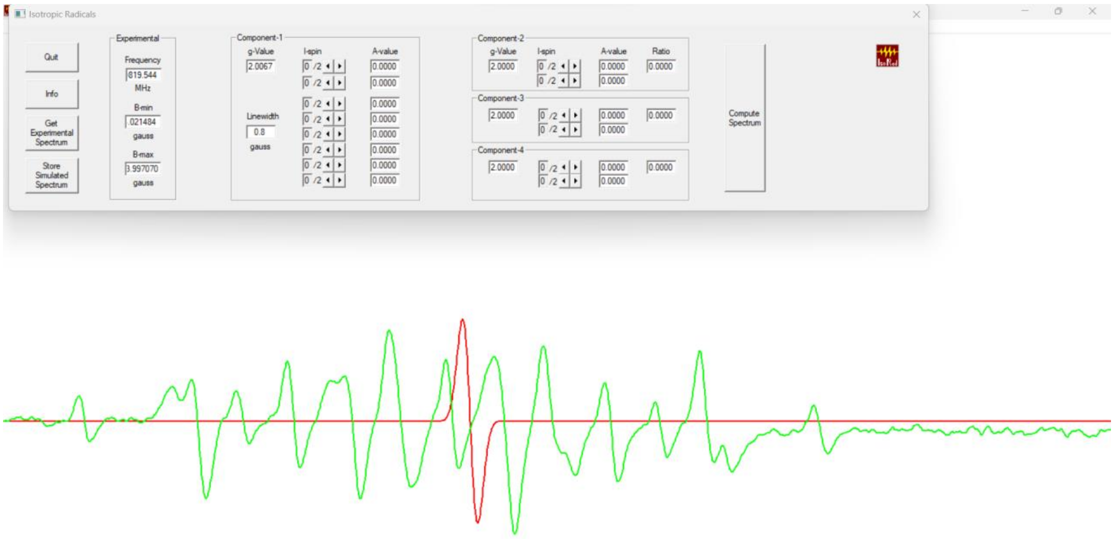

d. Analysis of the experimental spectrum shows that silyl radicals, hydrogen radicals and carbon radicals may exist in the system. We first simulate the hydrogen radical. The DMPO-H radical structure formed by the addition of hydrogen radicals to DMPO is shown below. One nitrogen atom and two hydrogen atoms will affect the splitting of oxygen radicals (oxygen, carbon, silicon, etc. will not cause the free radical signal to break).

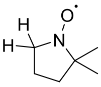

e. In component-1, fill the I-spin column with 2/2, 1/2, 1/2. Fill in 14.0, 22.0, 22.0 in the A-value column. Click on "Compute Spectrum" to obtain the following preliminary simulation spectrum of hydrogen radicals:

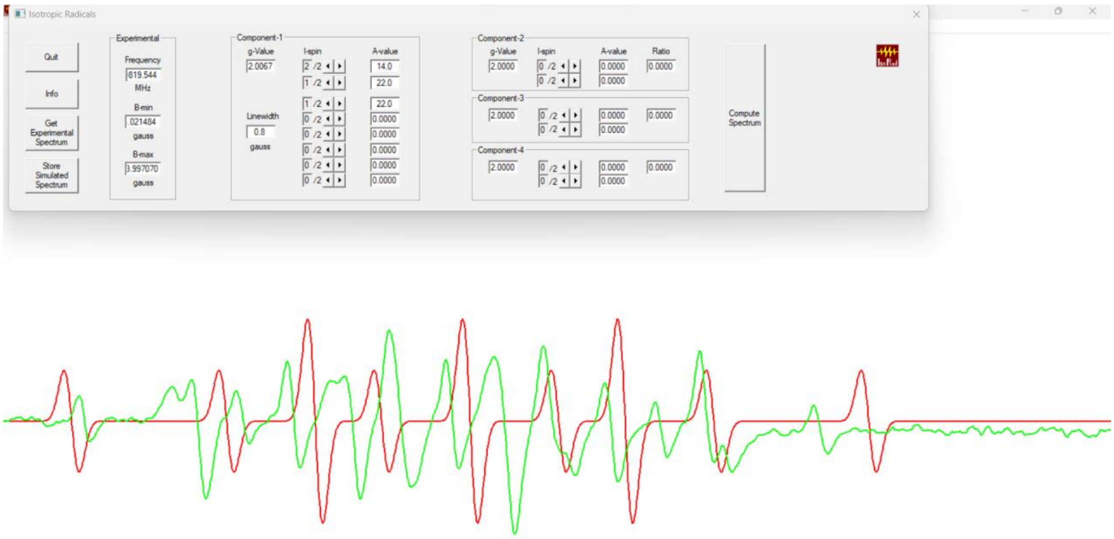

f. Under different testing conditions such as different solvents, the measured g and A values of free radicals will differ. Then adjust the A-value based on the experimental spectrum. The final adjustment was g1=2.0076; AN=14.5 G; AH1 = AH2 =18.8 G. The following spectrum is obtained:

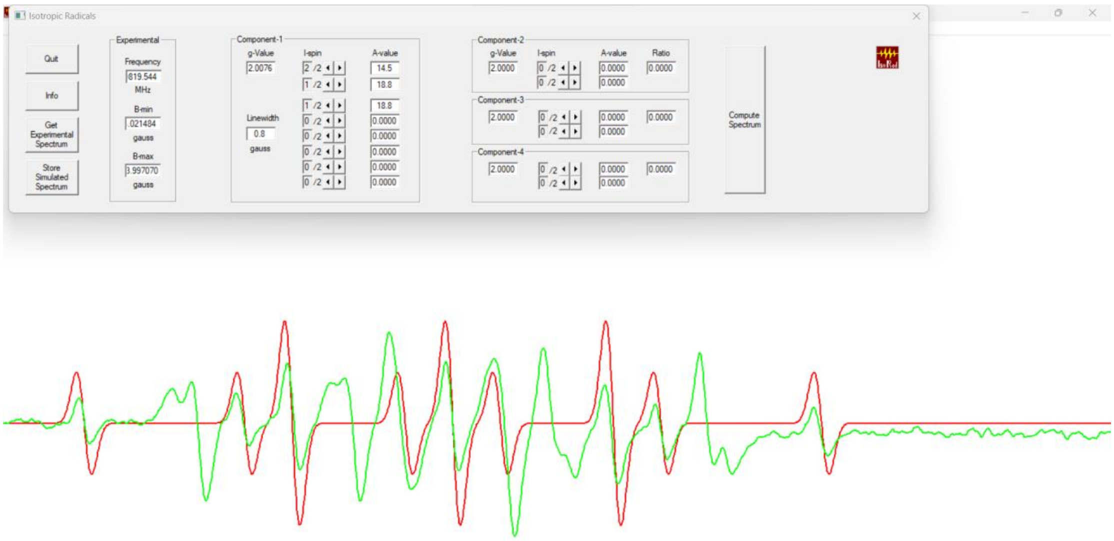

g. Then we add carbon radicals, and the parameters are filled in component-2. The simulation analysis process and operations are as follows. The final g-value is filled in 2.0076; the I-spin column is filled in 2/2, 1/2; and the A-value column is filled in 13.9, 18.0. Ratio refers to the ratio of component-2 to component-1 for this radical species. Fill in 1.5 based on the actual spectrum. The following simulation spectra were obtained:

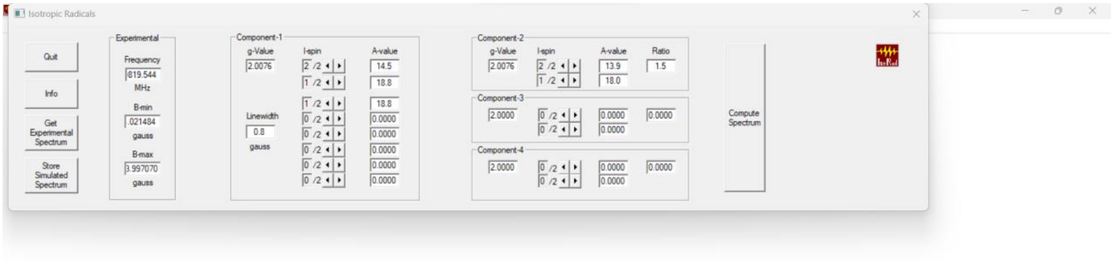

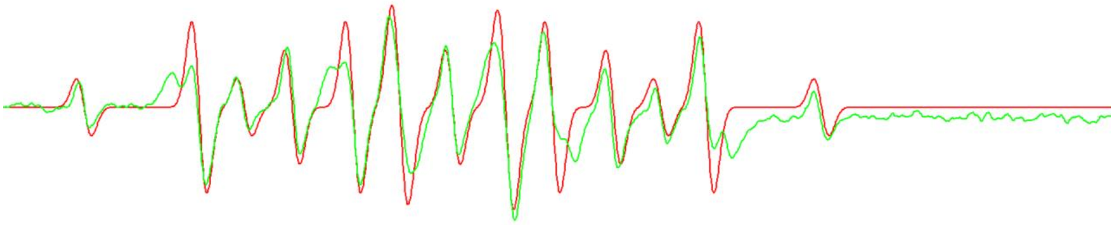

h. Finally, we add silyl radicals and fill their parameters in component-3. The simulation analysis process and operations are as follows. The final g-value is filled with 2.0076; the I-spin column is filled with 2/2, 1/2; and the A-value column is filled with 14.3, 20.6. Ratio refers to the ratio of component-3 to component-1 for this radical species. Fill in 0.7 based on the actual spectrum. The following simulation spectrum was obtained:

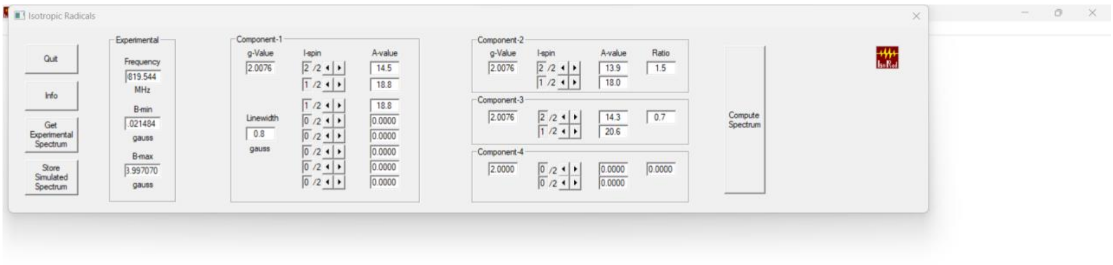

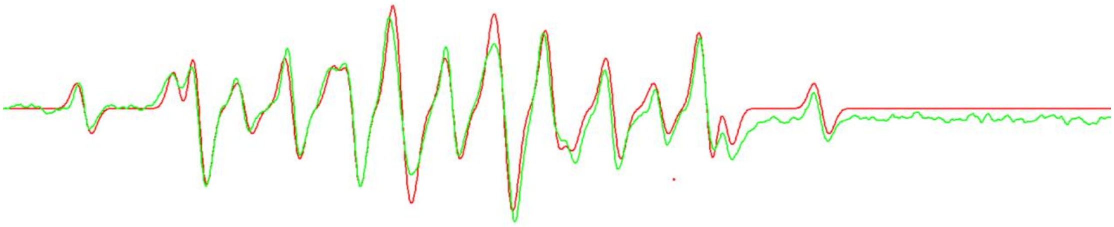

i. Click "Store Simulated Spectrum" to save the ordinate of the simulation data.

j. Take the abscissa of the raw data and the normalized ordinate of the raw data to draw the raw data spectrum, and take the abscissa of the raw data and the simulation data to draw the simulation spectrum:

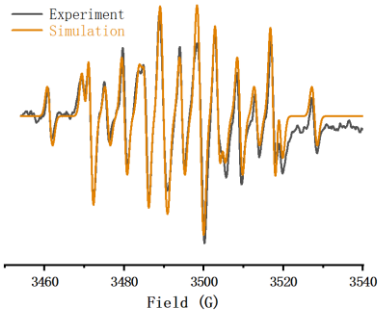

8.5 NMR control experiments.

A. comparing the ^{1}H NMR of **1a** in THF-d8, and Toluene-d8 (with tetramethylsilane as an internal standard). We found that when the Lewis basicity of the solvent increased, the chemical shift of the Si-H decreased, indicating that THF may coordinate with hydrosilacyclobutane.

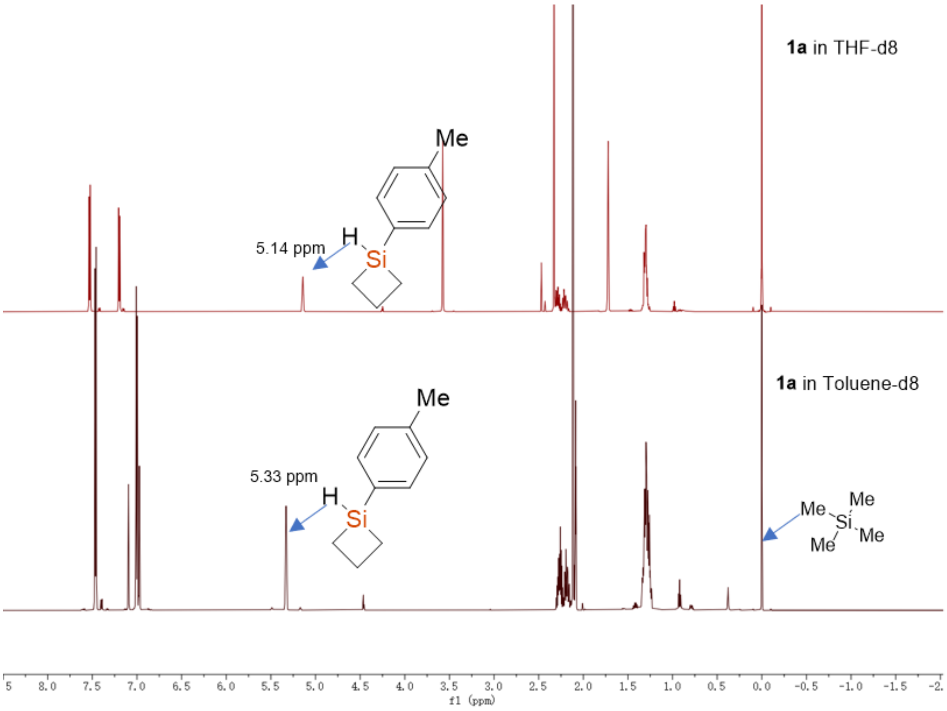

Supplementary Fig. 5. ^{1}H NMR of **1a** in THF-d8, and Toluene-d8

B. comparing the ^{29}Si NMR of **1f** in THF-d8 and Toluene-d8 (with tetramethylsilane as internal standard), We found that there was a slight spectroscopic differences, which indicates that hydrosilacyclobutane may have a weak coordination with THF.

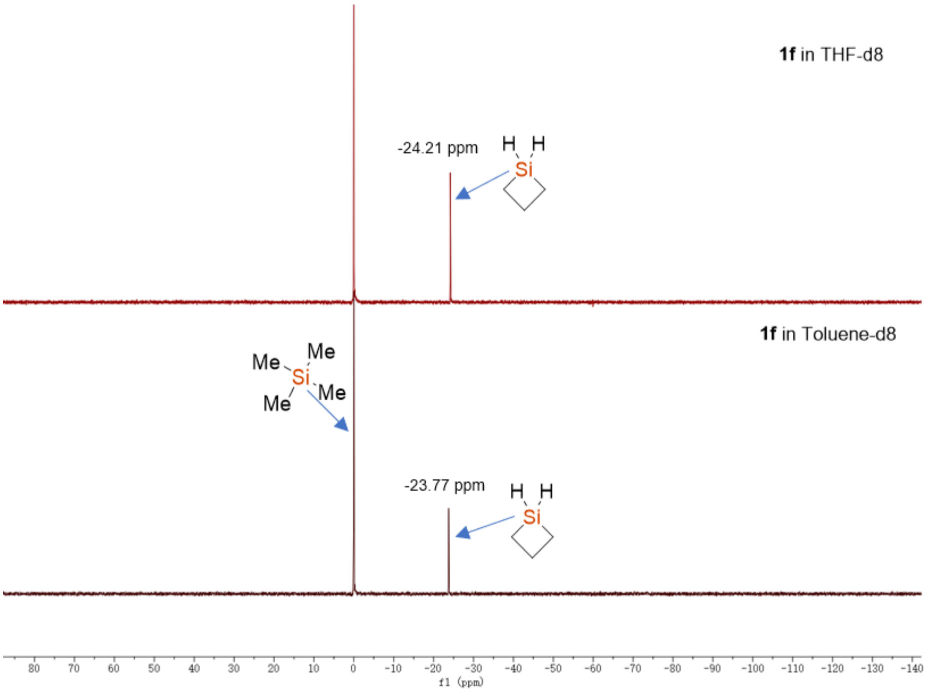

Supplementary Fig. 6. ^{29}Si NMR of **1f** in THF-d8, and Toluene-d8

9. Computational Methods

All density functional theory (DFT) calculations were performed using Gaussian 16 software package.[6] The M06-2X exchange-correlation functional,[7] combined with the 6-311G(d,p) basis set,[8] is utilized to optimize structures in the tetrahydrofuran solvent, accounting for solvation effects through the application of the SMD continuum solvation model.[9] Vibrational frequency calculations were conducted for all stationary points to confirm that each optimized structure represents a local minimum, ensuring that all optimized compounds exhibit no imaginary frequencies.

10. NMR spectra for new compounds

^{1}H NMR of **3a** (600 MHz, $CDCl_3$, 25 ºC)

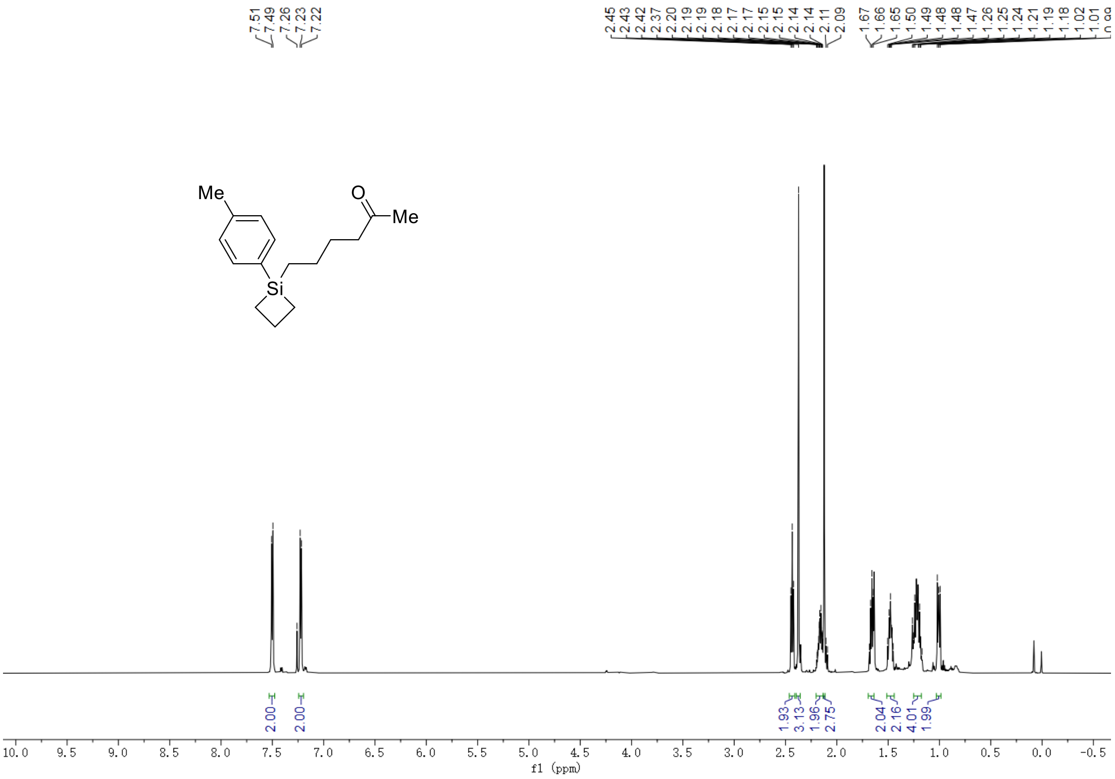

^{13}C NMR of **3a** (151 MHz, $CDCl_3$, 25 ºC)

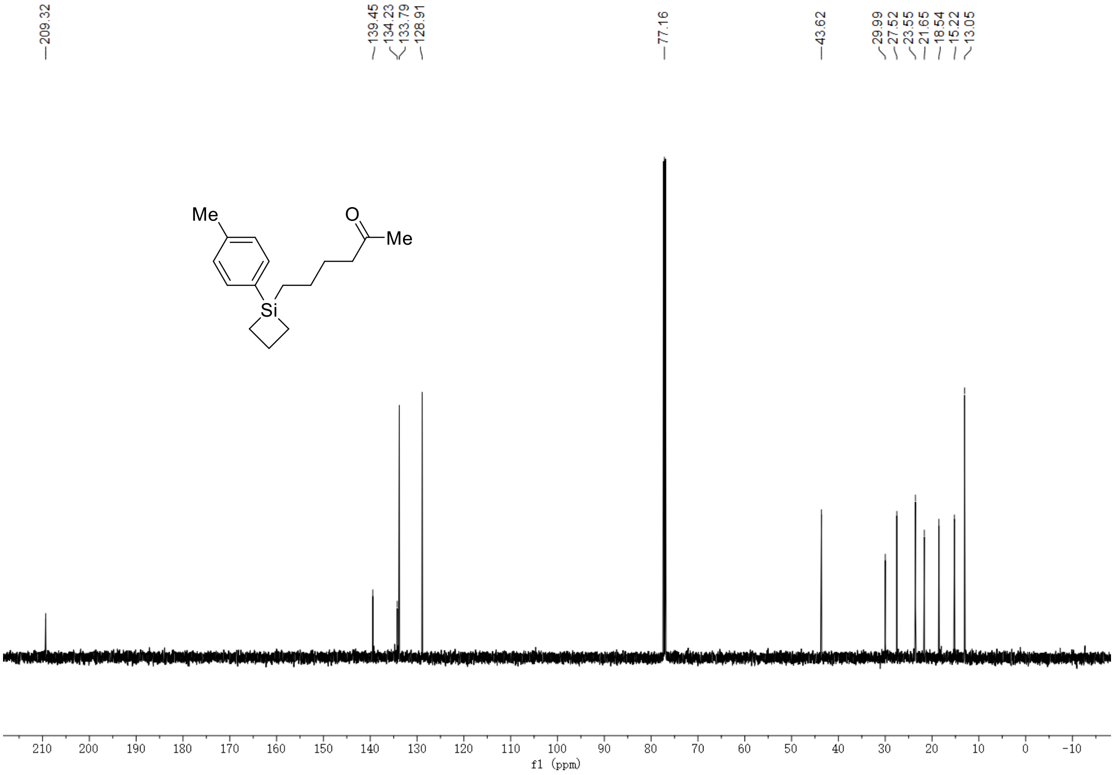

^{29}Si NMR of **3a** (119 MHz, $CDCl_3$, 25 ºC)

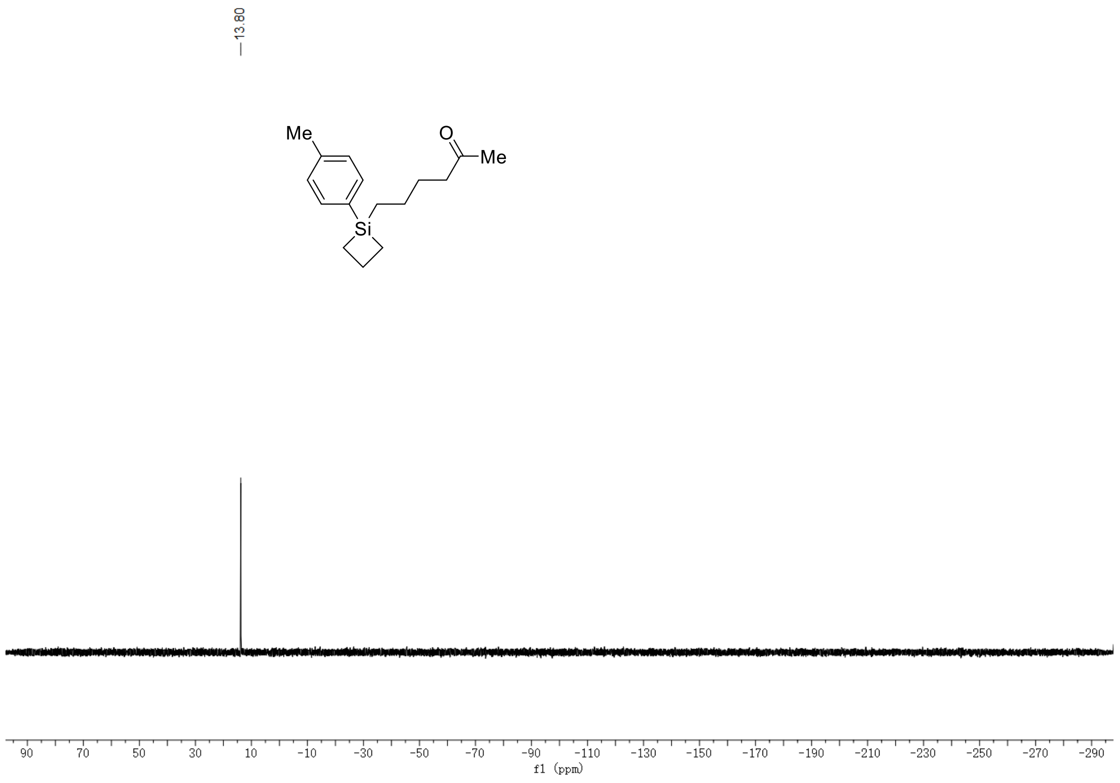

^{1}H NMR of **3b** (400 MHz, $CDCl_3$, 25 ºC)

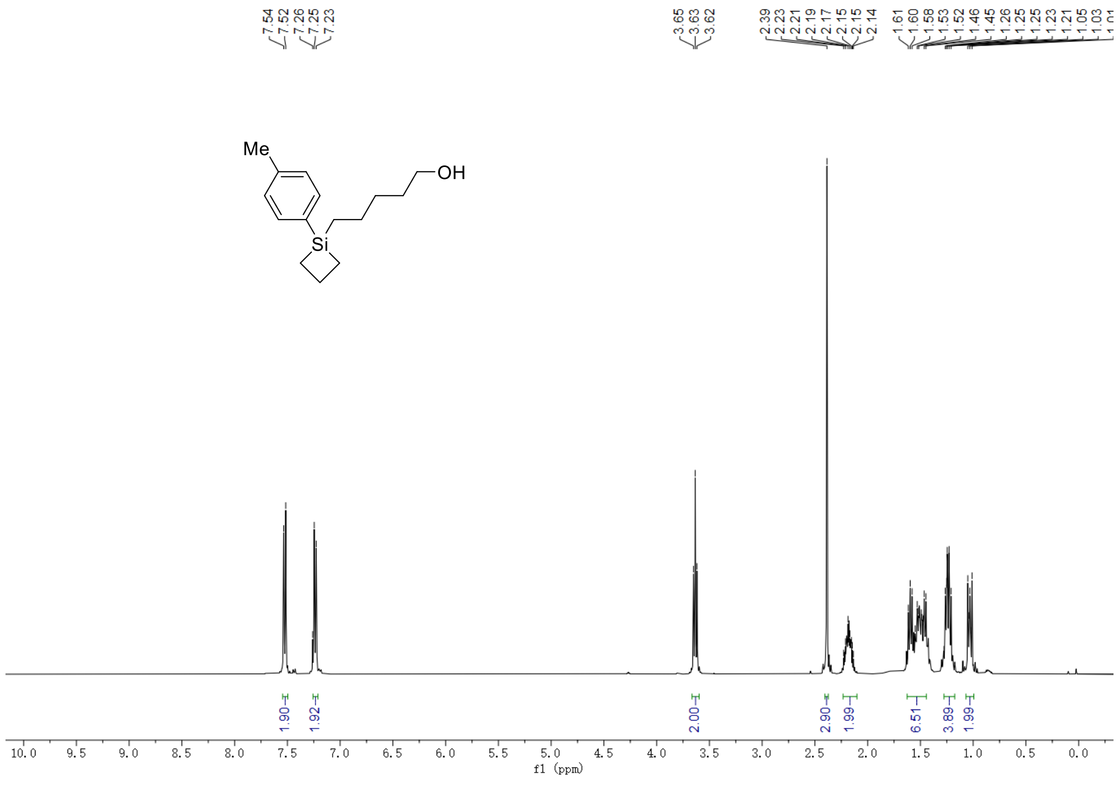

^{13}C NMR of **3b** (151 MHz, $CDCl_3$, 25 ºC)

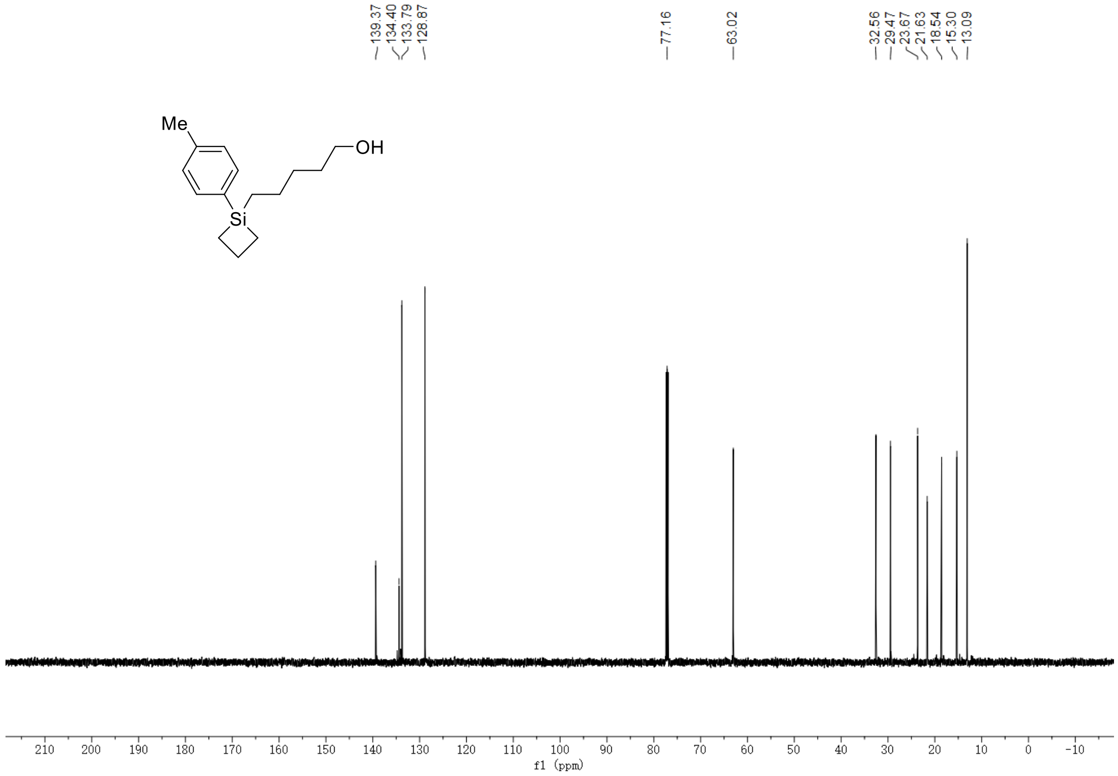

^{29}Si NMR of **3b** (119 MHz, $CDCl_3$, 25 ºC)

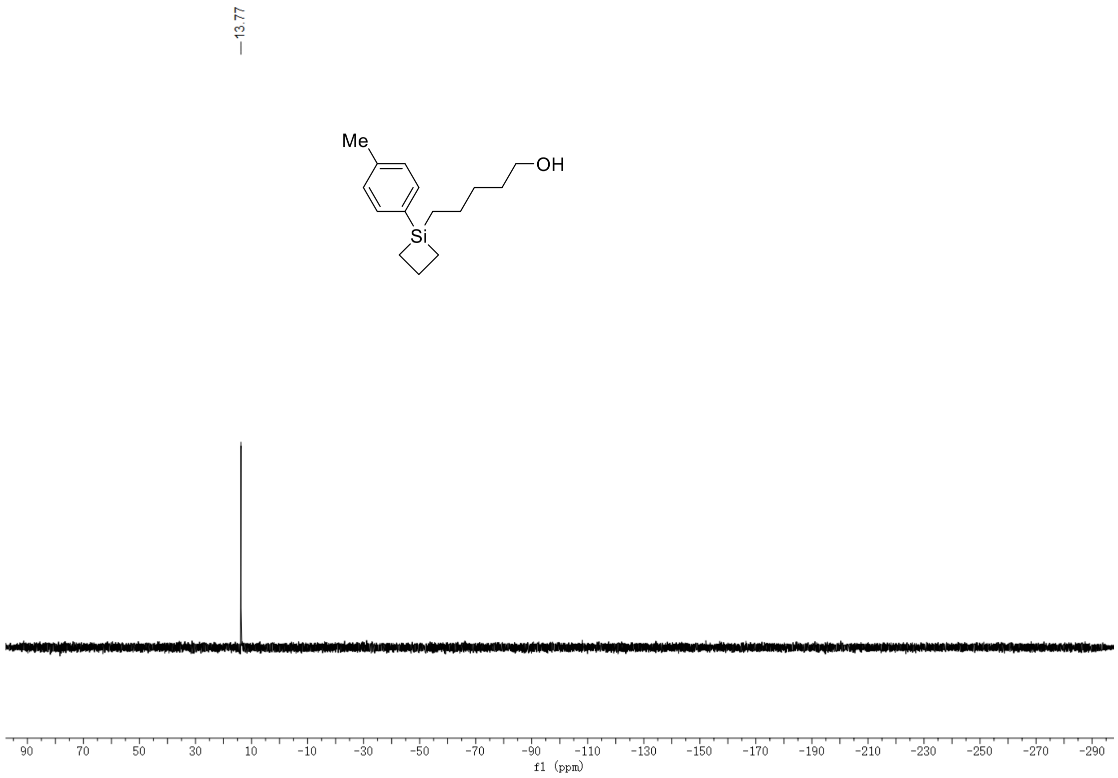

^{1}H NMR of **3c** (600 MHz, $CDCl_3$, 25 ºC)

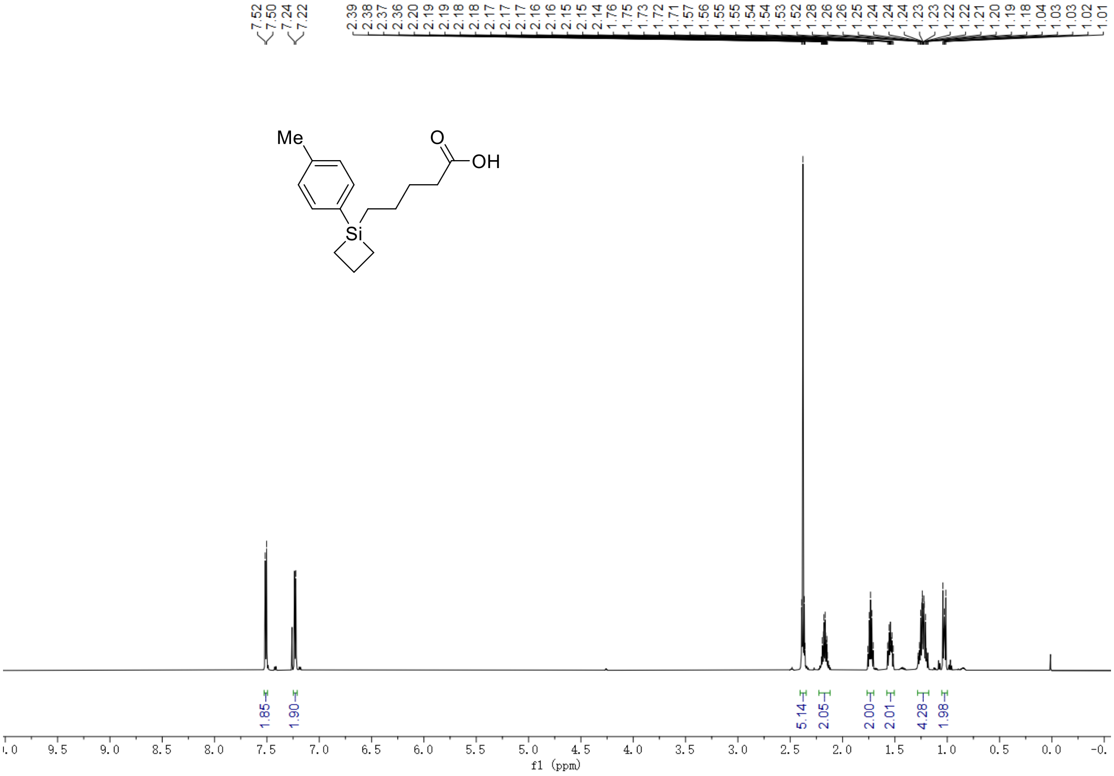

^{13}C NMR of **3c** (151 MHz, $CDCl_3$, 25 ºC)

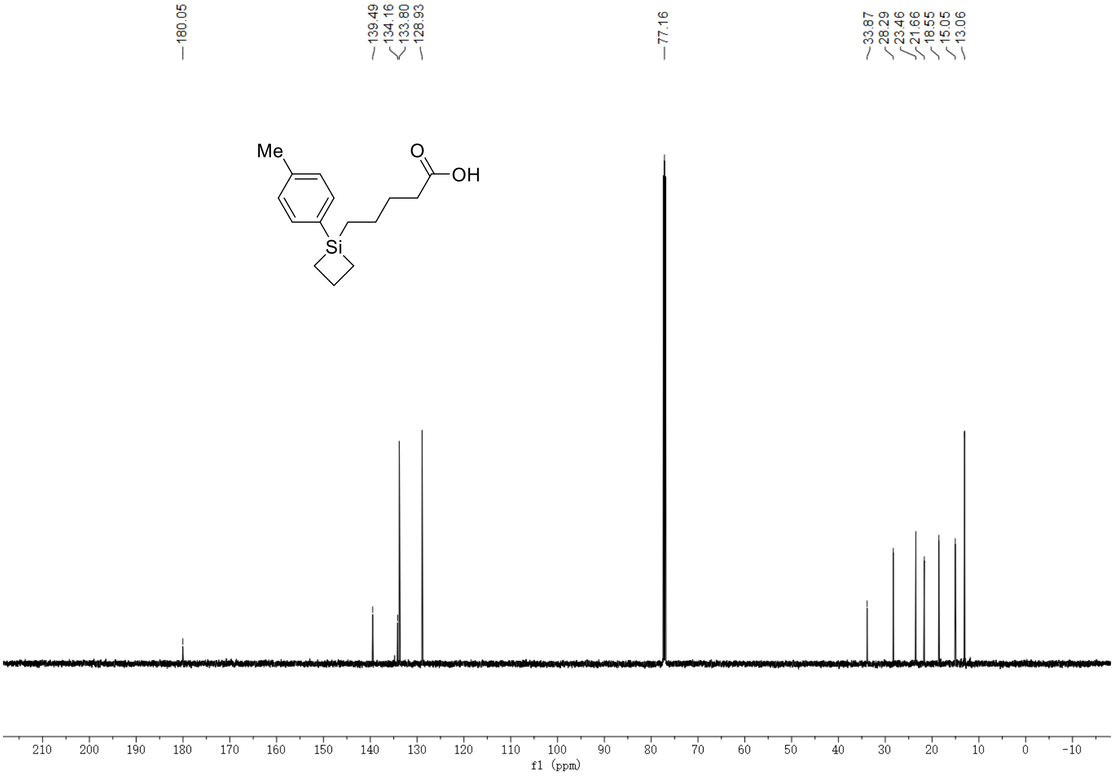

^{29}Si NMR of **3c** (119 MHz, $CDCl_3$, 25 ºC)

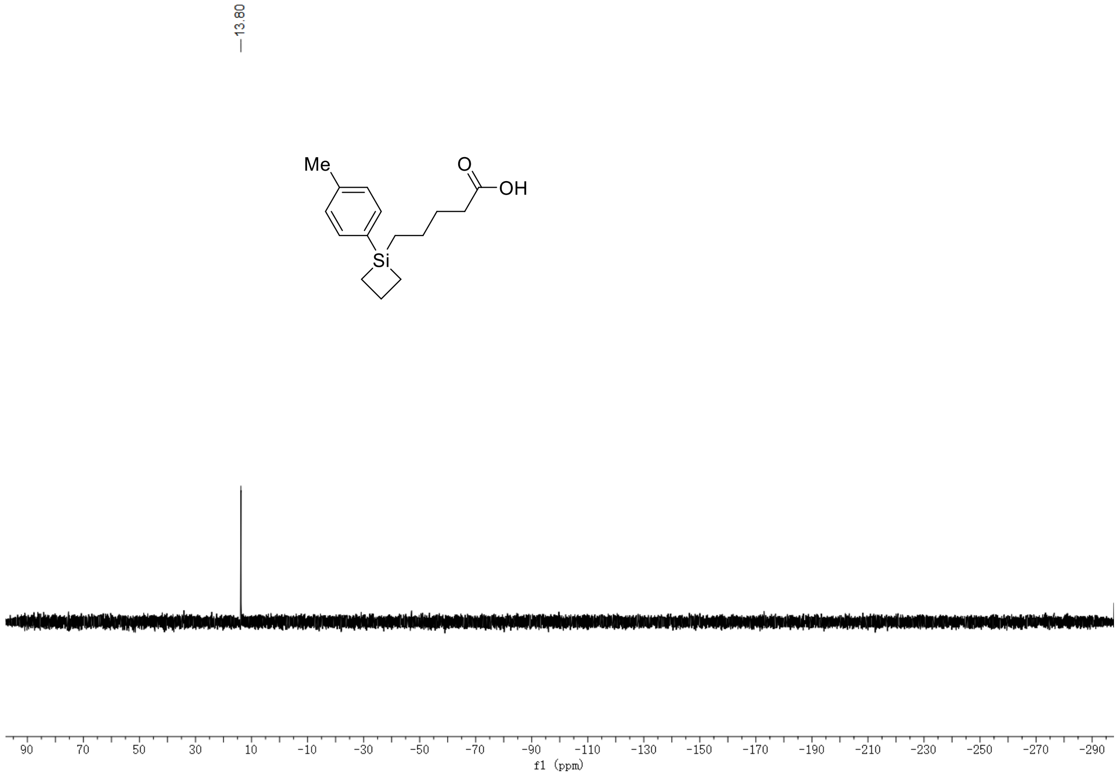

^{1}H NMR of **3d** (400 MHz, $CDCl_3$, 25 ºC)

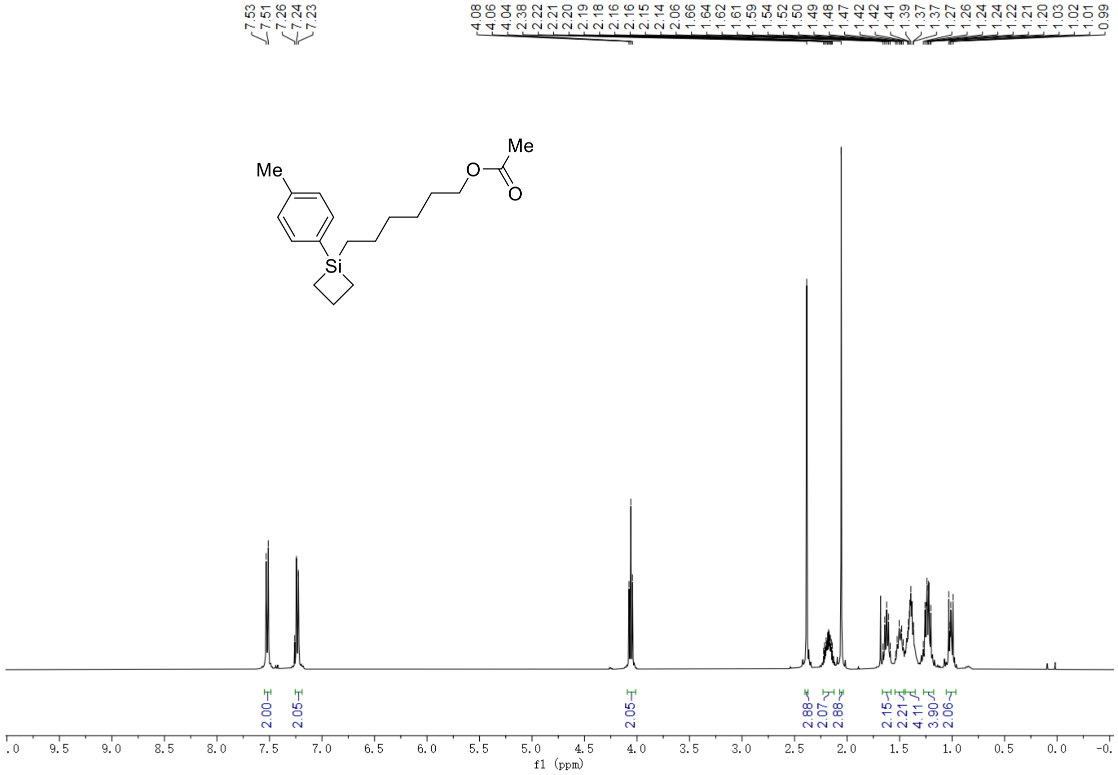

^{13}C NMR of **3d** (101 MHz, $CDCl_3$, 25 ºC)

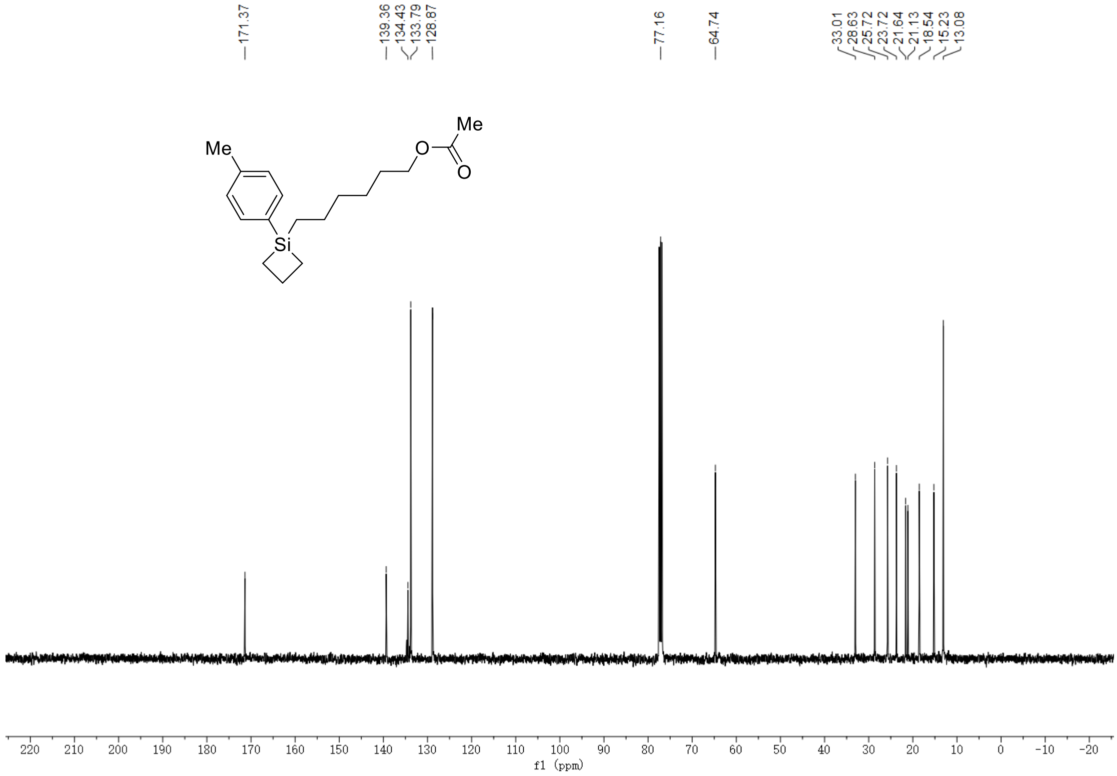

^{29}Si NMR of **3d** (119 MHz, $CDCl_3$, 25 ºC)

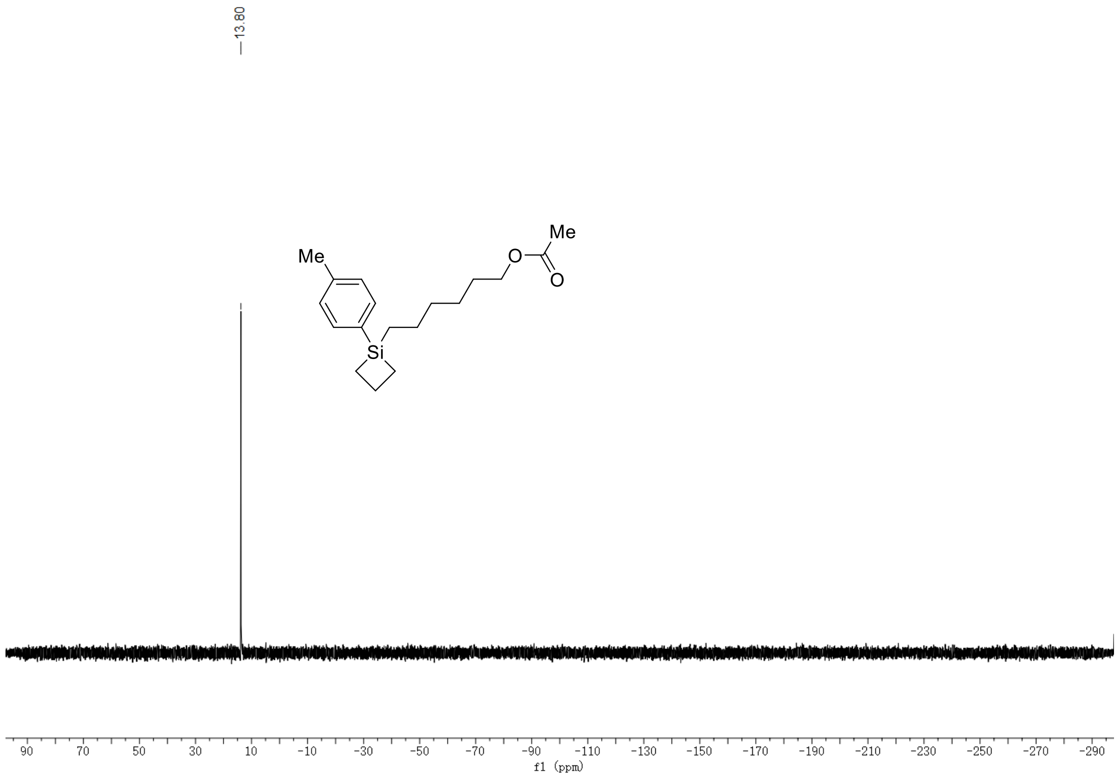

^{1}H NMR of **3e** (400 MHz, $CDCl_3$, 25 ºC)

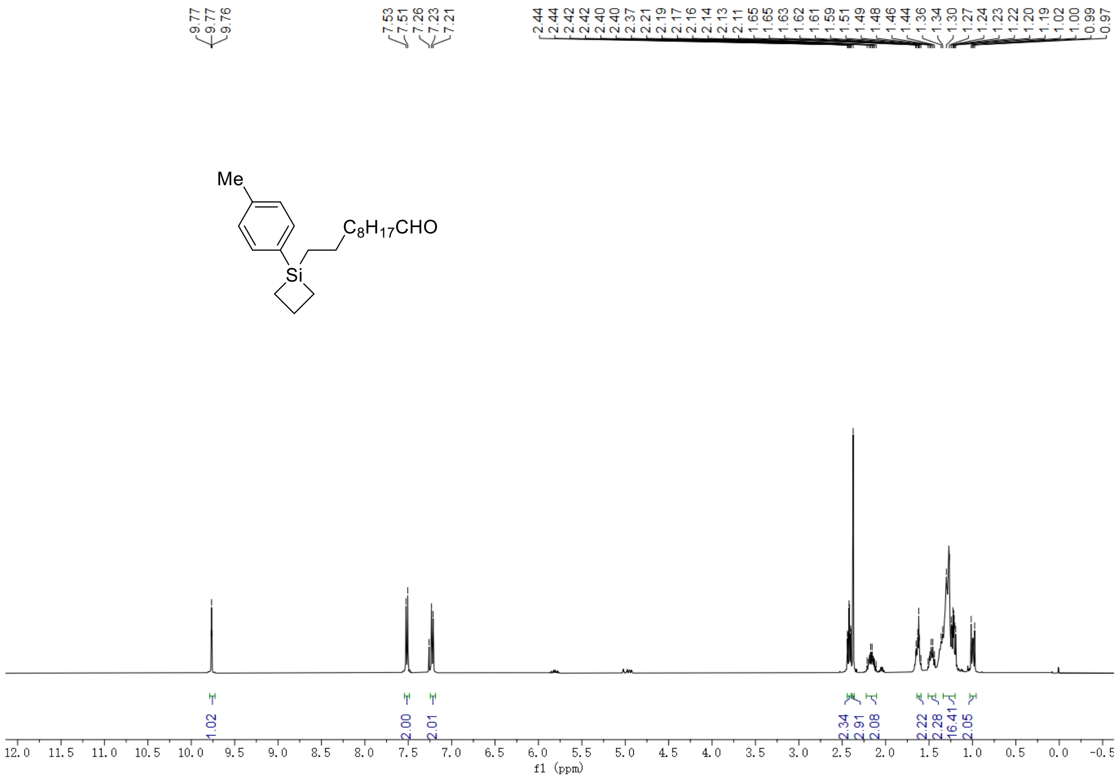

^{13}C NMR of **3e** (101 MHz, $CDCl_3$, 25 ºC)

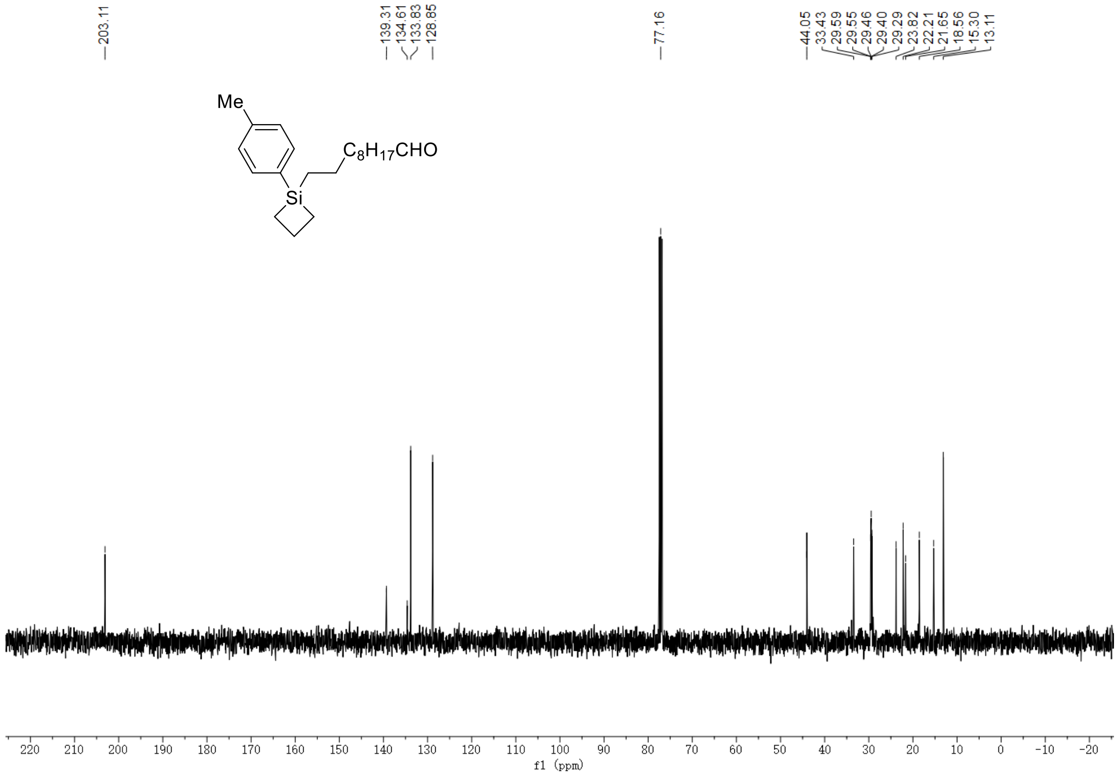

^{29}Si NMR of **3e** (119 MHz, $CDCl_3$, 25 ºC)

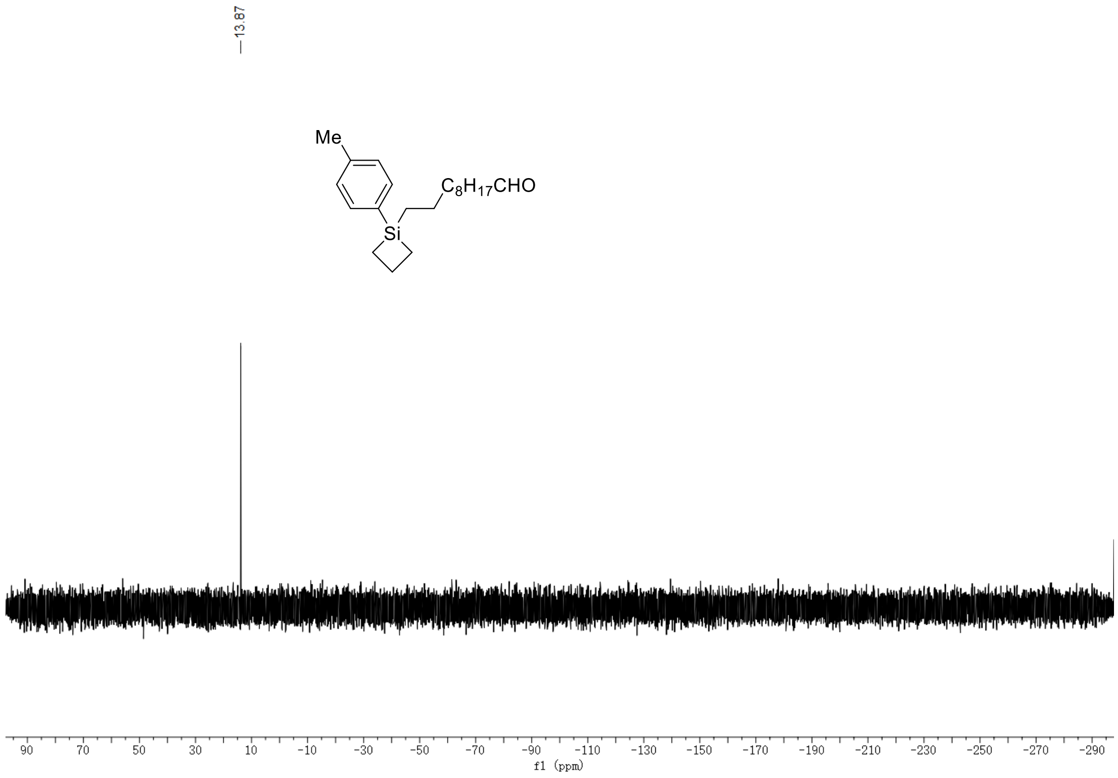

^{1}H NMR of **3f** (400 MHz, $CDCl_3$, 25 ºC)

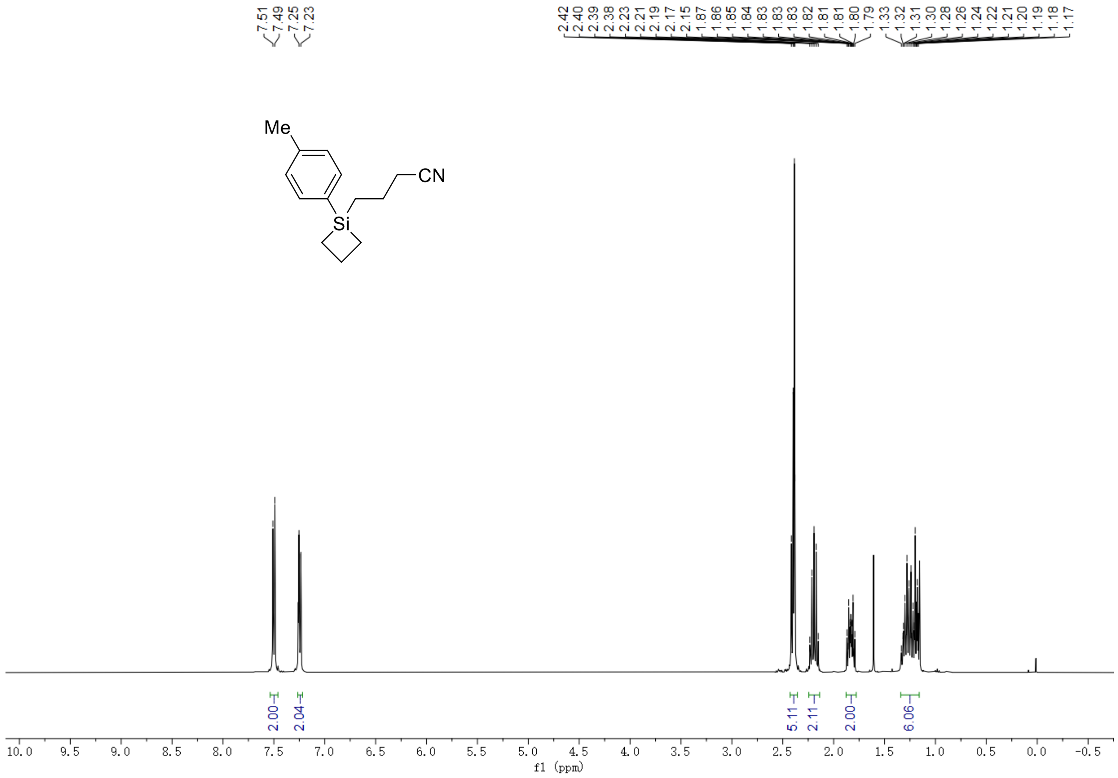

^{13}C NMR of **3f** (151 MHz, $CDCl_3$, 25 ºC)

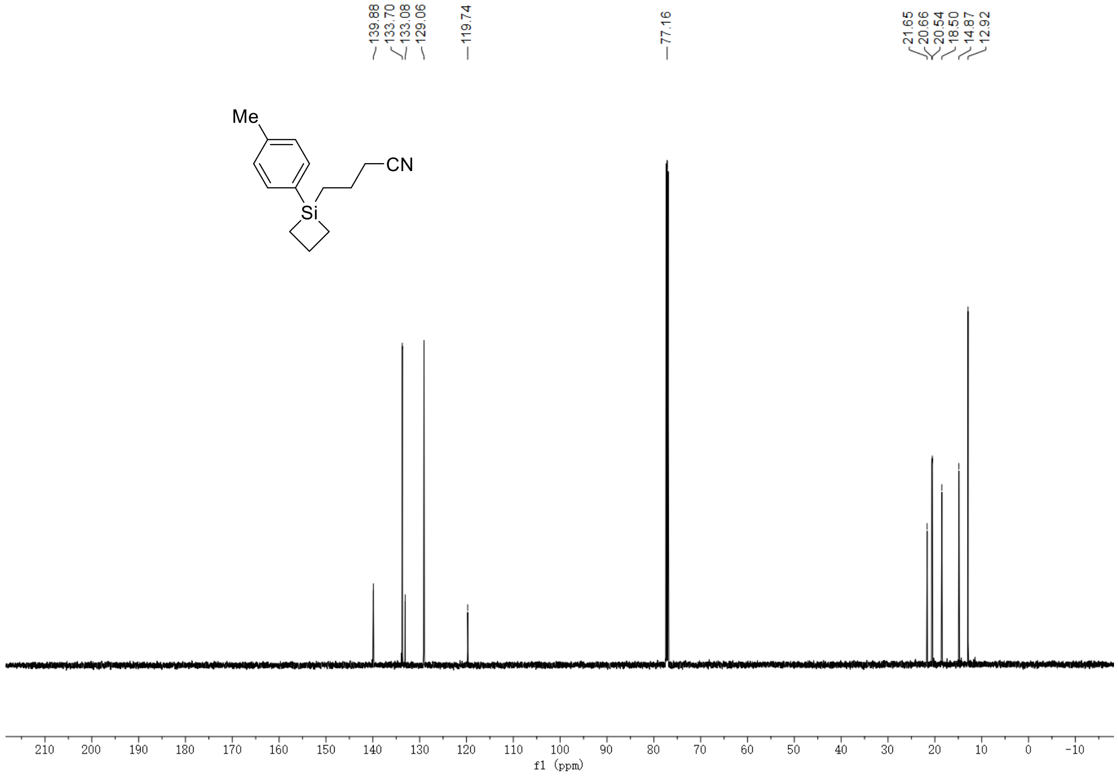

^{29}Si NMR of **3f** (119 MHz, $CDCl_3$, 25 ºC)

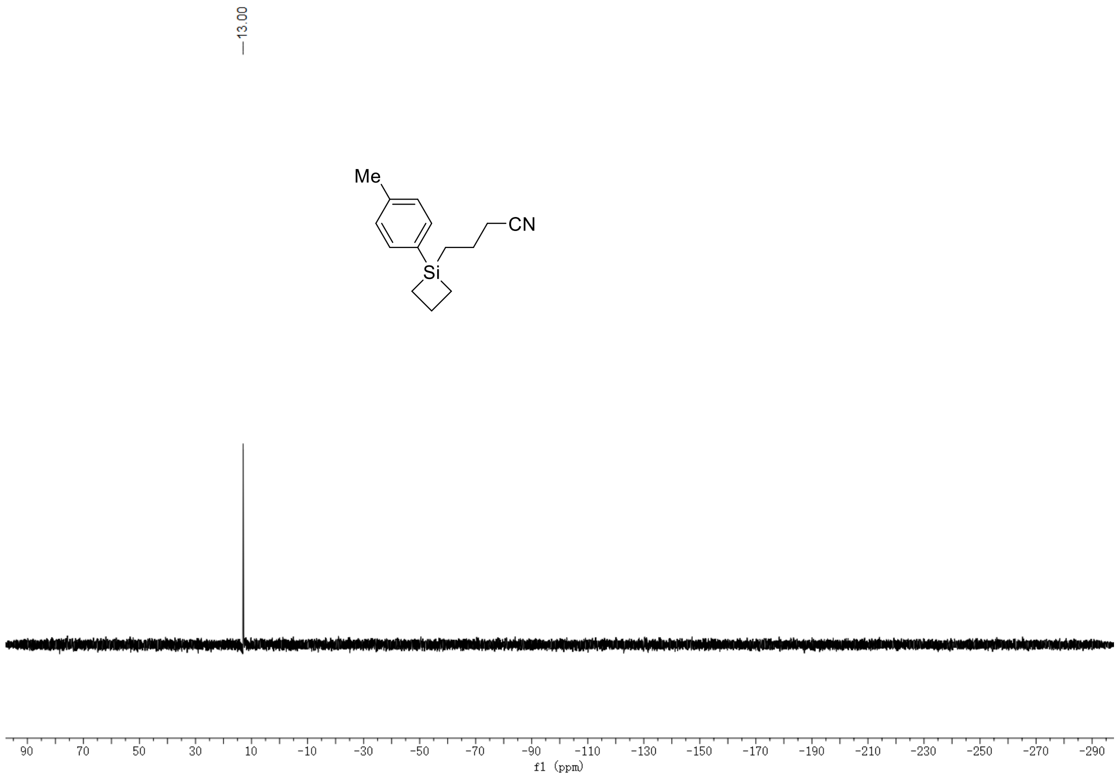

^{1}H NMR of **3g** (400 MHz, $CDCl_3$, 25 ºC)

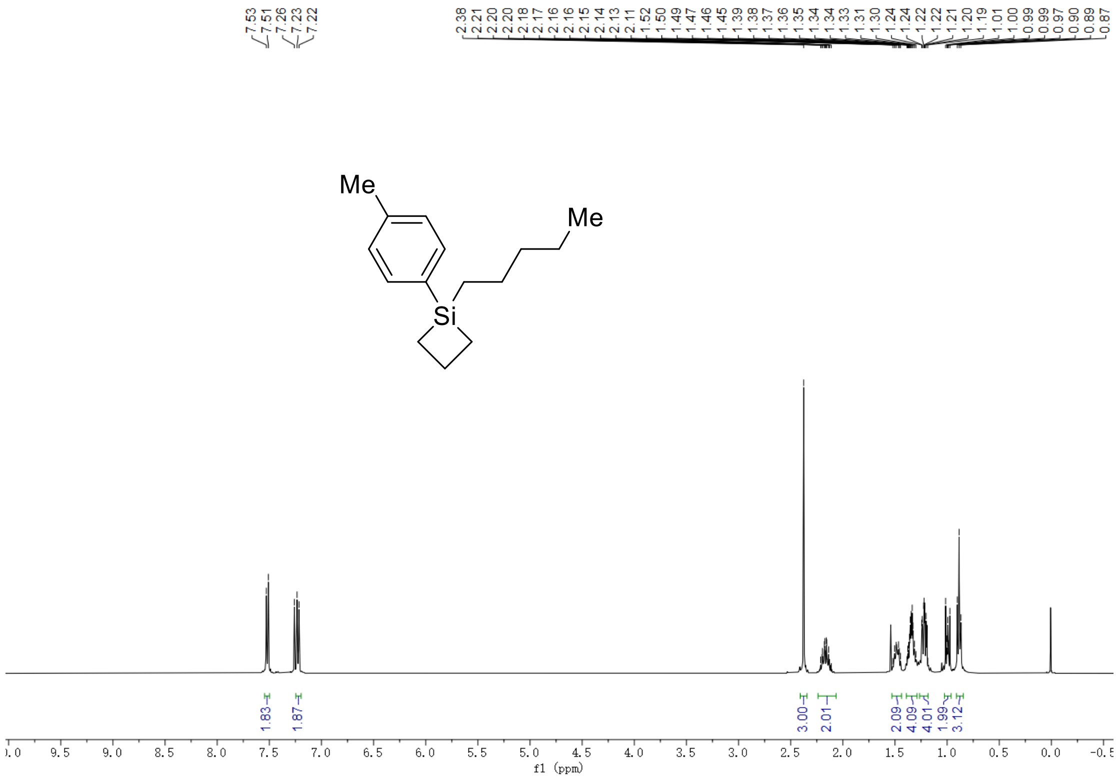

^{13}C NMR of **3g** (151 MHz, $CDCl_3$, 25 ºC)

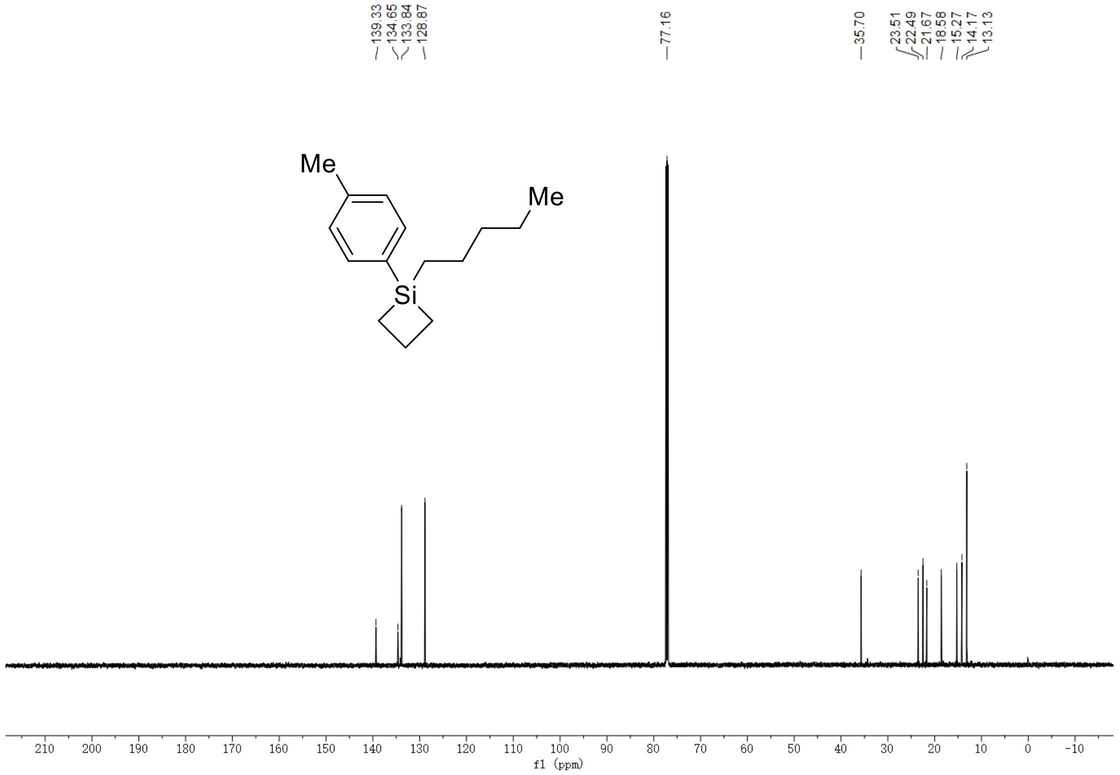

^{29}Si NMR of **3g** (119 MHz, $CDCl_3$, 25 ºC)

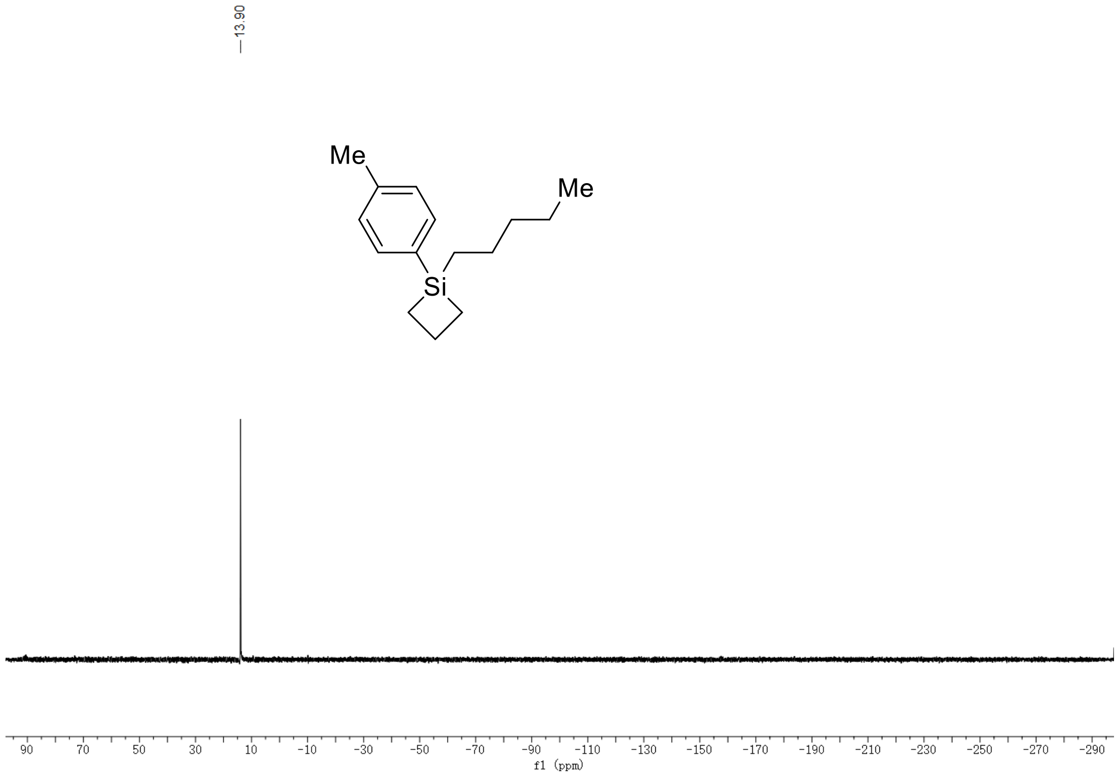

^{1}H NMR of **3h** (600 MHz, $CDCl_3$, 25 ºC)

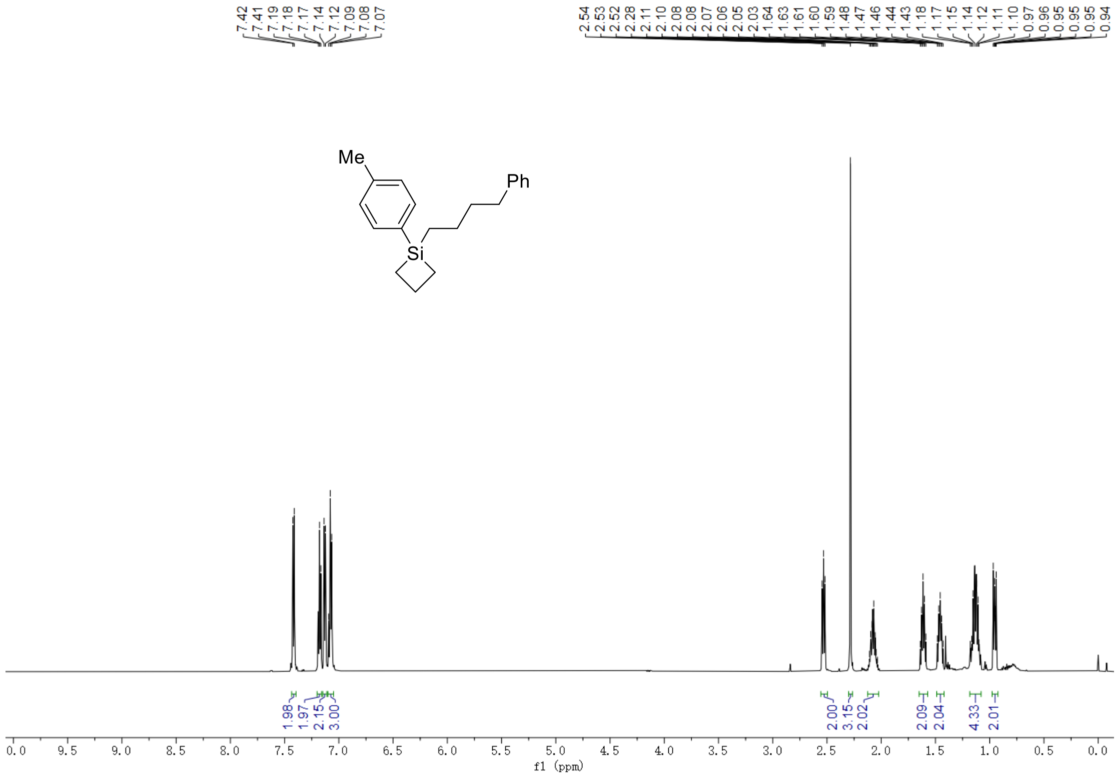

^{13}C NMR of **3h** (151 MHz, $CDCl_3$, 25 ºC)

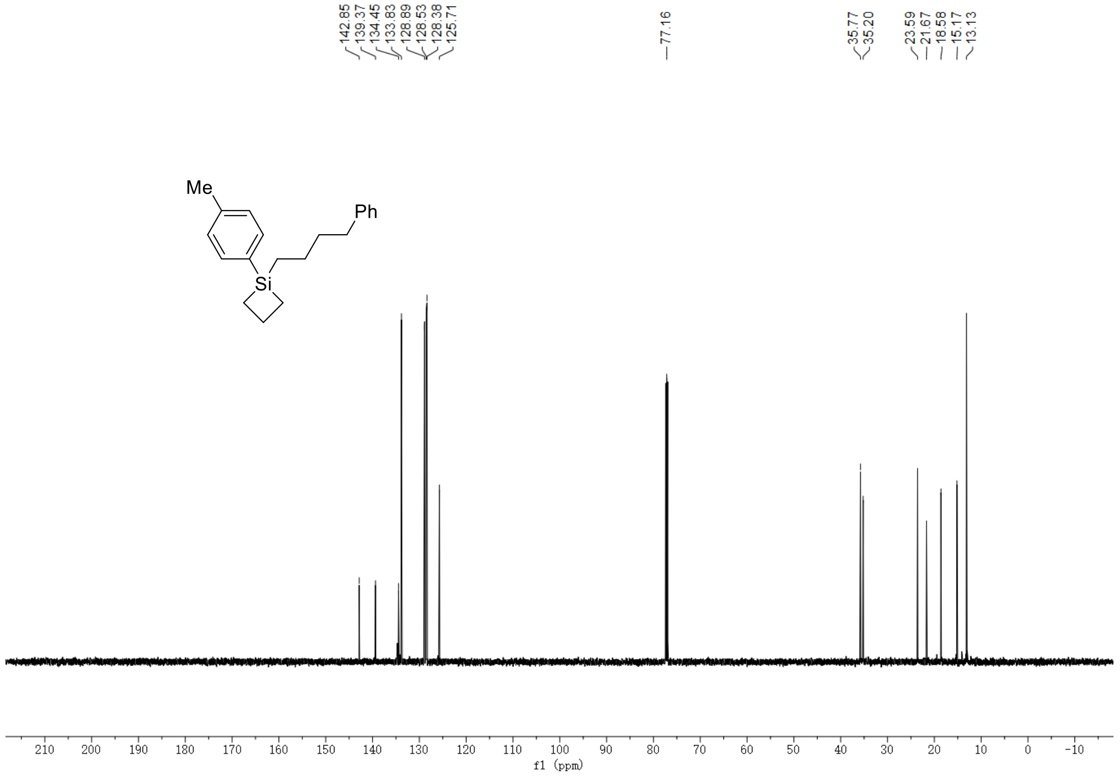

^{29}Si NMR of **3h** (119 MHz, $CDCl_3$, 25 ºC)

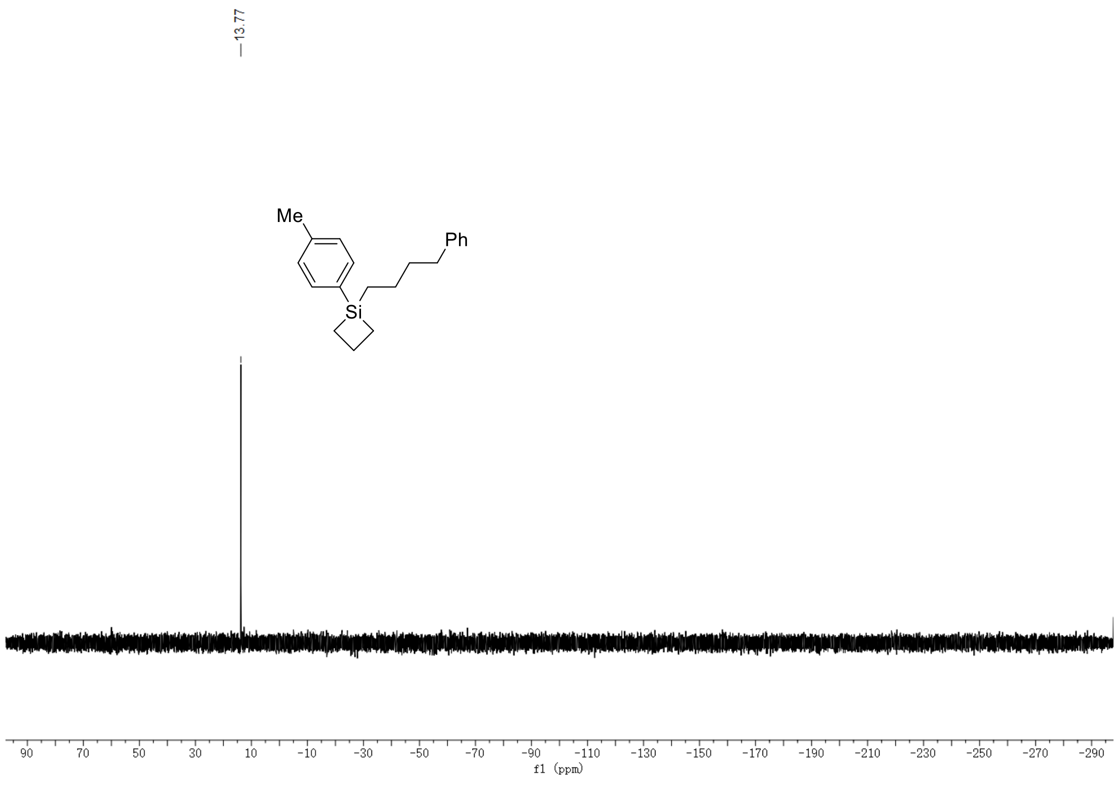

^{1}H NMR of **3i** (400 MHz, $CDCl_3$, 25 ºC)

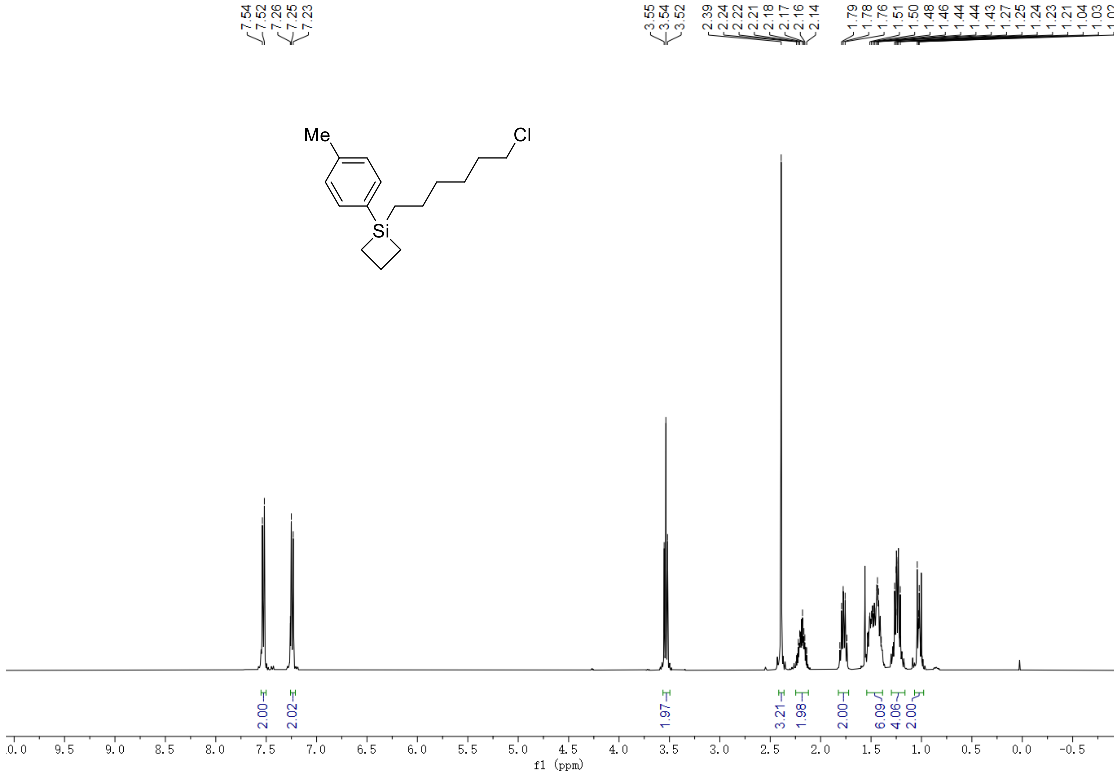

^{13}C NMR of **3i** (151 MHz, $CDCl_3$, 25 ºC)

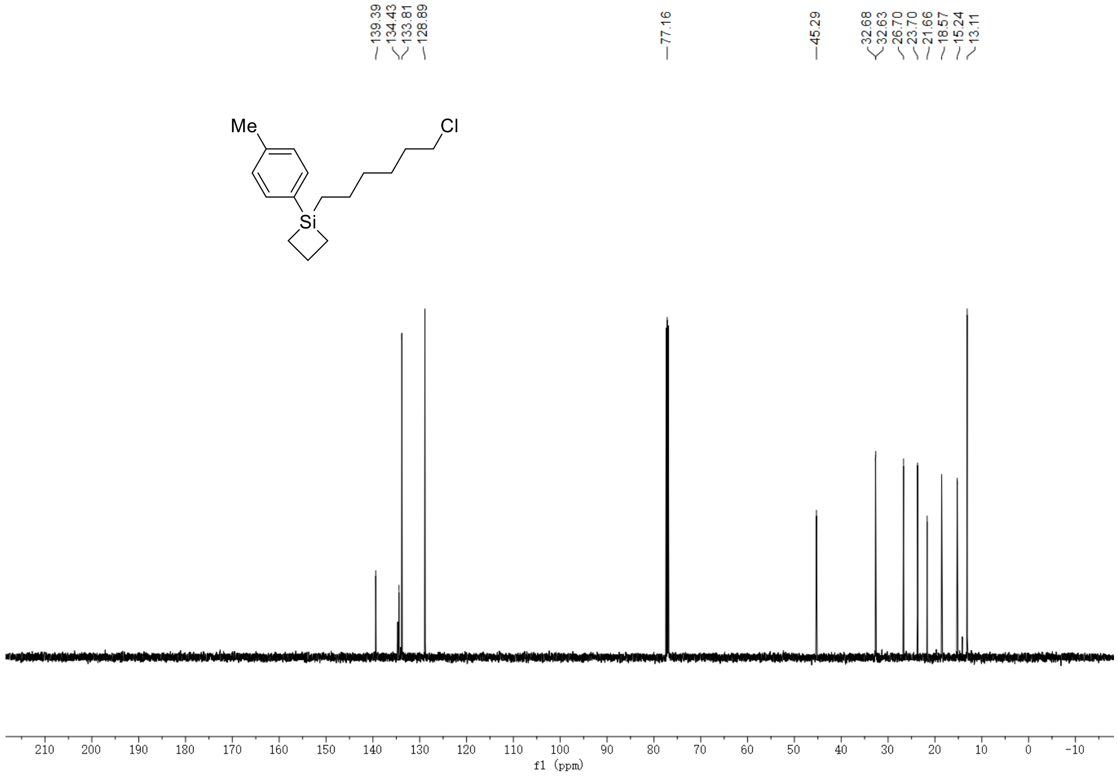

^{29}Si NMR of **3i** (119 MHz, $CDCl_3$, 25 ºC)

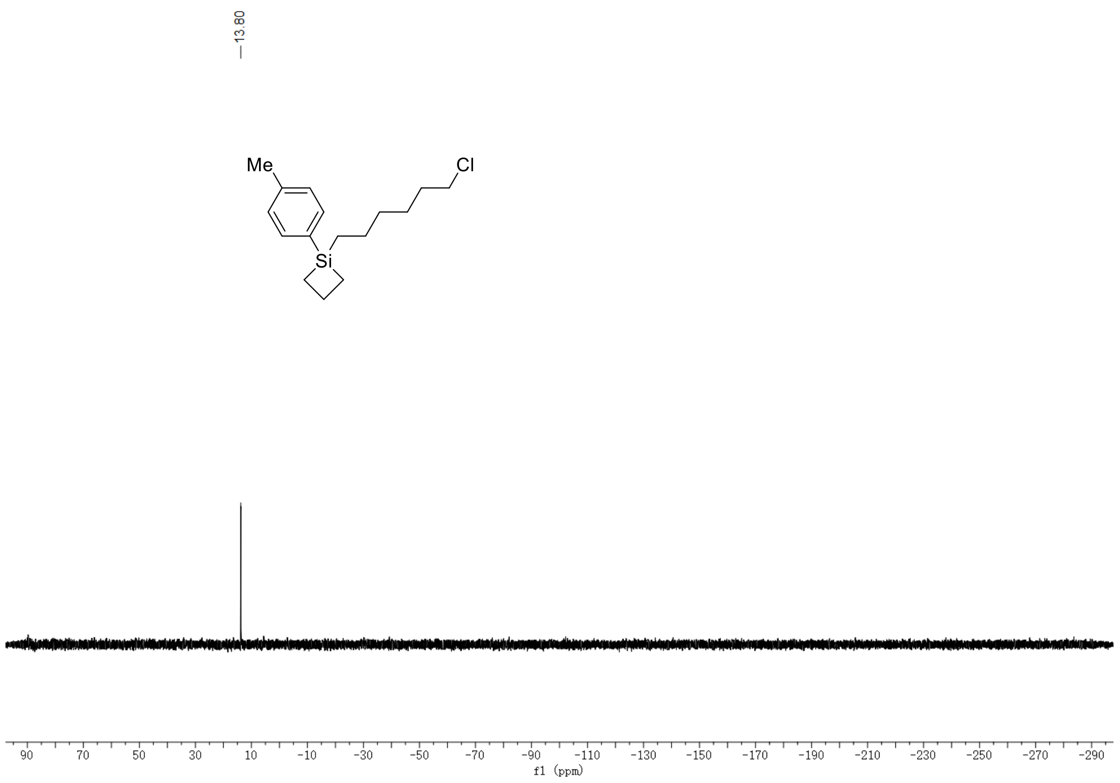

^{1}H NMR of **3j** (600 MHz, $CDCl_3$, 25 ºC)

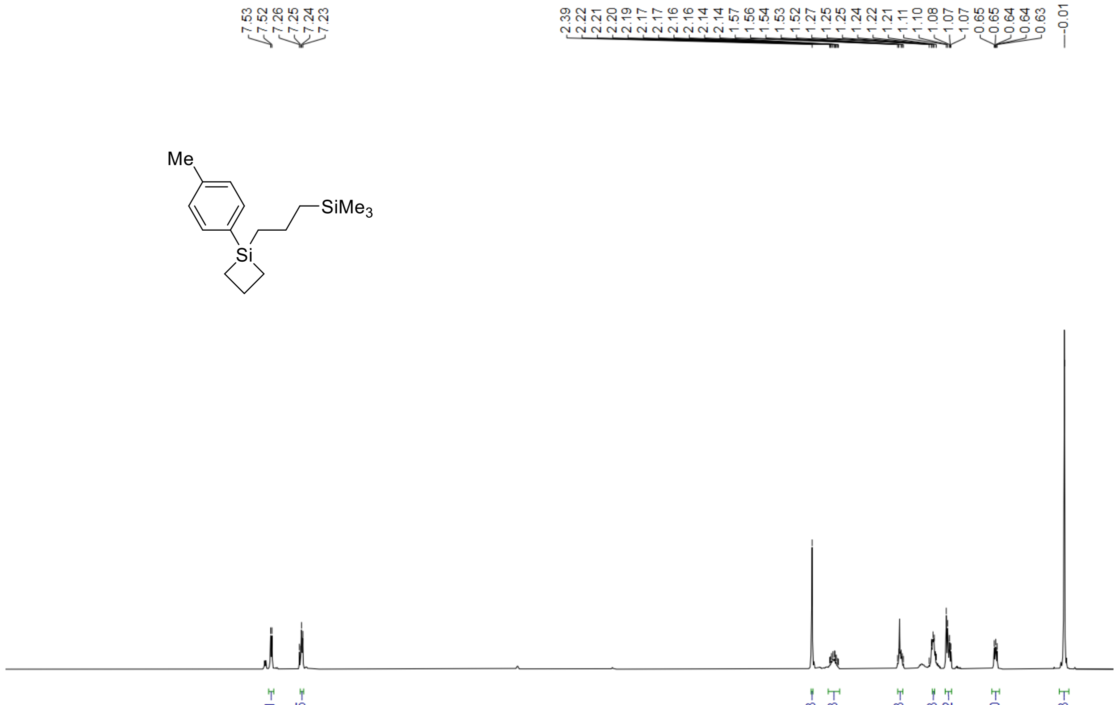

^{13}C NMR of **3j** (101 MHz, $CDCl_3$, 25 ºC)

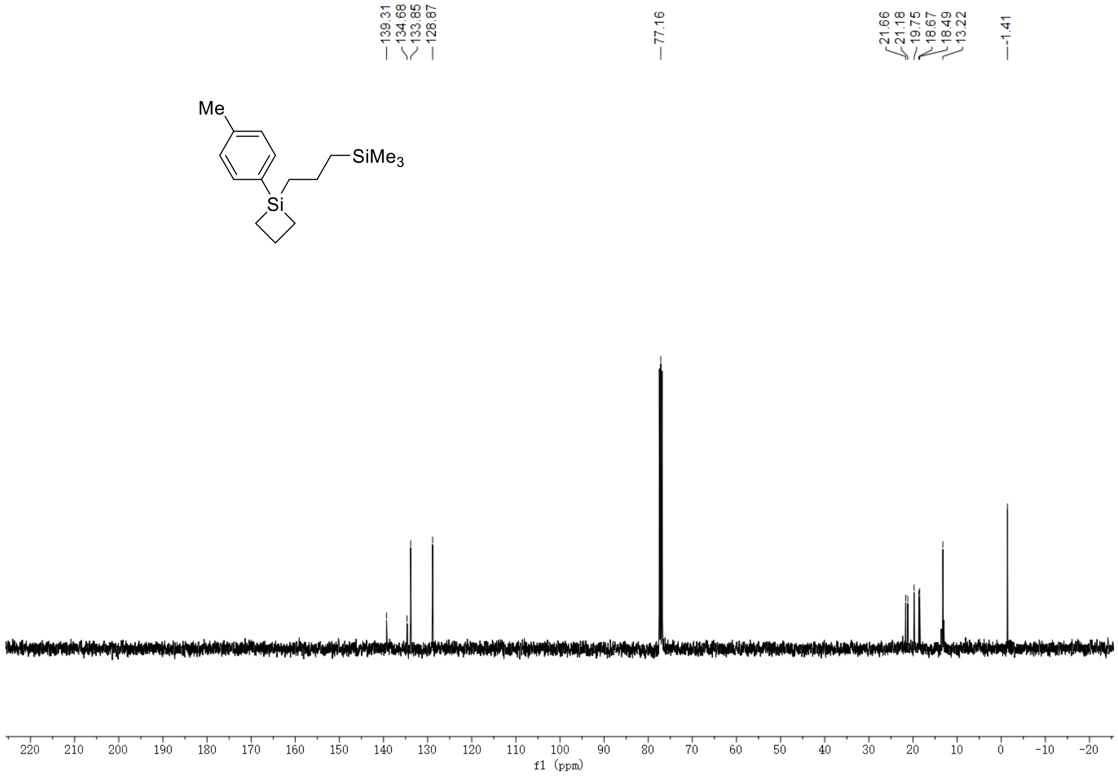

^{29}Si NMR of **3j** (119 MHz, $CDCl_3$, 25 ºC)

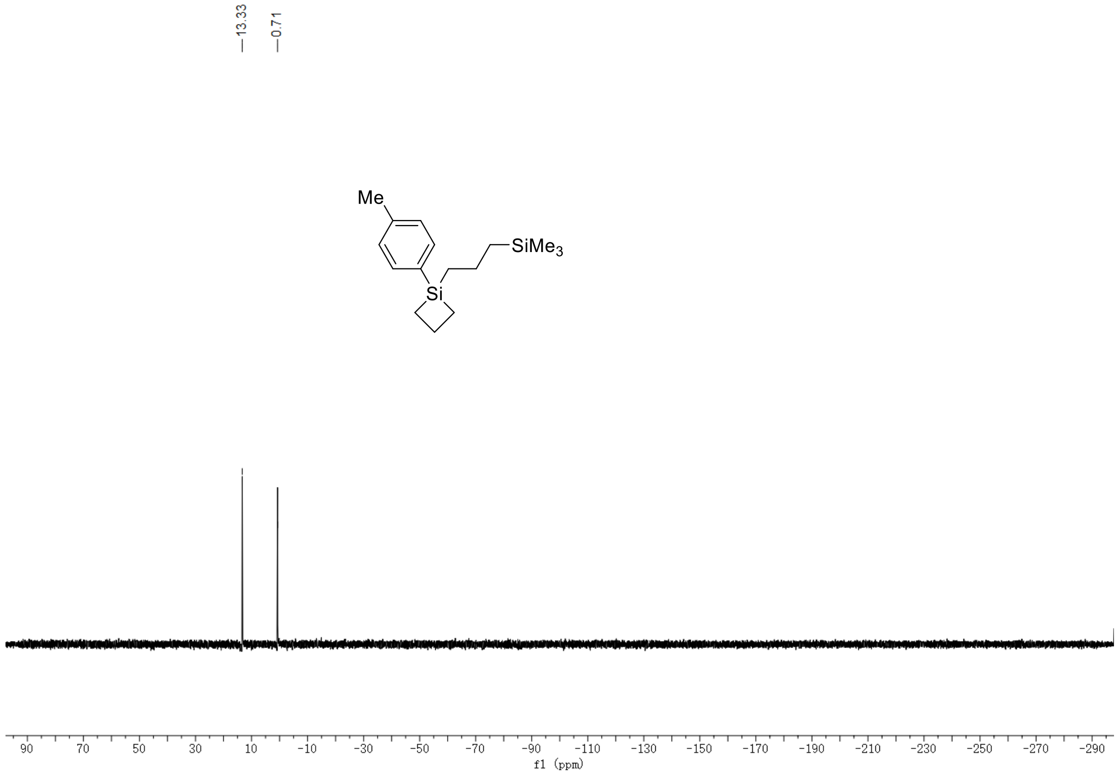

^{1}H NMR of **3k** (400 MHz, $CDCl_3$, 25 ºC)

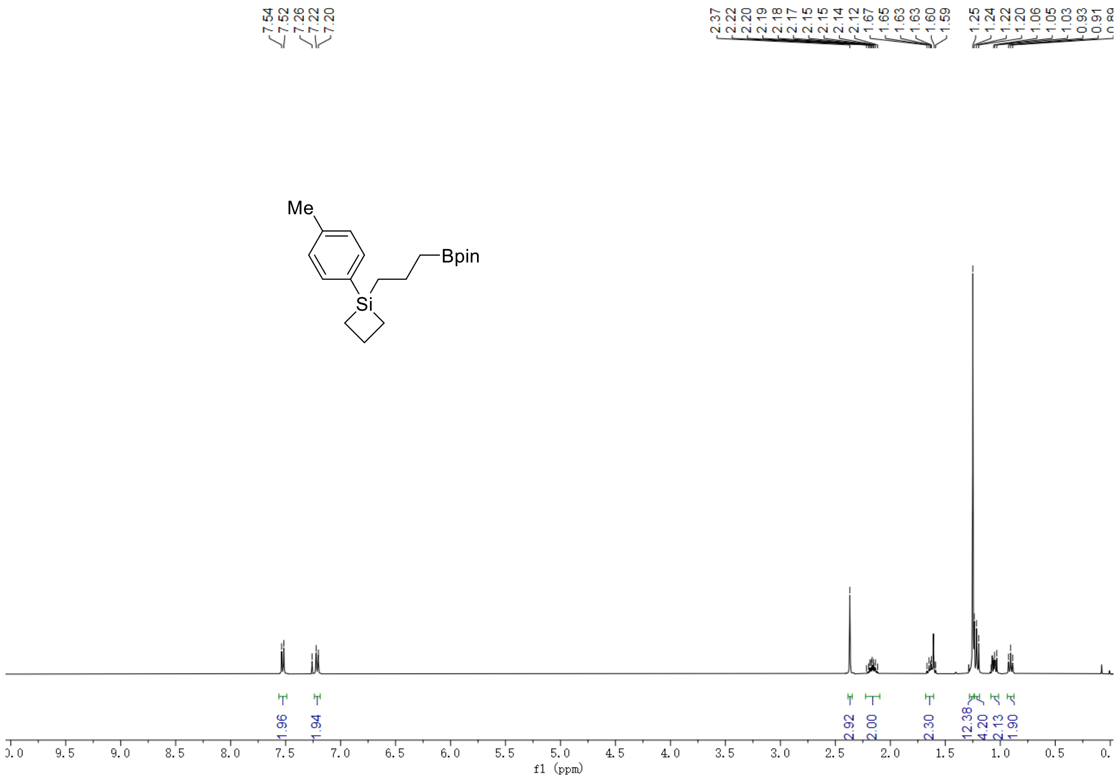

^{13}C NMR of **3k** (151 MHz, $CDCl_3$, 25 ºC)

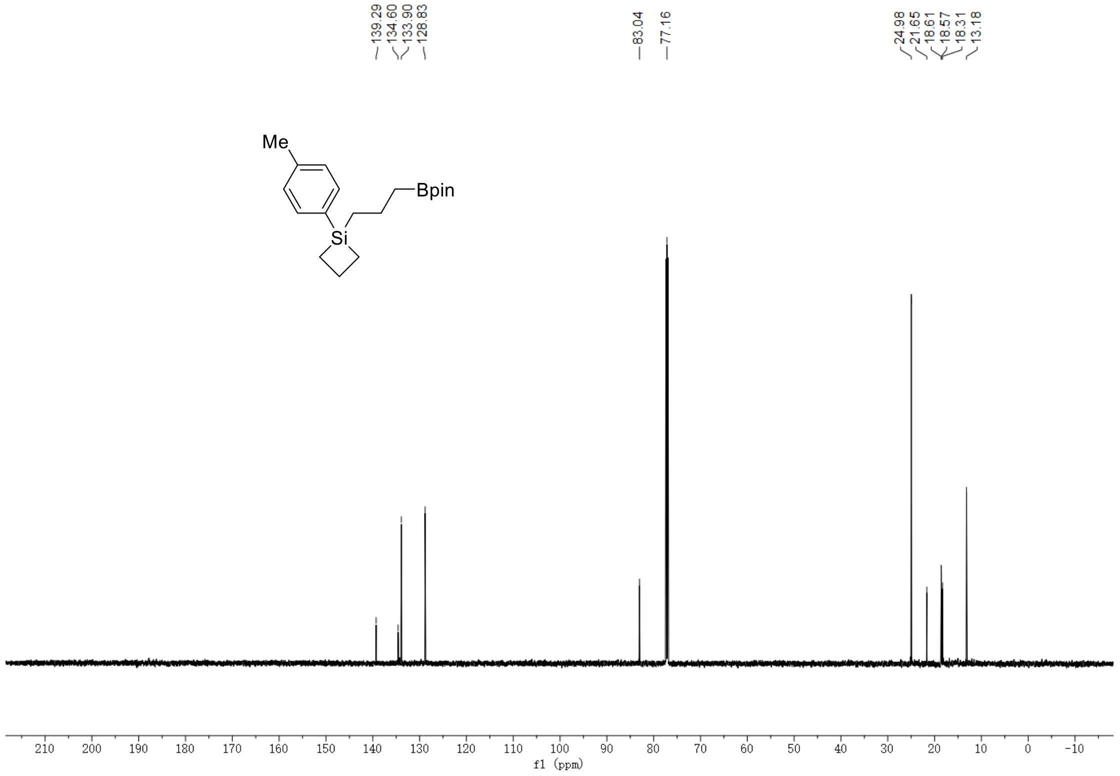

^{29}Si NMR of **3k** (119 MHz, $CDCl_3$, 25 ºC)

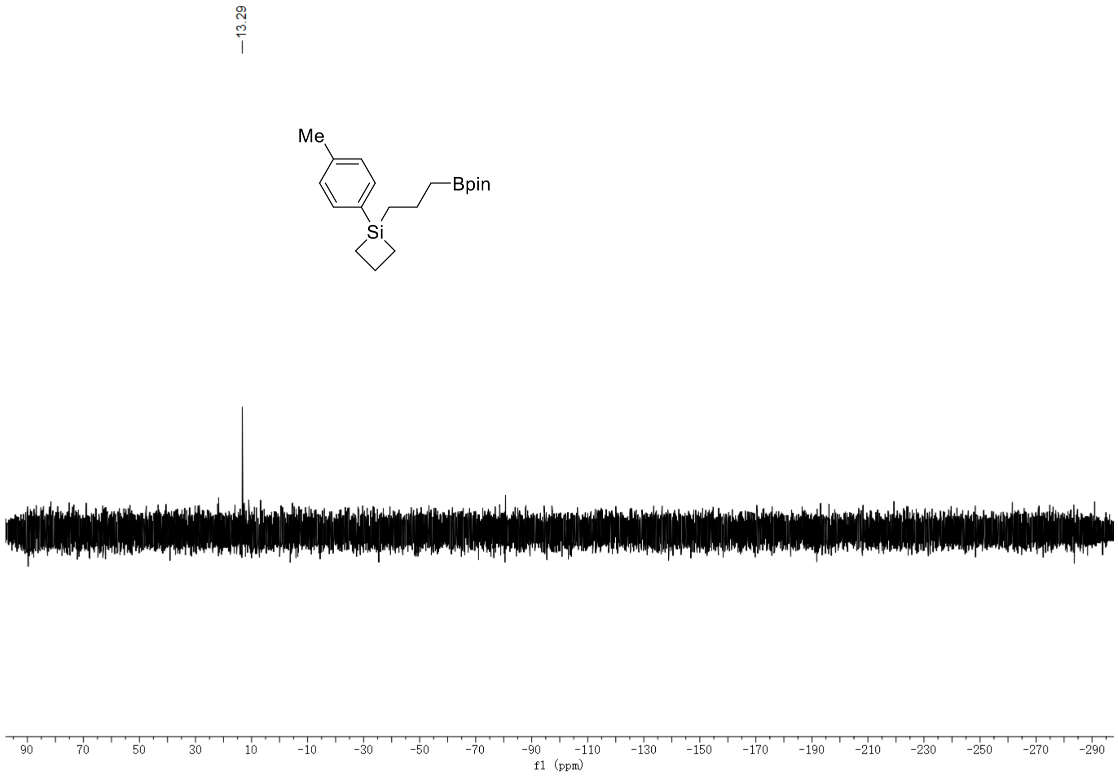

^{1}H NMR of **3l** (600 MHz, $CDCl_3$, 25 ºC)

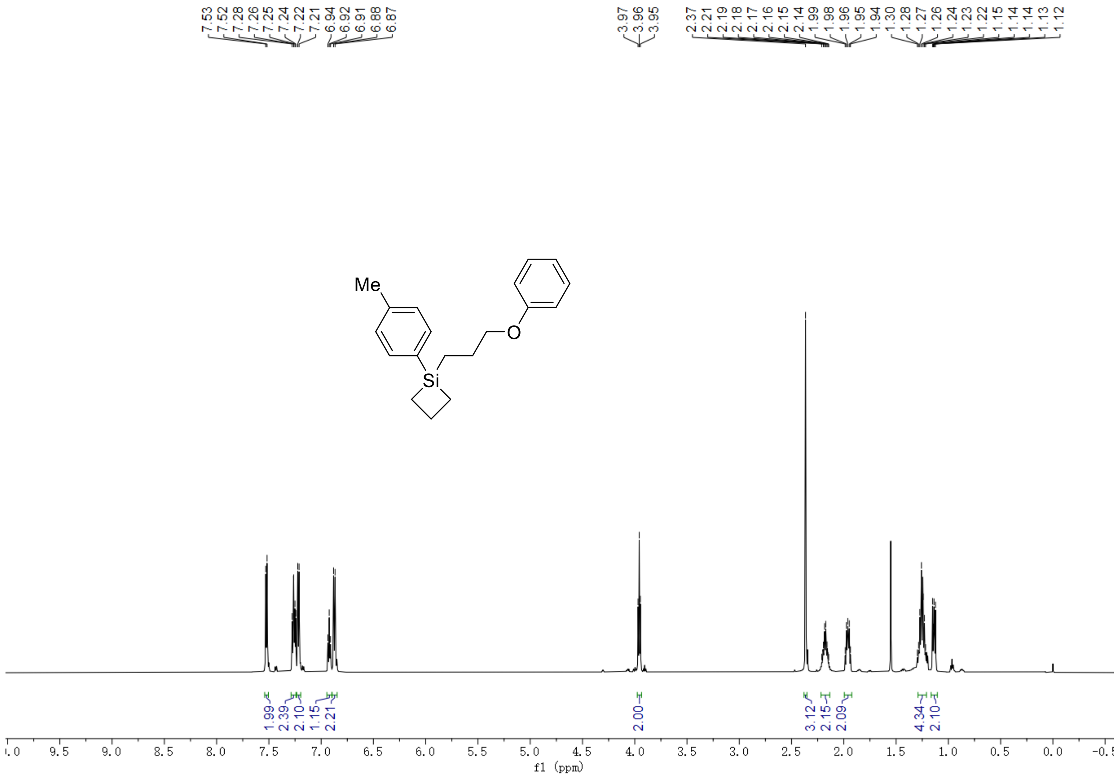

^{13}C NMR of **3l** (151 MHz, $CDCl_3$, 25 ºC)

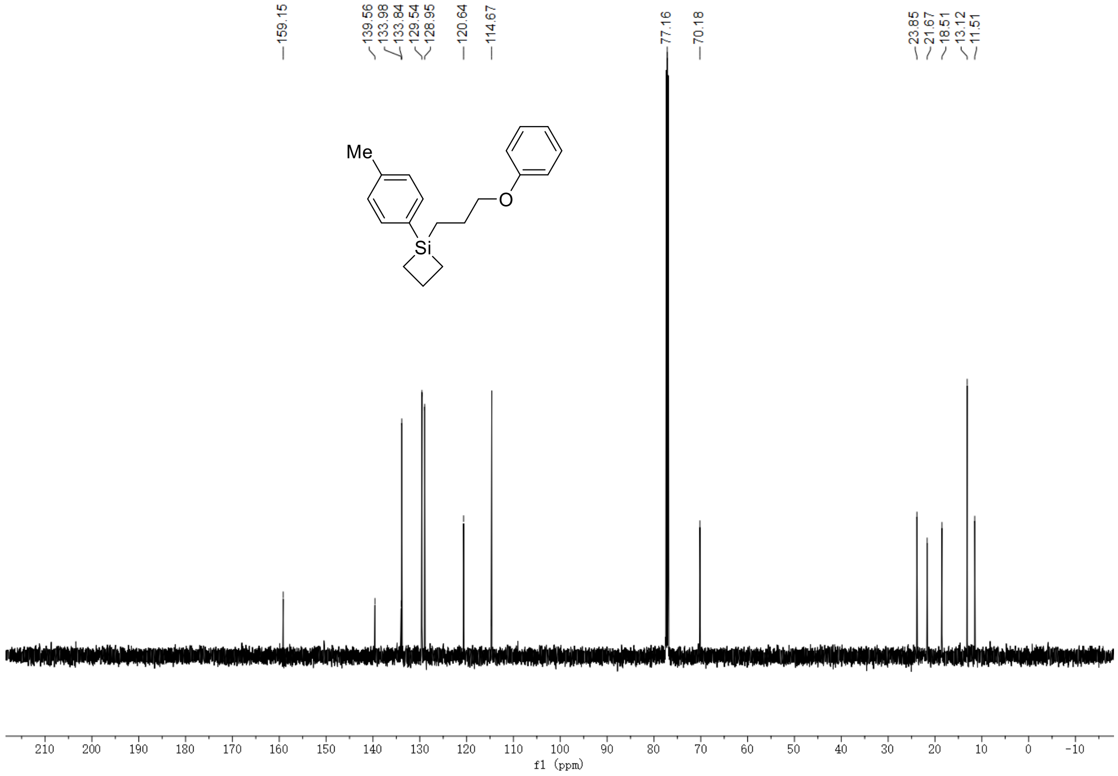

^{29}Si NMR of **3l** (119 MHz, $CDCl_3$, 25 ºC)

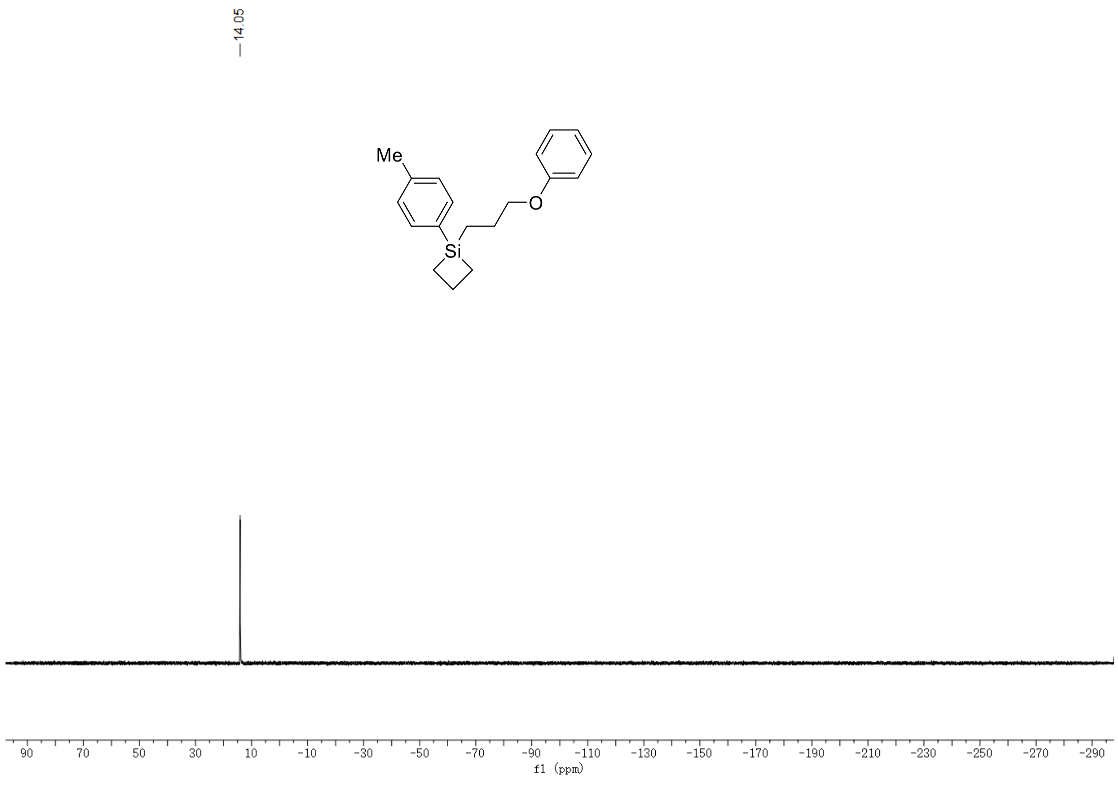

^{1}H NMR of **3m** (400 MHz, $CDCl_3$, 25 ºC)

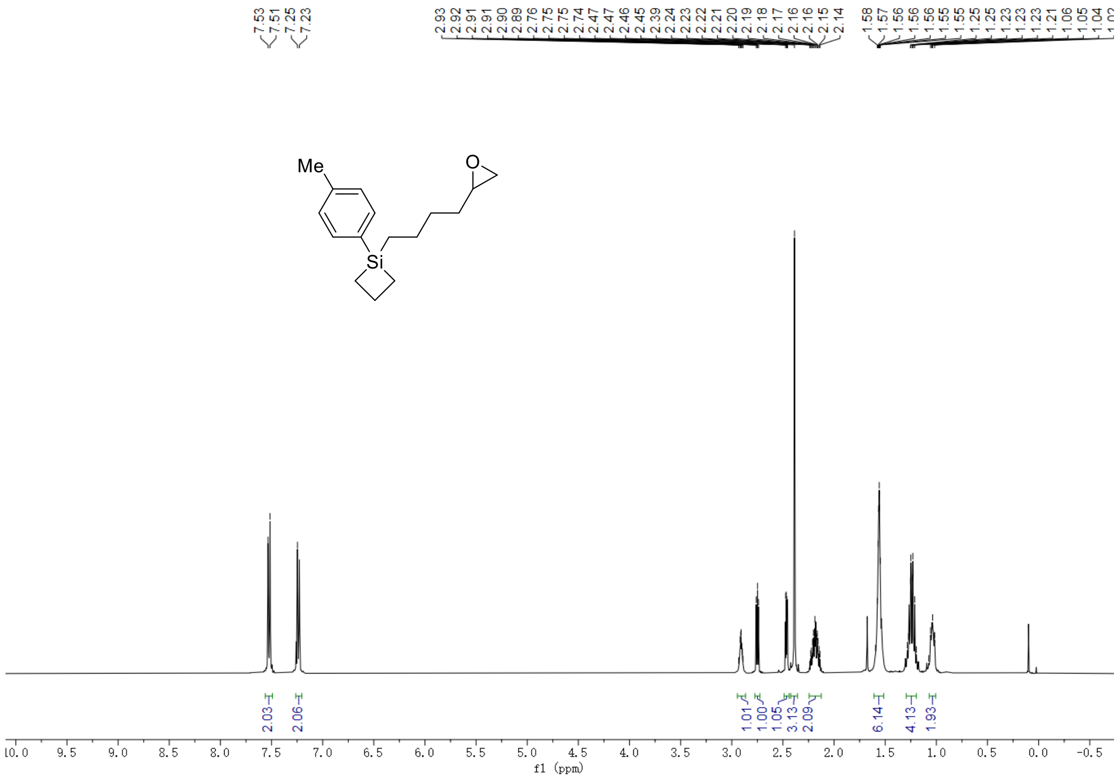

^{13}C NMR of **3m** (151 MHz, $CDCl_3$, 25 ºC)

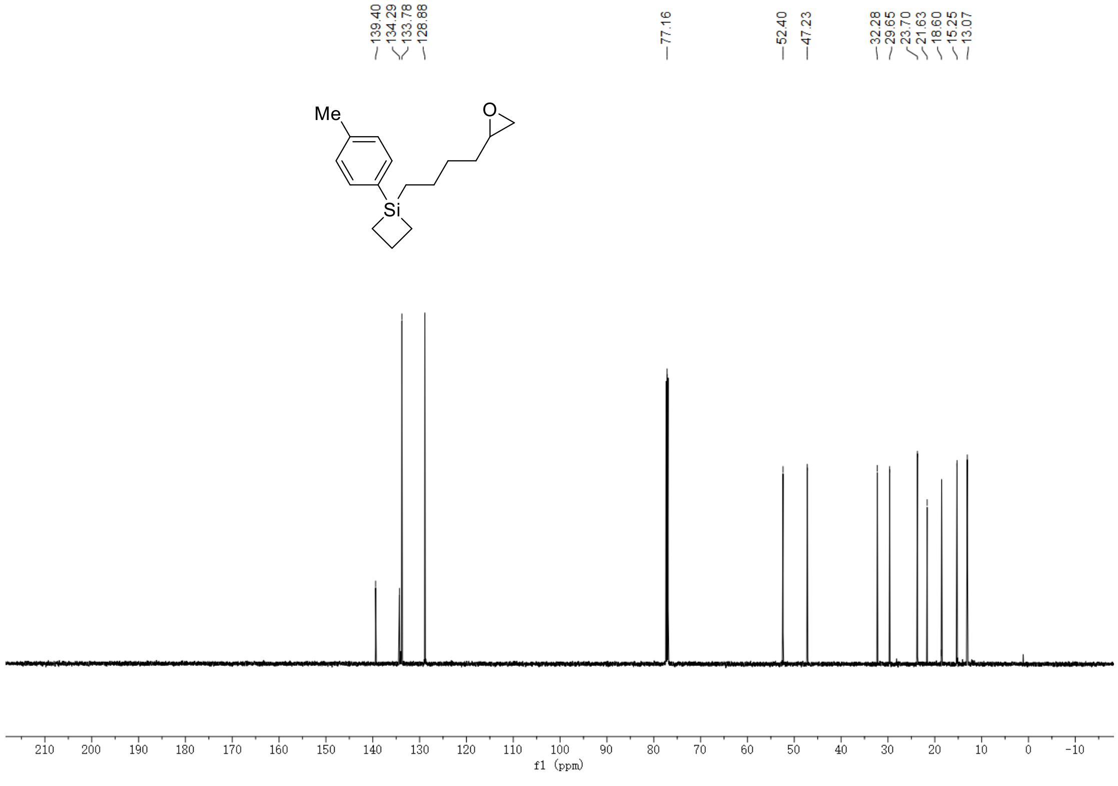

^{29}Si NMR of **3m** (119 MHz, $CDCl_3$, 25 ºC)

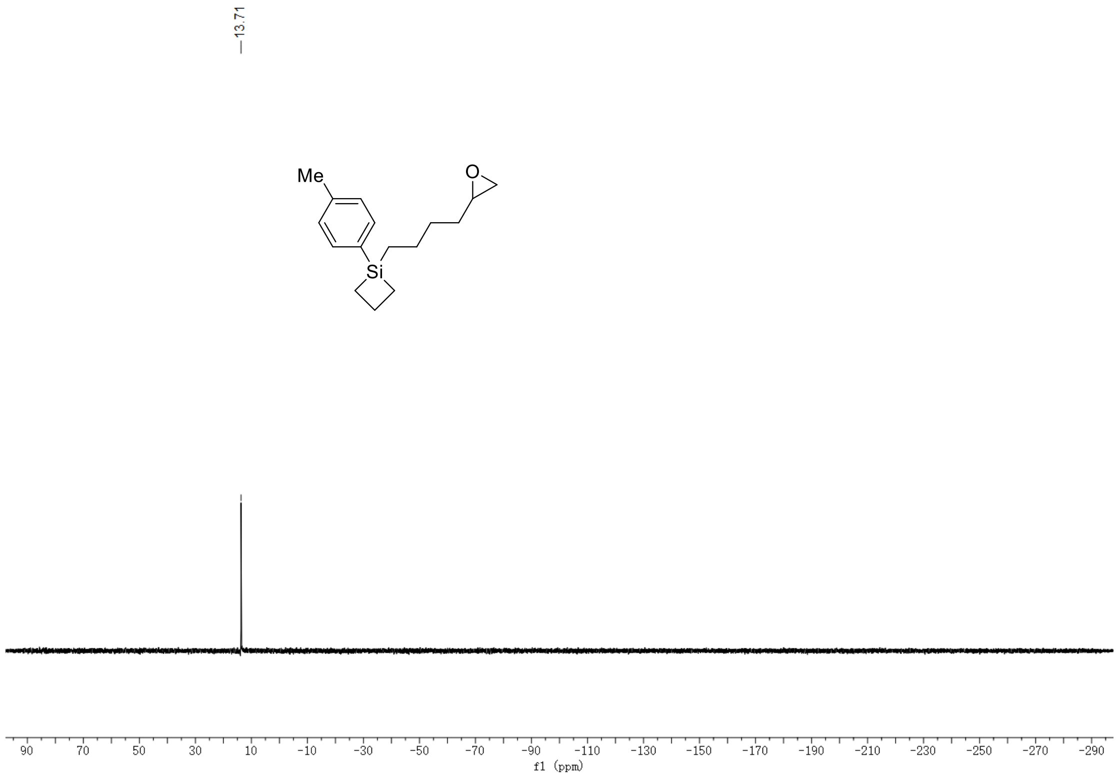

^{1}H NMR of **3n** (600 MHz, $CDCl_3$, 25 ºC)

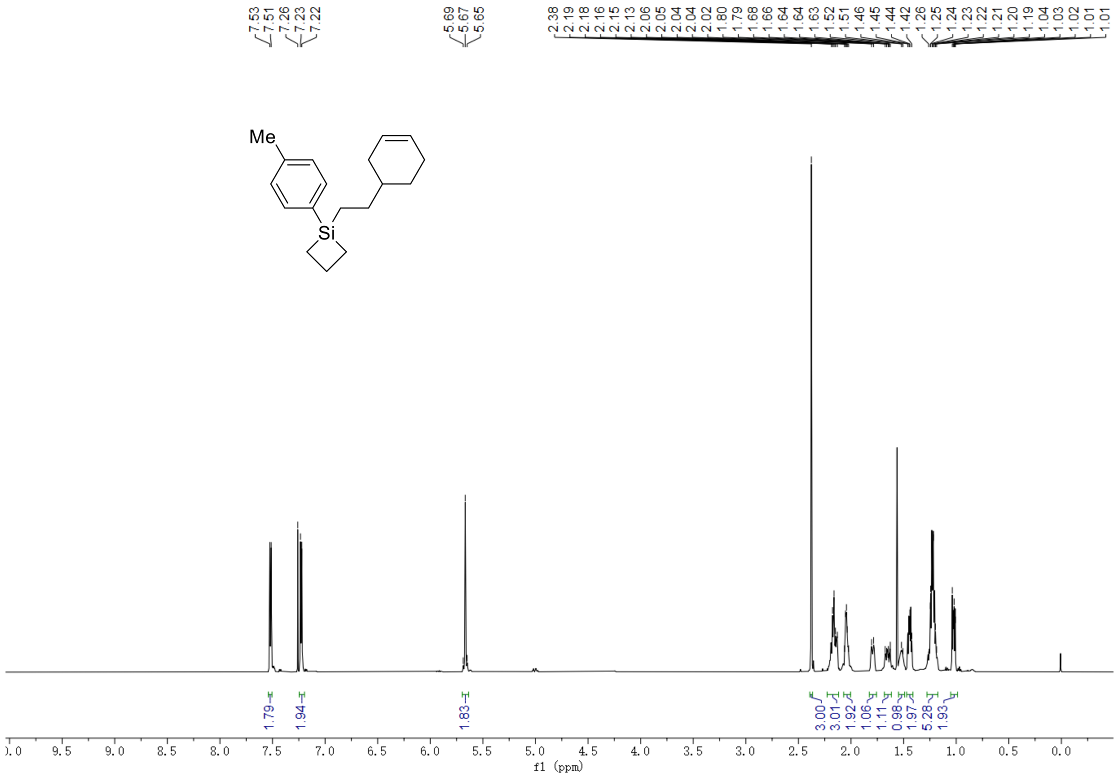

^{13}C NMR of **3n** (151 MHz, $CDCl_3$, 25 ºC)

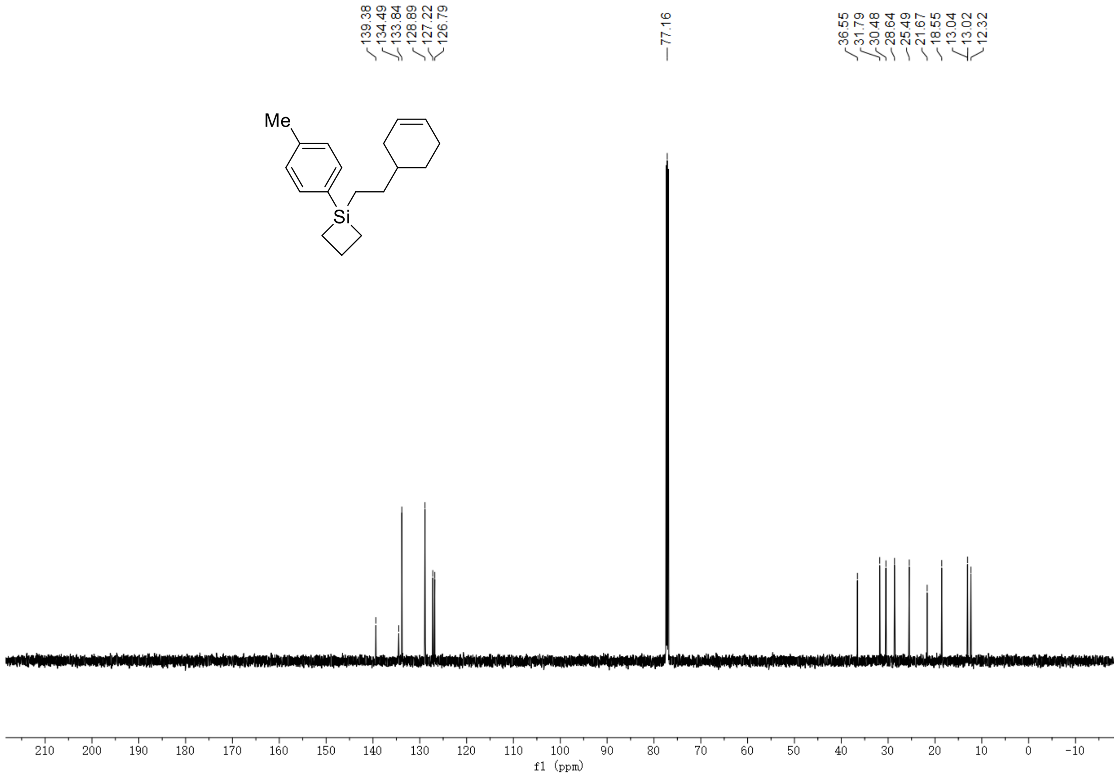

^{29}Si NMR of **3n** (119 MHz, $CDCl_3$, 25 ºC)

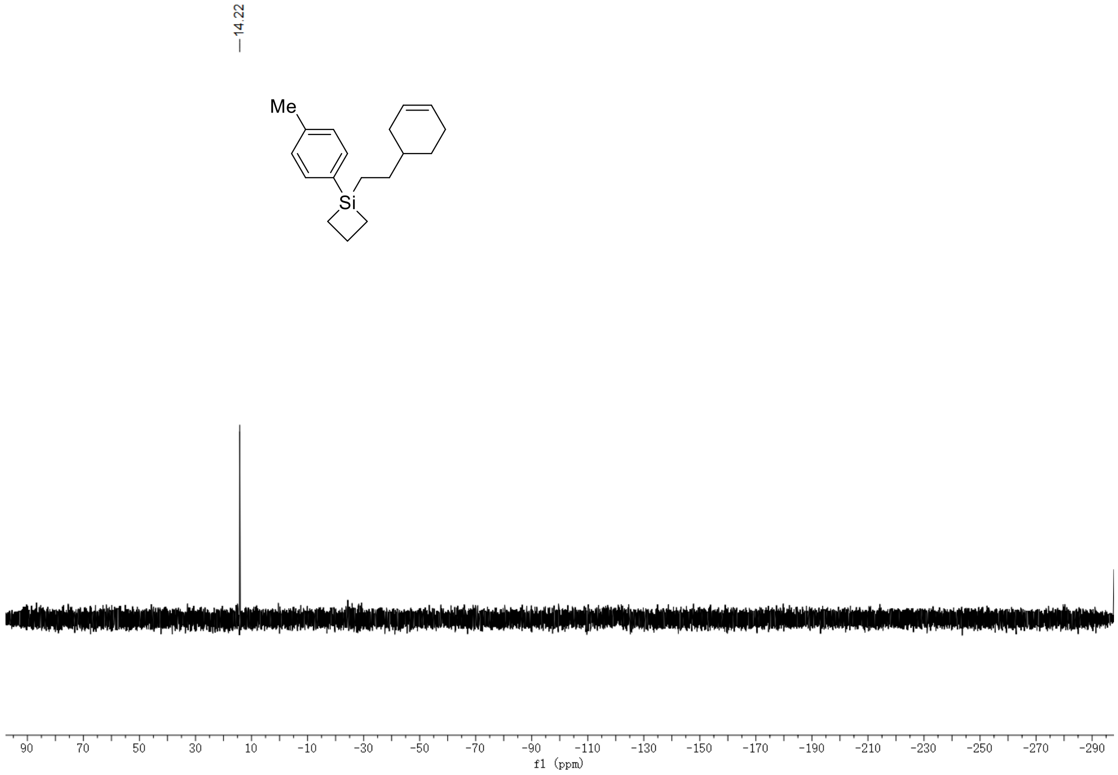

^{1}H NMR of **3o** (400 MHz, $CDCl_3$, 25 ºC)

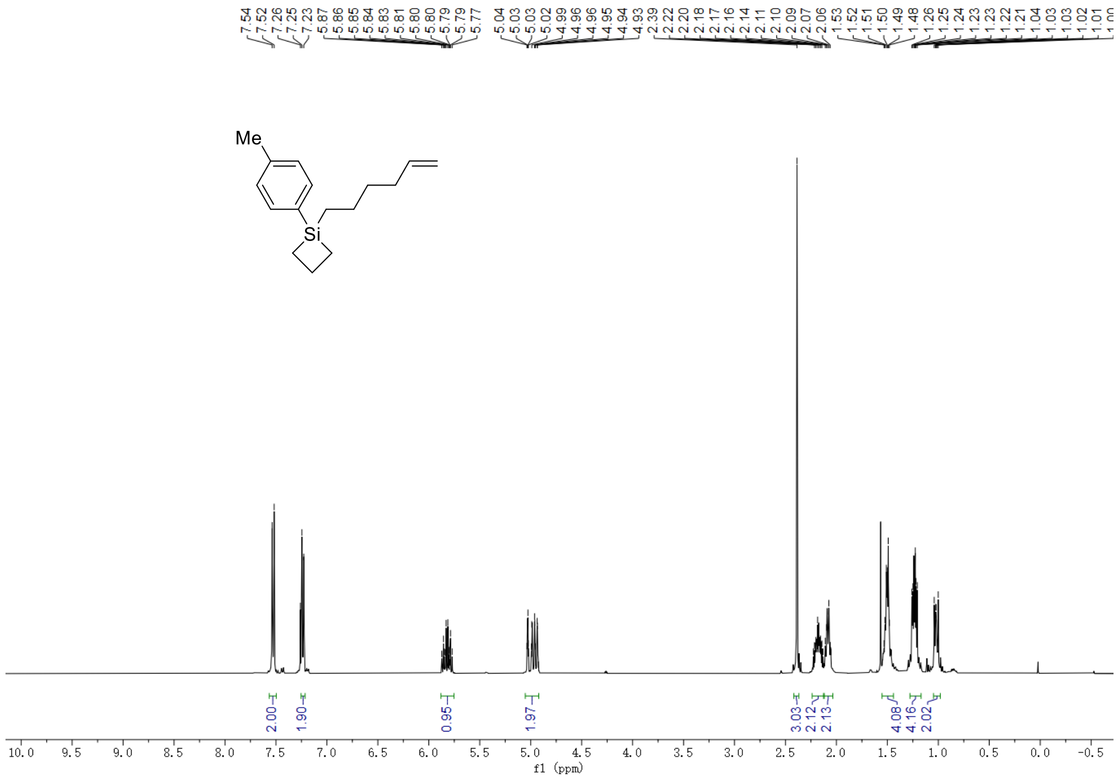

^{13}C NMR of **3o** (101 MHz, $CDCl_3$, 25 ºC)

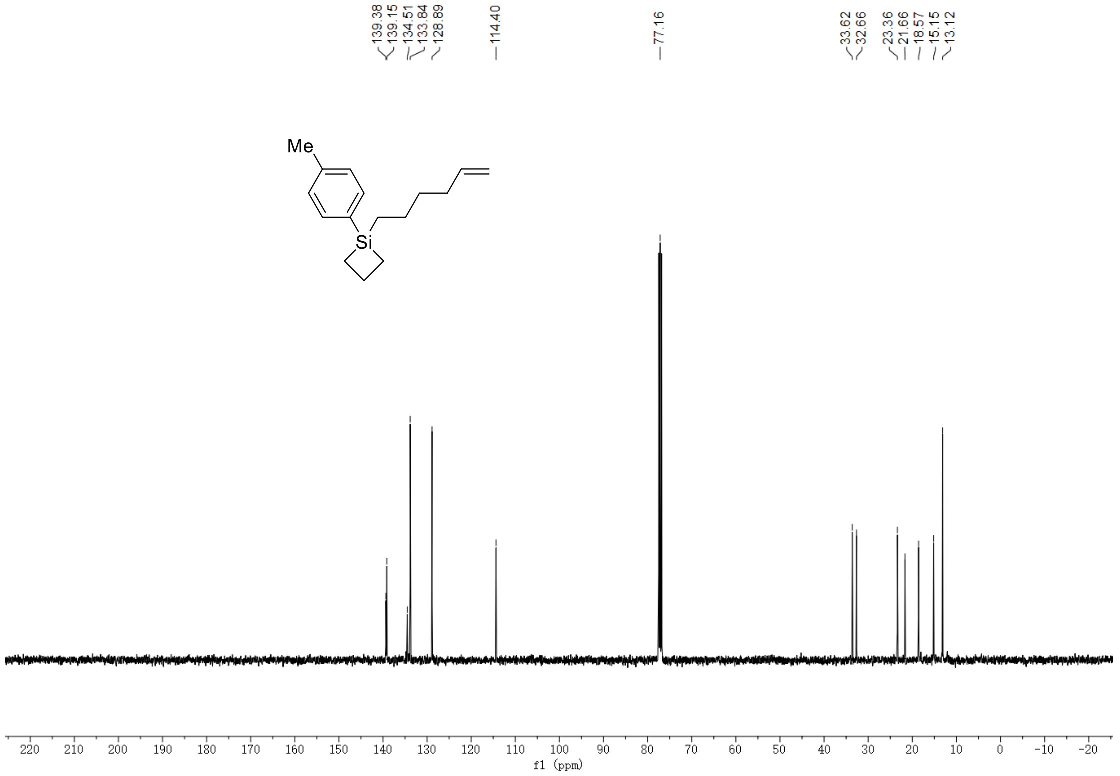

^{29}Si NMR of **3o** (119 MHz, $CDCl_3$, 25 ºC)

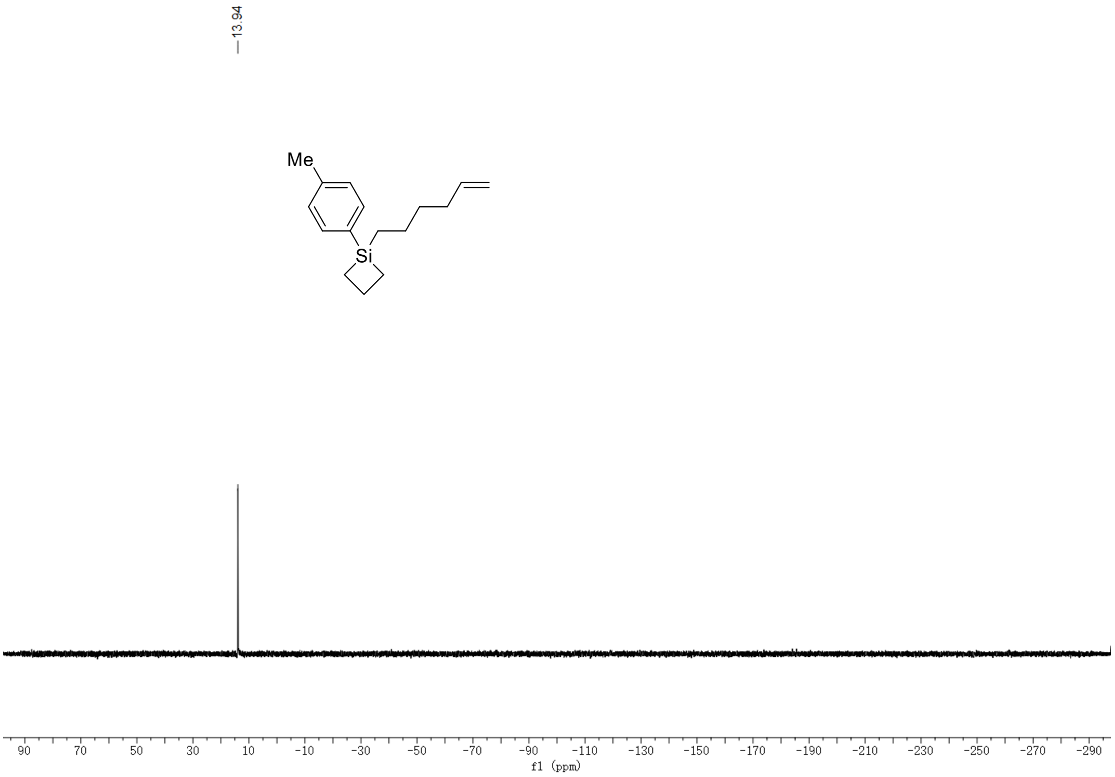

^{1}H NMR of **3p** (400 MHz, $CDCl_3$, 25 ºC)

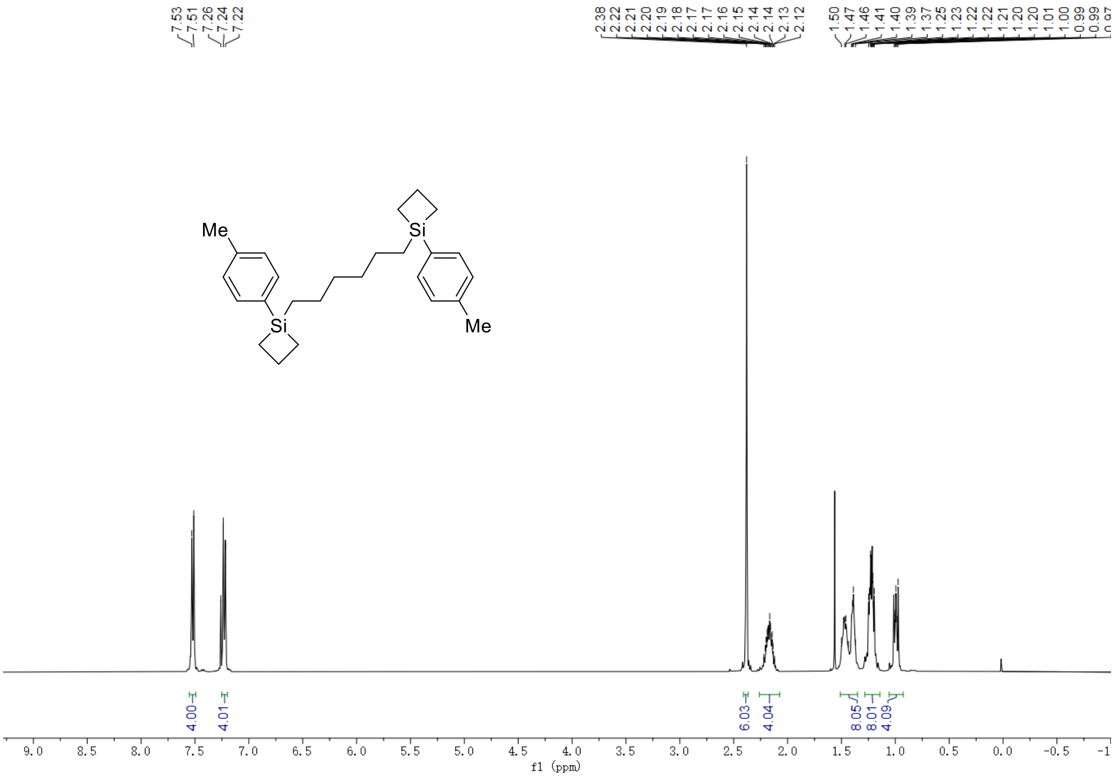

^{13}C NMR of **3p** (101 MHz, $CDCl_3$, 25 ºC)

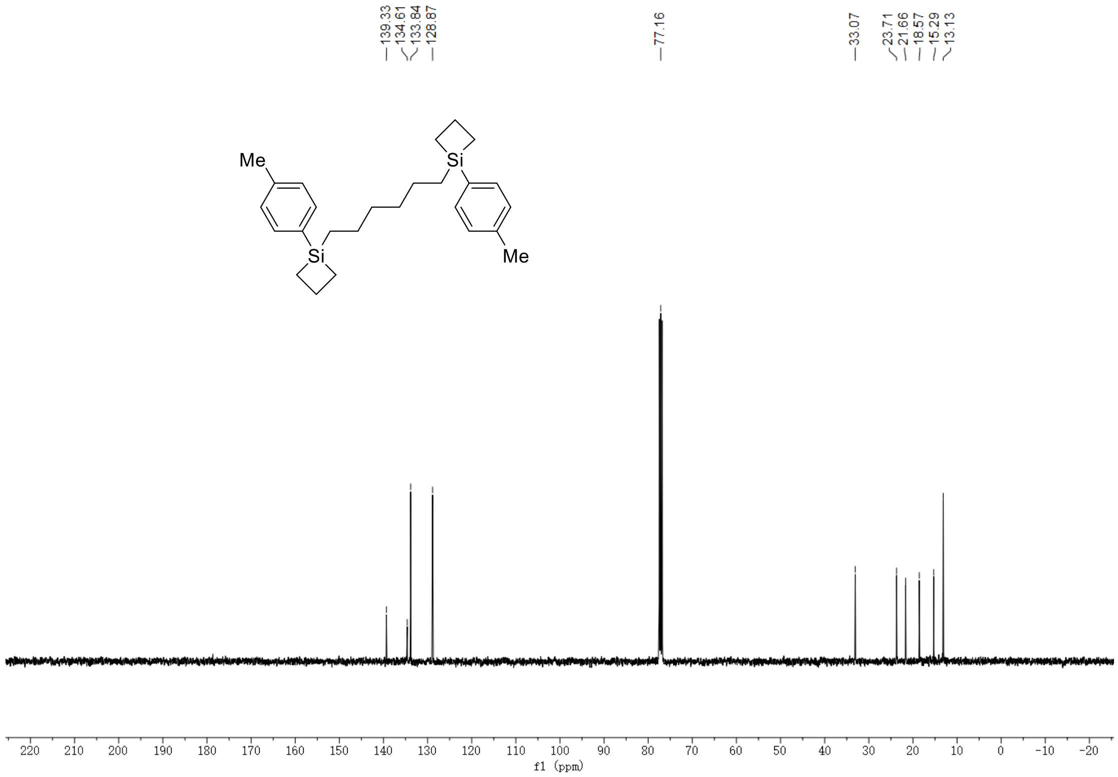

^{29}Si NMR of **3p** (119 MHz, $CDCl_3$, 25 ºC)

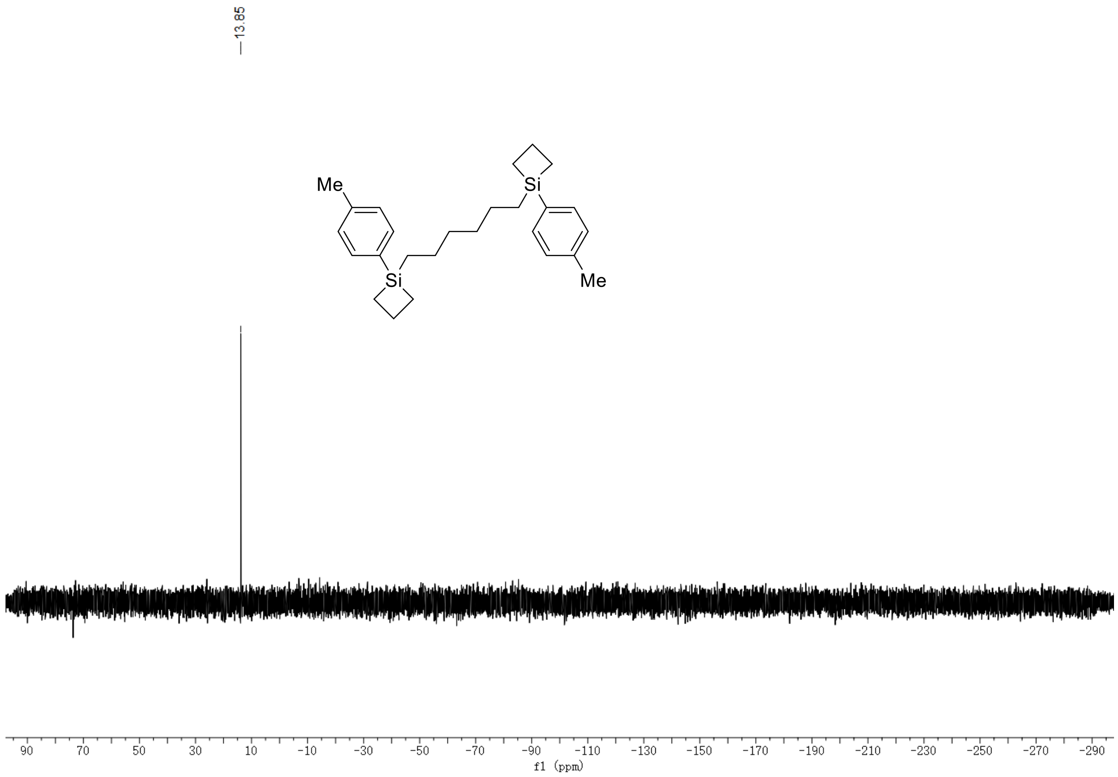

^{1}H NMR of **3q** (400 MHz, $CDCl_3$, 25 ºC)

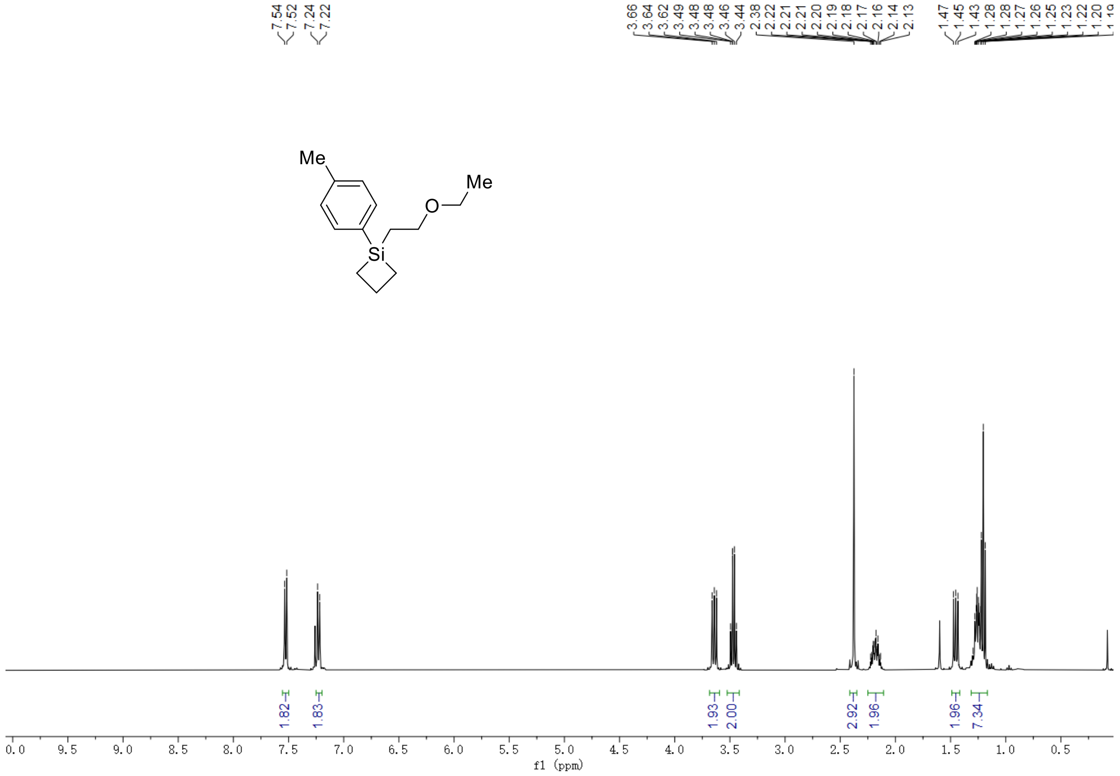

^{13}C NMR of **3q** (101 MHz, $CDCl_3$, 25 ºC)

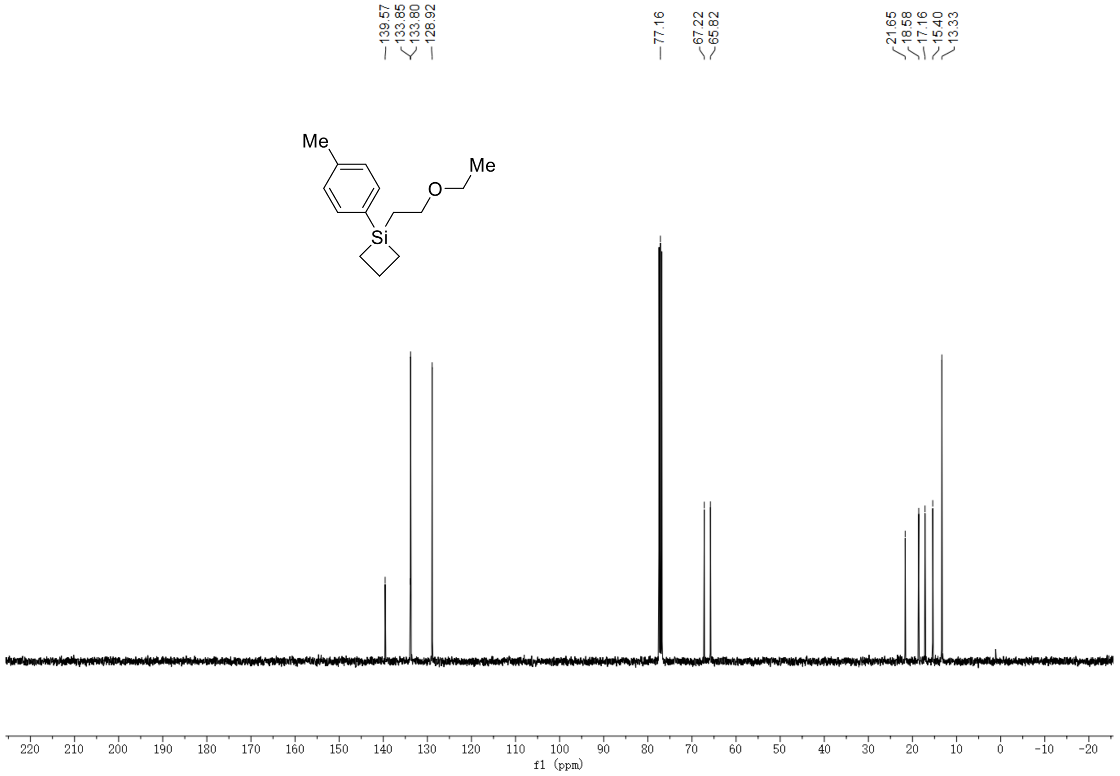

^{29}Si NMR of **3q** (119 MHz, $CDCl_3$, 25 ºC)

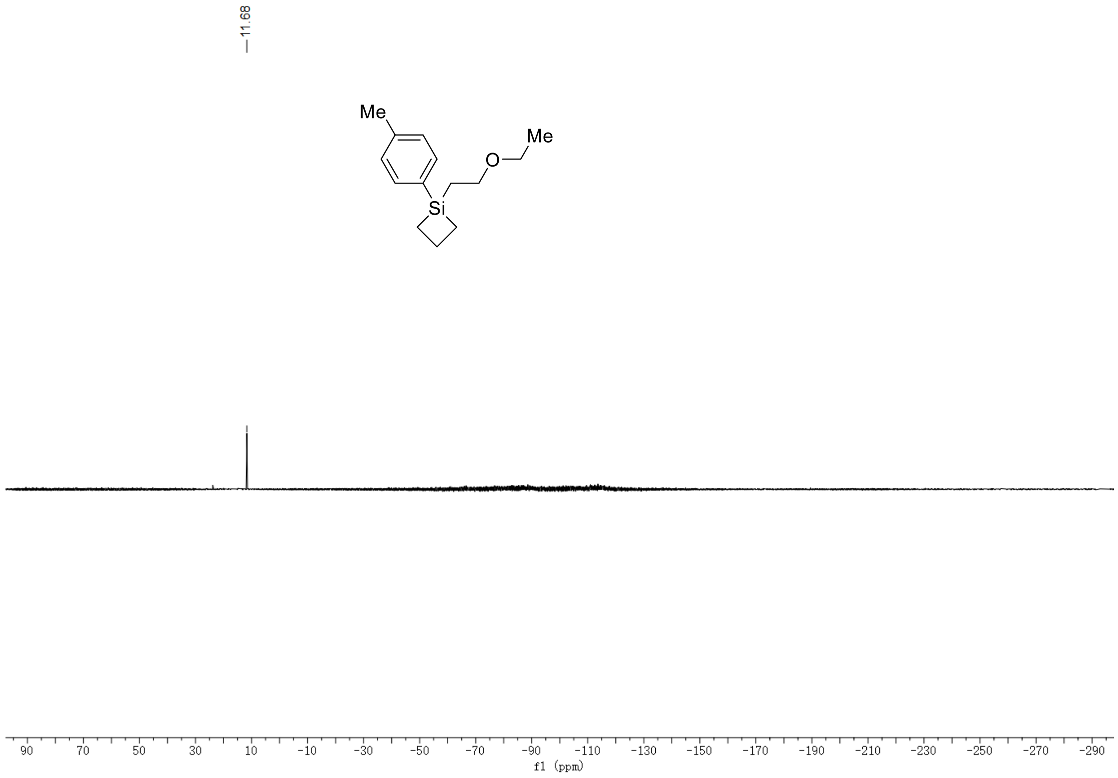

^{1}H NMR of **3r** (400 MHz, $CDCl_3$, 25 ºC)

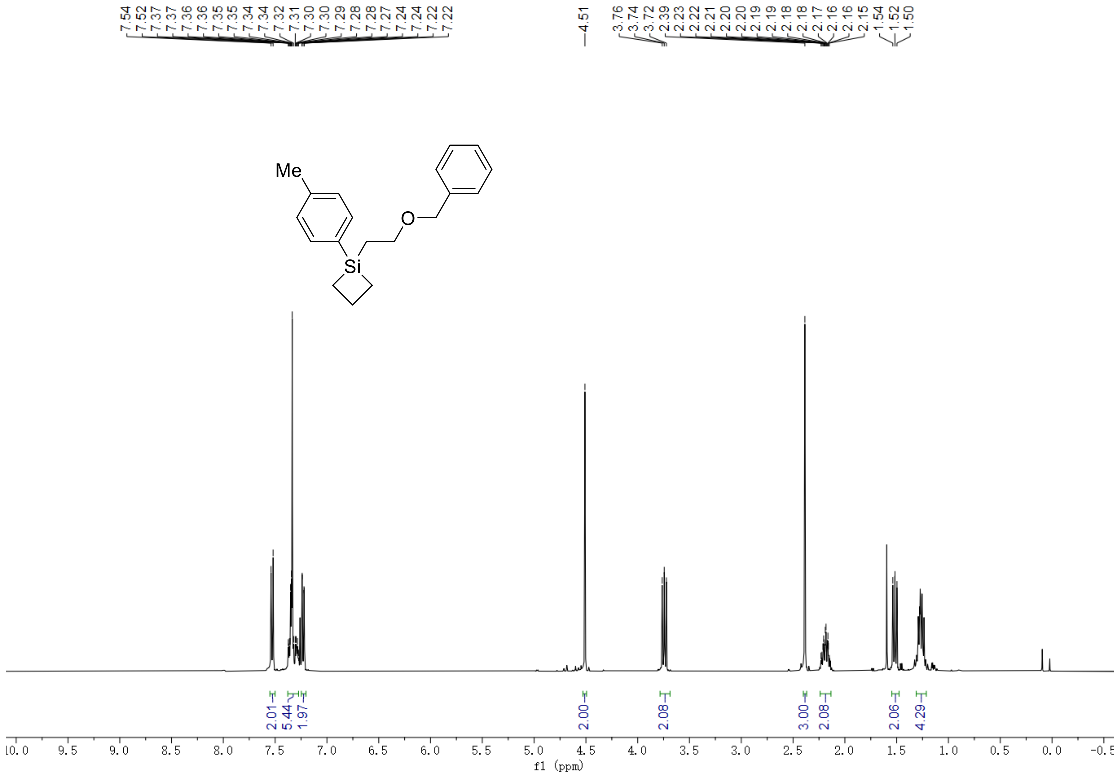

^{13}C NMR of **3r** (151 MHz, $CDCl_3$, 25 ºC)

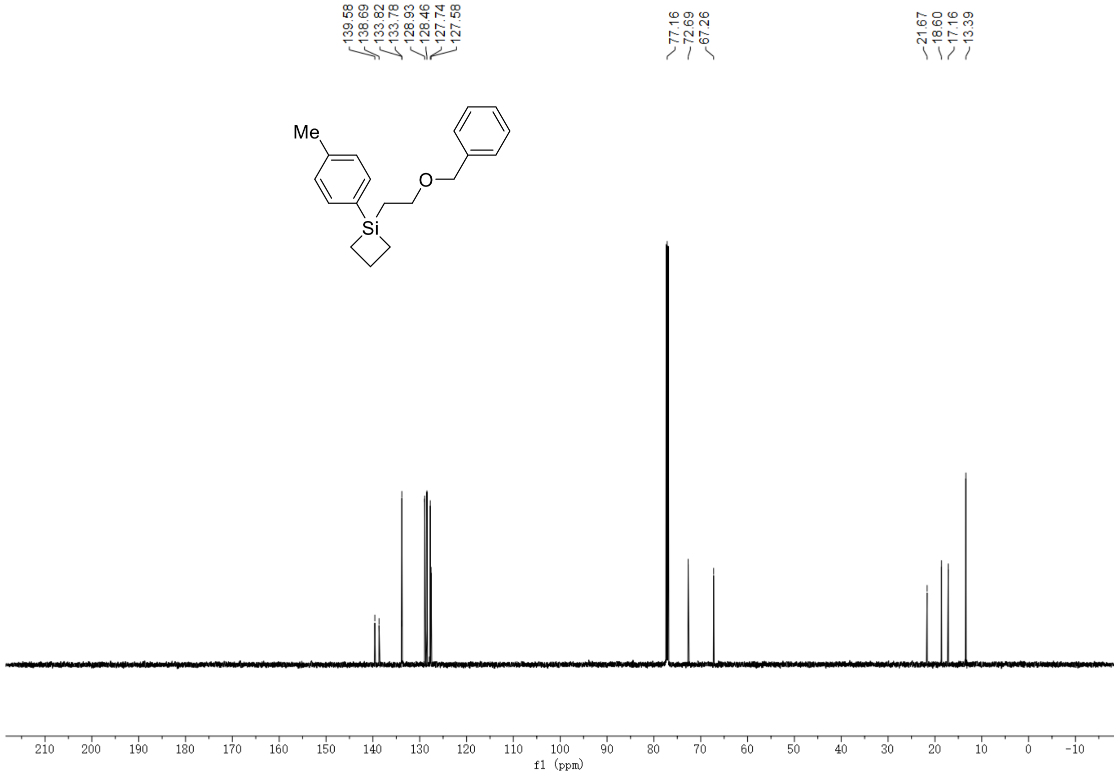

^{29}Si NMR of **3r** (119 MHz, $CDCl_3$, 25 ºC)

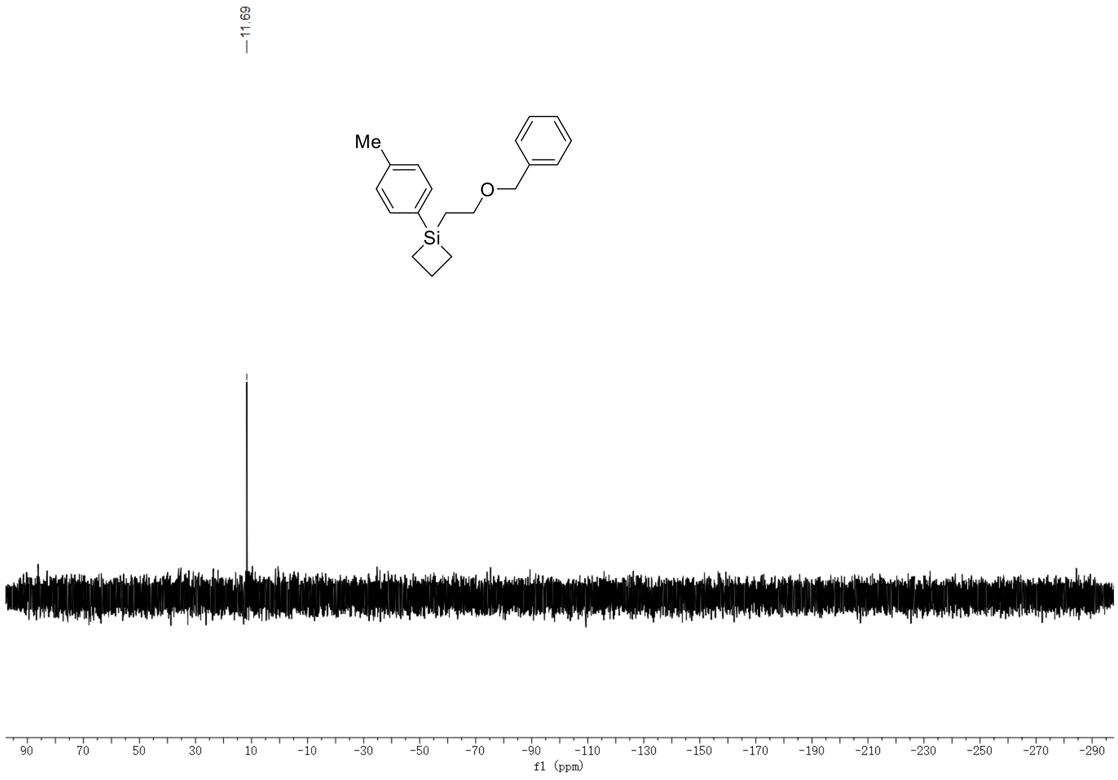

^{1}H NMR of **3s** (400 MHz, $CDCl_3$, 25 ºC)

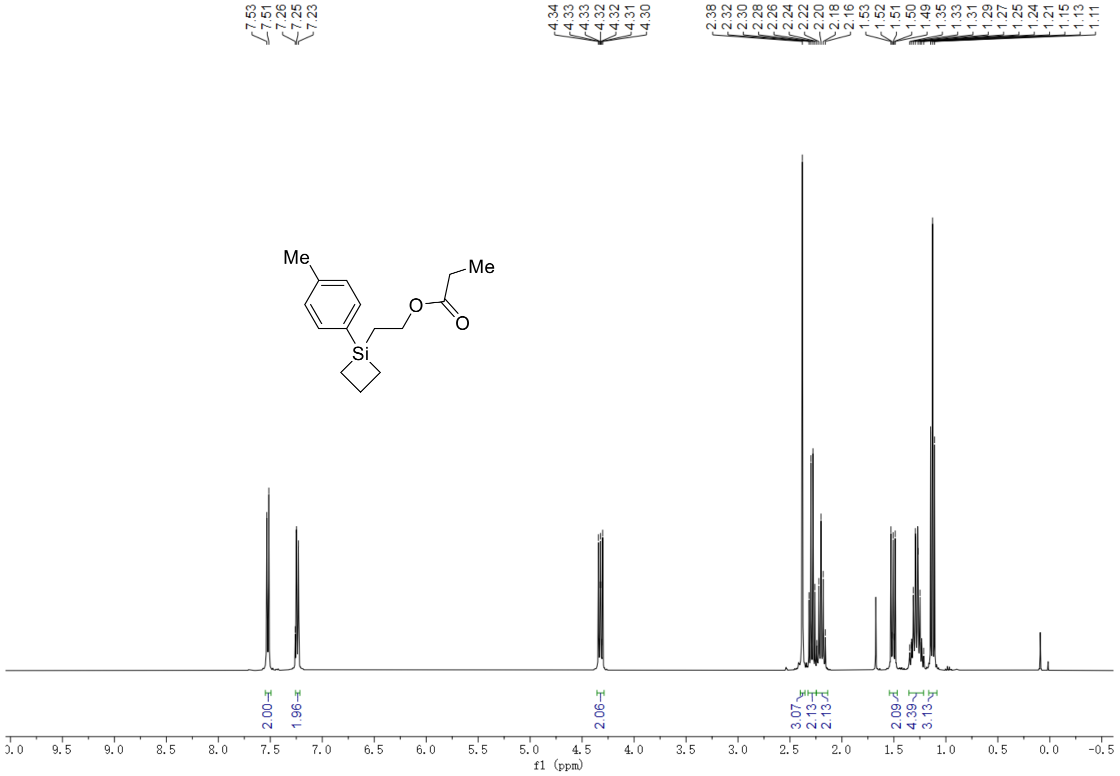

^{13}C NMR of **3s** (151 MHz, $CDCl_3$, 25 ºC)

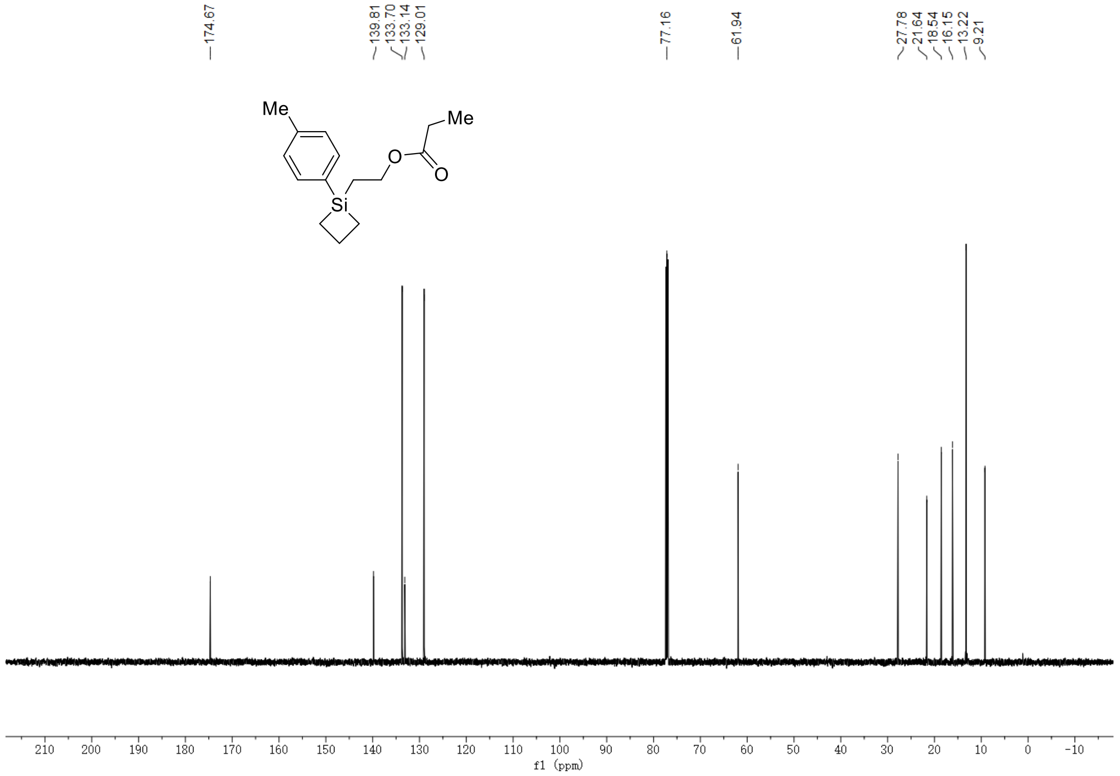

^{29}Si NMR of **3s** (119 MHz, $CDCl_3$, 25 °C)

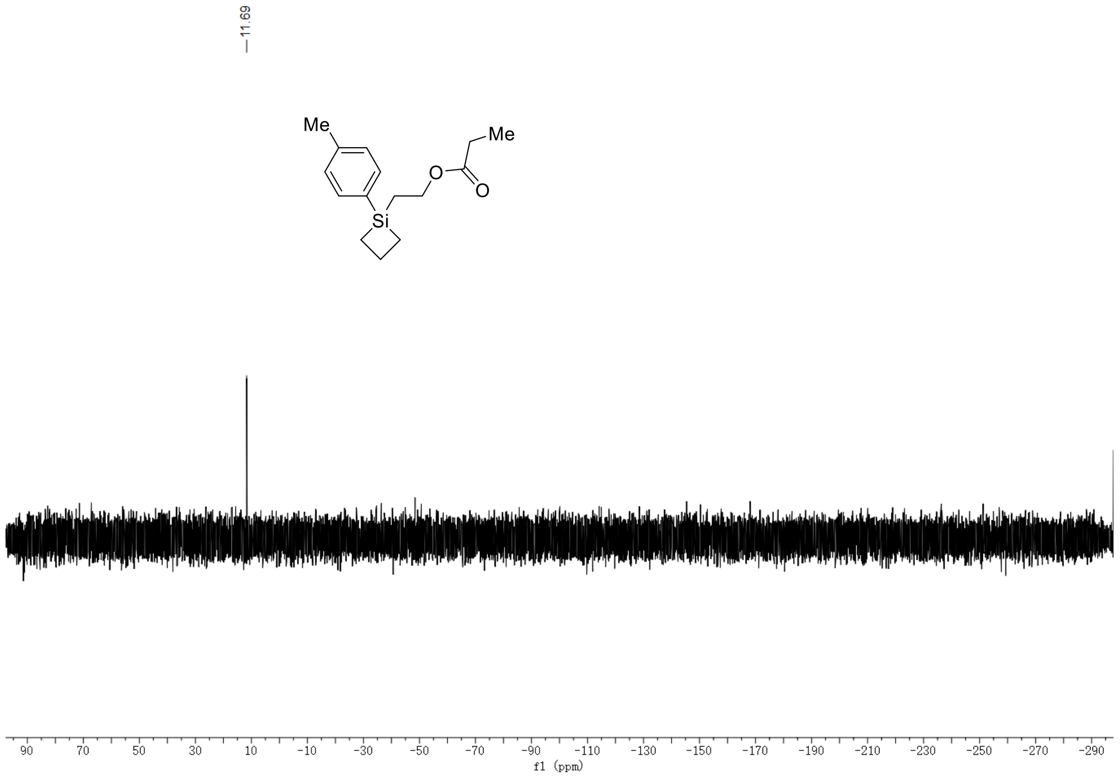

^{1}H NMR of **3t** (400 MHz, $CDCl_3$, 25 °C)

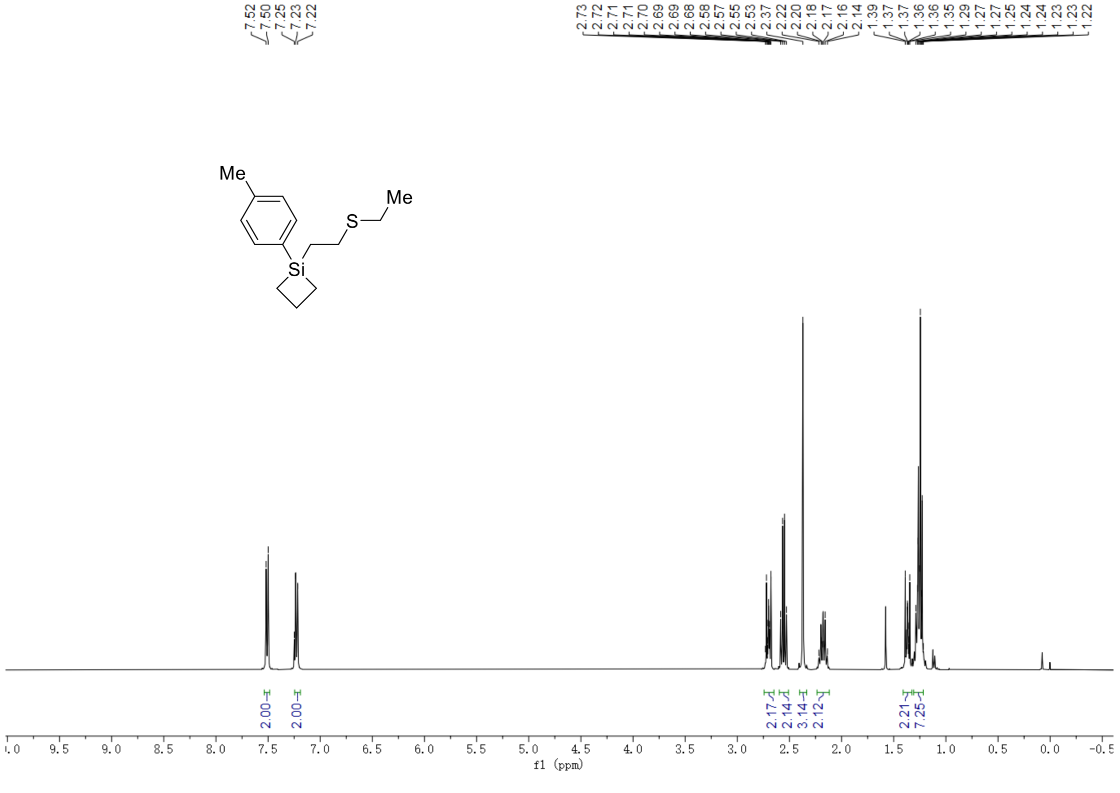

^{13}C NMR of **3t** (151 MHz, $CDCl_3$, 25 ºC)

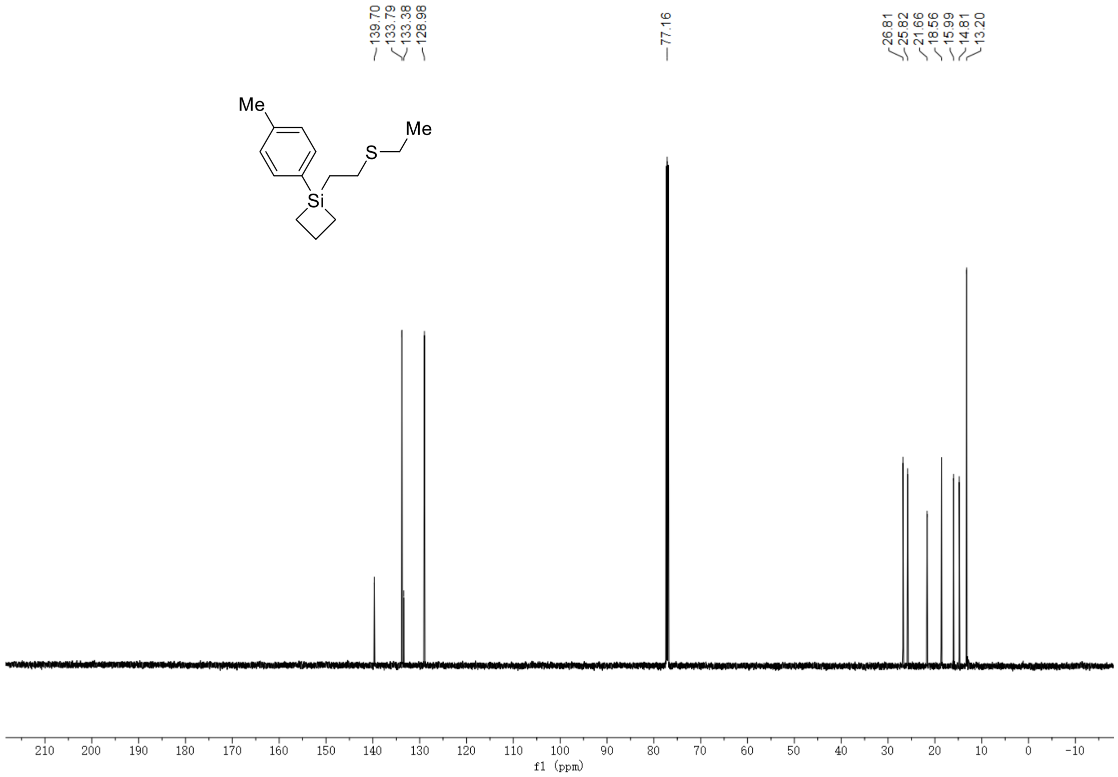

^{29}Si NMR of **3t** (119 MHz, $CDCl_3$, 25 ºC)

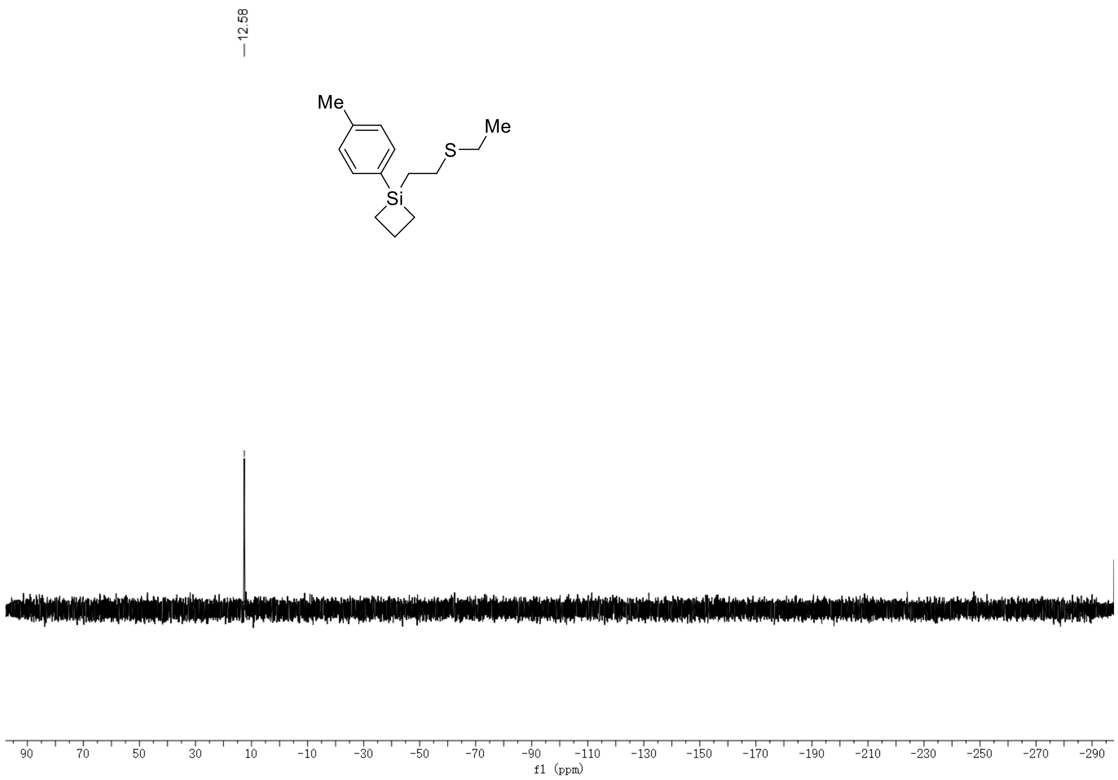

^{1}H NMR of **3u** (400 MHz, $CDCl_3$, 25 ºC)

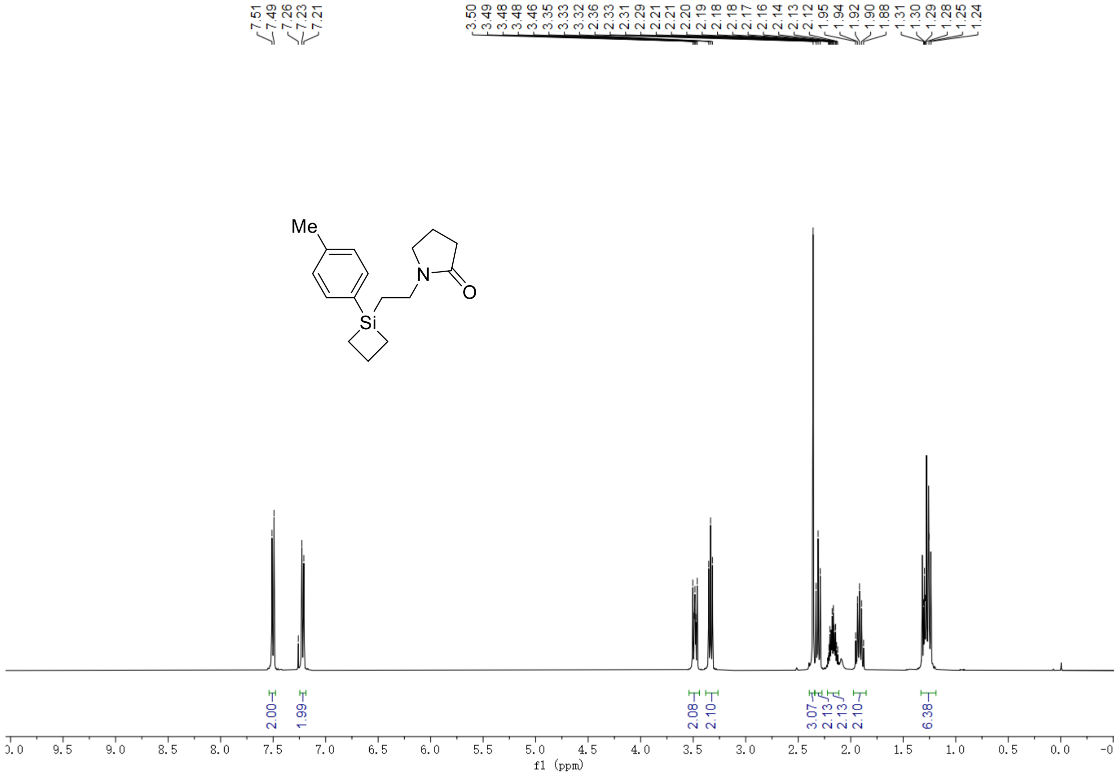

^{13}C NMR of **3u** (151 MHz, $CDCl_3$, 25 ºC)

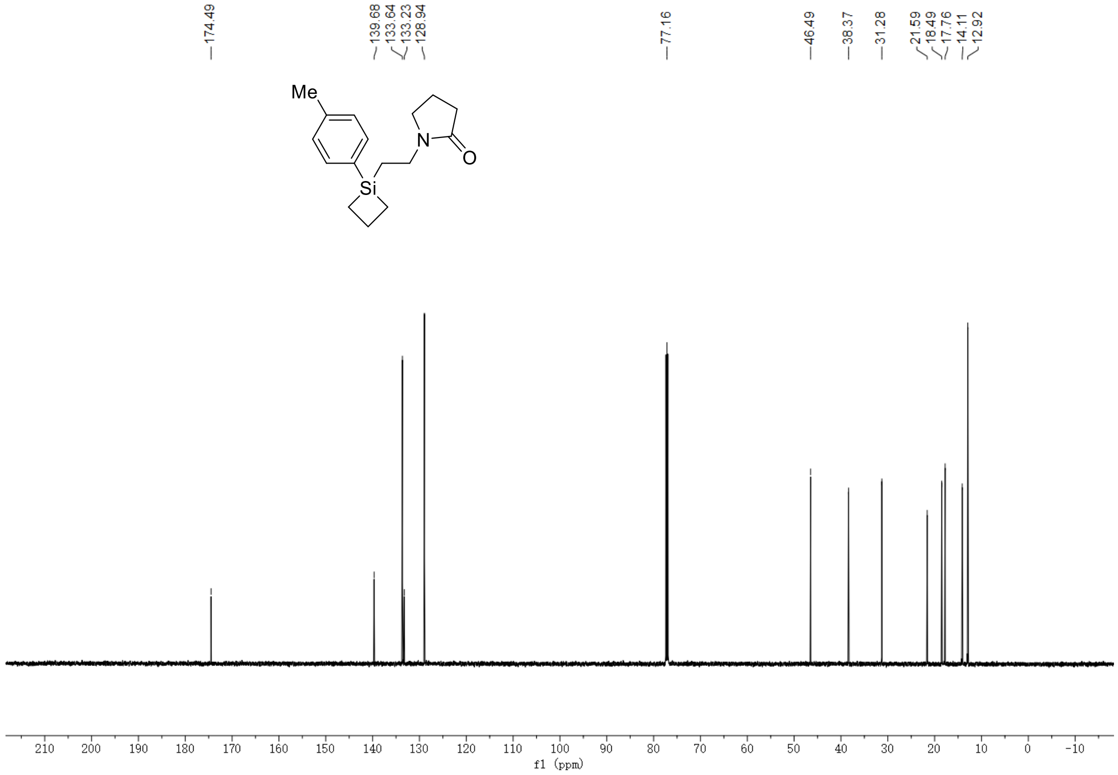

^{29}Si NMR of **3u** (119 MHz, $CDCl_3$, 25 ºC)

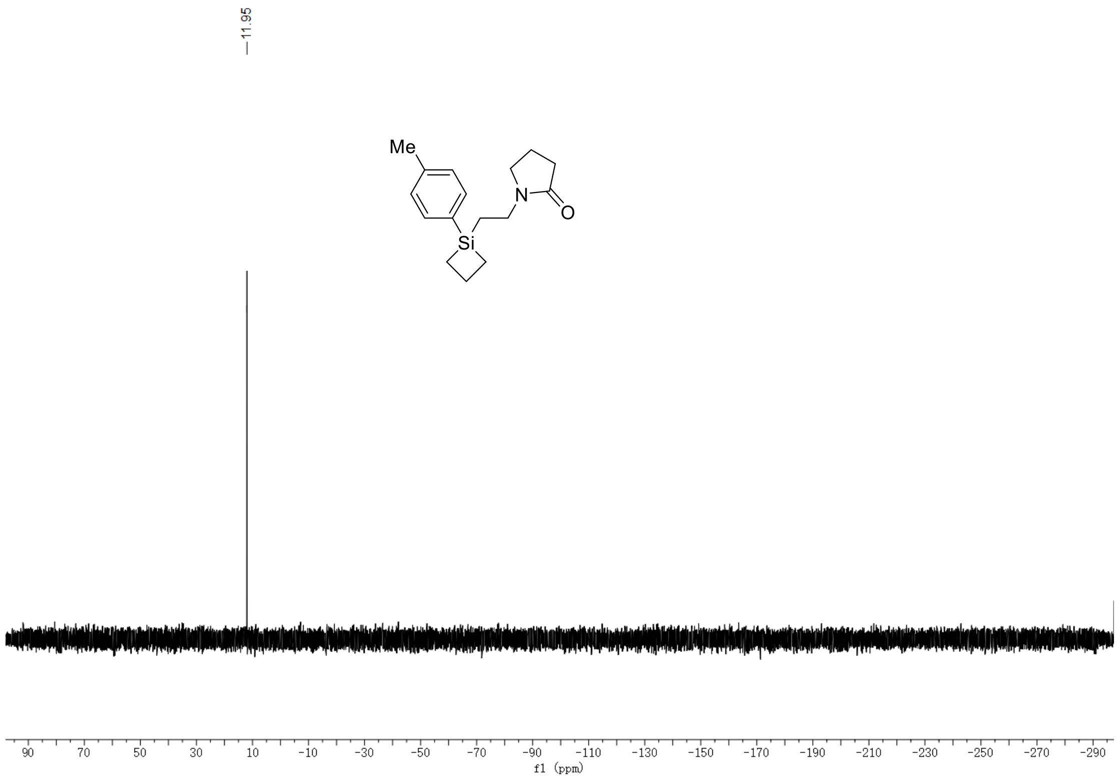

^{1}H NMR of **3v** (400 MHz, $CDCl_3$, 25 ºC)

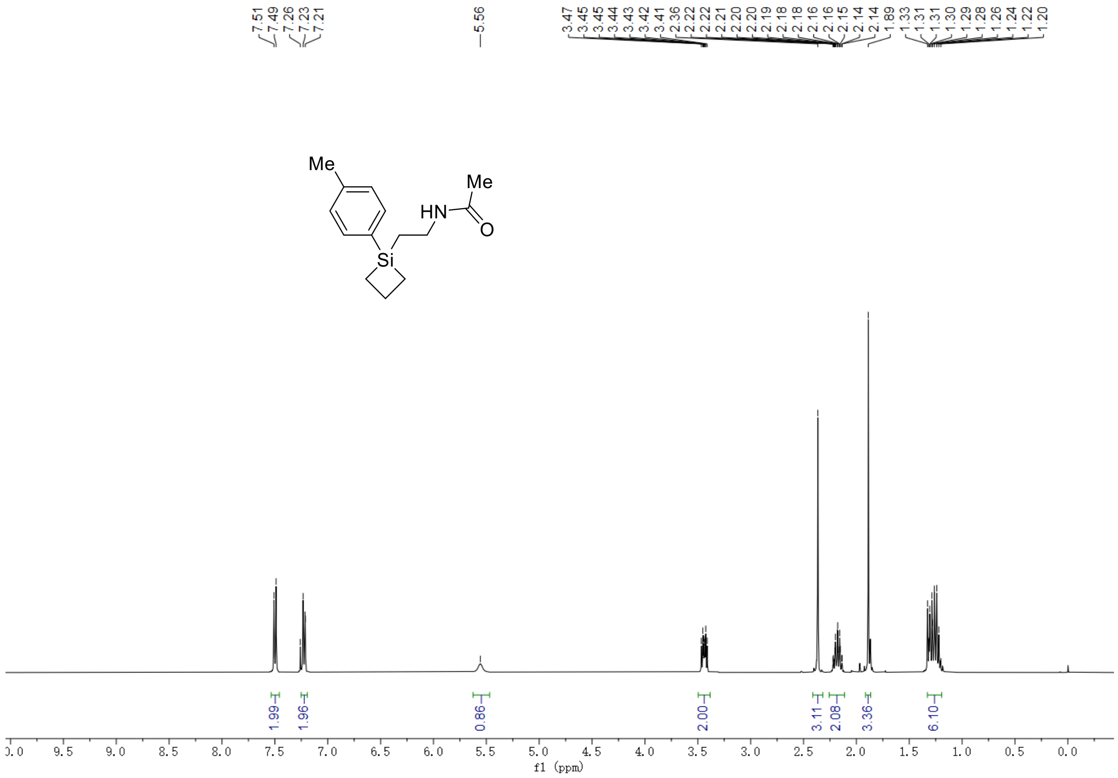

^{13}C NMR of **3v** (151 MHz, $CDCl_3$, 25 ºC)

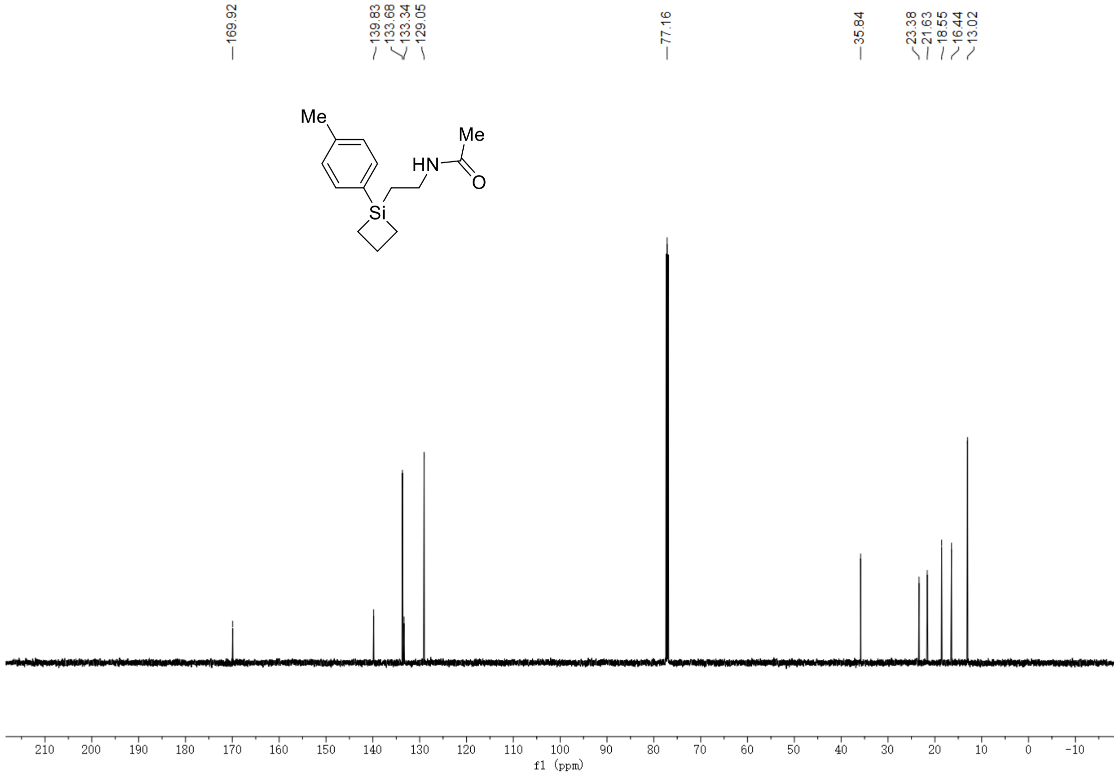

^{29}Si NMR of **3v** (119 MHz, $CDCl_3$, 25 ºC)

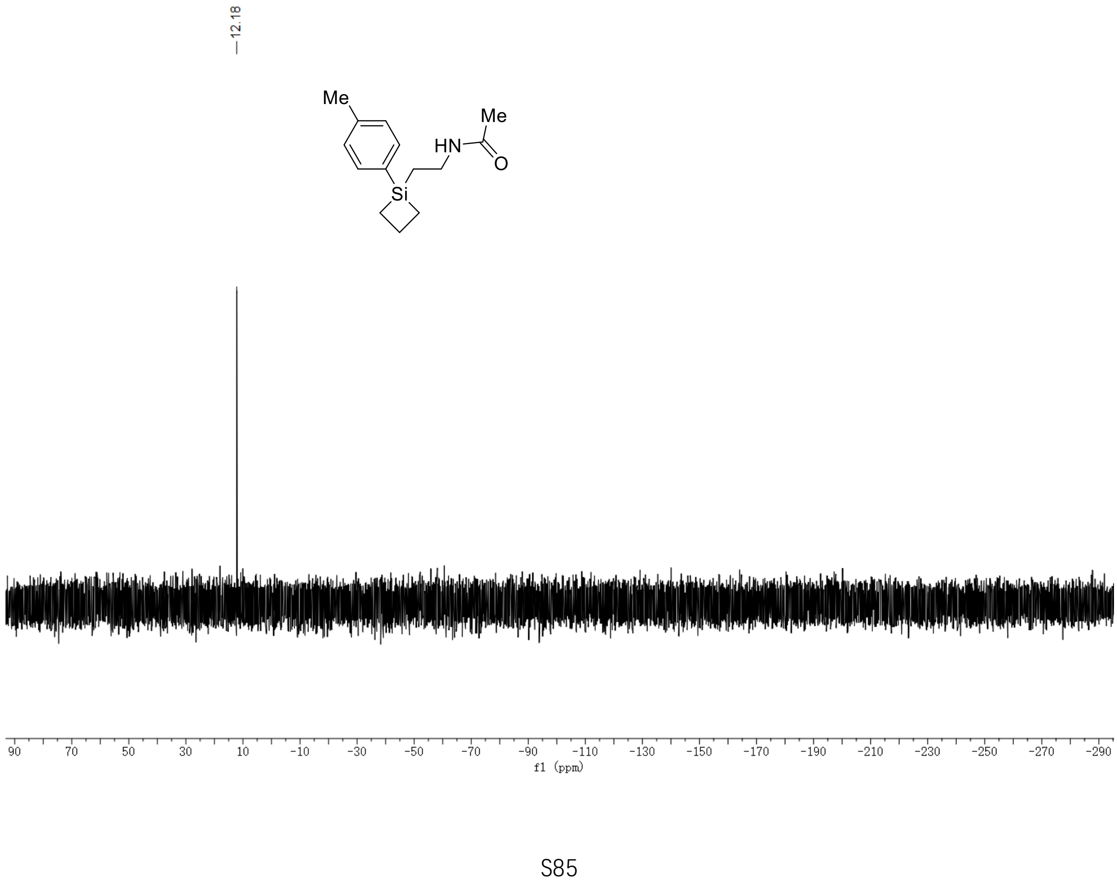

^{1}H NMR of **3w** (400 MHz, $CDCl_3$, 25 ºC)

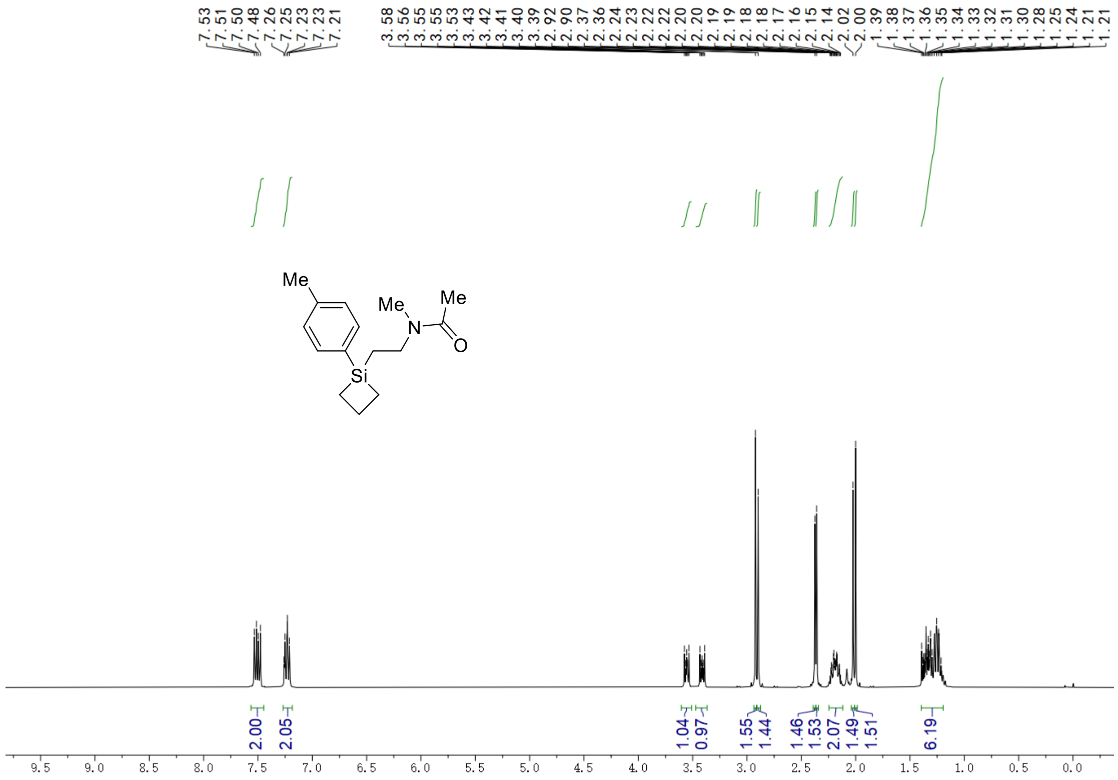

^{13}C NMR of **3w** (151 MHz, $CDCl_3$, 25 ºC)

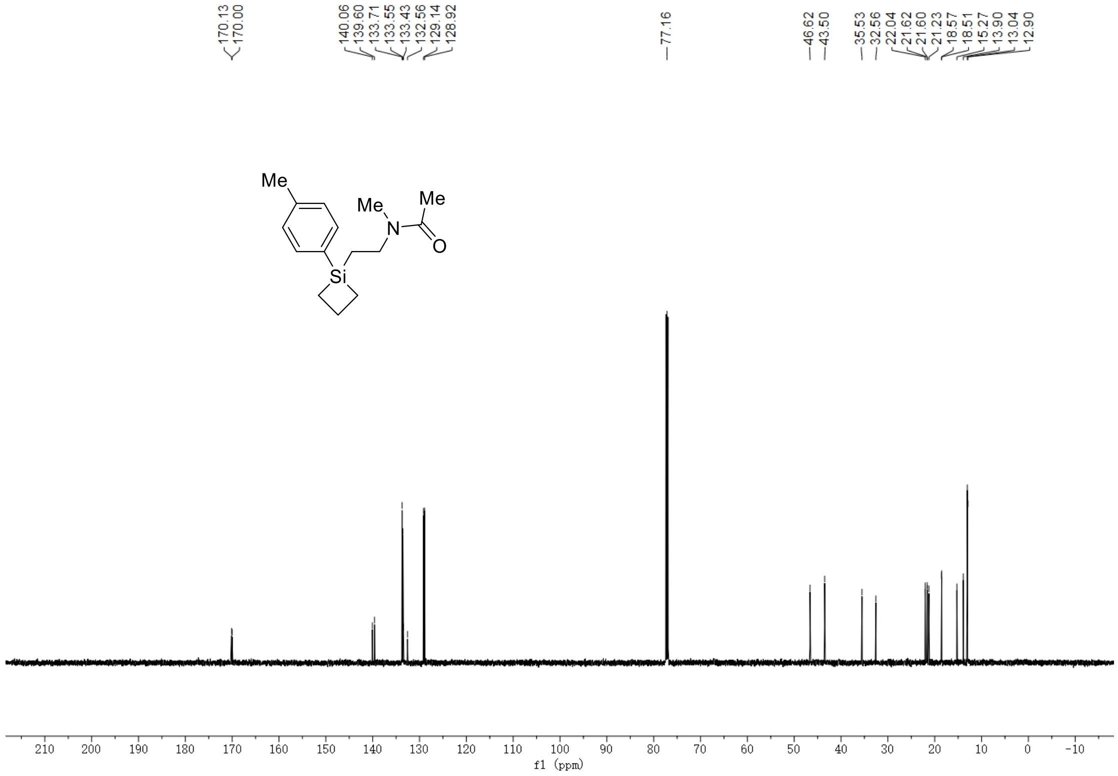

^{29}Si NMR of **3w** (119 MHz, $CDCl_3$, 25 ºC)

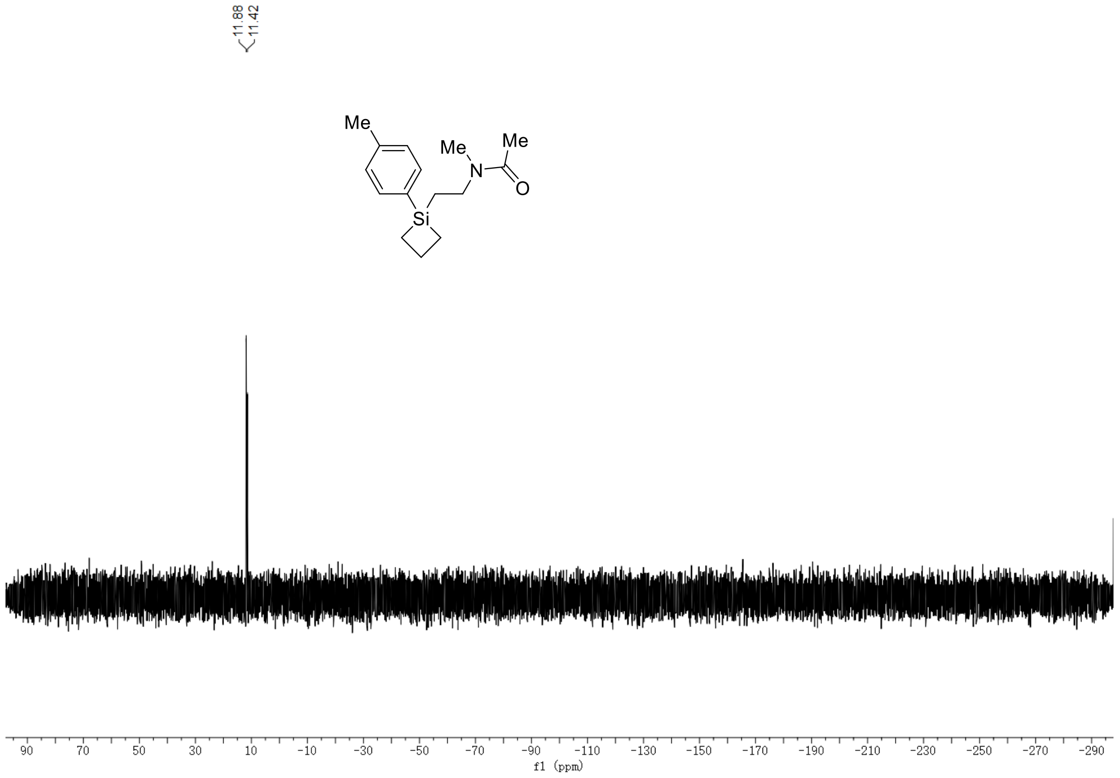

^{1}H NMR of **3x** (400 MHz, $CDCl_3$, 25 ºC)

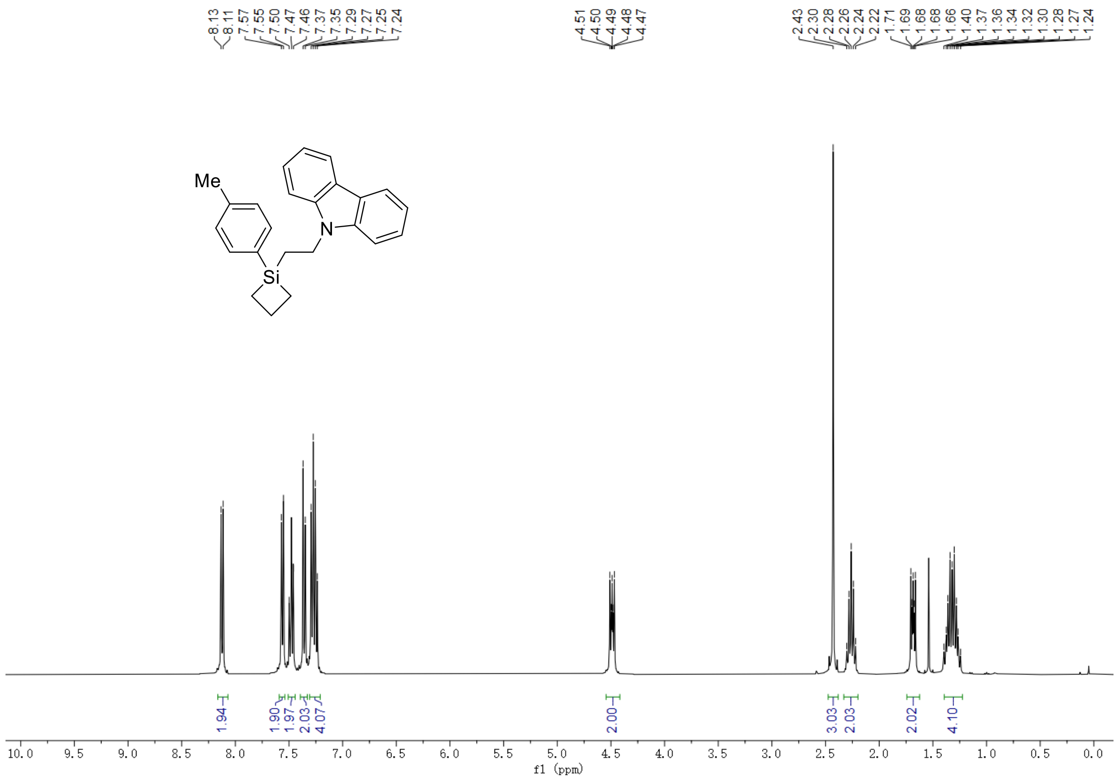

^{13}C NMR of **3x** (151 MHz, $CDCl_3$, 25 ºC)

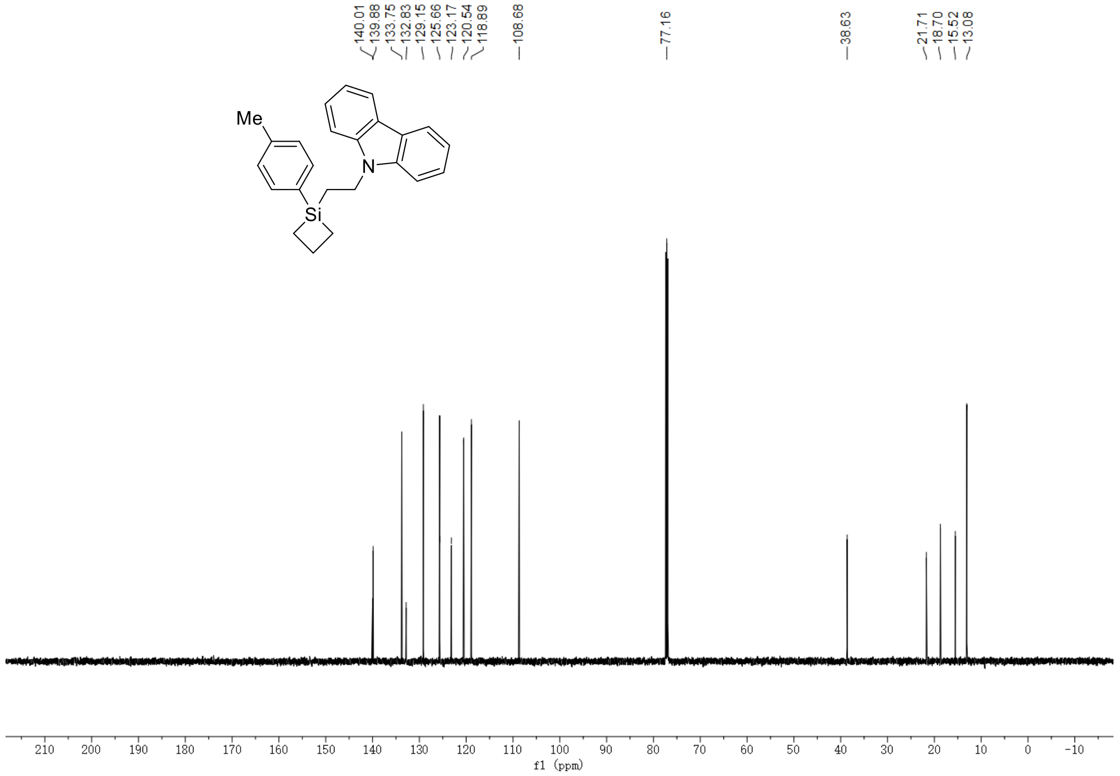

^{29}Si NMR of **3x** (119 MHz, $CDCl_3$, 25 ºC)

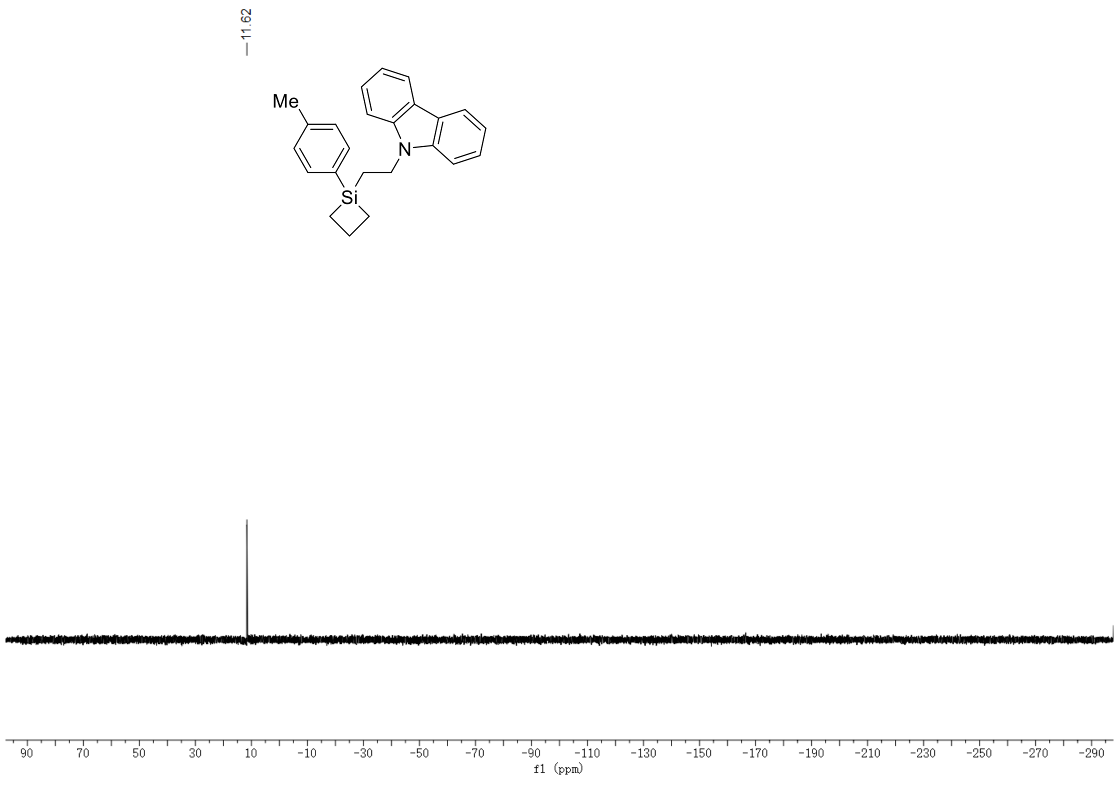

^{1}H NMR of **3y** (400 MHz, $CDCl_3$, 25 ºC)

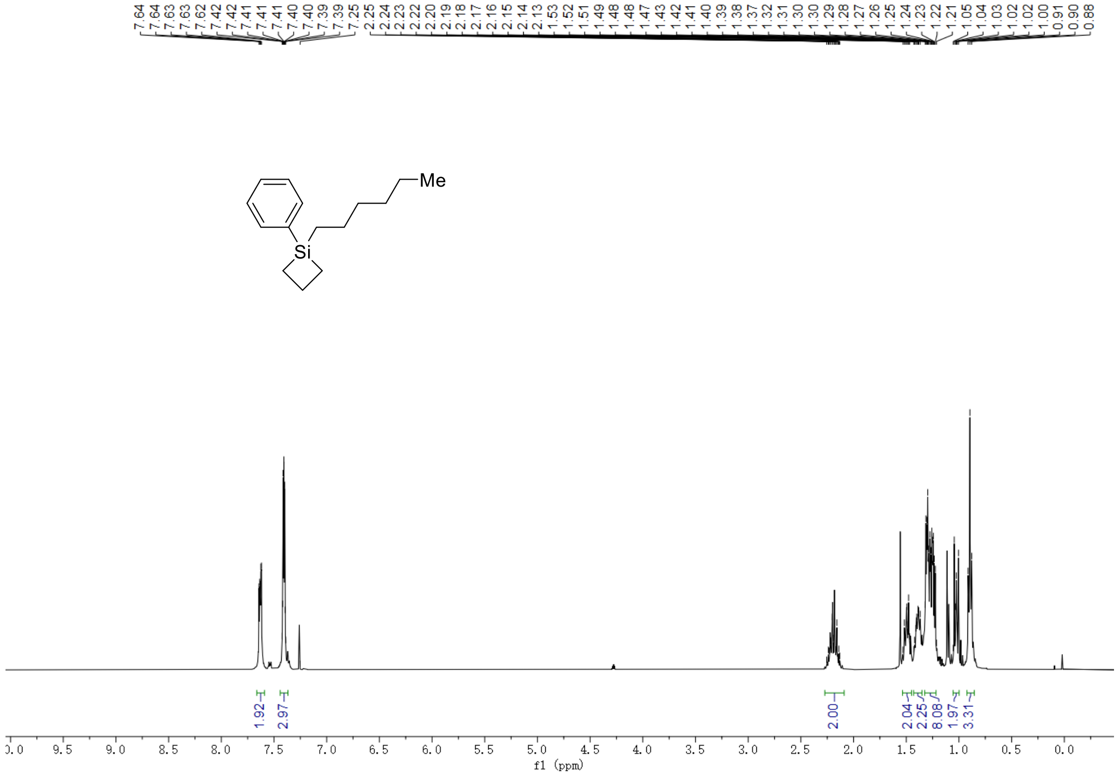

^{13}C NMR of **3y** (151 MHz, $CDCl_3$, 25 ºC)

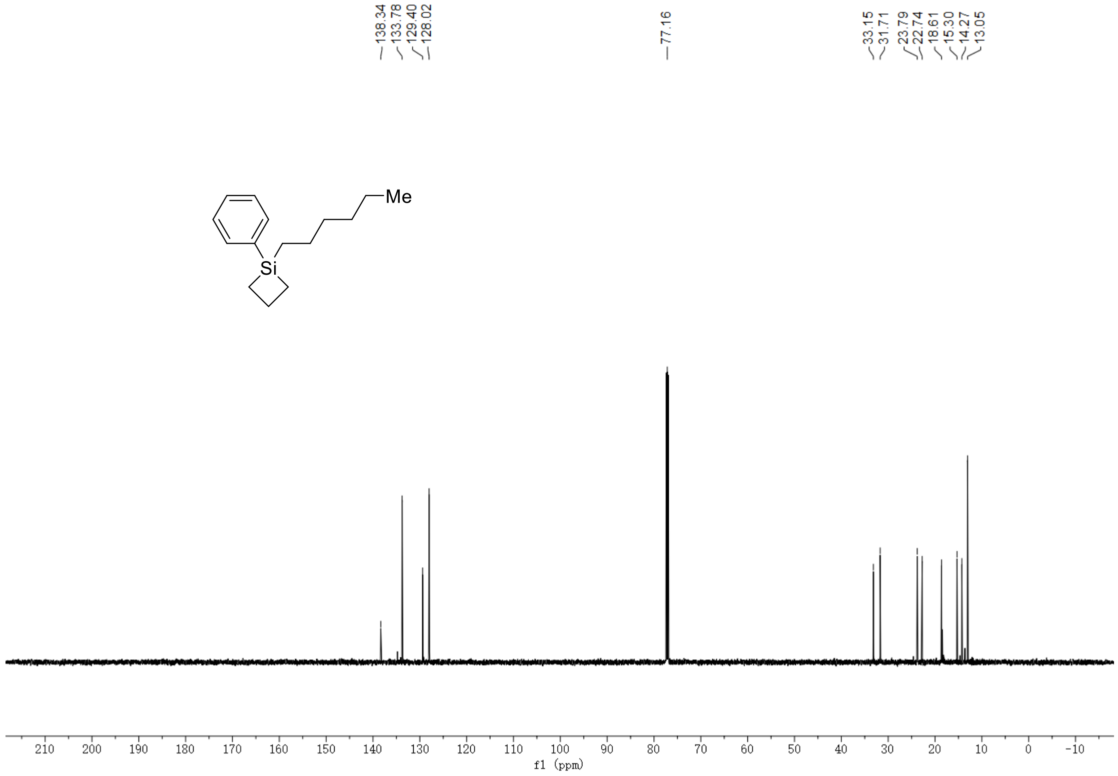

^{29}Si NMR of **3y** (119 MHz, $CDCl_3$, 25 ºC)

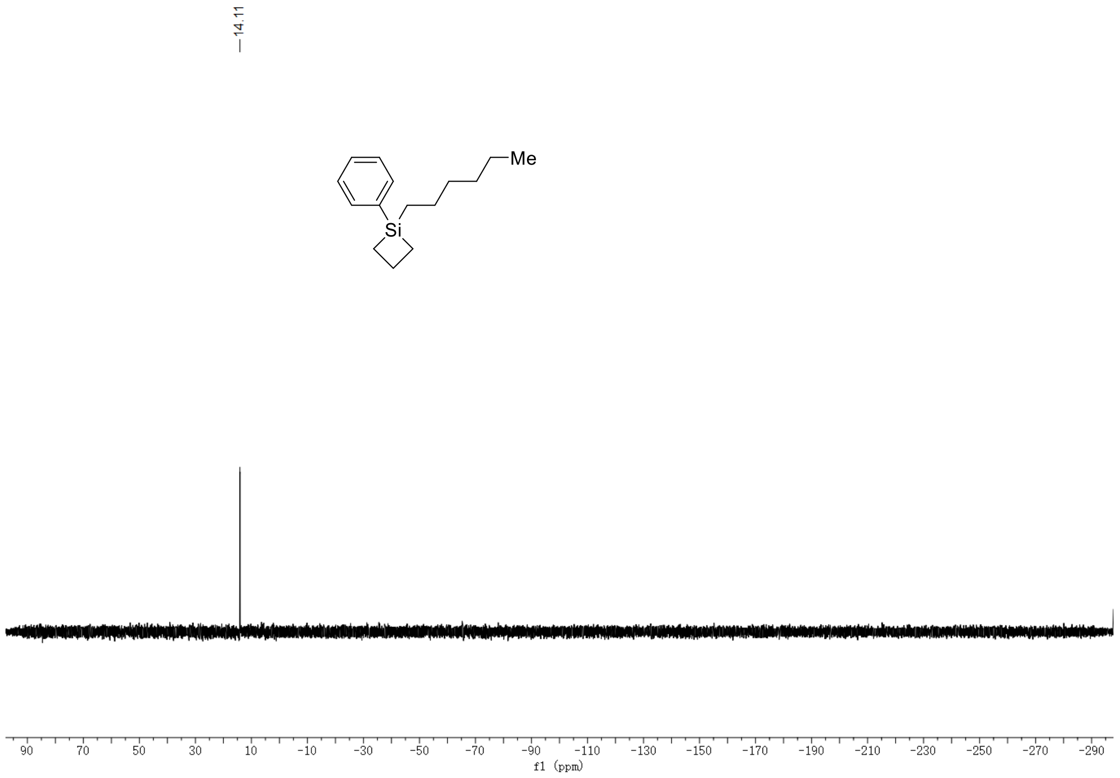

^{1}H NMR of **3z** (400 MHz, $CDCl_3$, 25 ºC)

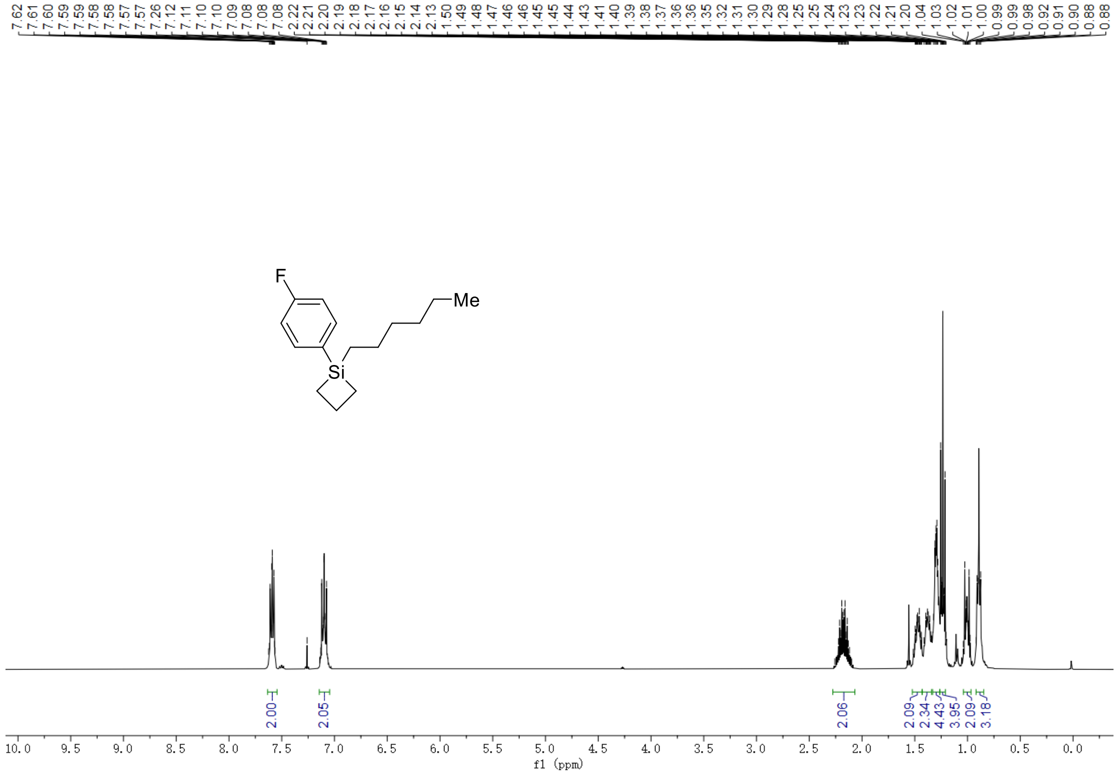

^{13}C NMR of **3z** (101 MHz, $CDCl_3$, 25 ºC)

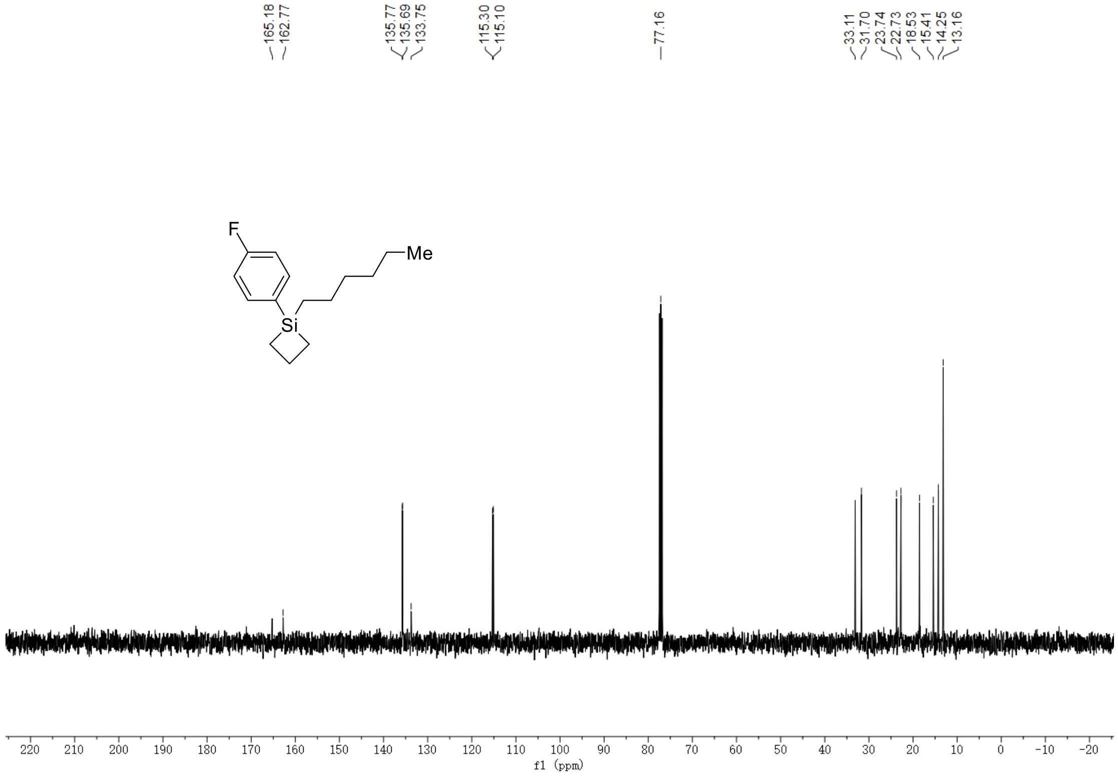

^{29}Si NMR of **3z** (119 MHz, $CDCl_3$, 25 ºC)

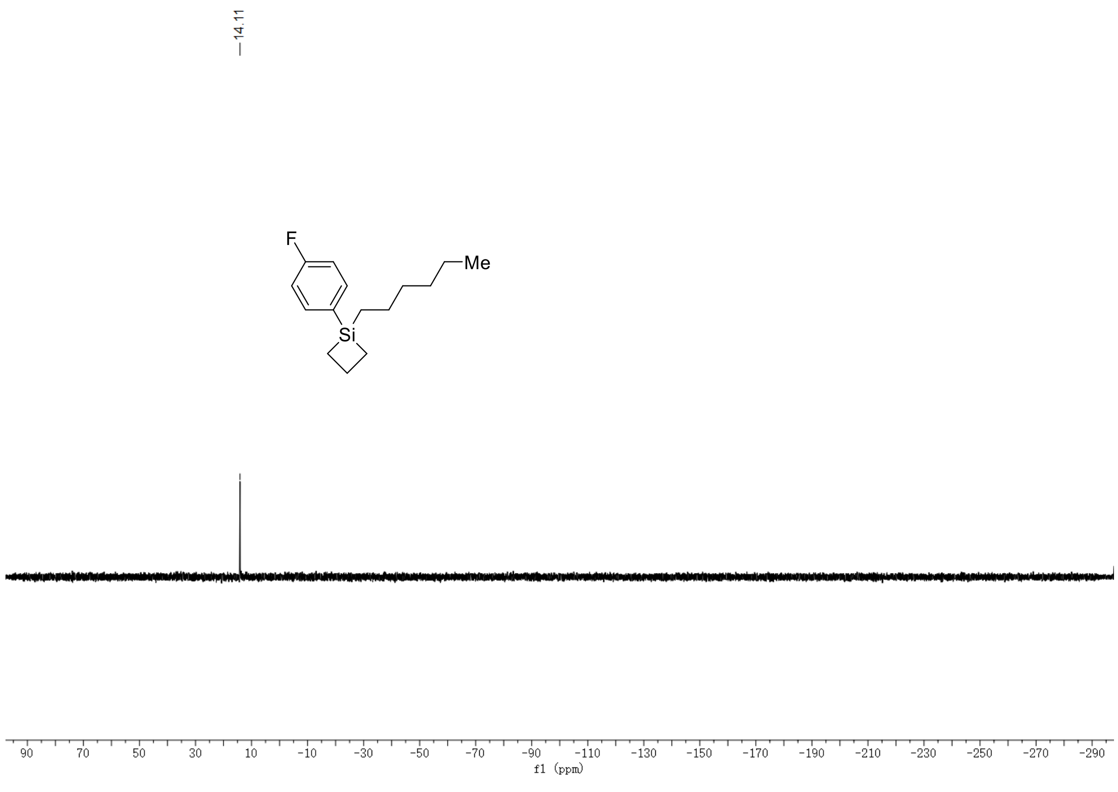

^{1}H NMR of **3aa** (400 MHz, $CDCl_3$, 25 ºC)

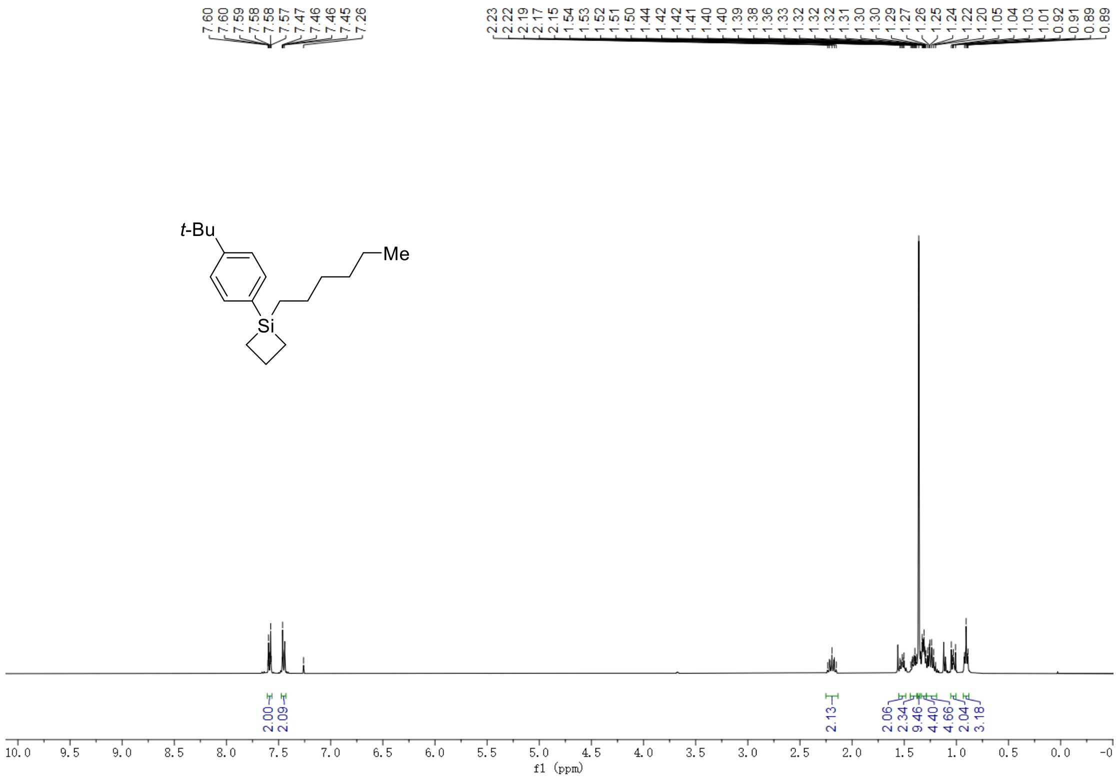

^{13}C NMR of **3aa** (151 MHz, $CDCl_3$, 25 ºC)

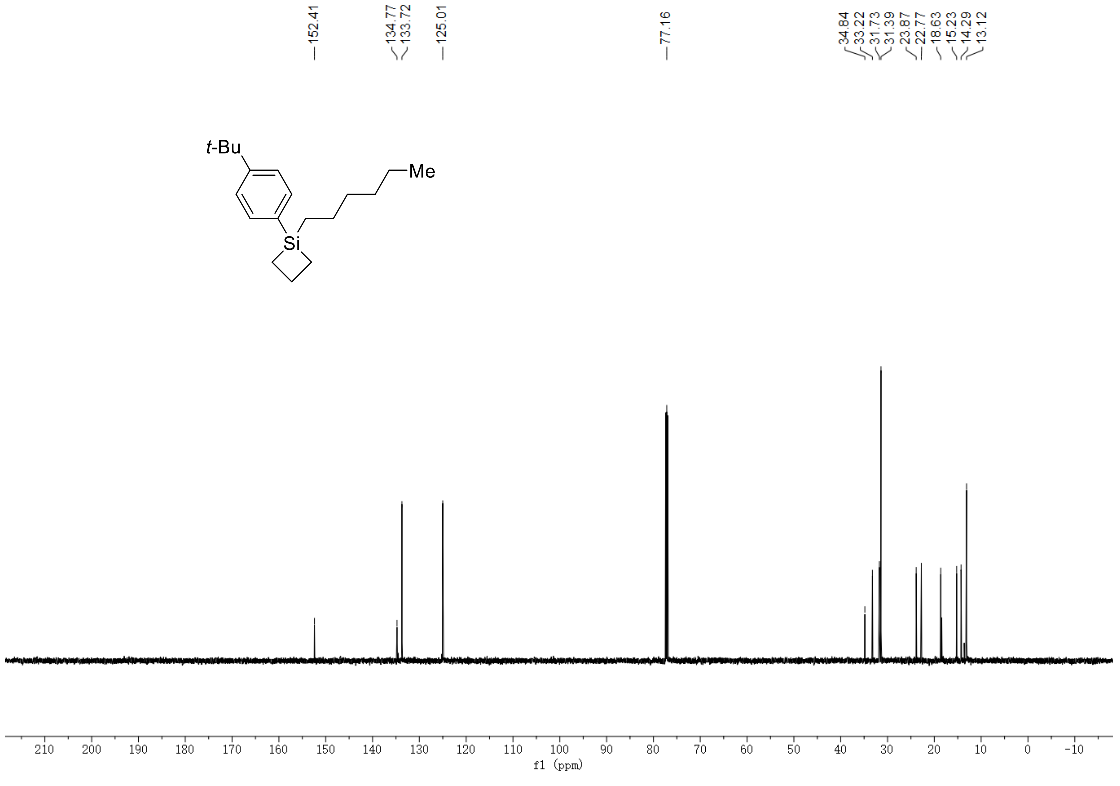

^{29}Si NMR of **3aa** (119 MHz, $CDCl_3$, 25 ºC)

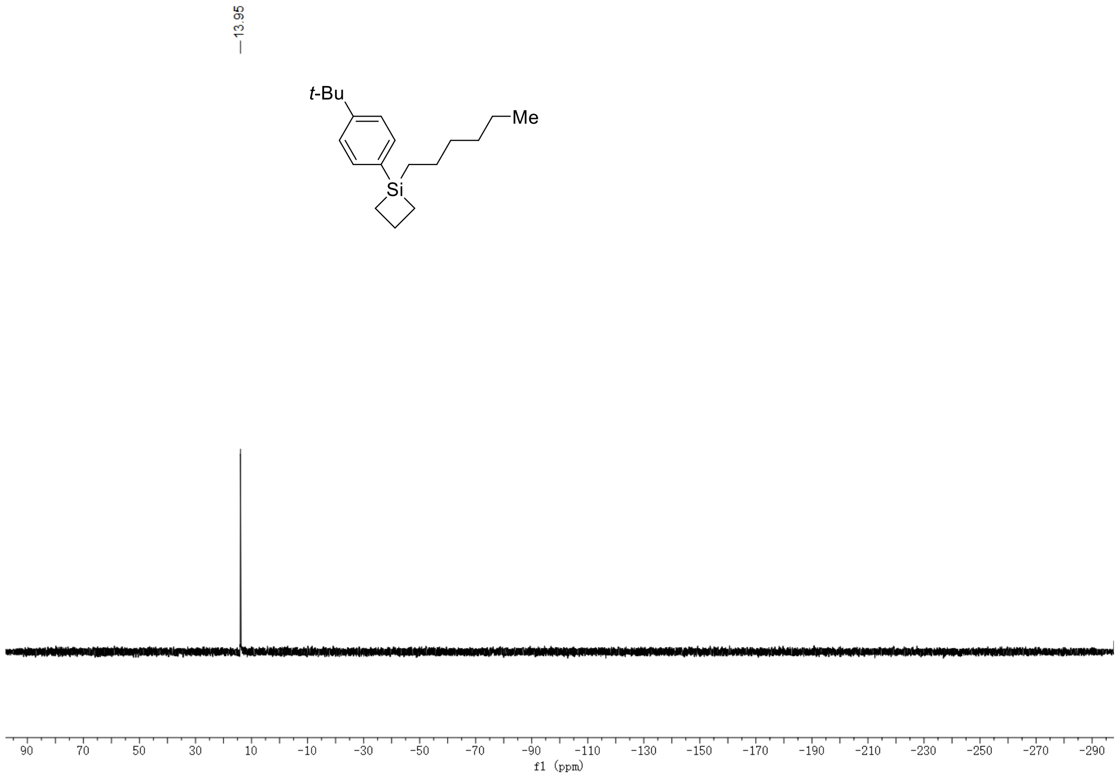

^{1}H NMR of **3ab** (600 MHz, $CDCl_3$, 25 ºC)

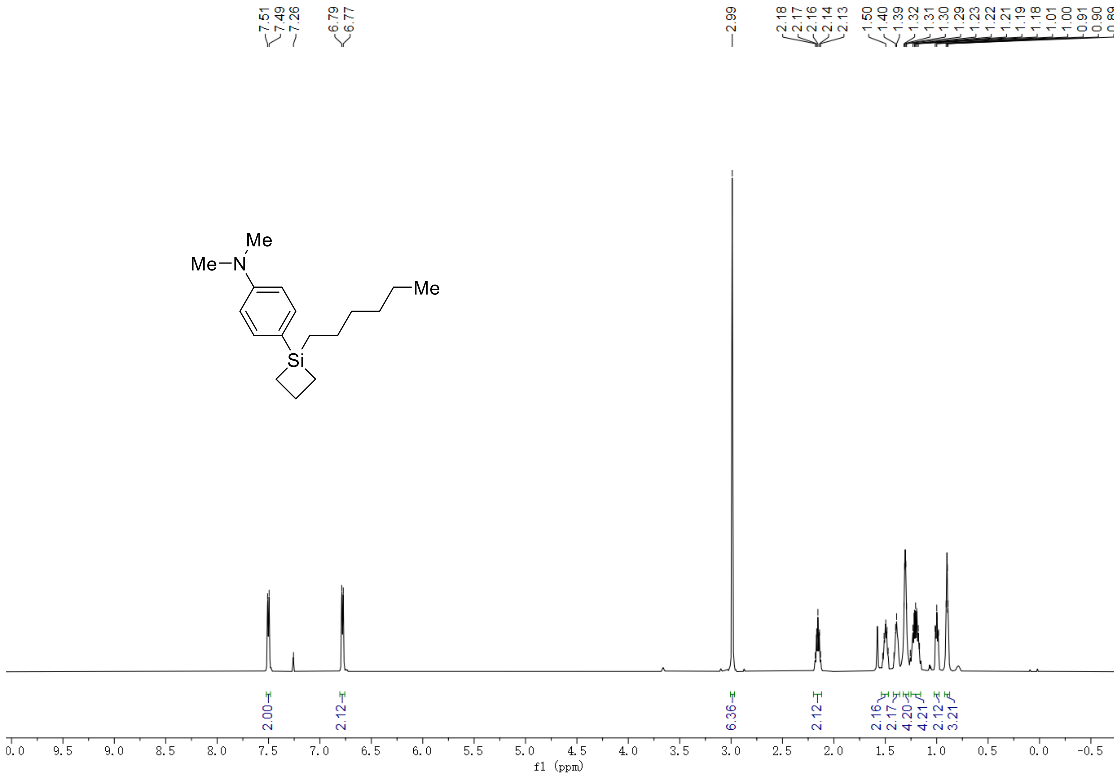

^{13}C NMR of **3ab** (151 MHz, $CDCl_3$, 25 ºC)

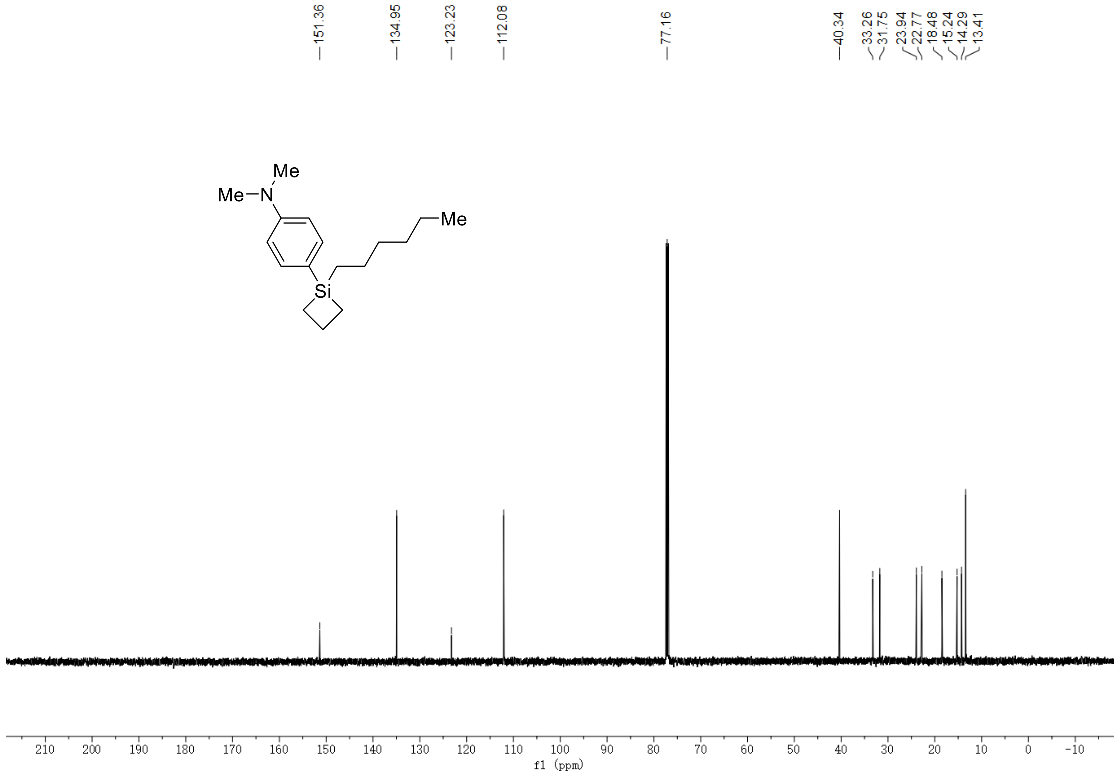

^{29}Si NMR of **3ab** (119 MHz, $CDCl_3$, 25 ºC)

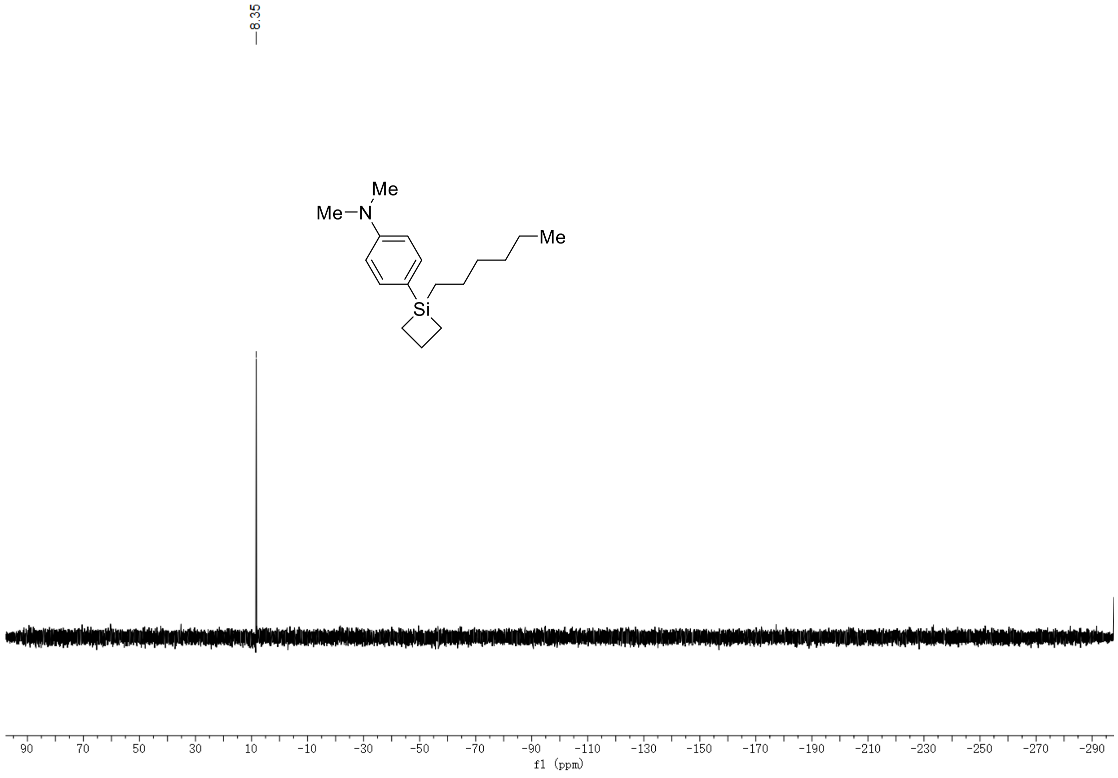

^{1}H NMR of **3ac** (600 MHz, $CDCl_3$, 25 ºC)

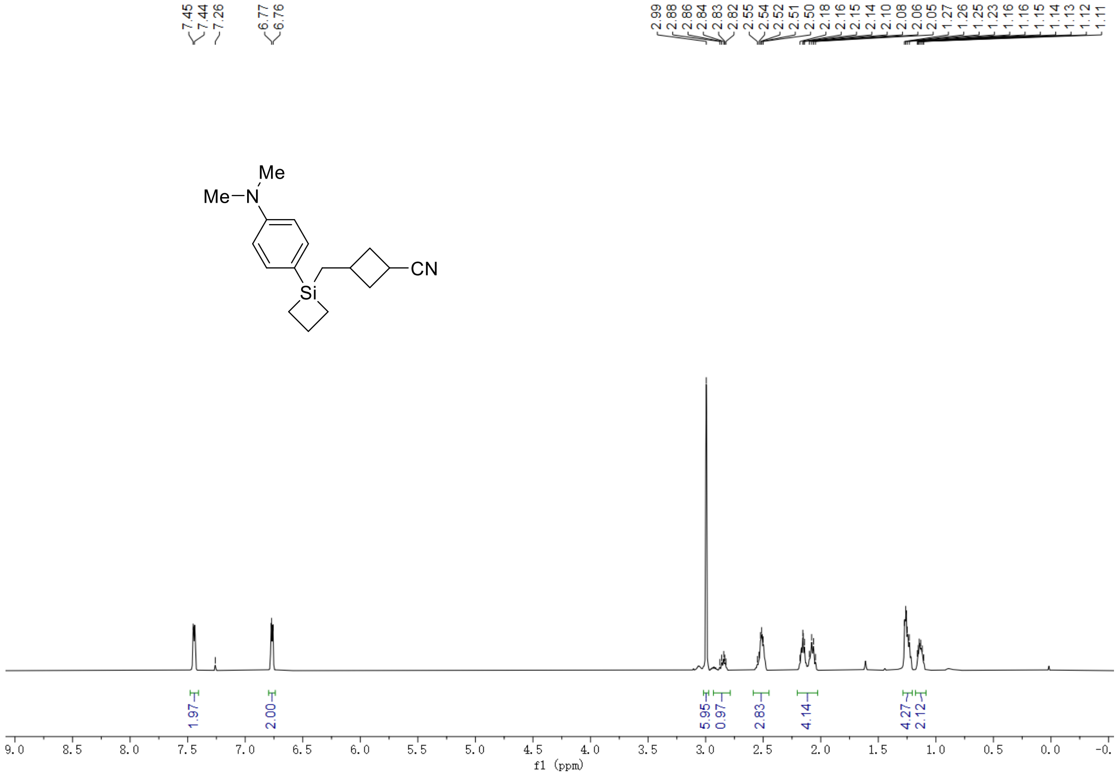

^{13}C NMR of **3ac** (151 MHz, $CDCl_3$, 25 ºC)

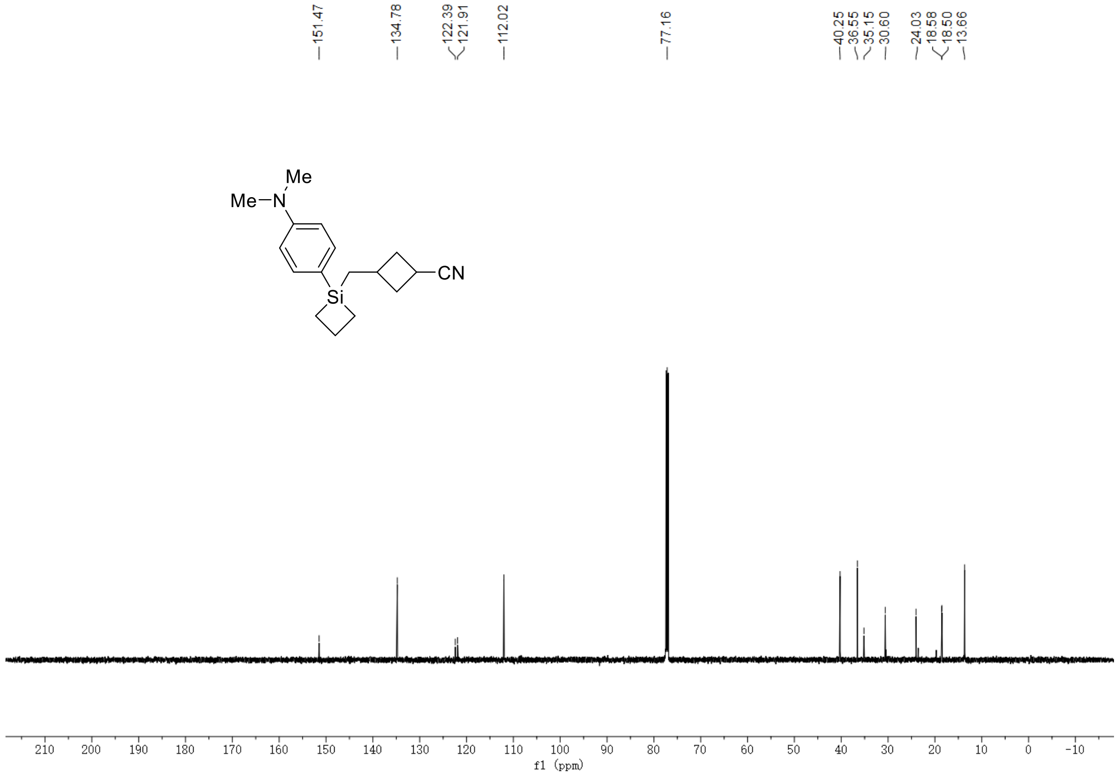

^{29}Si NMR of **3ac** (119 MHz, $CDCl_3$, 25 ºC)

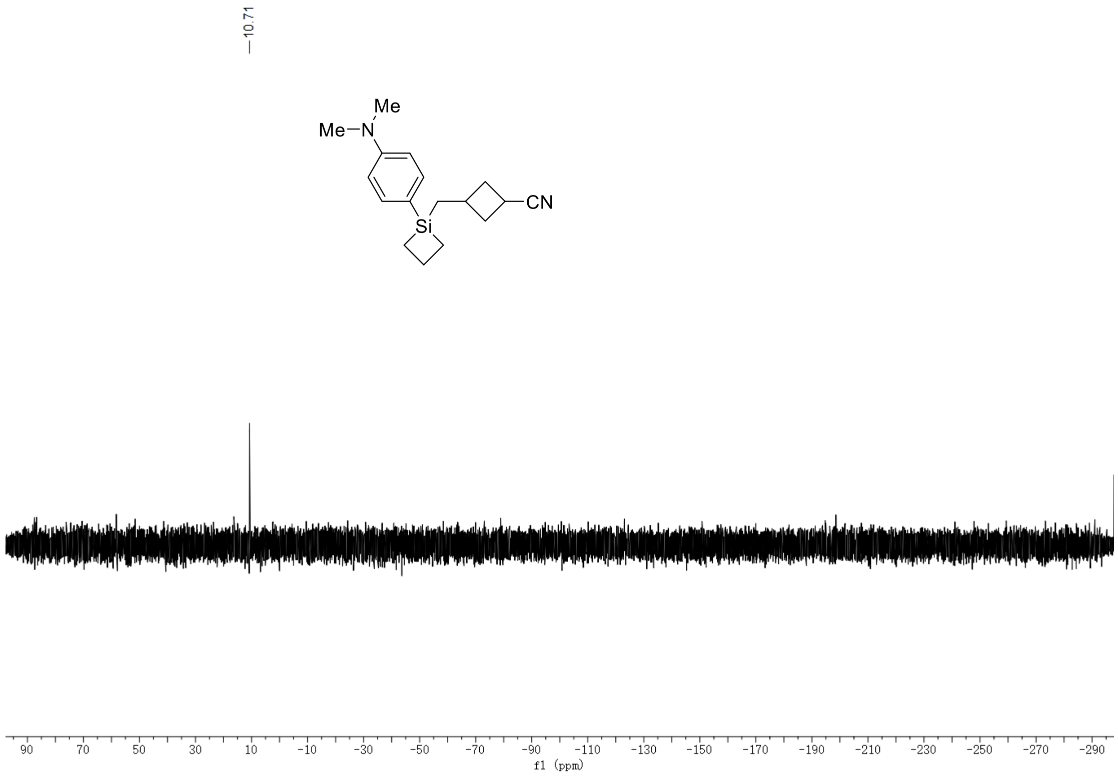

^{1}H NMR of **3ad** (400 MHz, $CDCl_3$, 25 ºC)

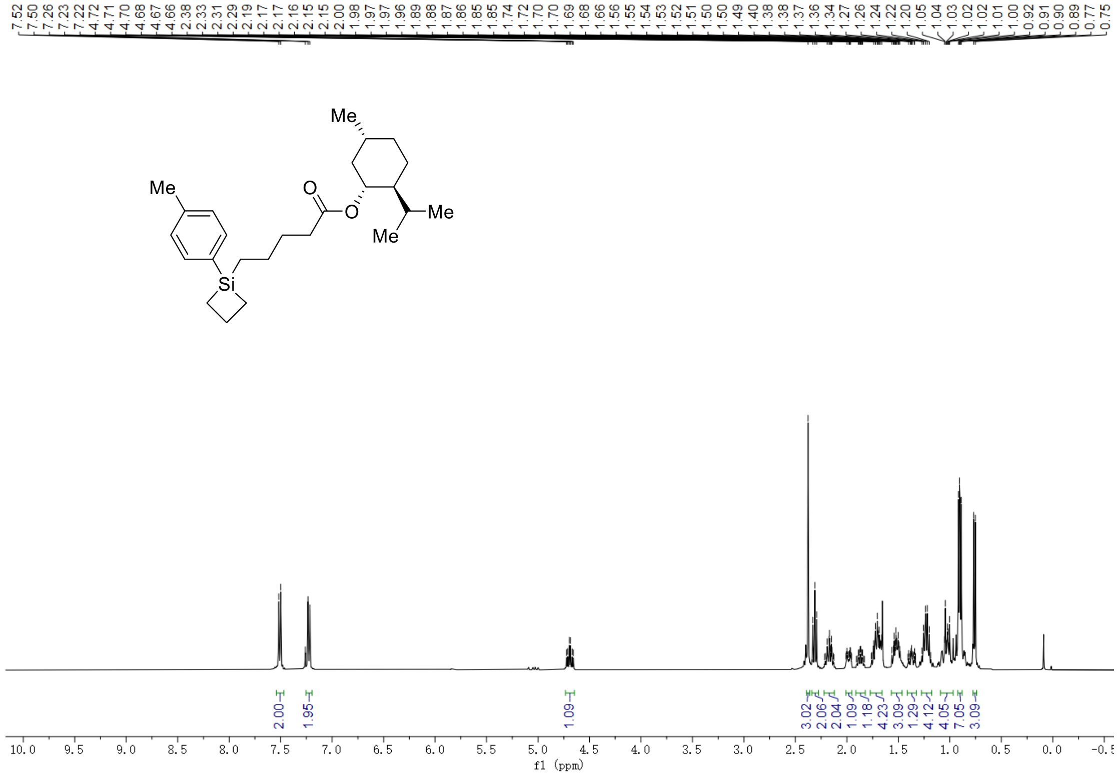

^{13}C NMR of **3ad** (101 MHz, $CDCl_3$, 25 ºC)

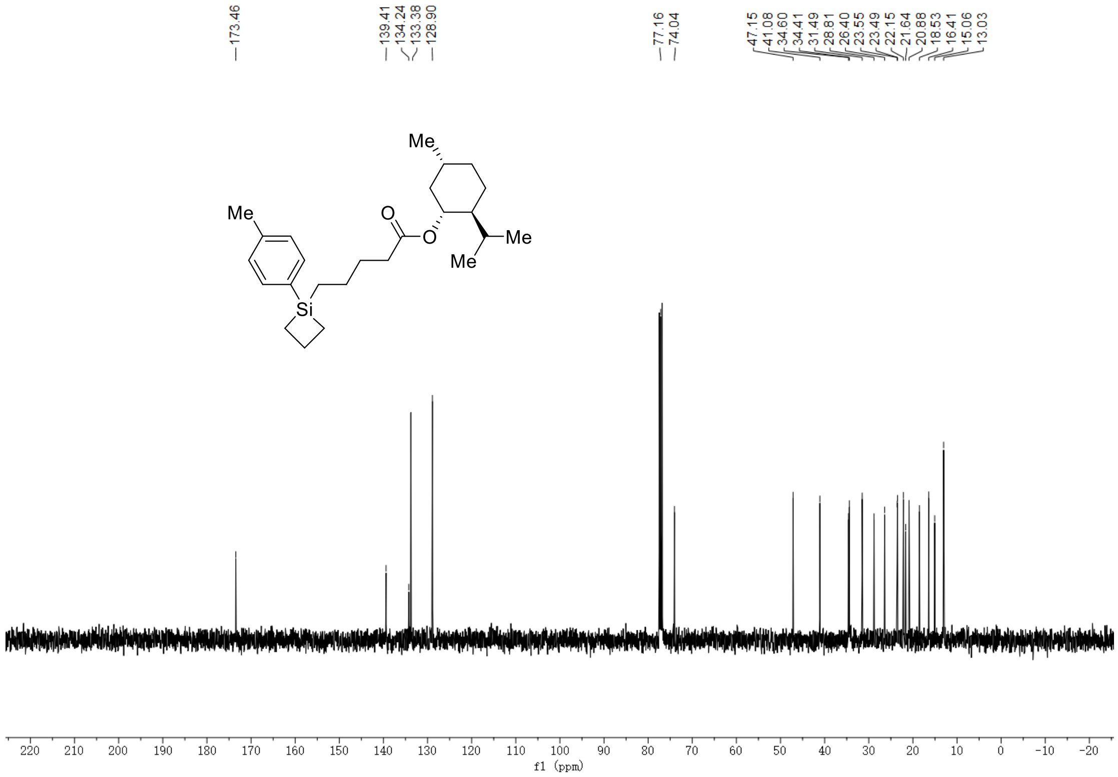

^{29}Si NMR of **3ad** (119 MHz, $CDCl_3$, 25 ºC)

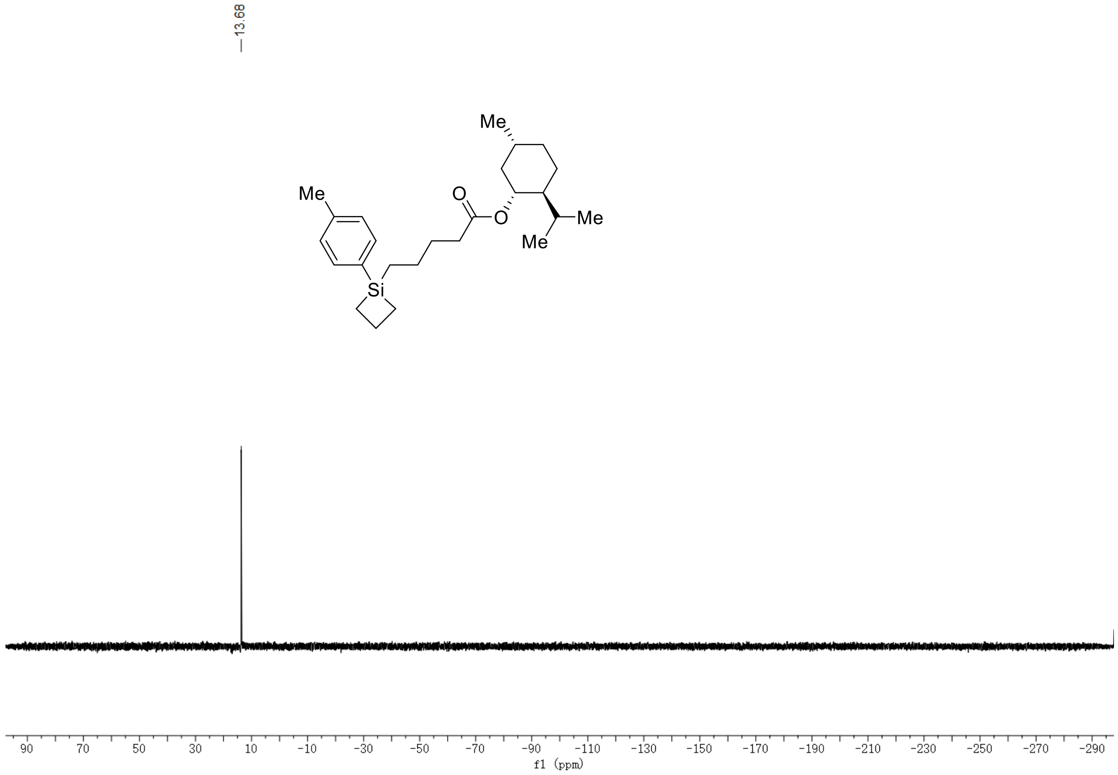

^{1}H NMR of **3ae** (400 MHz, $CDCl_3$, 25 ºC)

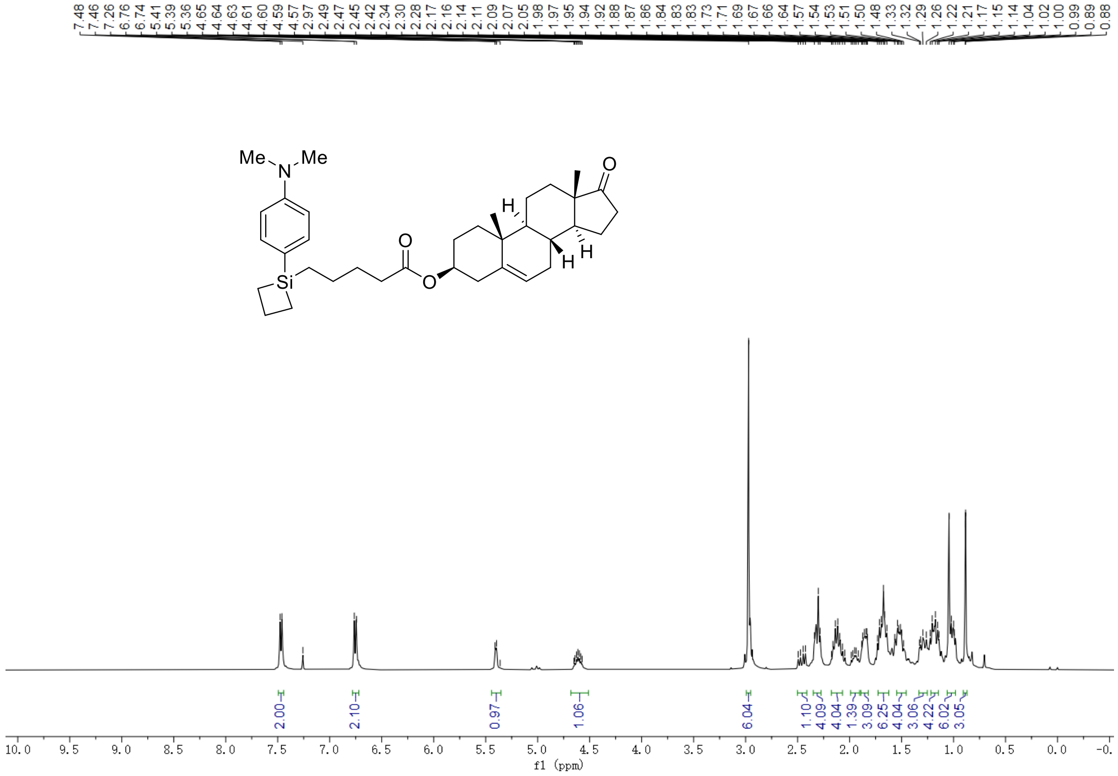

^{13}C NMR of **3ae** (151 MHz, $CDCl_3$, 25 ºC)

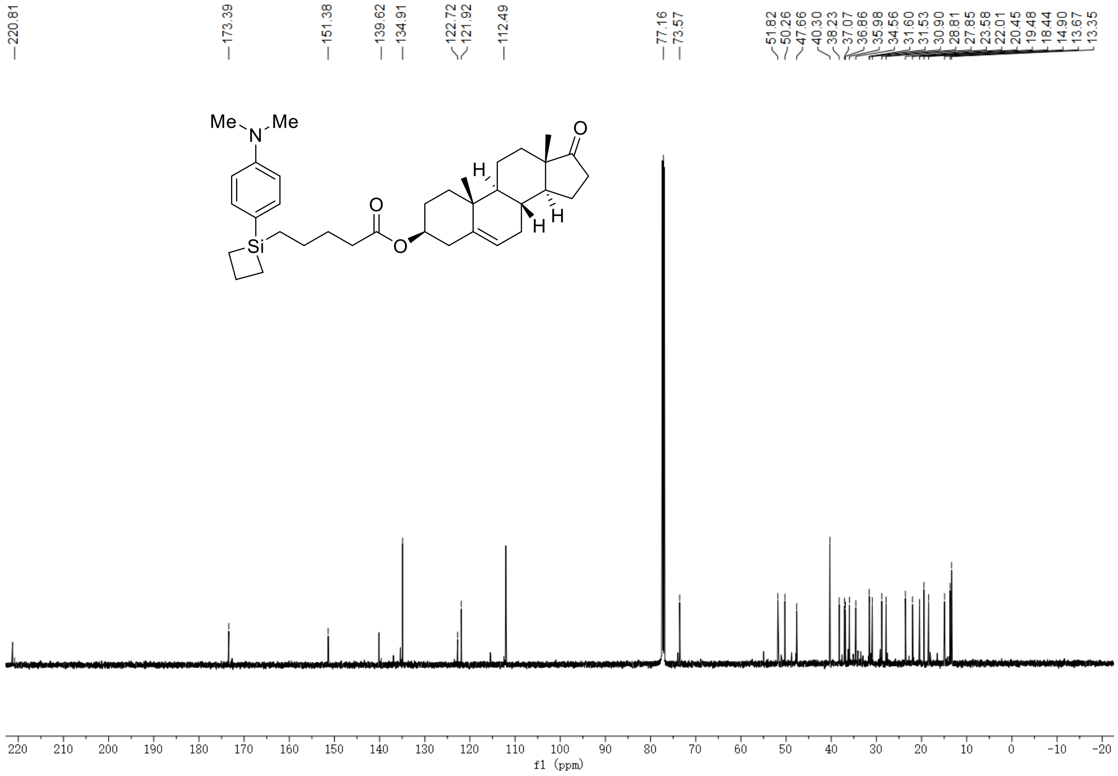

^{29}Si NMR of **3ae** (119 MHz, $CDCl_3$, 25 ºC)

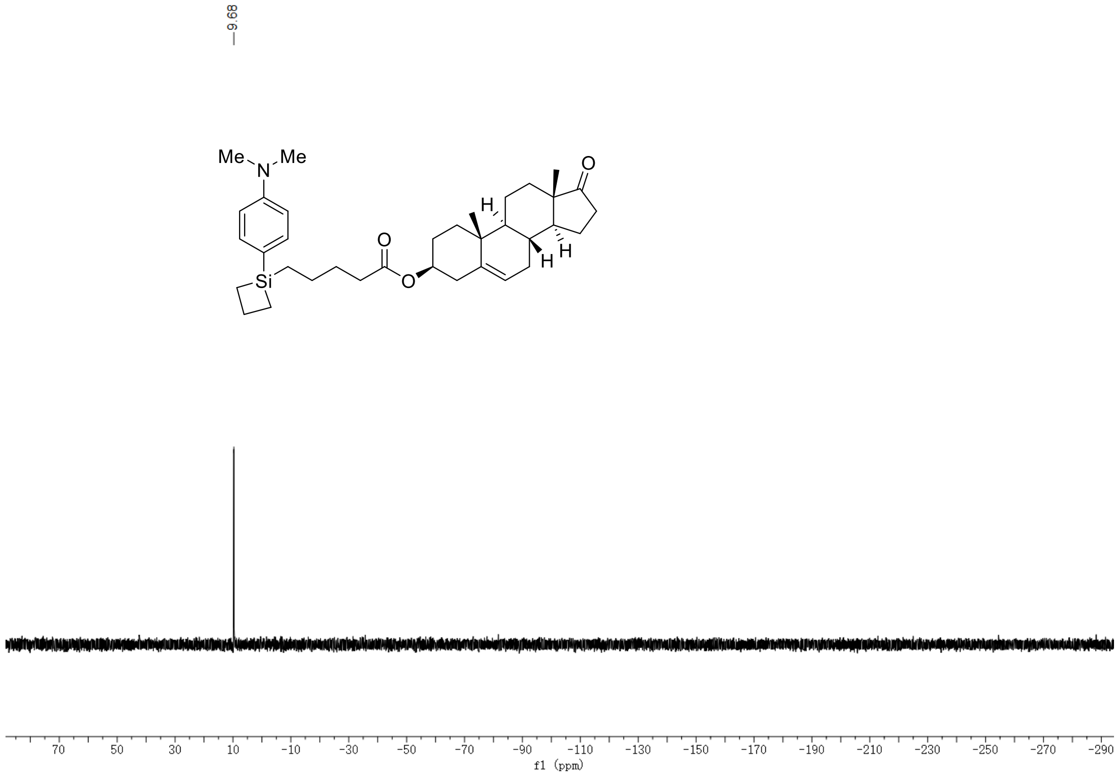

^{1}H NMR of **3af** (400 MHz, $CDCl_3$, 25 ºC)

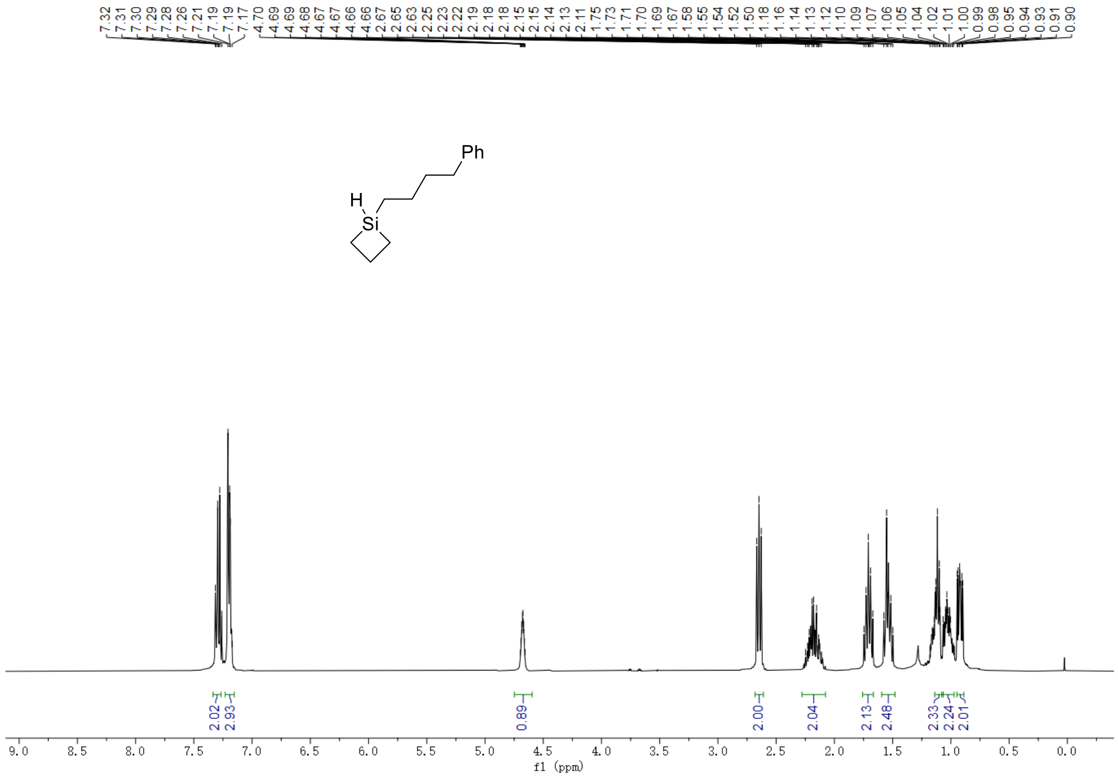

^{13}C NMR of **3af** (151 MHz, $CDCl_3$, 25 ºC)

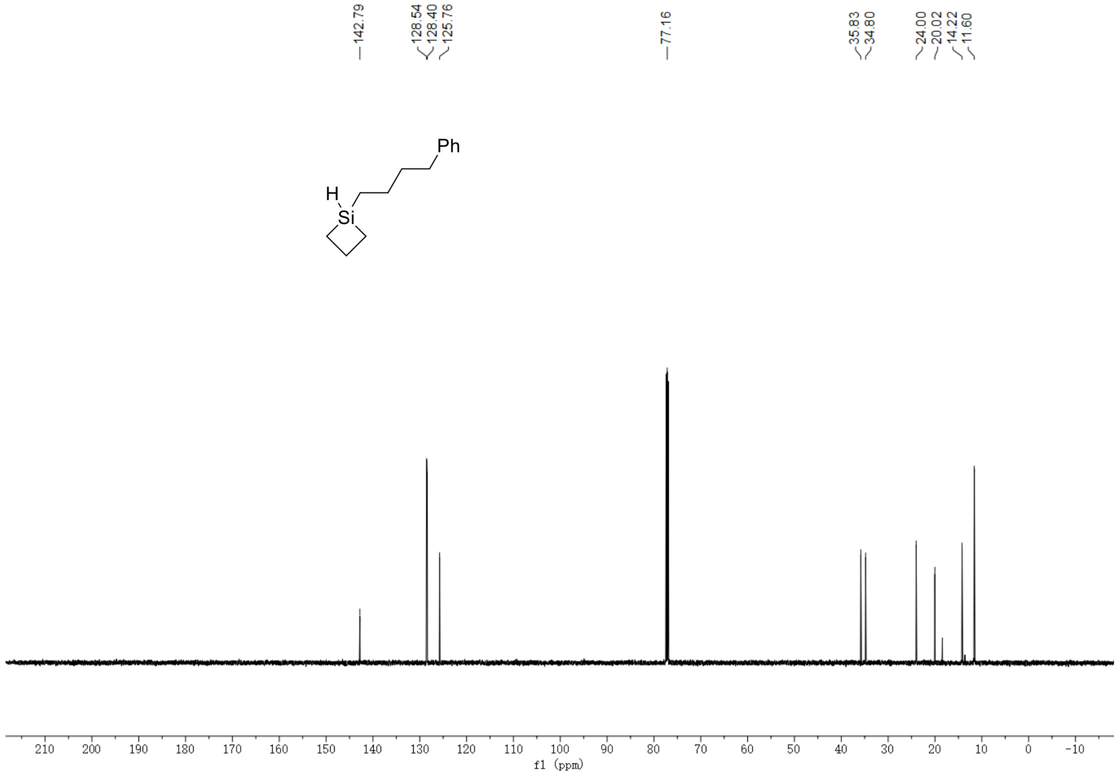

^{29}Si NMR of **3af** (119 MHz, $CDCl_3$, 25 ºC)

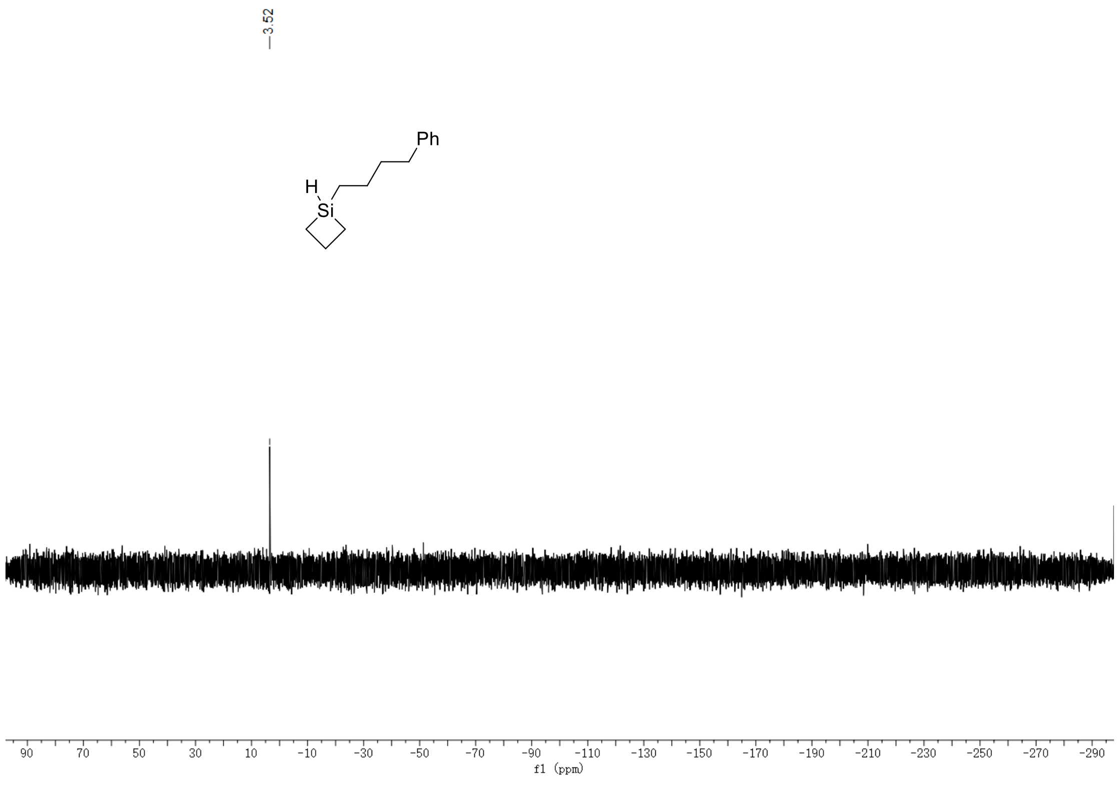

^{1}H NMR of **3ag** (600 MHz, $CDCl_3$, 25 ºC)

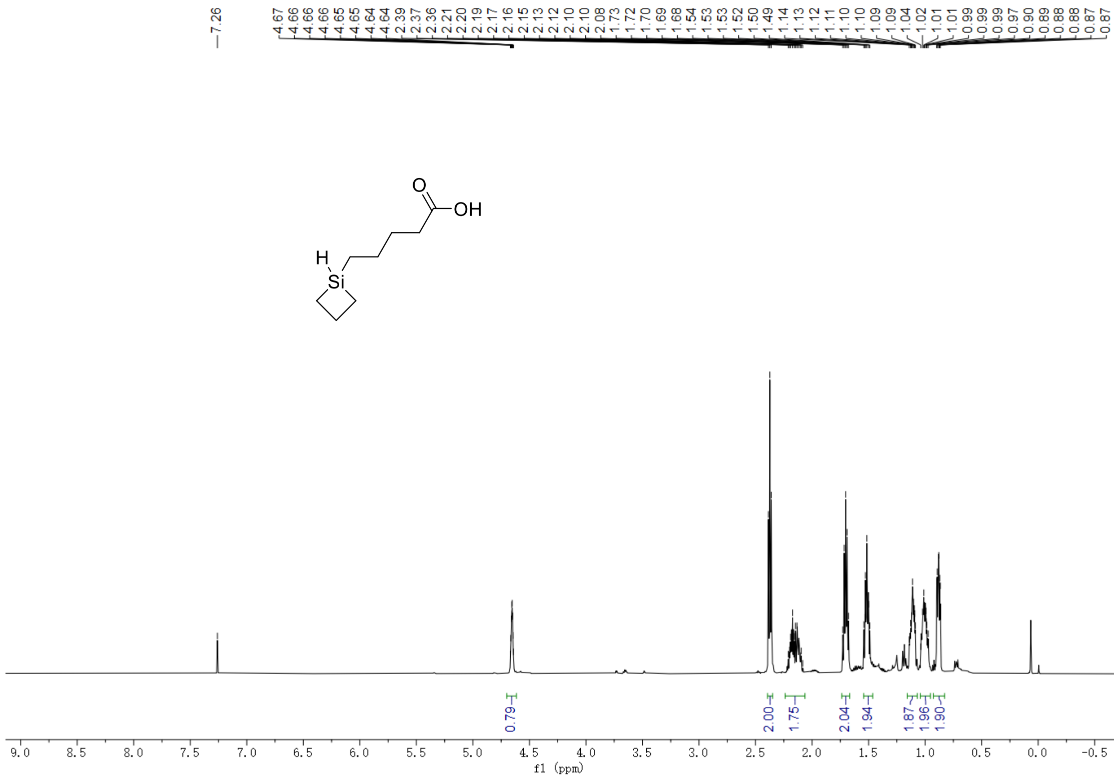

^{13}C NMR of **3ag** (151 MHz, $CDCl_3$, 25 ºC)

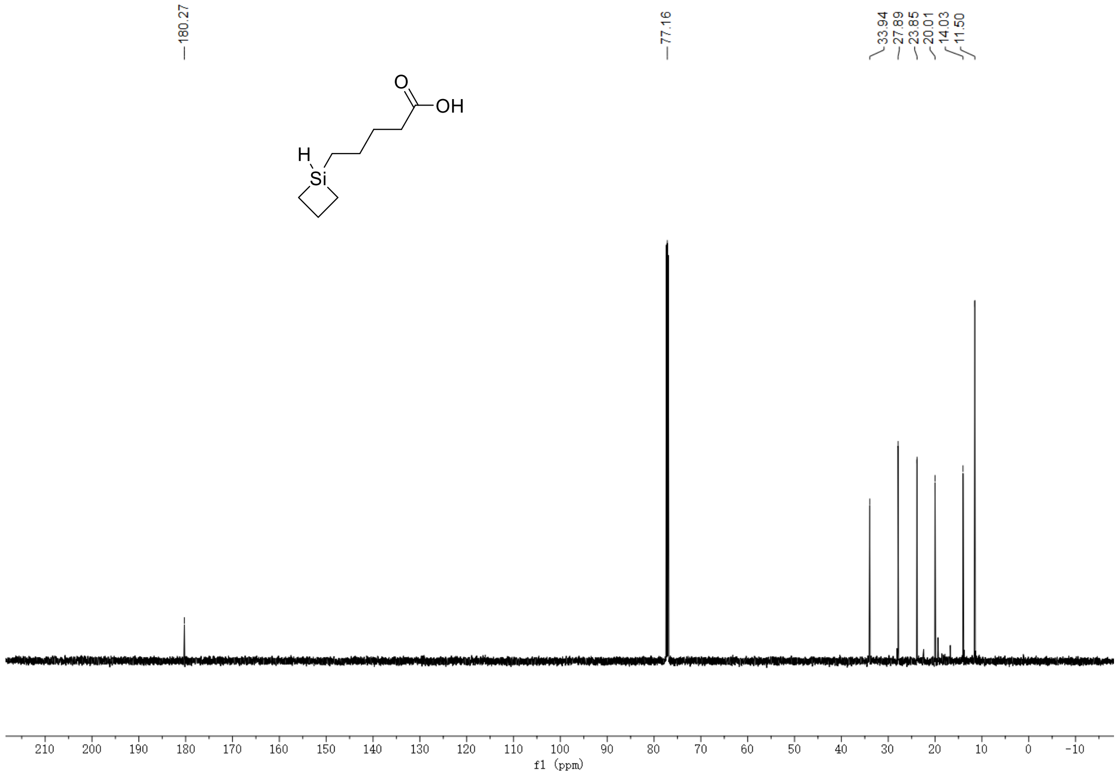

^{29}Si NMR of **3ag** (119 MHz, $CDCl_3$, 25 ºC)

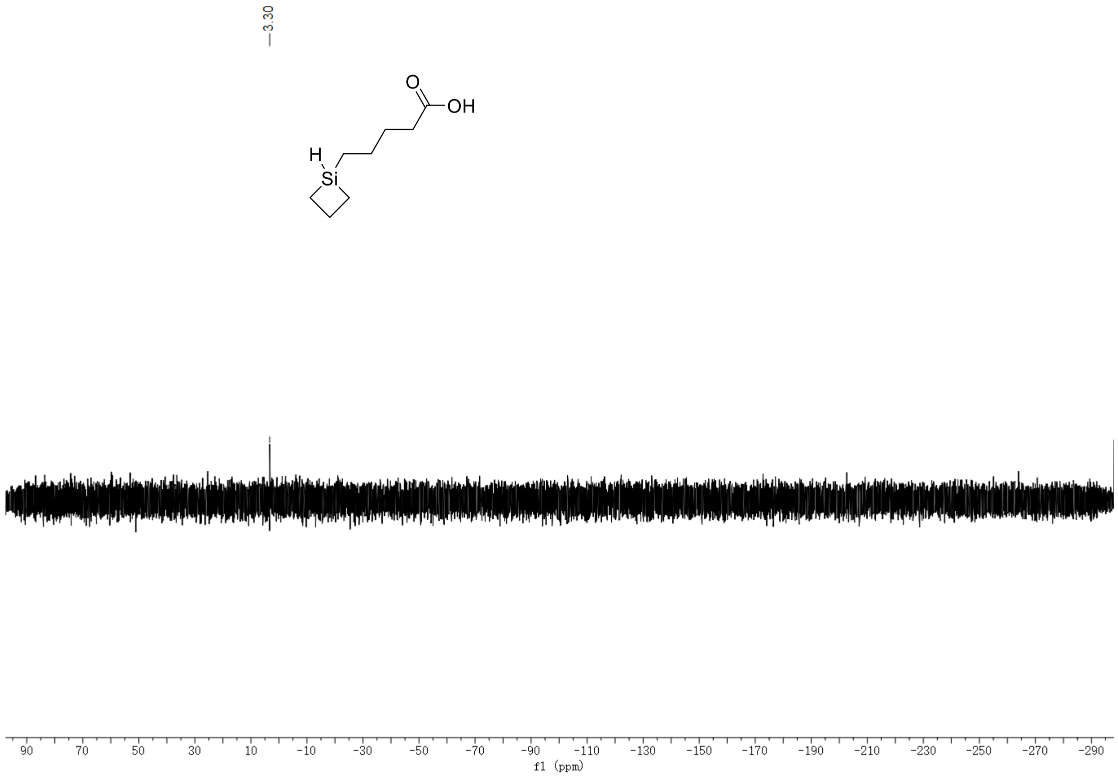

^{1}H NMR of **3ah** (600 MHz, $CDCl_3$, 25 ºC)

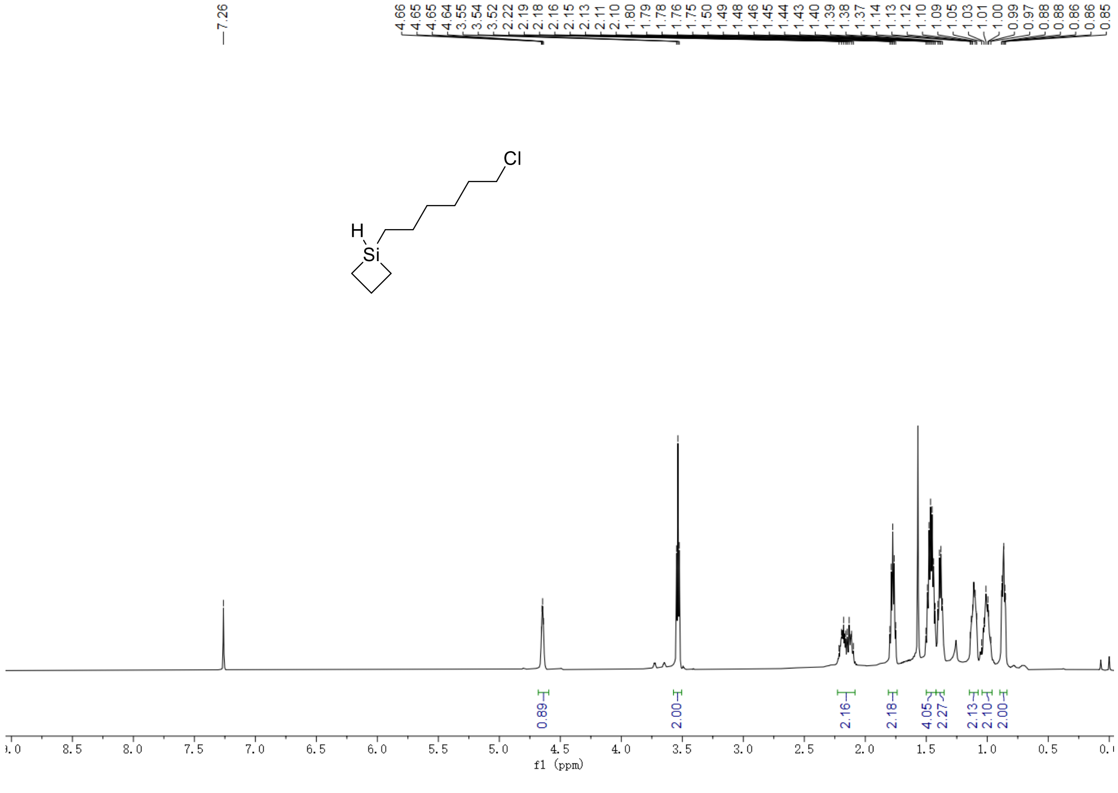

^{13}C NMR of **3ah** (151 MHz, $CDCl_3$, 25 ºC)

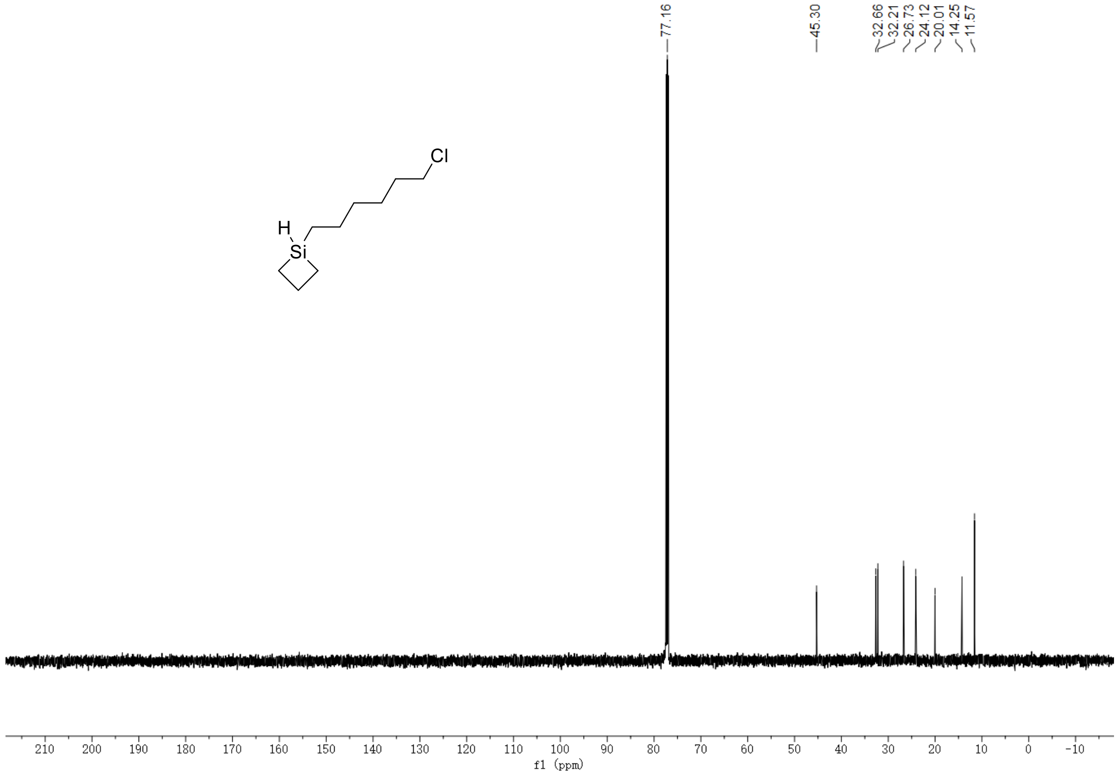

^{29}Si NMR of **3af** (119 MHz, $CDCl_3$, 25 ºC)

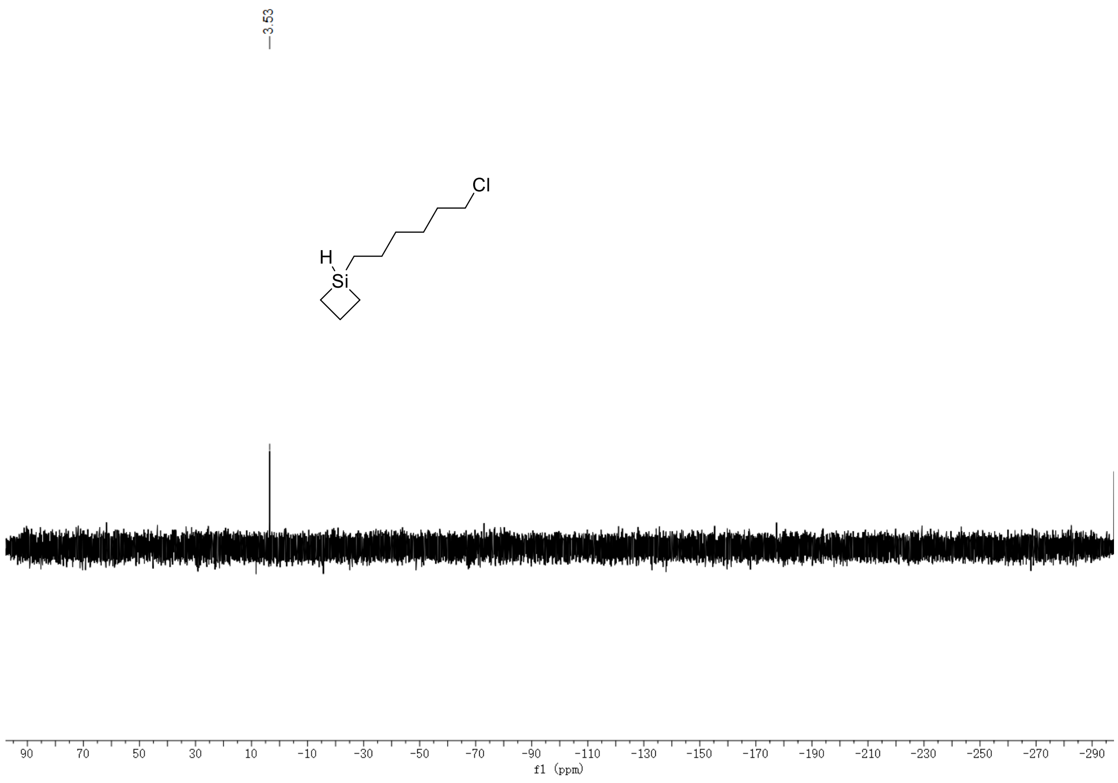

^{1}H NMR of **3ai** (400 MHz, $CDCl_3$, 25 ºC)

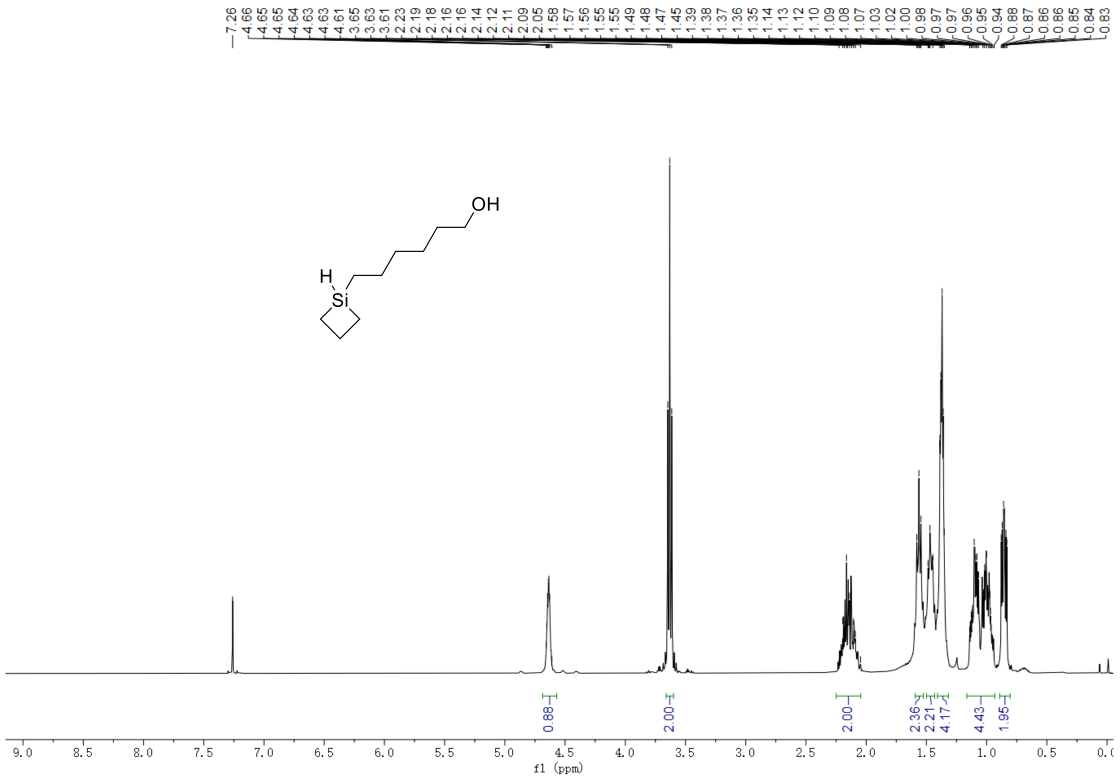

^{13}C NMR of **3ai** (151 MHz, $CDCl_3$, 25 ºC)

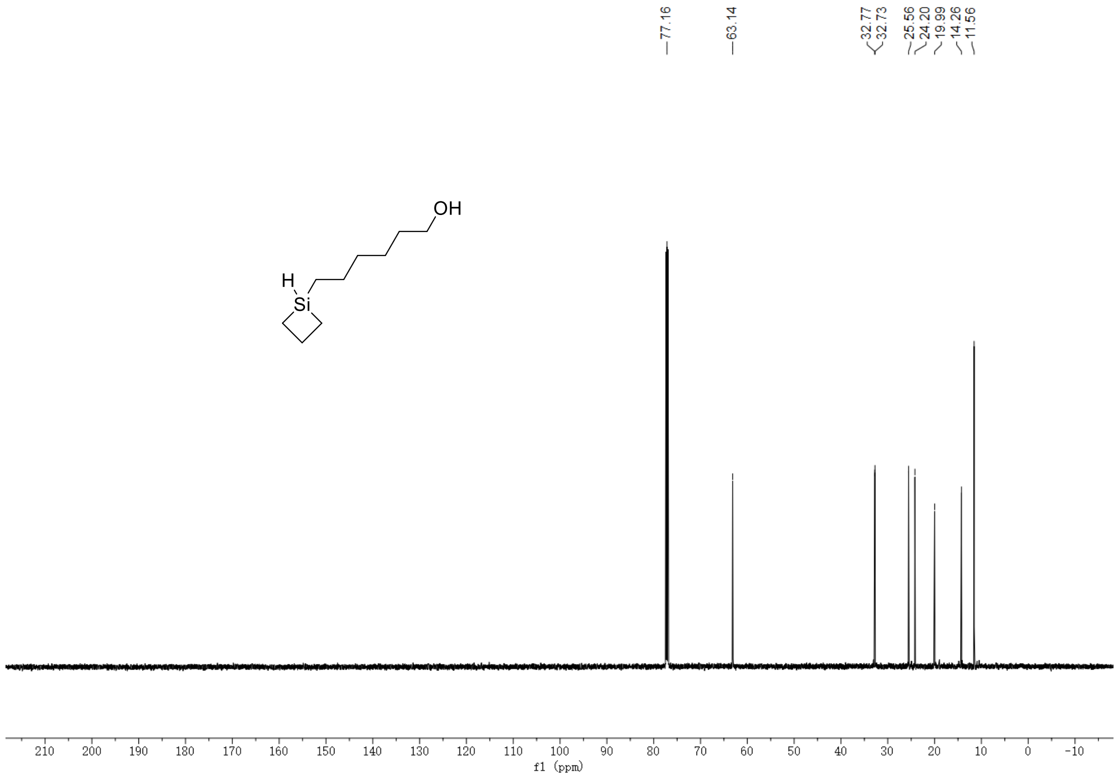

^{29}Si NMR of **3ai** (119 MHz, $CDCl_3$, 25 ºC)

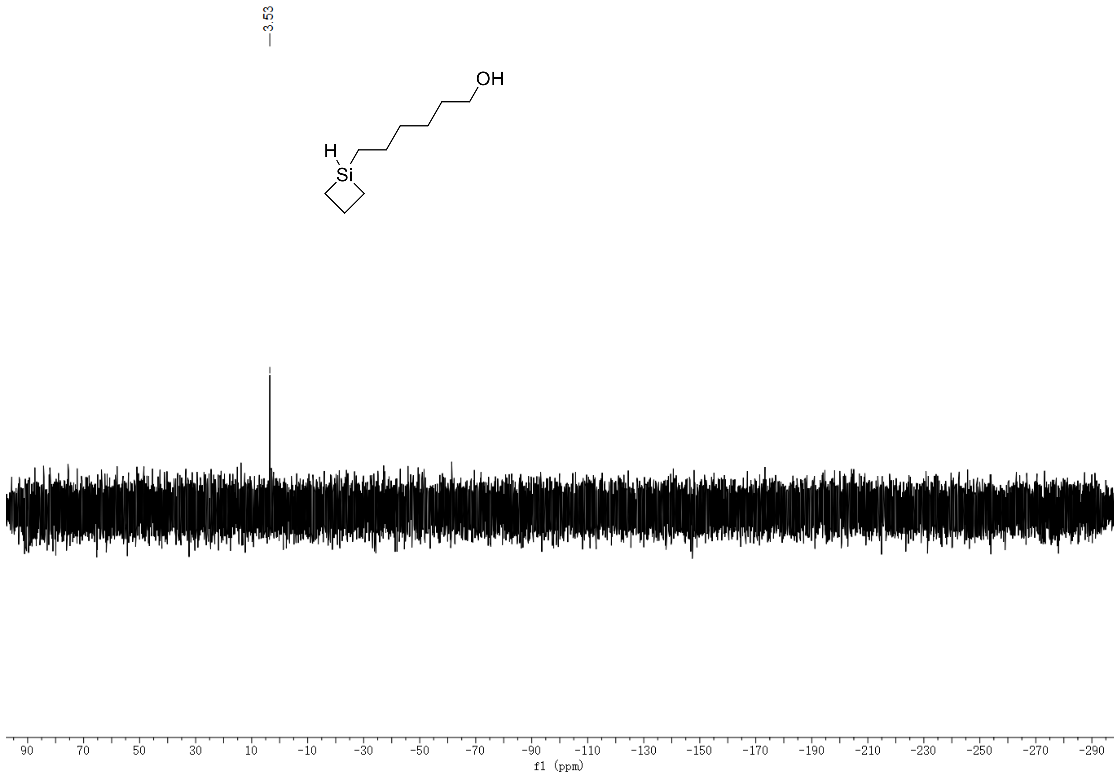

^{1}H NMR of **3aj** (400 MHz, $CDCl_3$, 25 ºC)

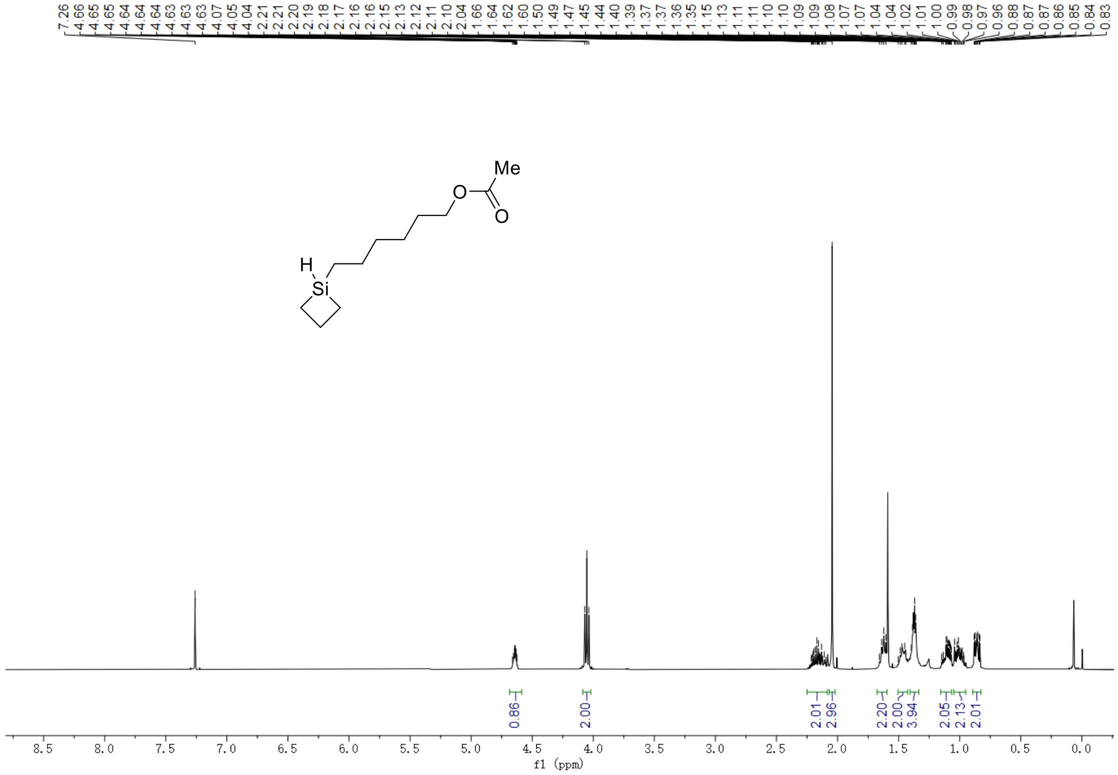

^{13}C NMR of **3aj** (151 MHz, $CDCl_3$, 25 ºC)

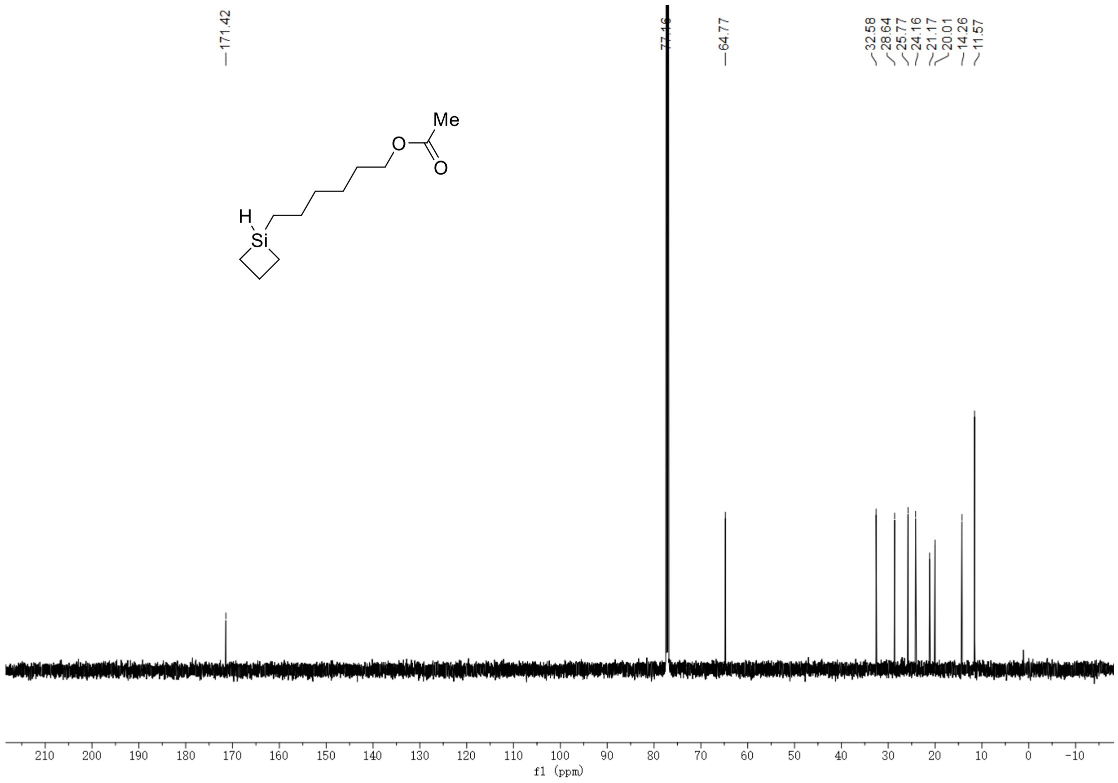

^{29}Si NMR of **3aj** (119 MHz, $CDCl_3$, 25 ºC)

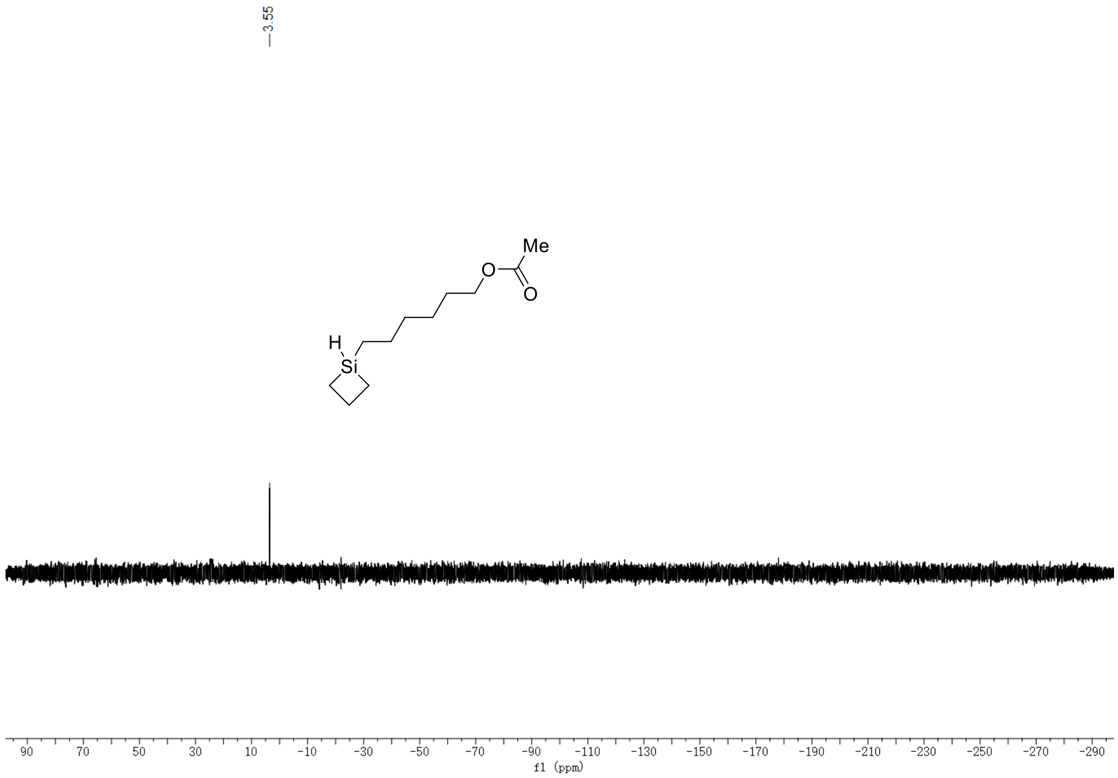

^{1}H NMR of **3ak** (400 MHz, $CDCl_3$, 25 ºC)

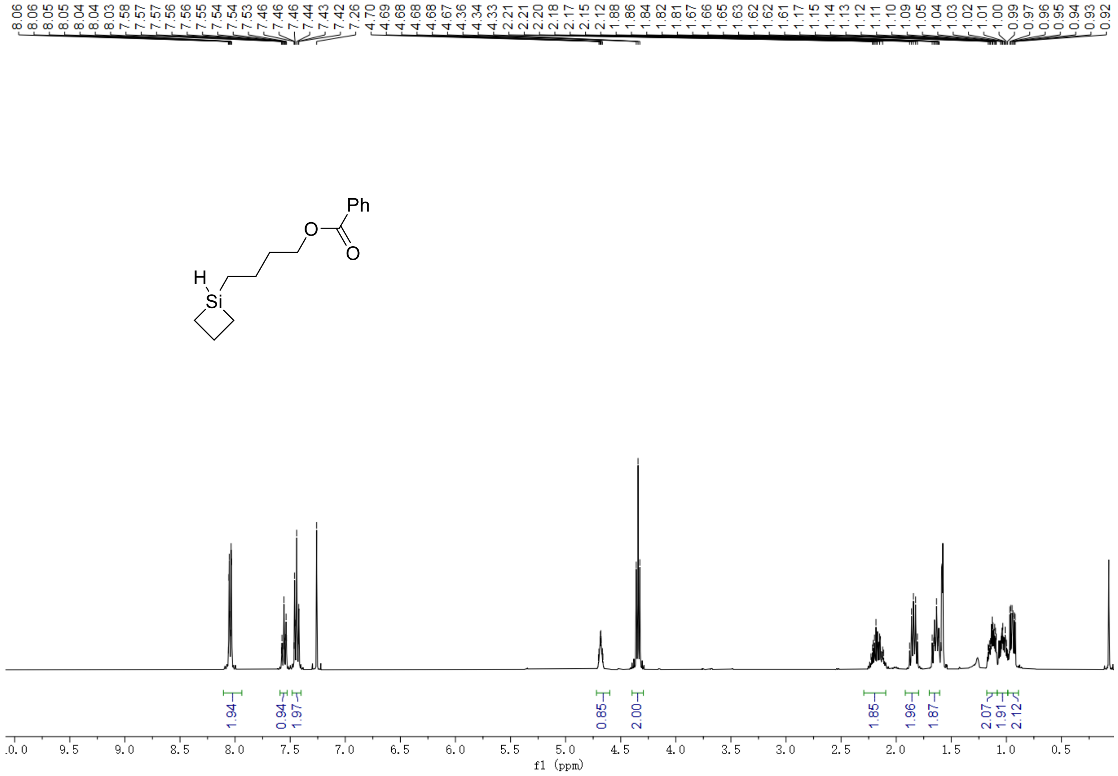

^{13}C NMR of **3ak** (151 MHz, $CDCl_3$, 25 ºC)

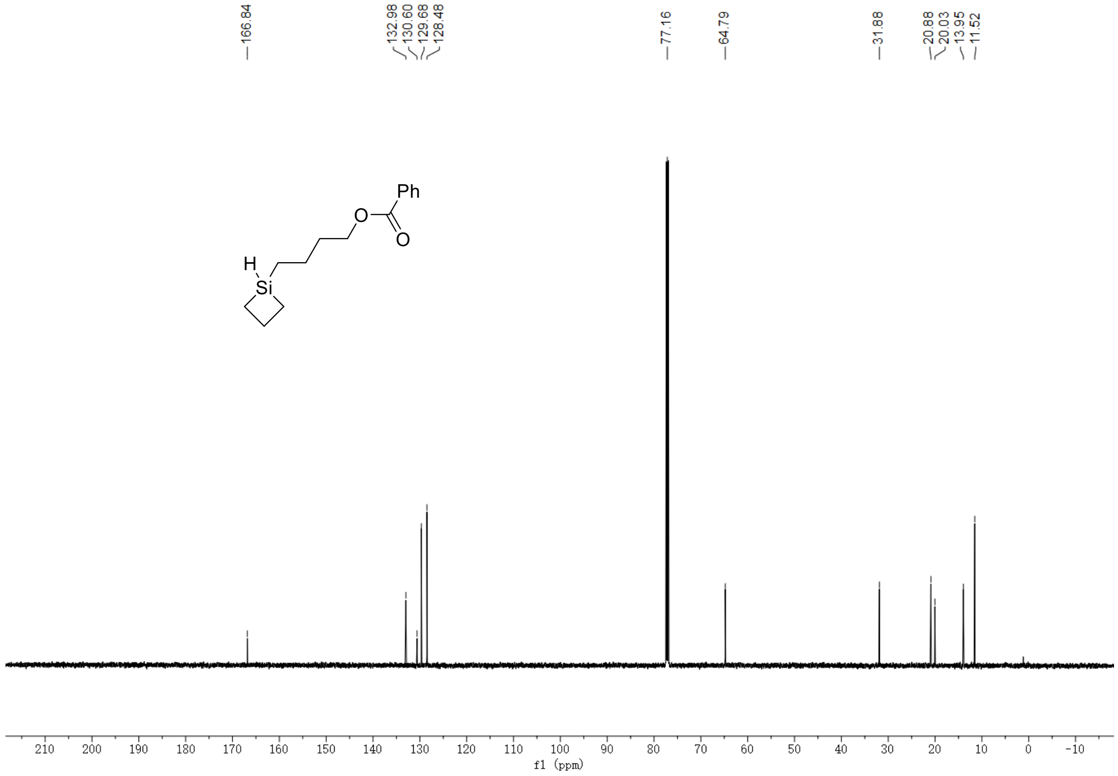

^{29}Si NMR of **3ak** (119 MHz, $CDCl_3$, 25 ºC)

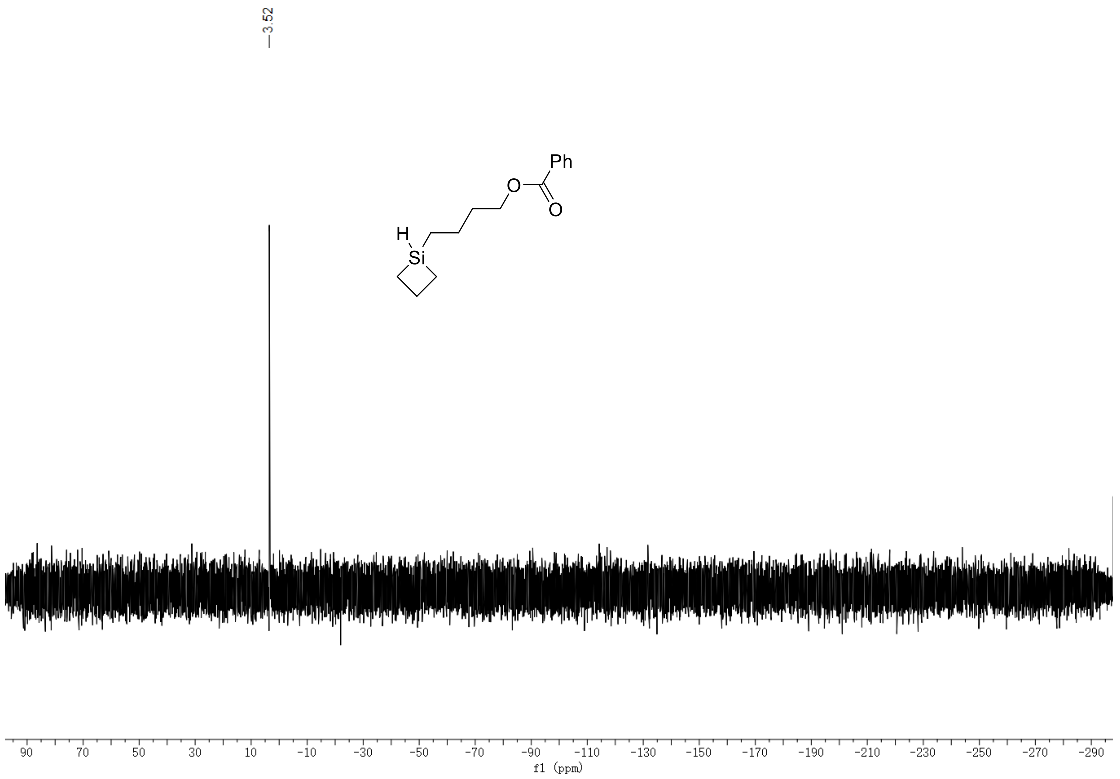

^{1}H NMR of **3al** (600 MHz, $CDCl_3$, 25 ºC)

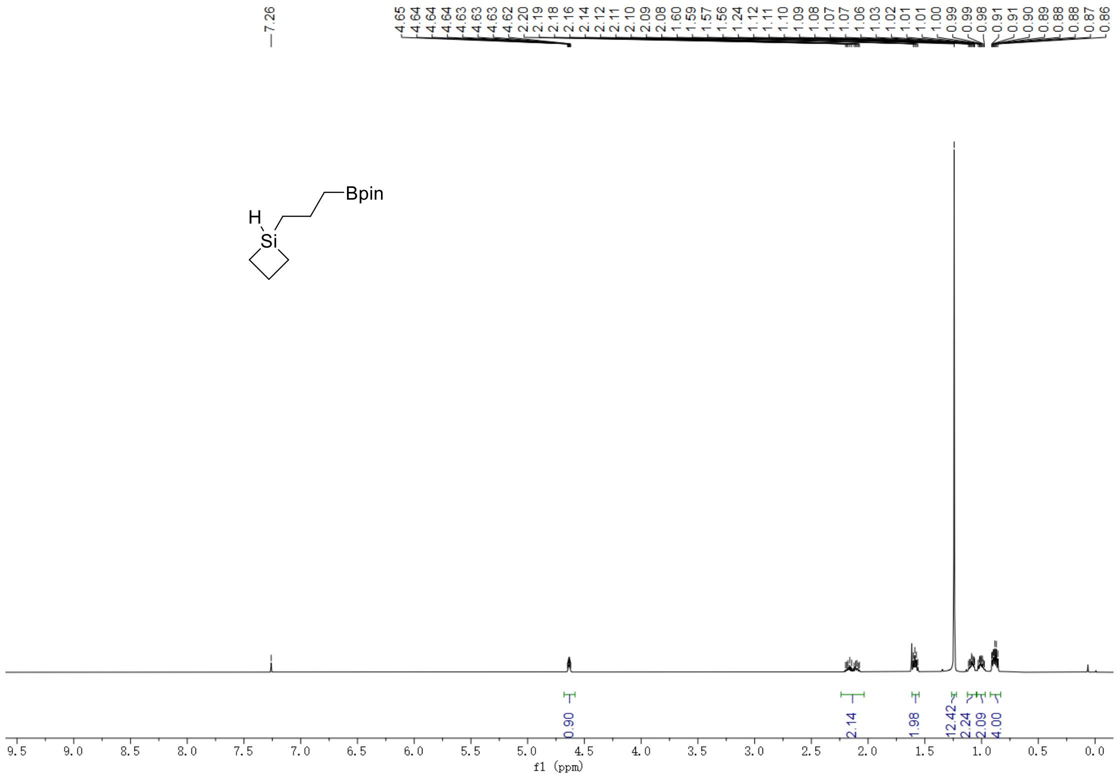

^{13}C NMR of **3al** (151 MHz, $CDCl_3$, 25 ºC)

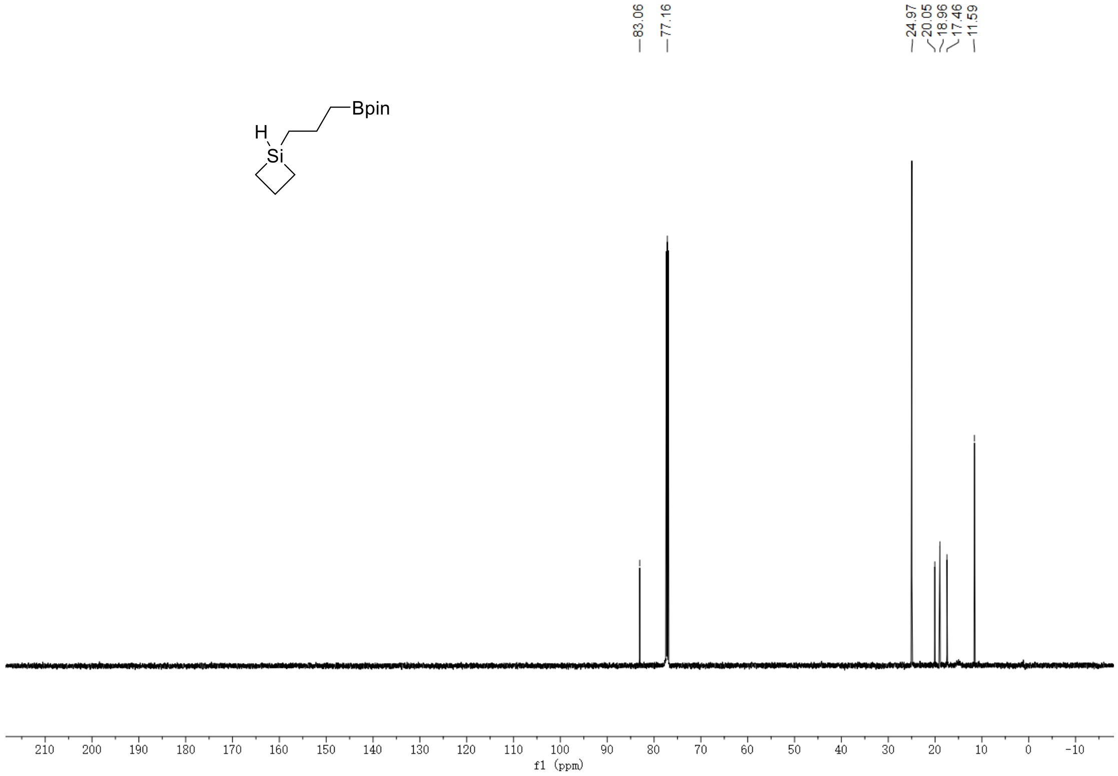

^{29}Si NMR of **3al** (119 MHz, $CDCl_3$, 25 ºC)

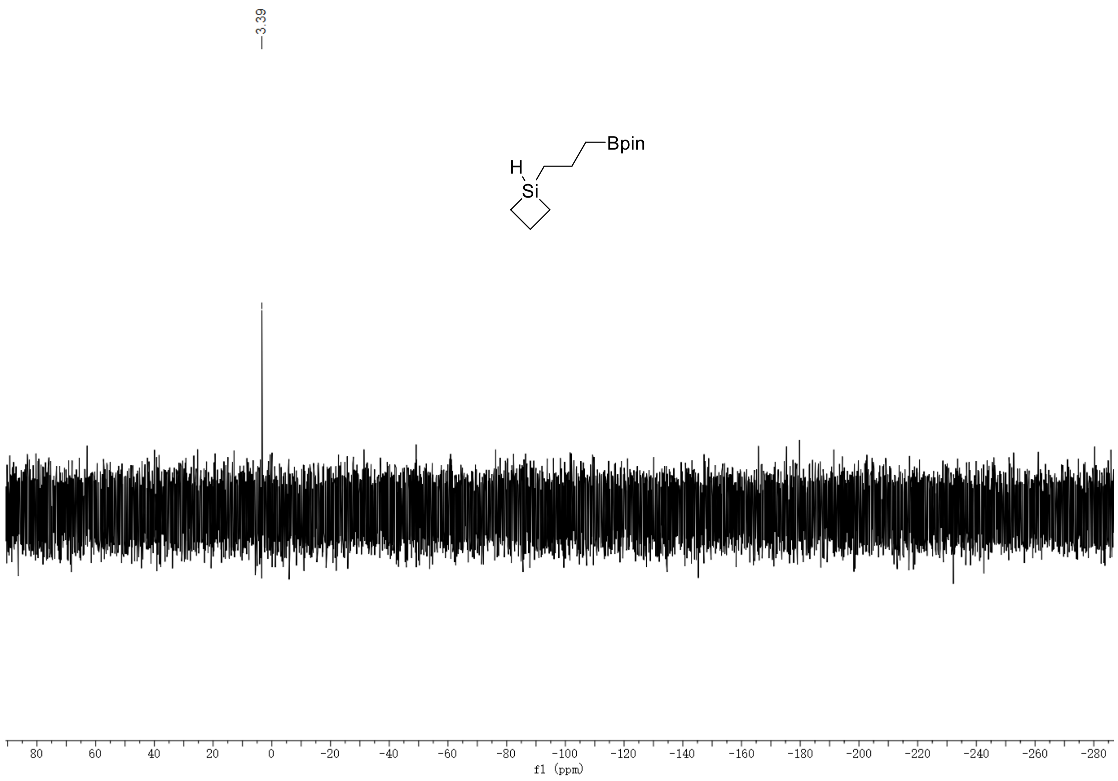

^{1}H NMR of **3am** (600 MHz, $CDCl_3$, 25 ºC)

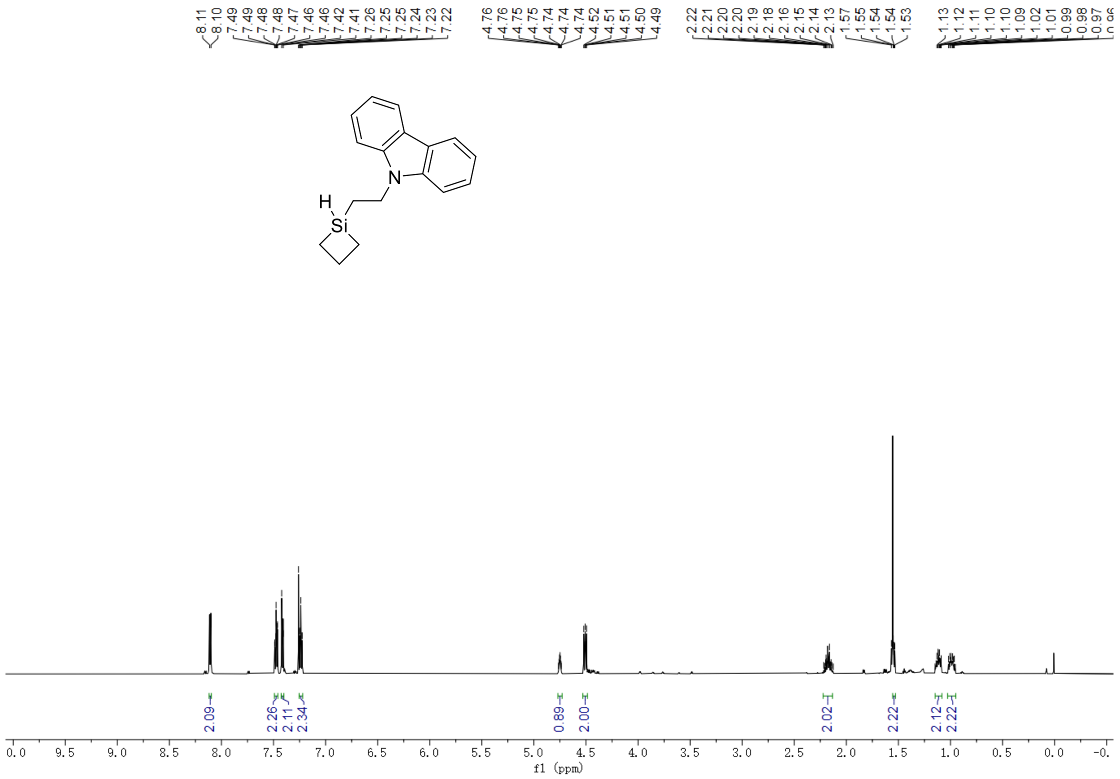

^{13}C NMR of **3am** (151 MHz, $CDCl_3$, 25 ºC)

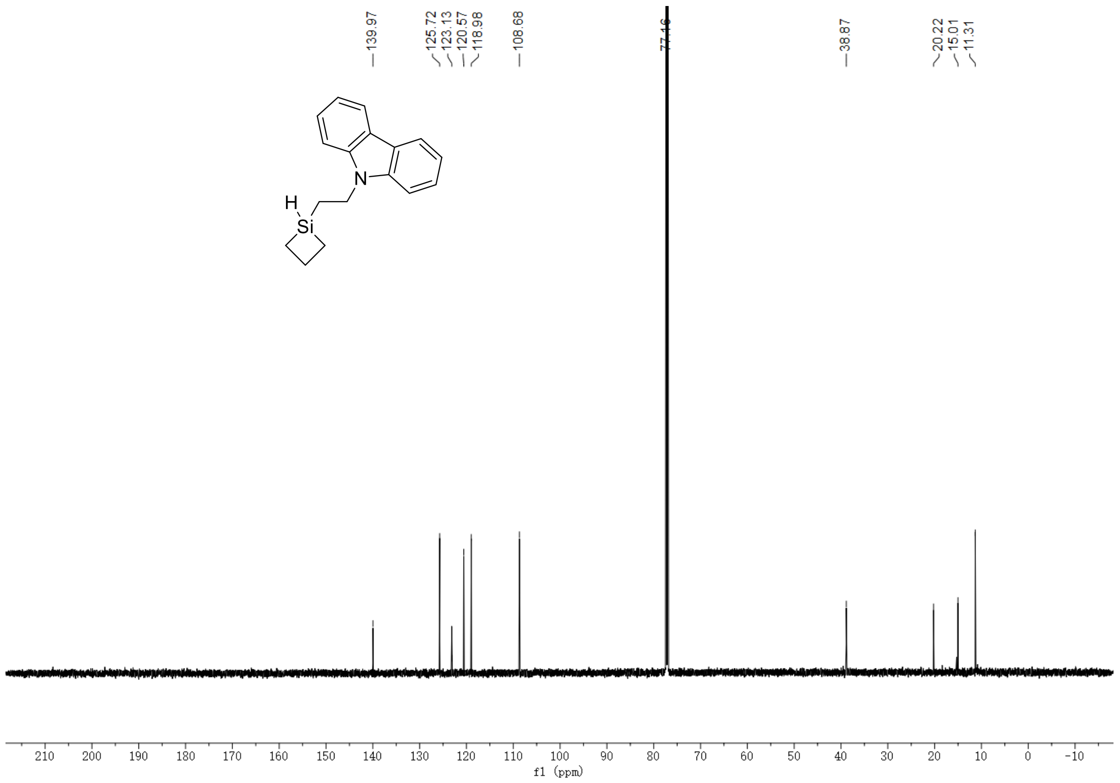

^{29}Si NMR of **3am** (119 MHz, $CDCl_3$, 25 ºC)

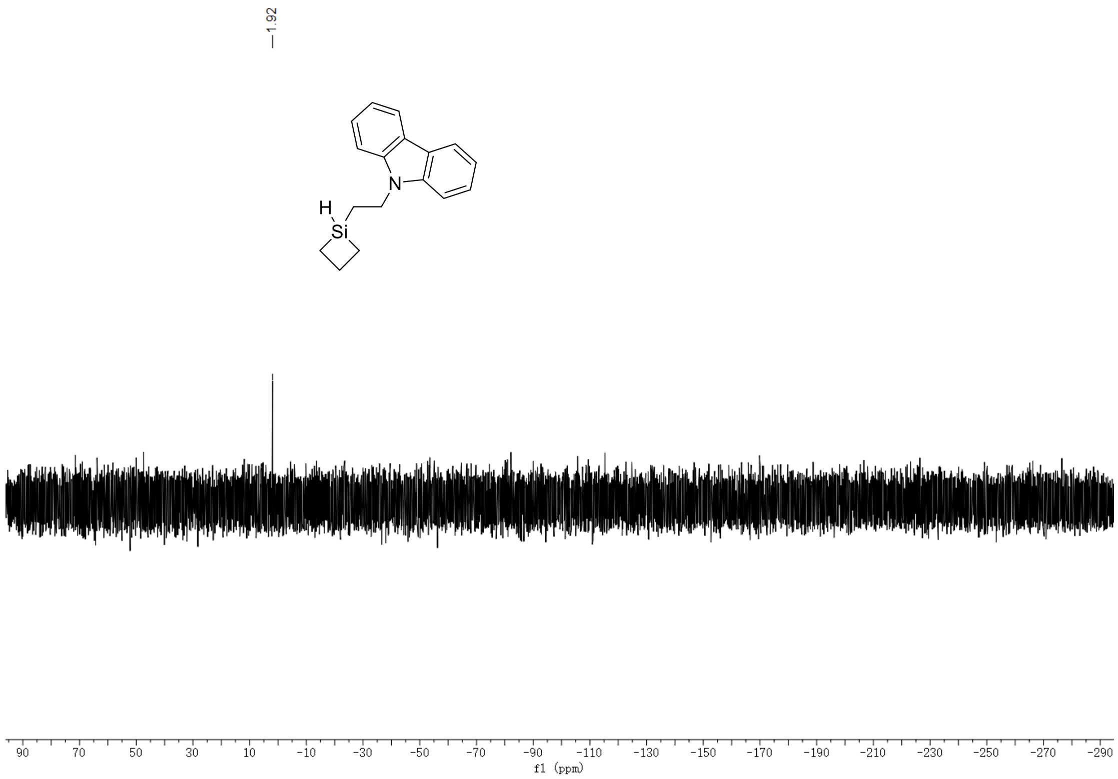

^{1}H NMR of **3an** (400 MHz, $CDCl_3$, 25 ºC)

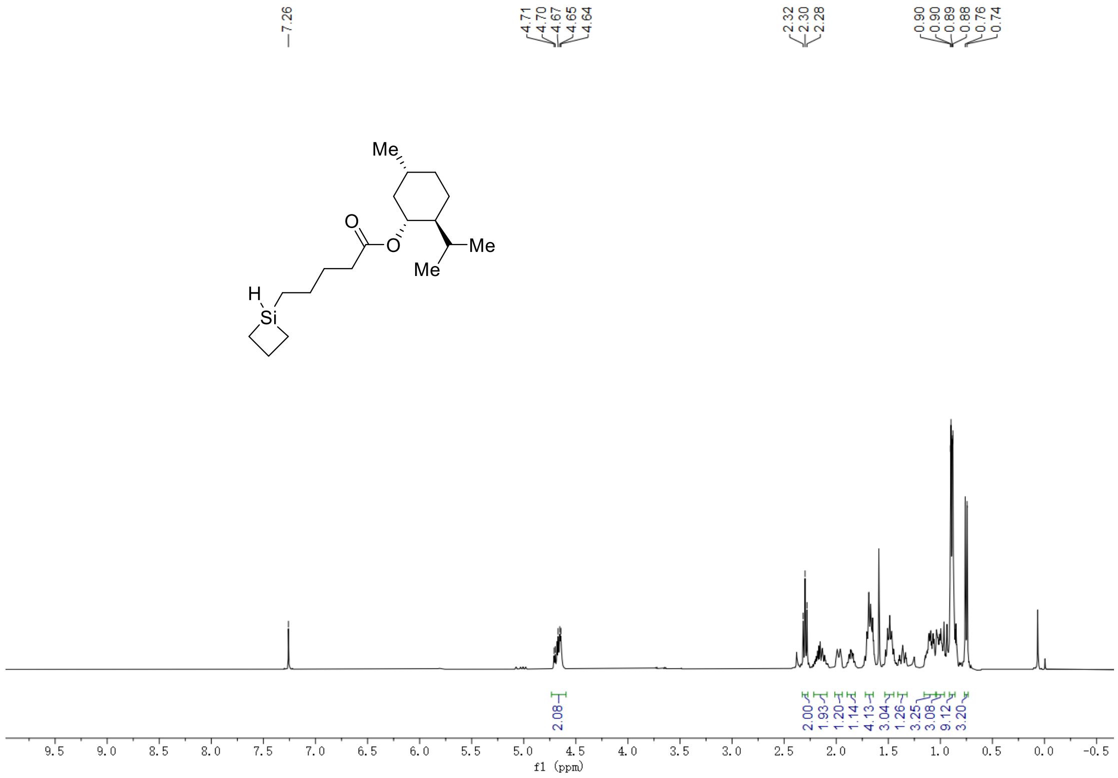

^{13}C NMR of **3an** (151 MHz, $CDCl_3$, 25 ºC)

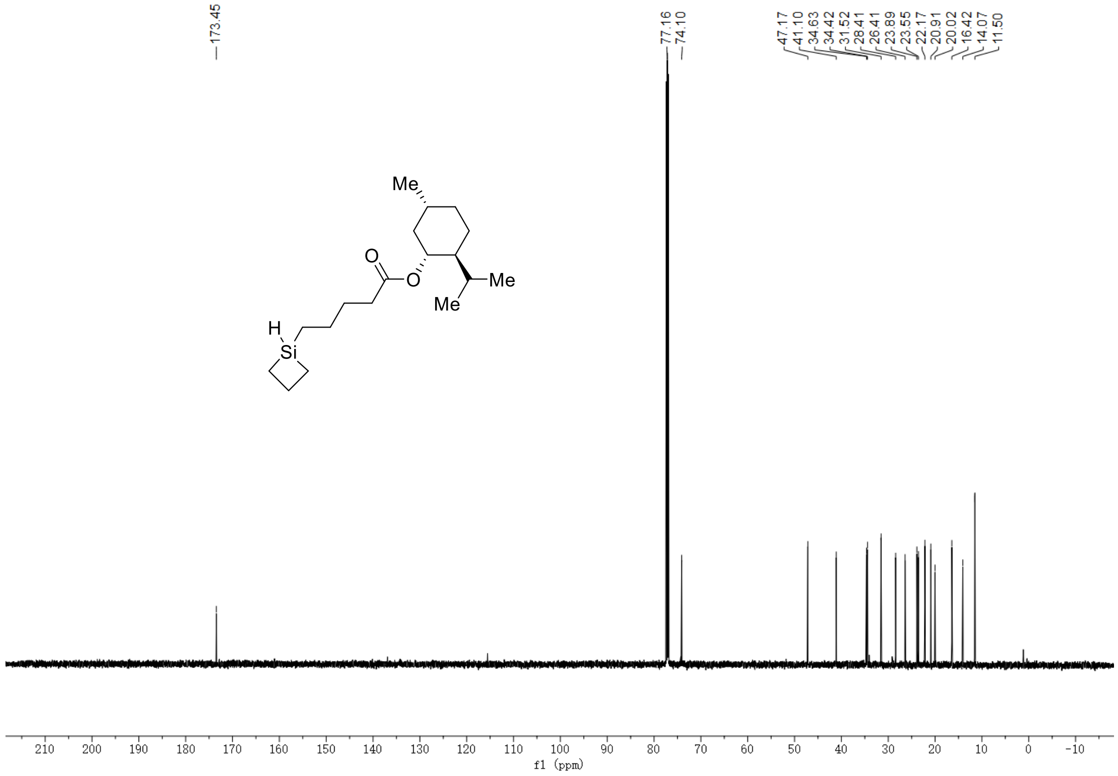

^{29}Si NMR of **3an** (119 MHz, $CDCl_3$, 25 ºC)

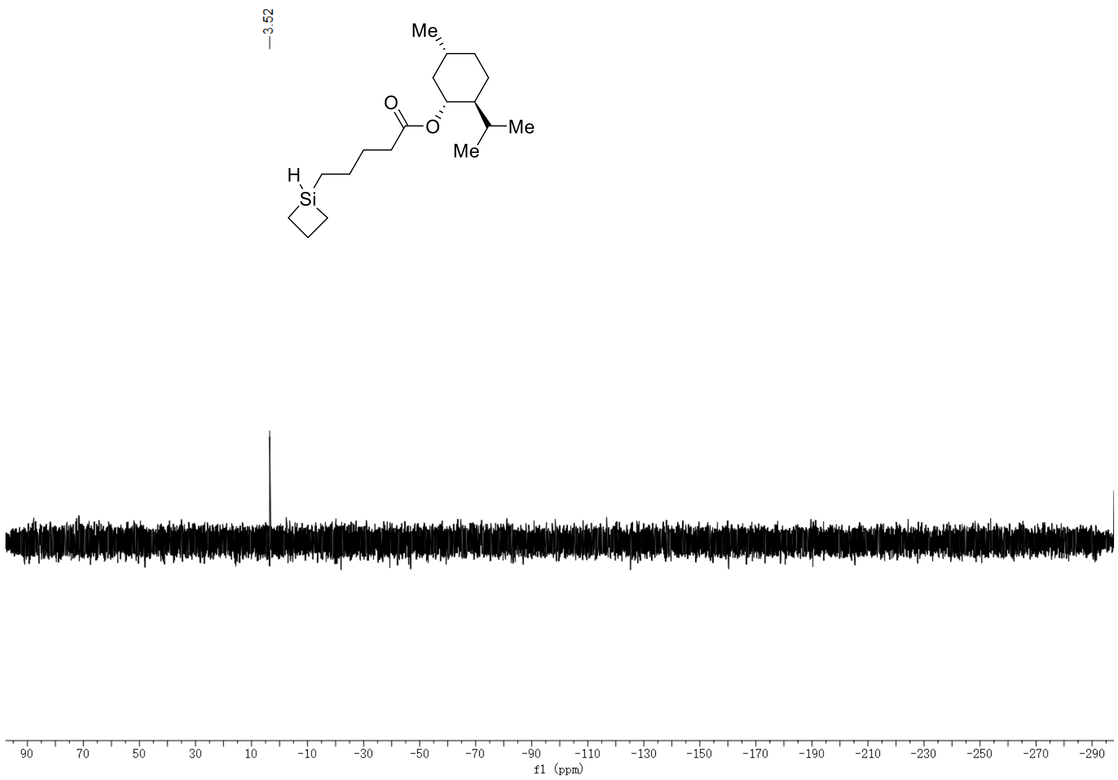

^{1}H NMR of **3ao** (600 MHz, $CDCl_3$, 25 ºC)

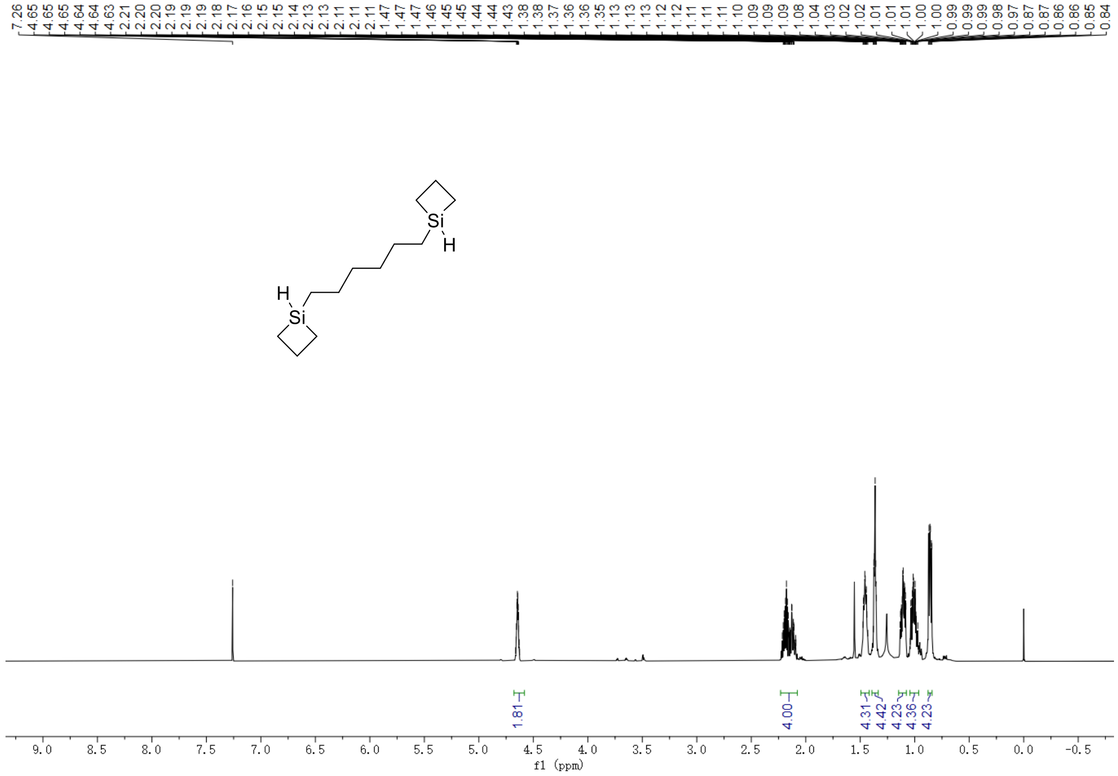

^{13}C NMR of **3ao** (151 MHz, $CDCl_3$, 25 ºC)

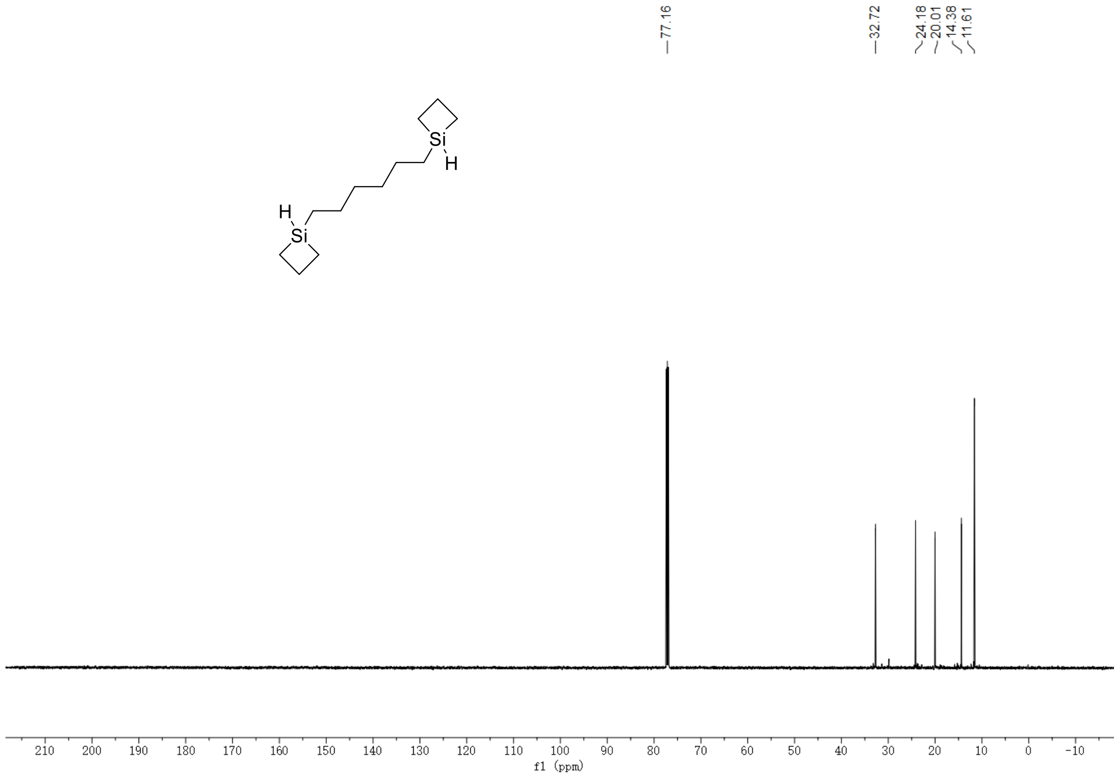

^{29}Si NMR of **3ao** (119 MHz, $CDCl_3$, 25 ºC)

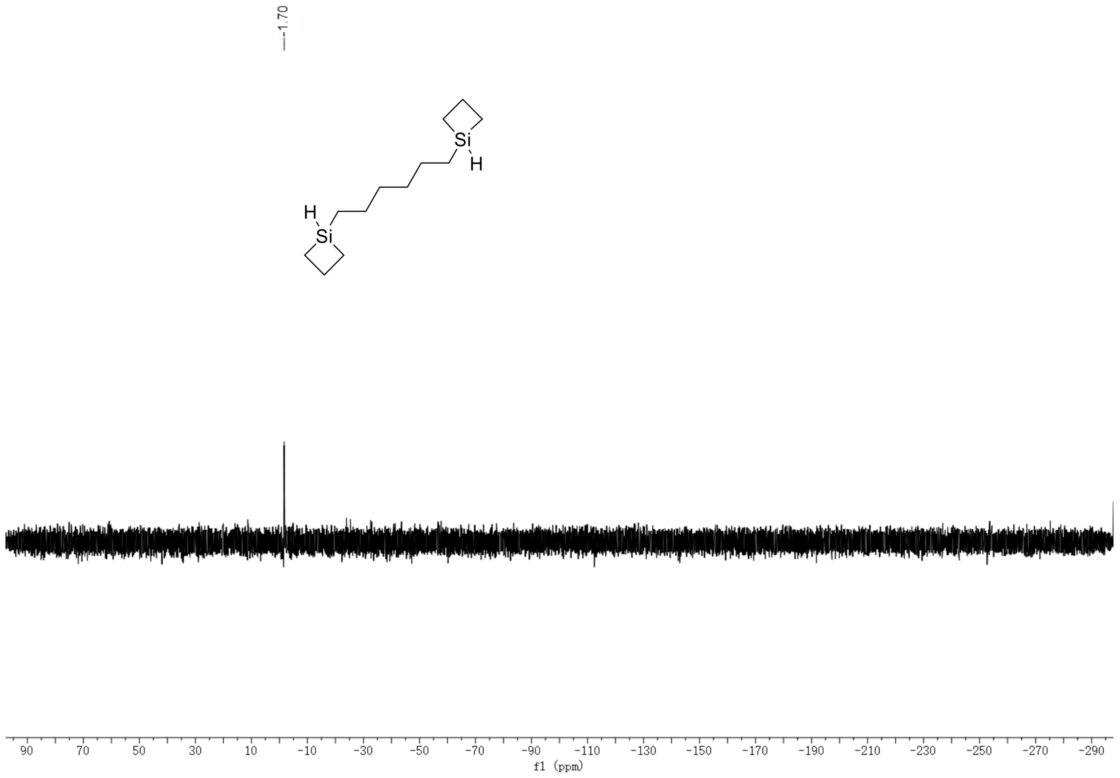

^{1}H NMR of **3ap** (400 MHz, $CDCl_3$, 25 ºC)

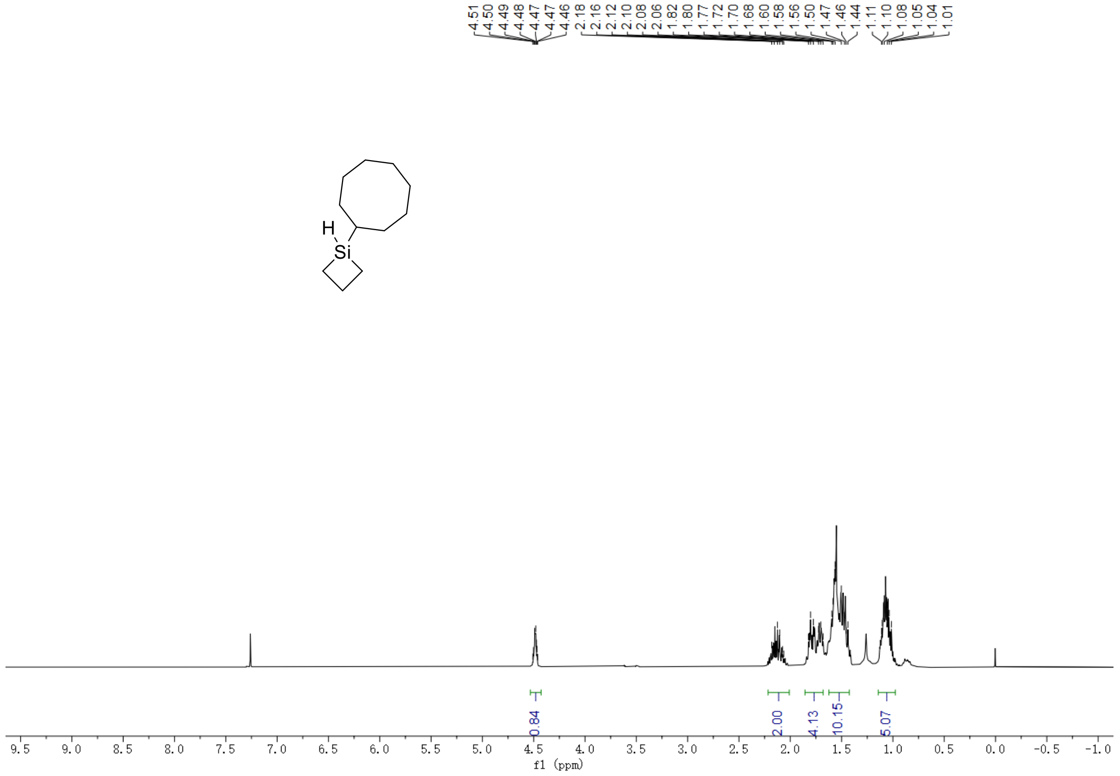

^{13}C NMR of **3ap** (151 MHz, $CDCl_3$, 25 ºC)

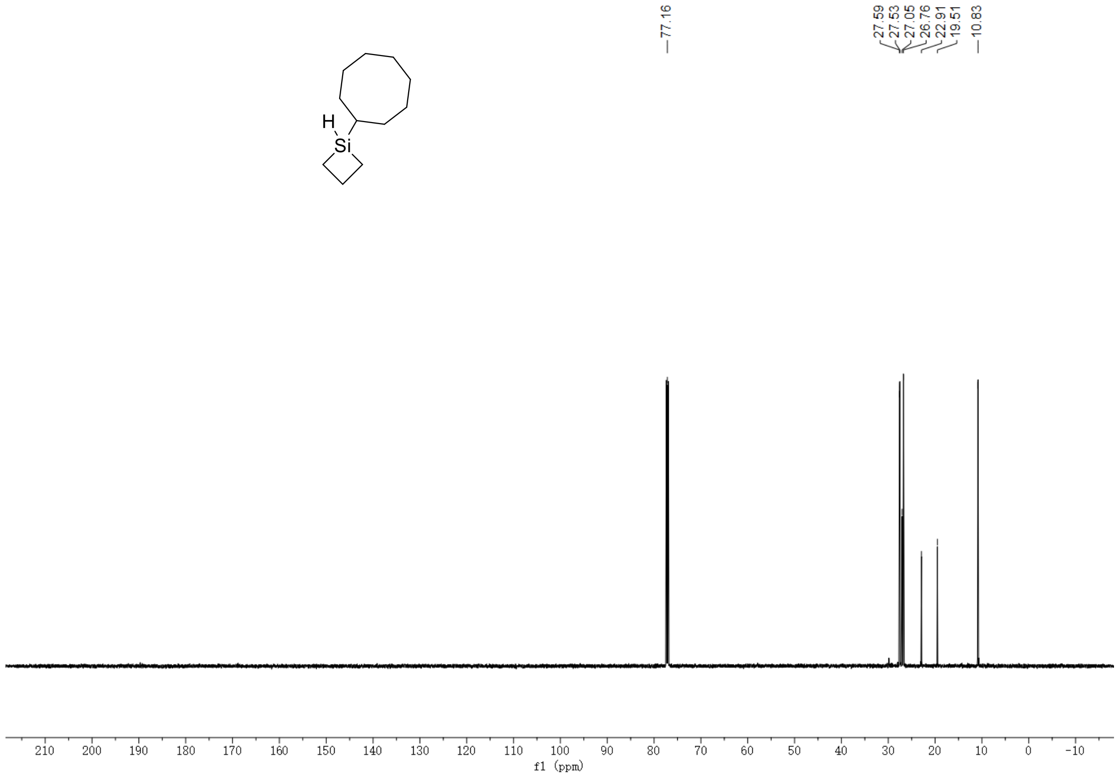

^{29}Si NMR of **3ap** (119 MHz, $CDCl_3$, 25 ºC)

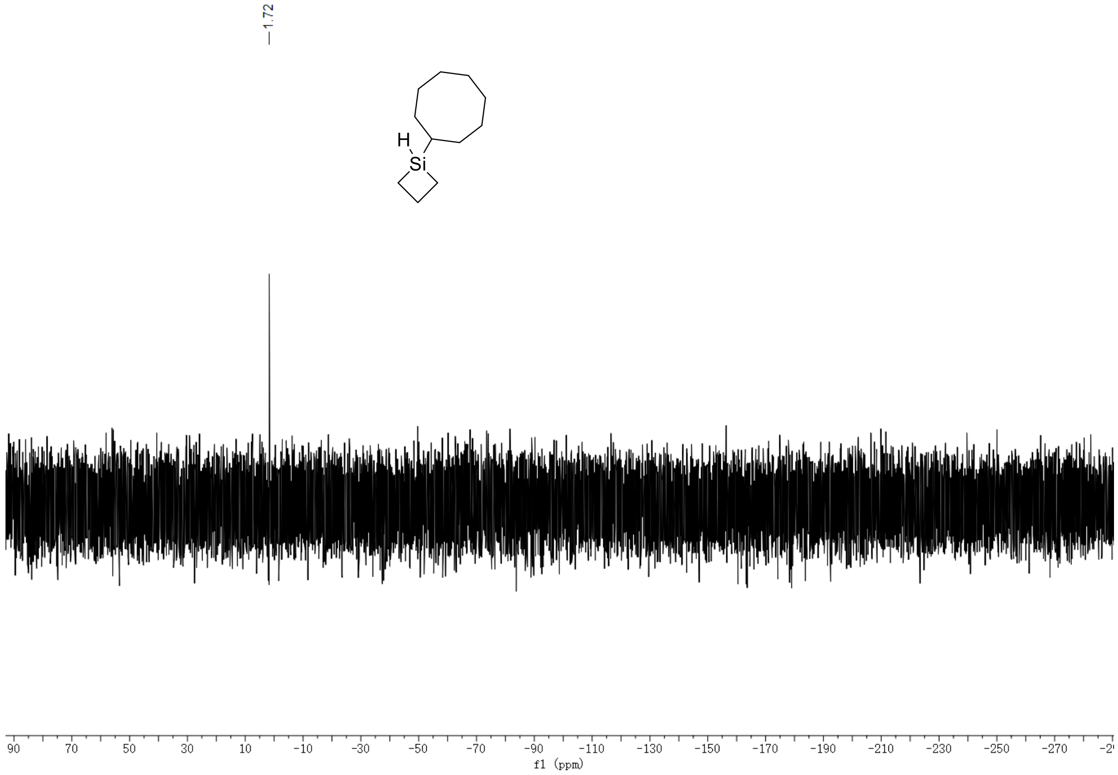

^{1}H NMR of **3aq** (600 MHz, $CDCl_3$, 25 ºC)

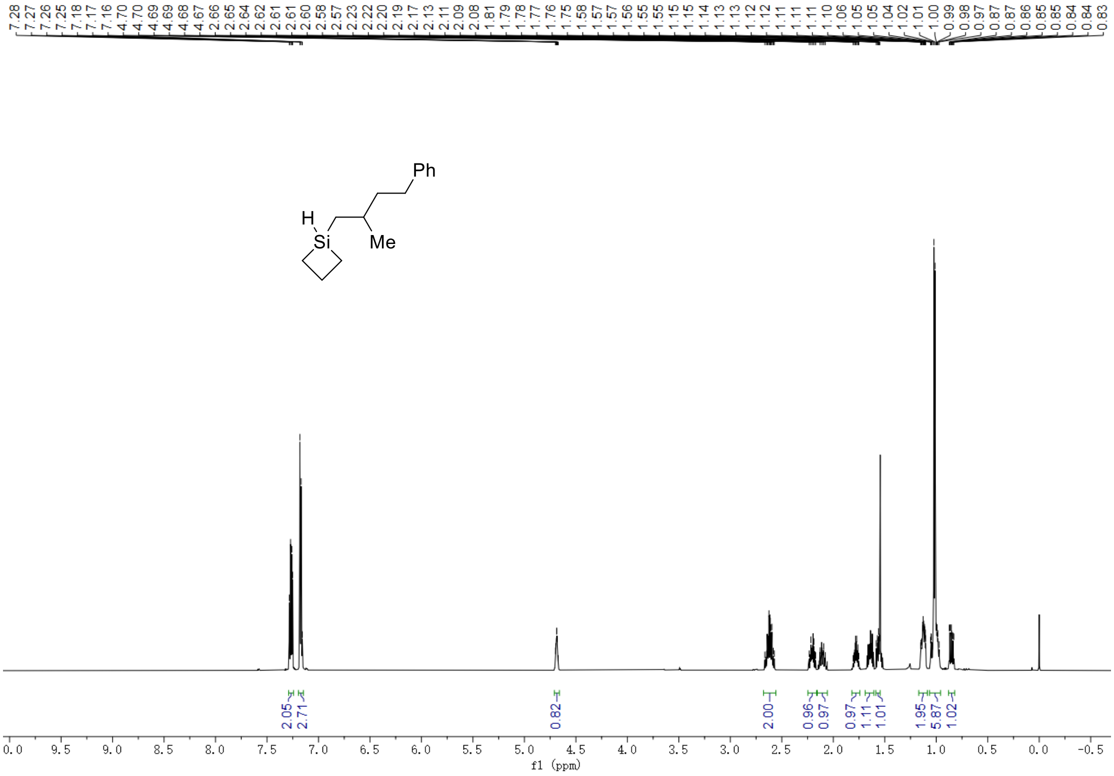

^{13}C NMR of **3aq** (151 MHz, $CDCl_3$, 25 ºC)

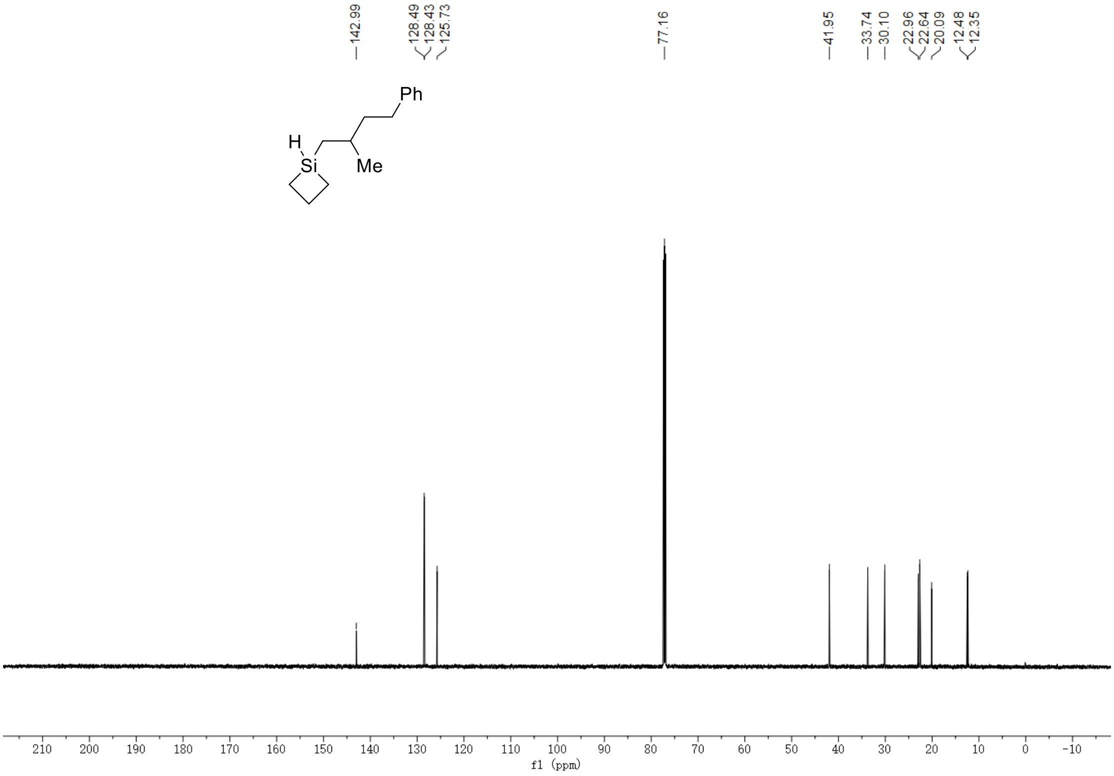

^{29}Si NMR of **3aq** (119 MHz, $CDCl_3$, 25 ºC)

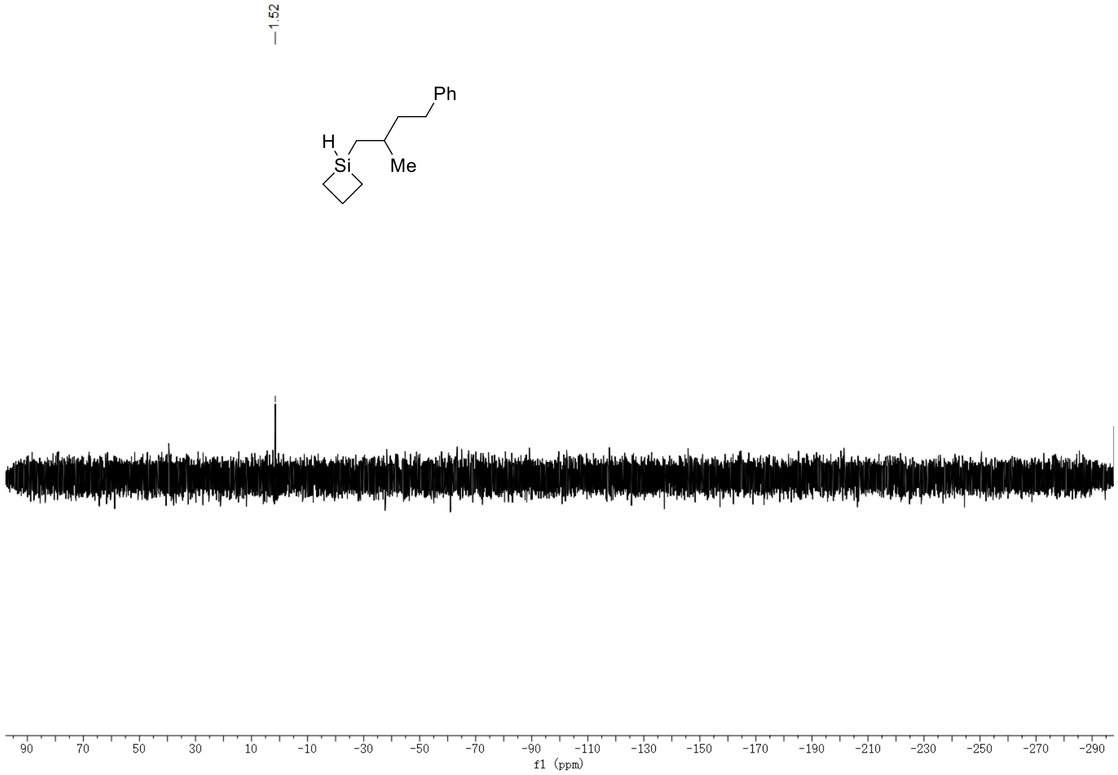

^{1}H NMR of **4aa** (600 MHz, $CDCl_3$, 25 ºC)

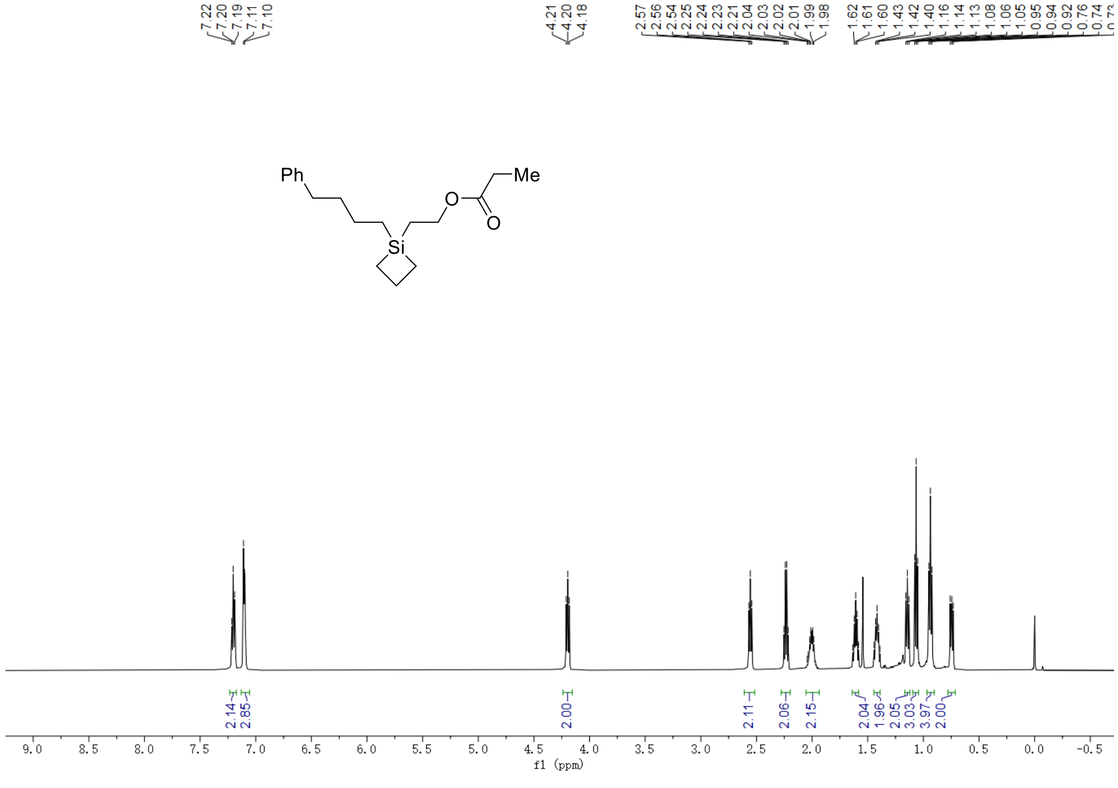

^{13}C NMR of **4aa** (151 MHz, $CDCl_3$, 25 ºC)

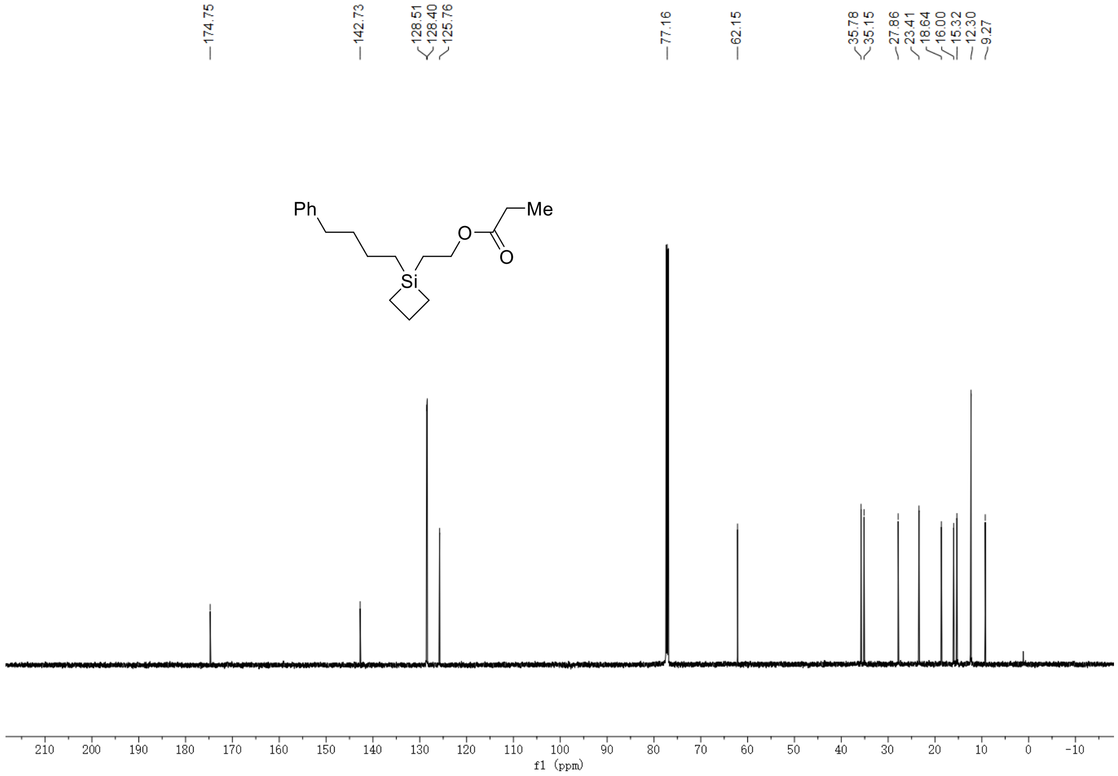

^{29}Si NMR of **4aa** (119 MHz, $CDCl_3$, 25 ºC)

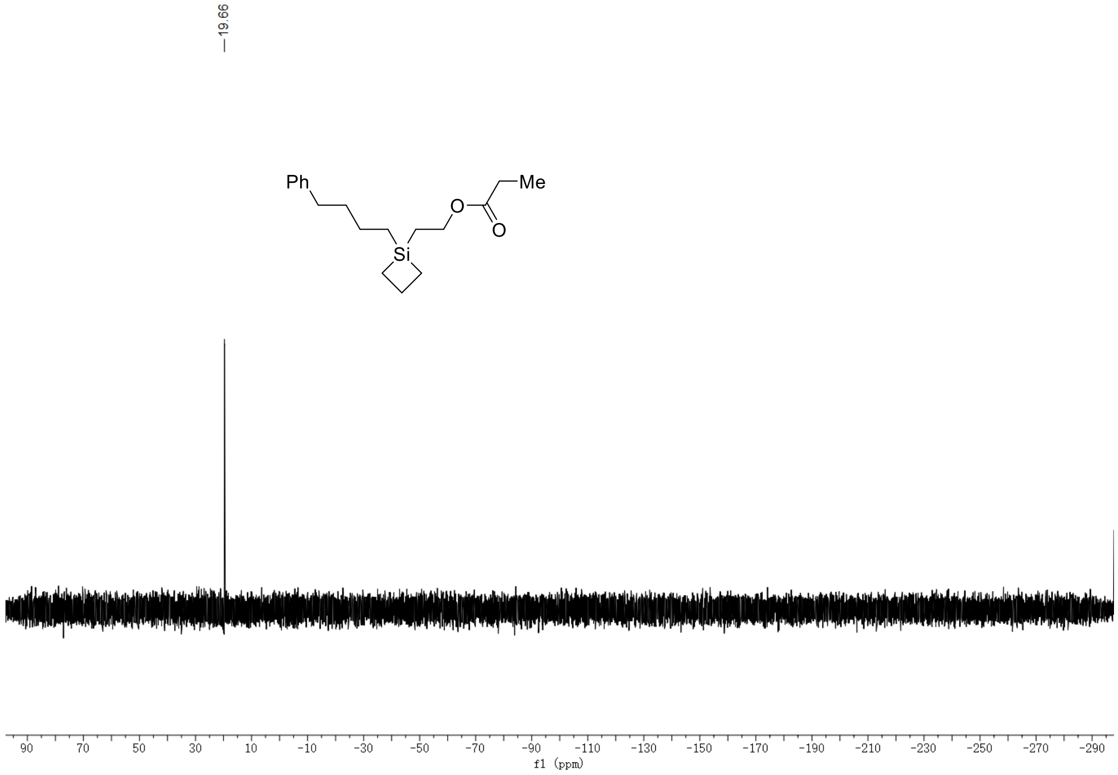

^{1}H NMR of **4ab** (600 MHz, $CDCl_3$, 25 ºC)

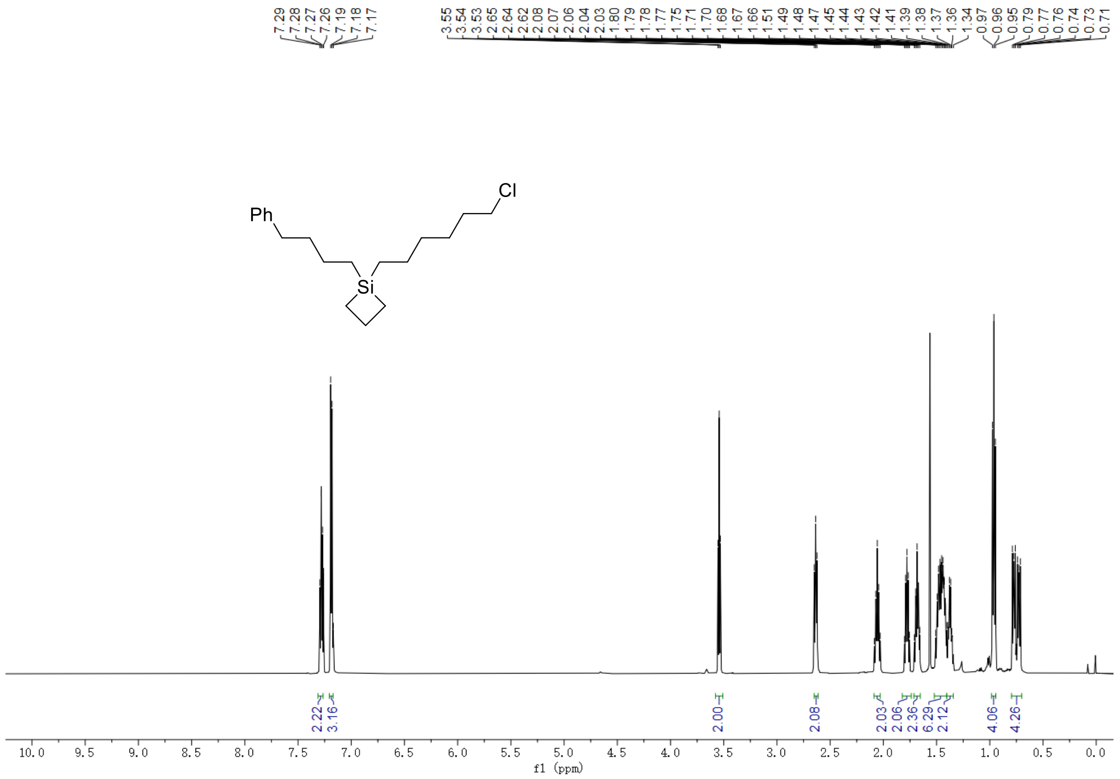

^{13}C NMR of **4ab** (151 MHz, $CDCl_3$, 25 ºC)

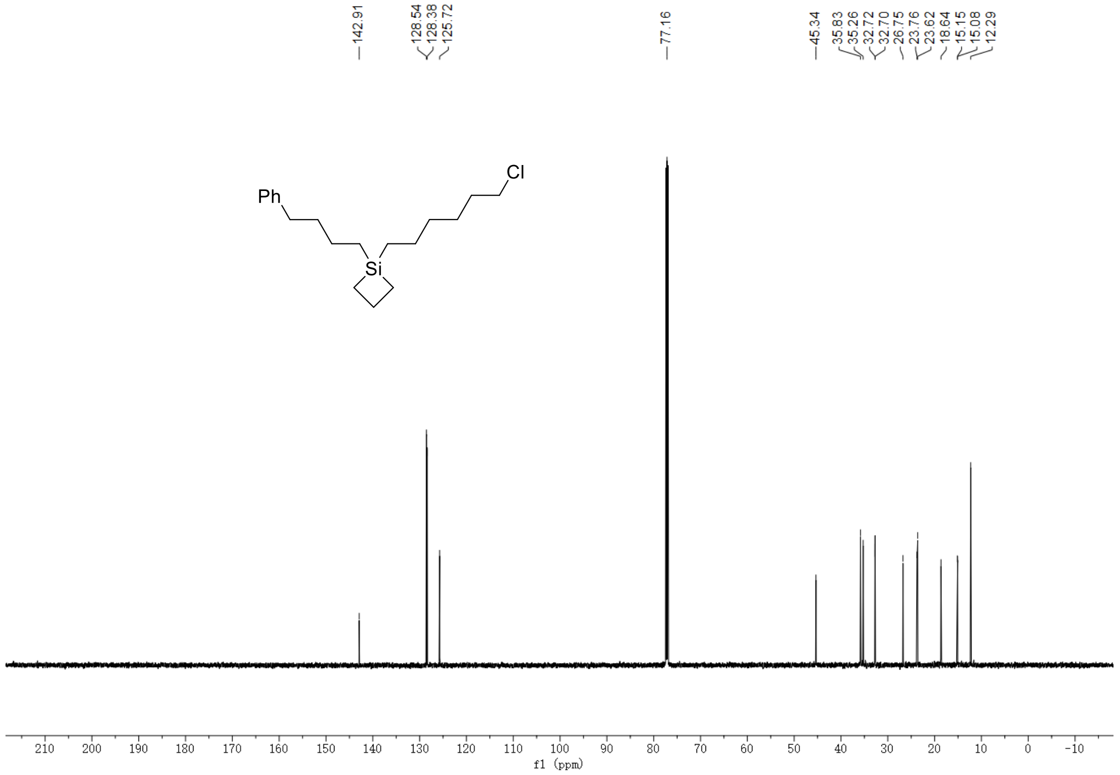

^{29}Si NMR of **4ab** (119 MHz, $CDCl_3$, 25 ºC)

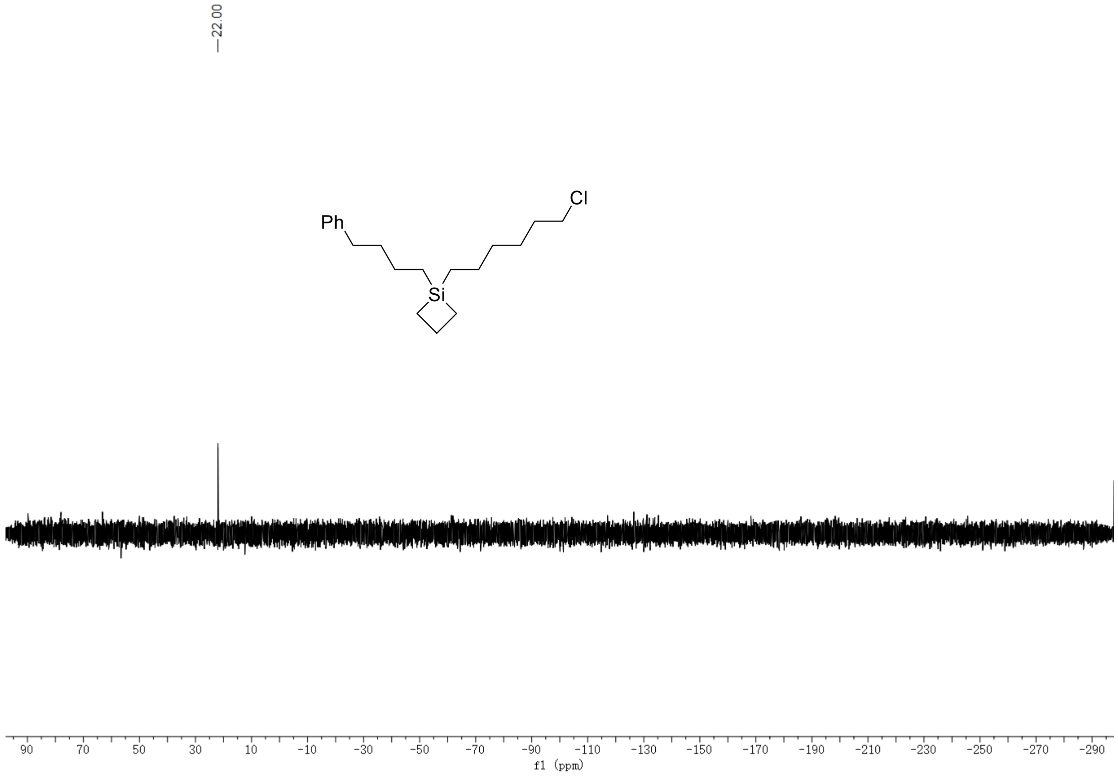

^{1}H NMR of **4ac** (400 MHz, $CDCl_3$, 25 ºC)

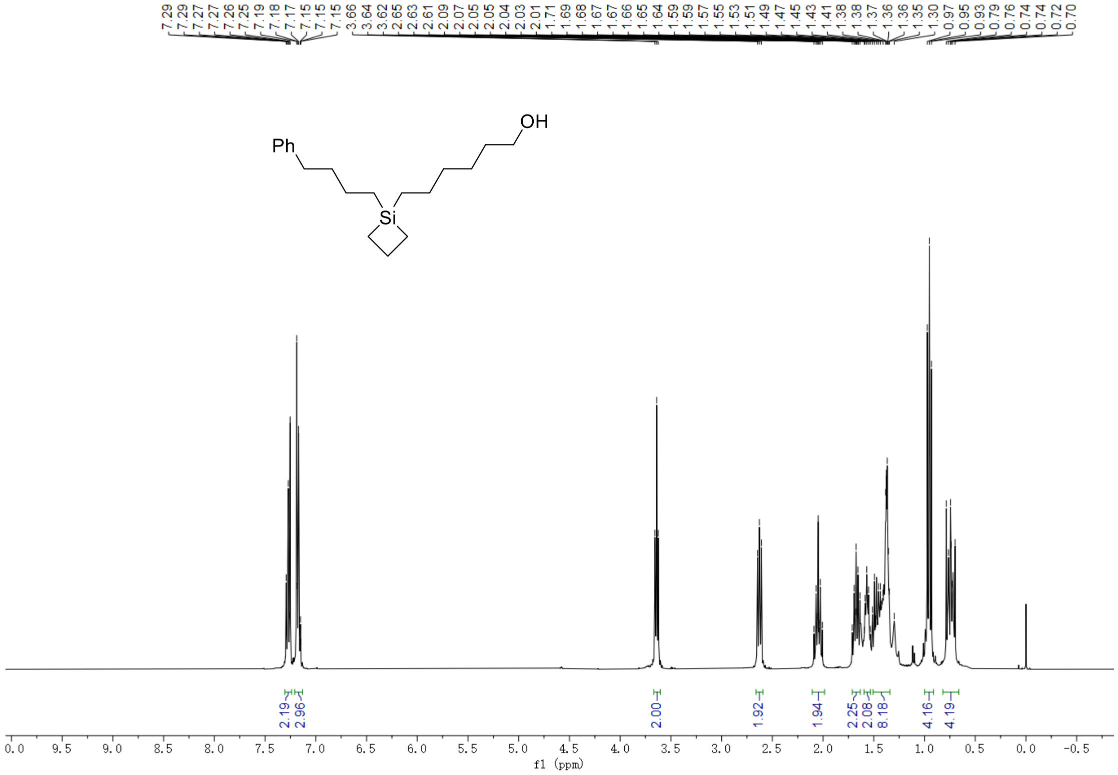

^{13}C NMR of **4ac** (151 MHz, $CDCl_3$, 25 ºC)

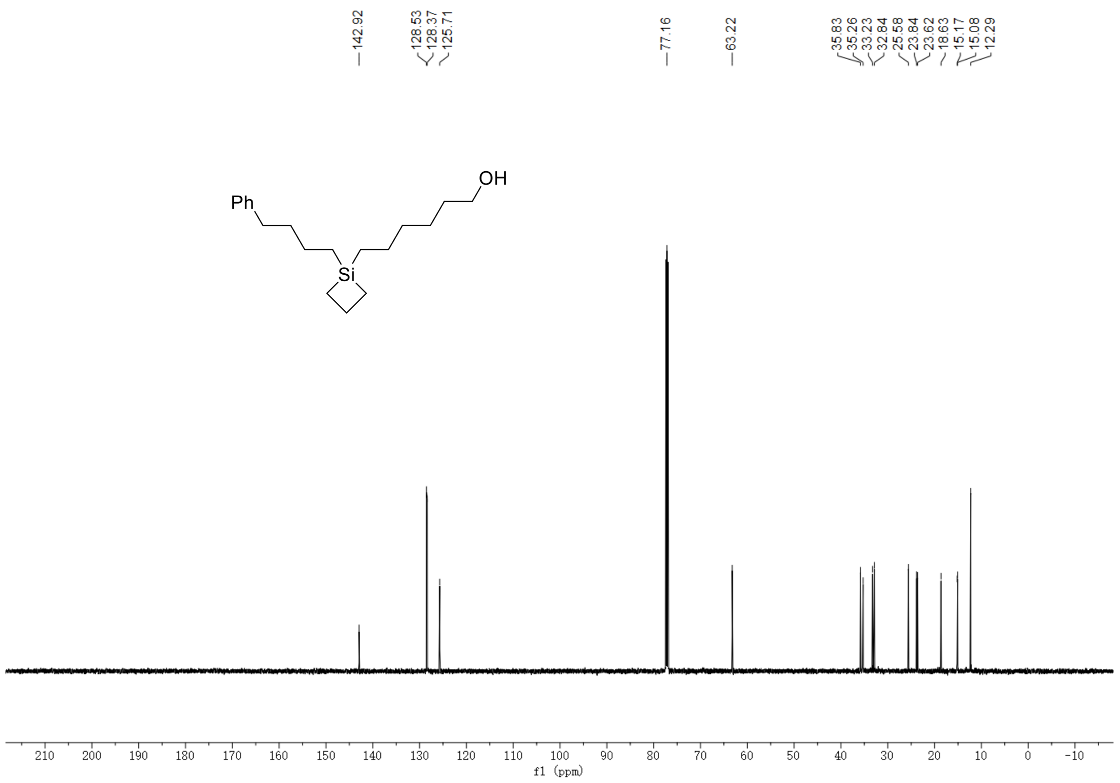

^{29}Si NMR of **4ac** (119 MHz, $CDCl_3$, 25 ºC)

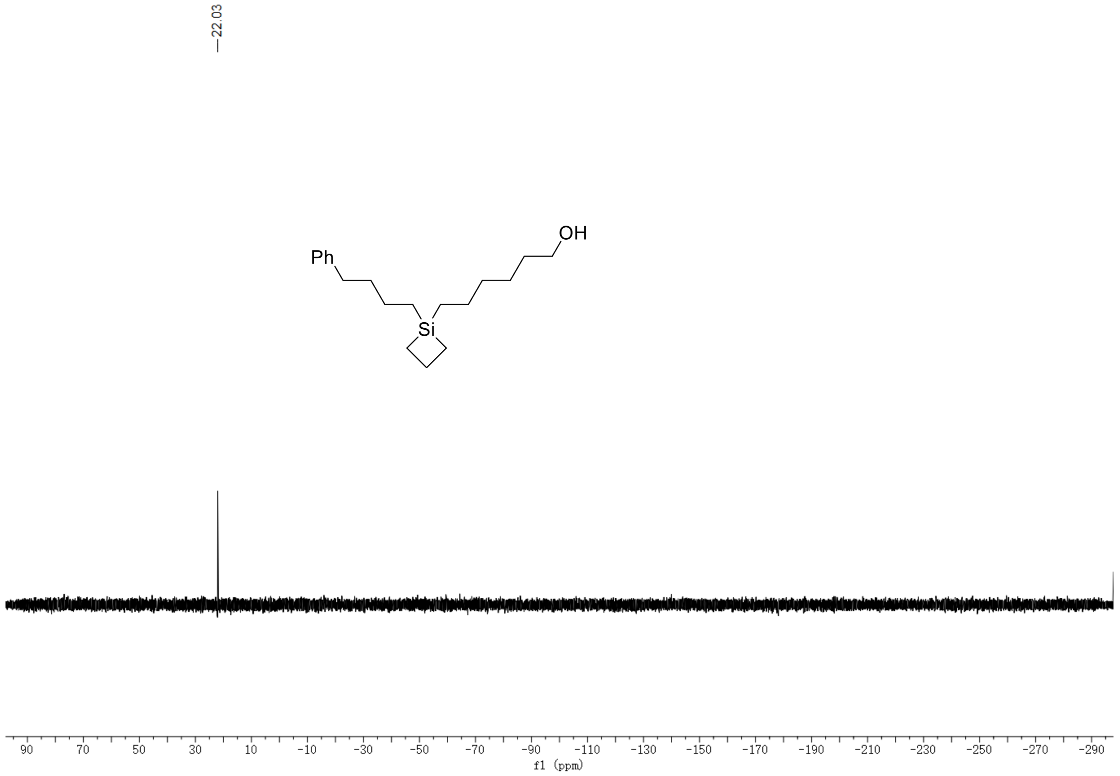

^{1}H NMR of **4ad** (600 MHz, $CDCl_3$, 25 ºC)

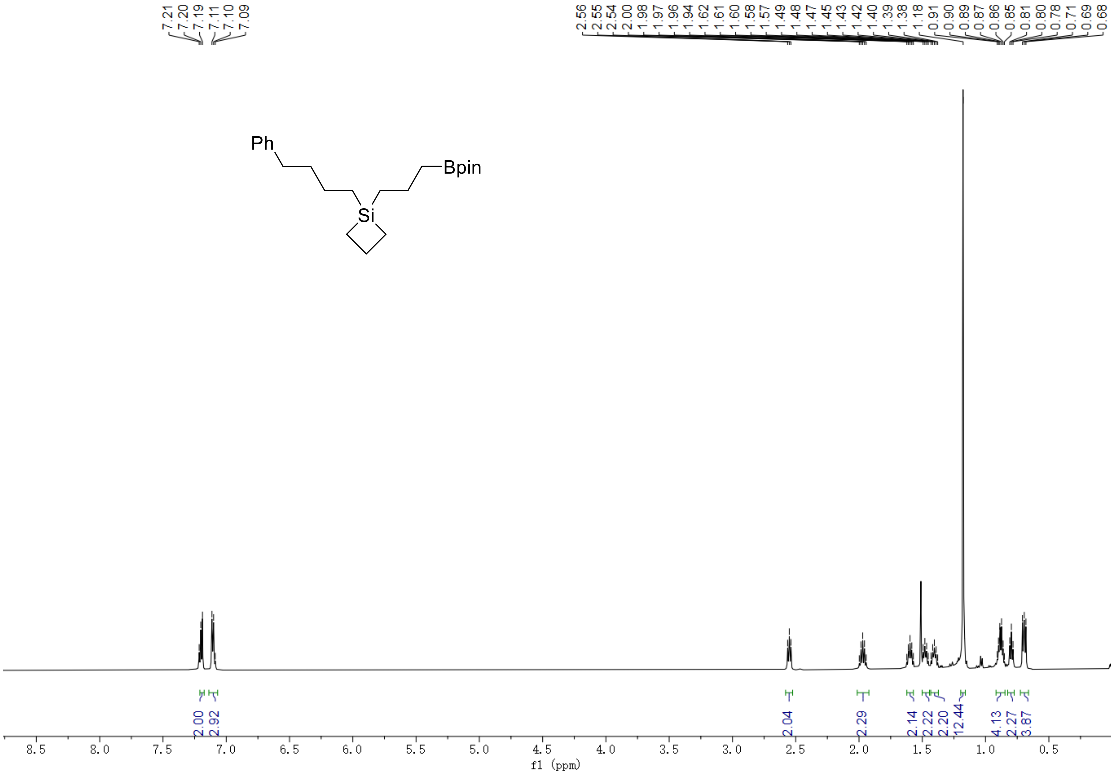

^{13}C NMR of **4ad** (151 MHz, $CDCl_3$, 25 ºC)

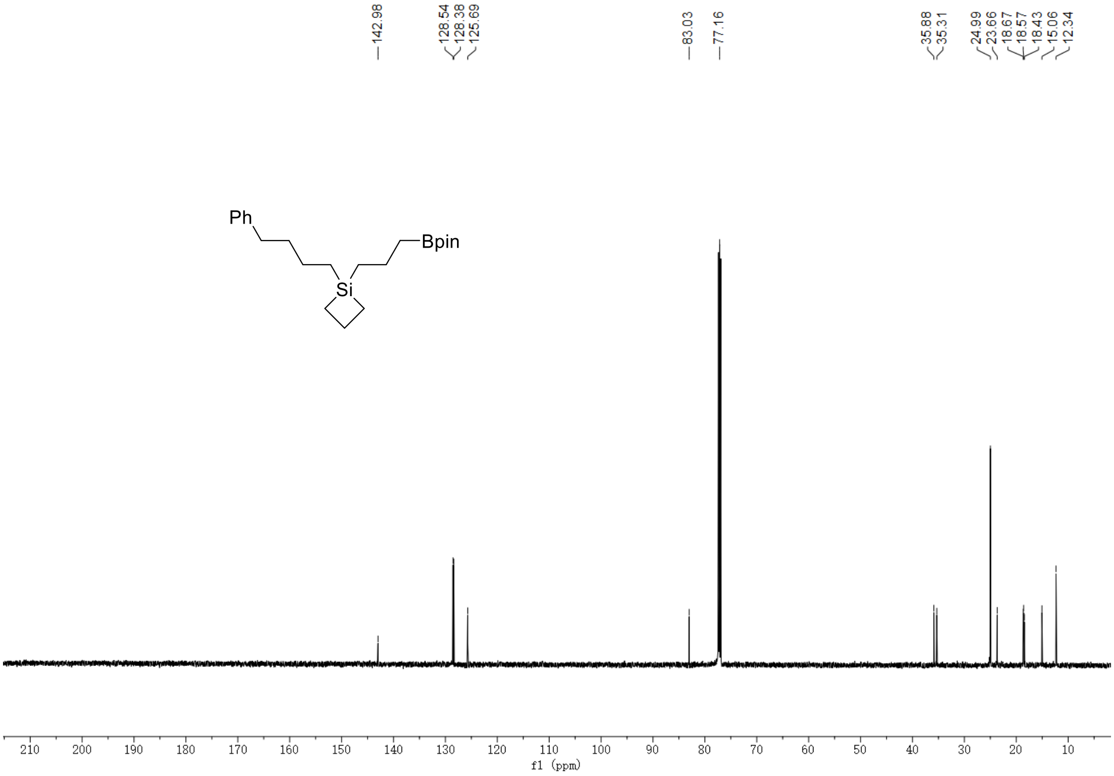

^{29}Si NMR of **4ad** (119 MHz, $CDCl_3$, 25 ºC)

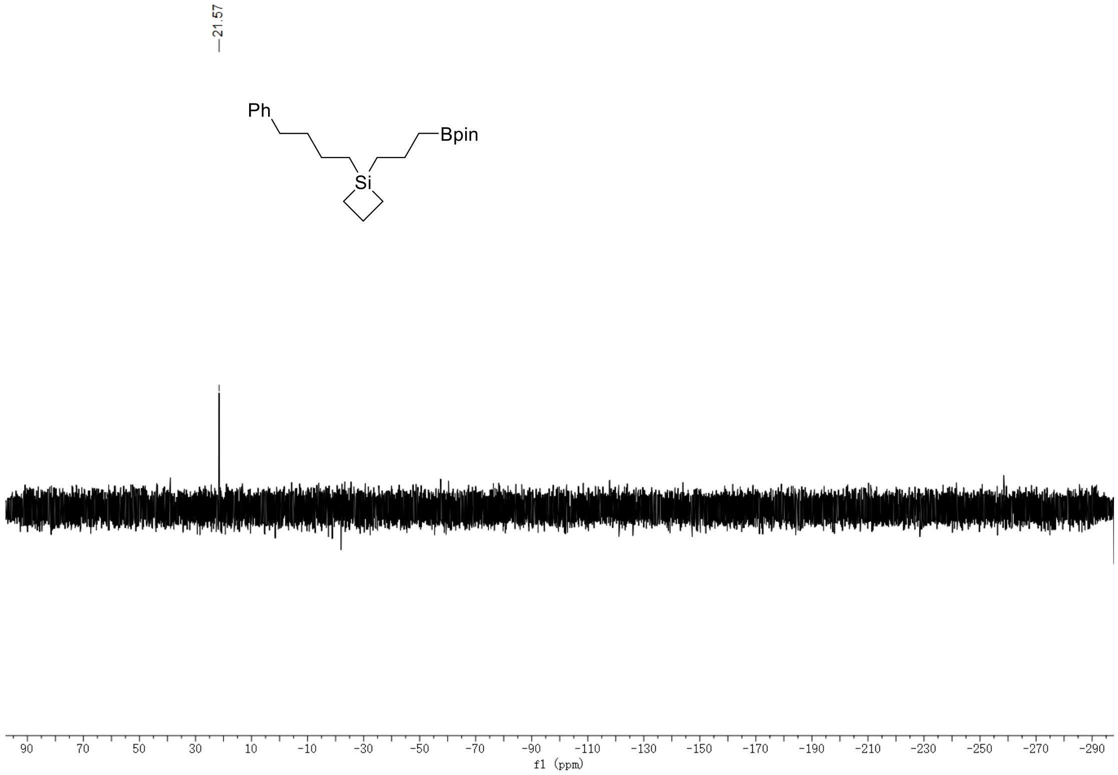

^{1}H NMR of **4ae** (400 MHz, $CDCl_3$, 25 ºC)

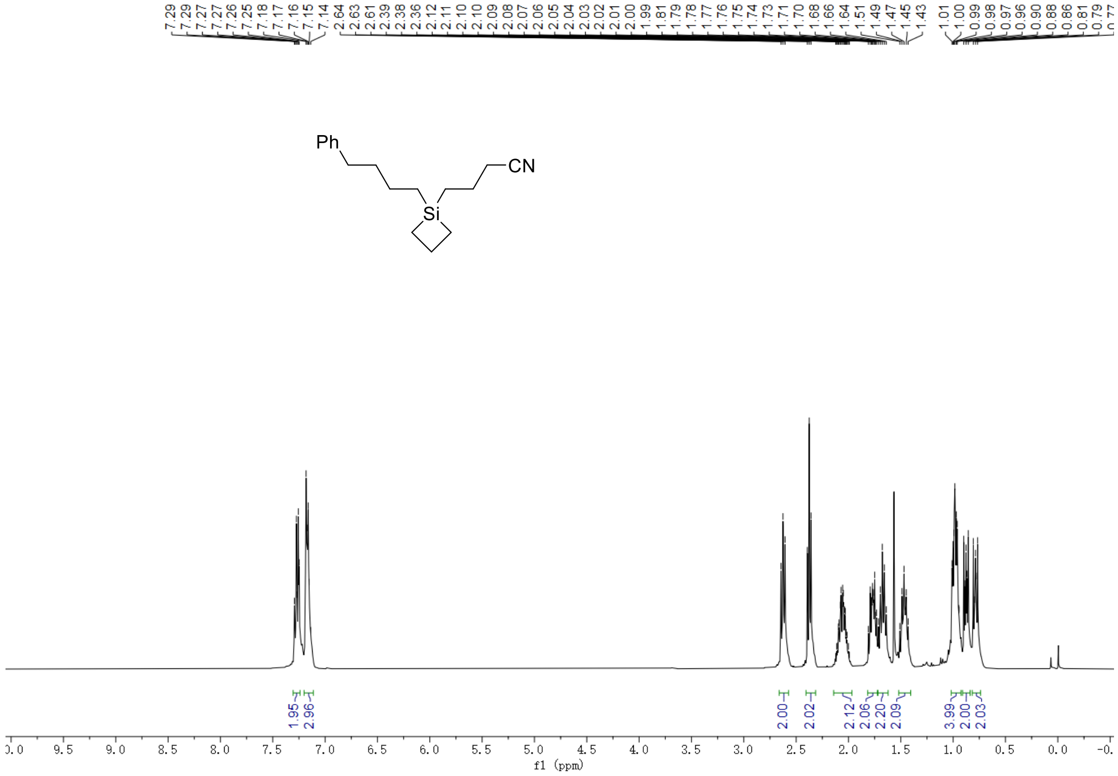

^{13}C NMR of **4ae** (151 MHz, $CDCl_3$, 25 ºC)

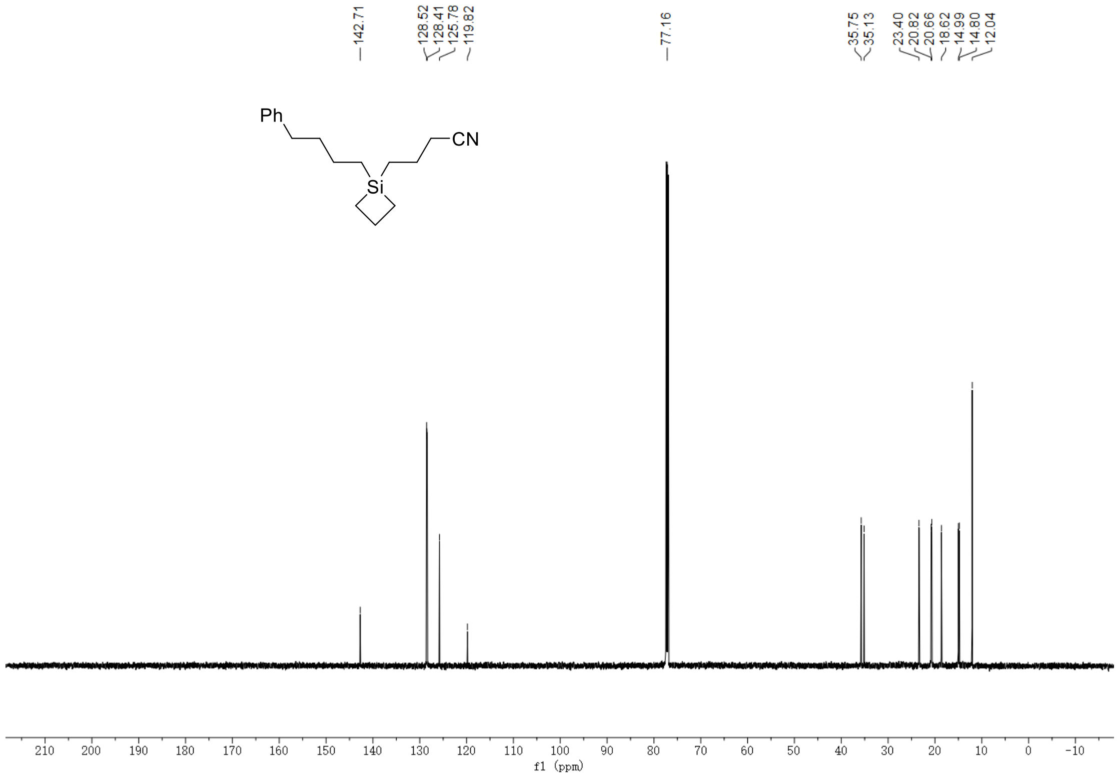

^{29}Si NMR of **4ae** (119 MHz, $CDCl_3$, 25 ºC)

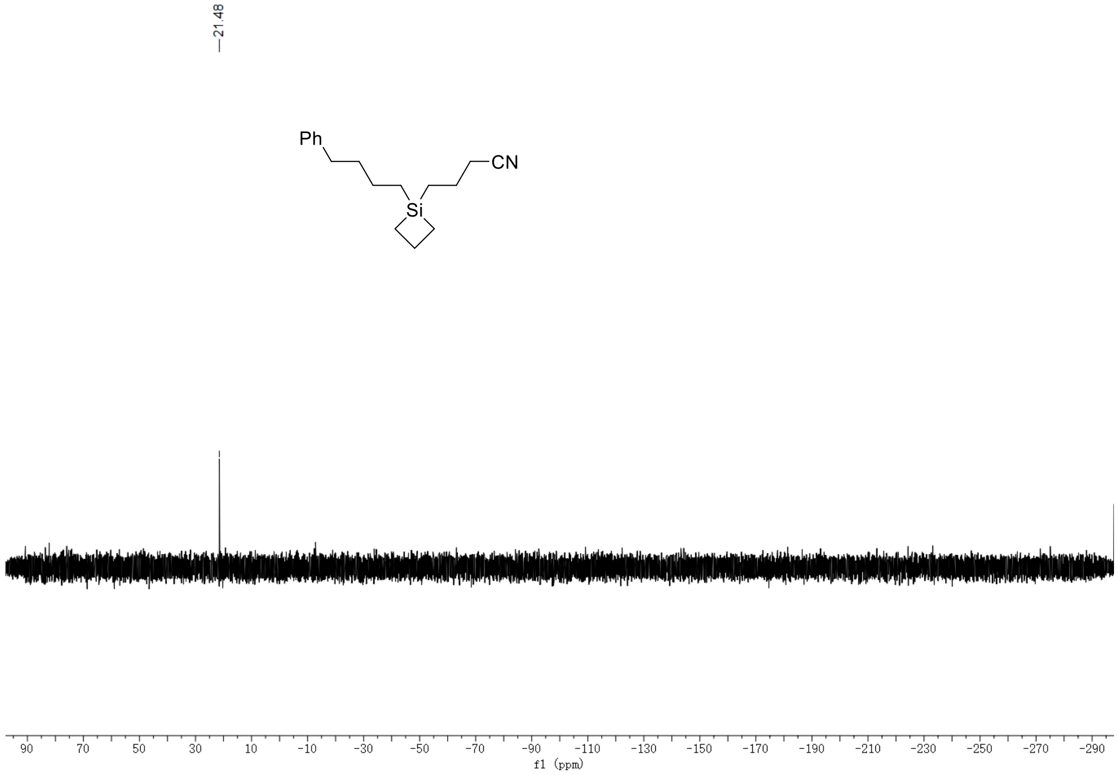

^{1}H NMR of **4af** (400 MHz, $CDCl_3$, 25 ºC)

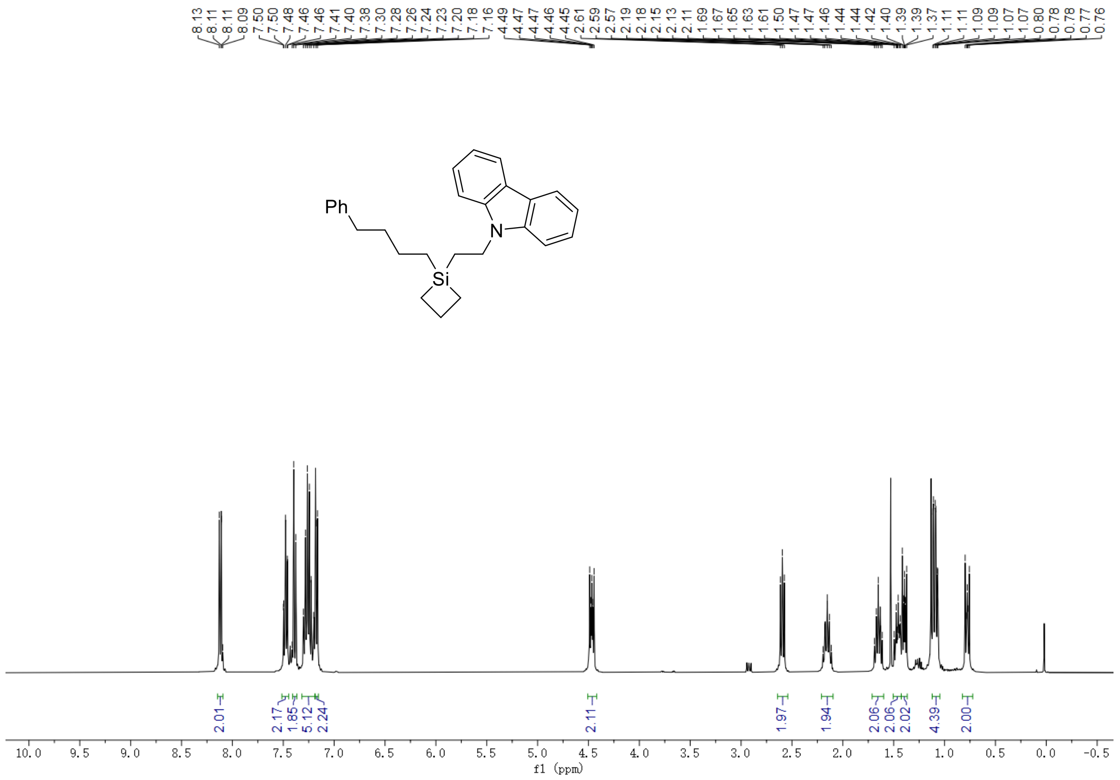

^{13}C NMR of **4af** (151 MHz, $CDCl_3$, 25 ºC)

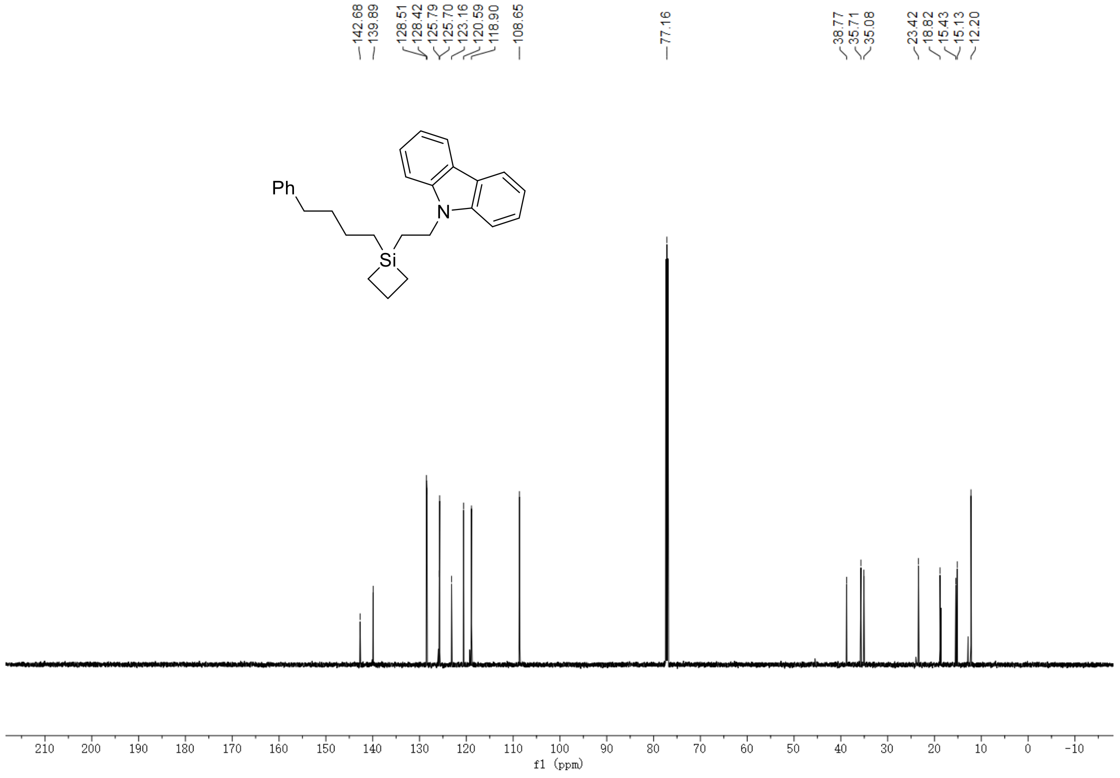

^{29}Si NMR of **4af** (119 MHz, $CDCl_3$, 25 ºC)

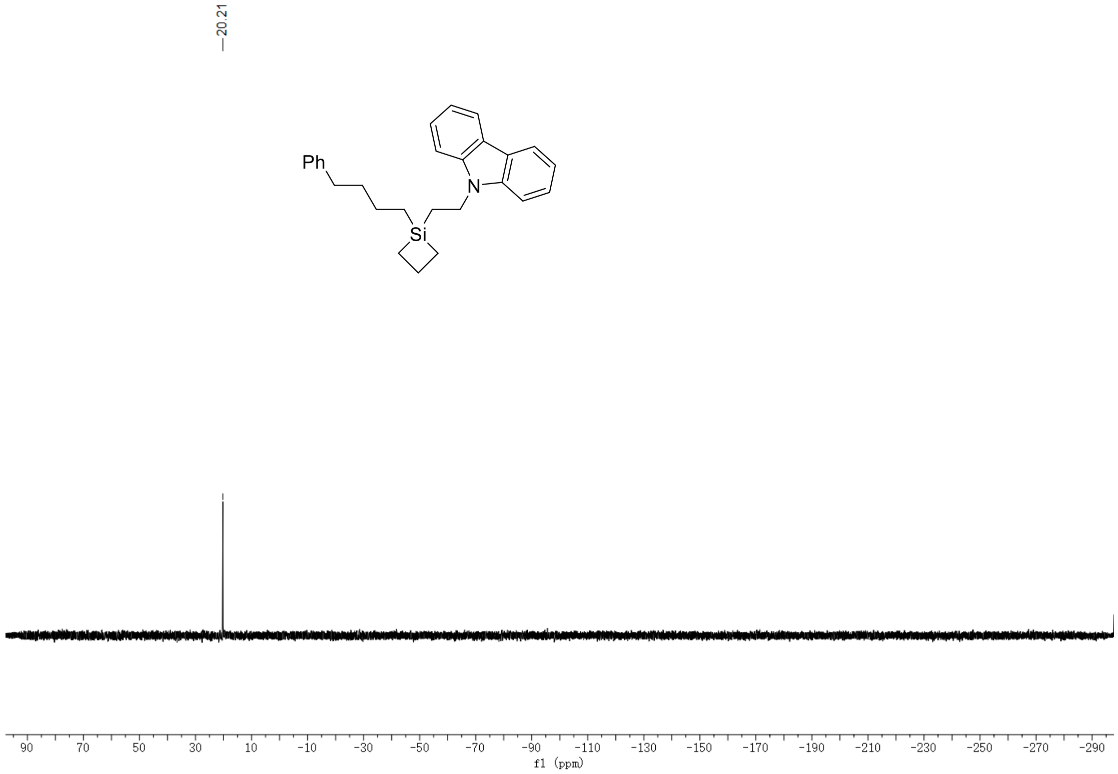

^{1}H NMR of **4ag** (400 MHz, $CDCl_3$, 25 ºC)

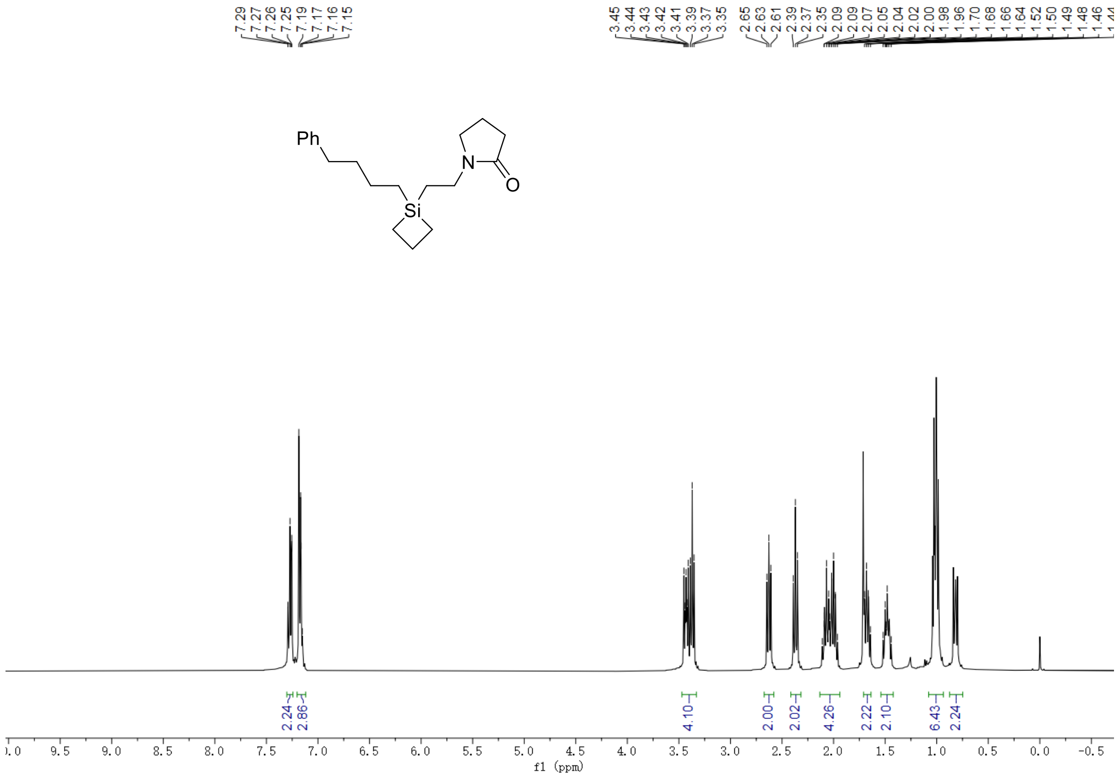

^{13}C NMR of **4ag** (151 MHz, $CDCl_3$, 25 ºC)

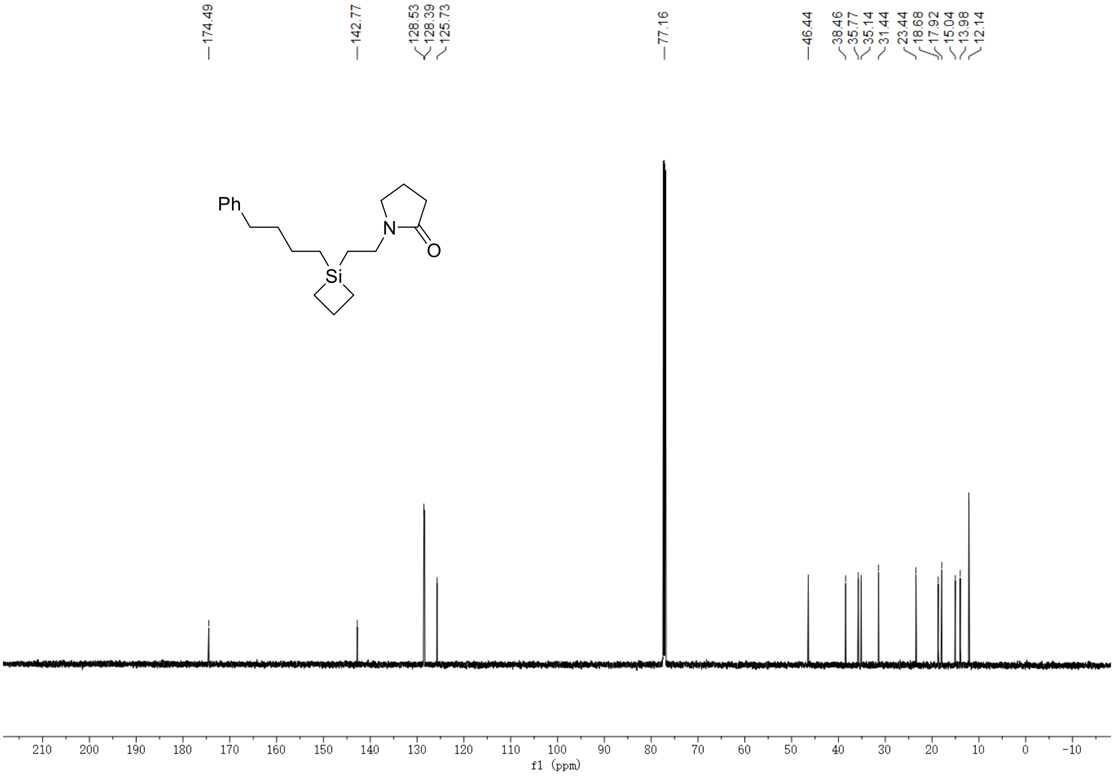

^{29}Si NMR of **4ag** (119 MHz, $CDCl_3$, 25 ºC)

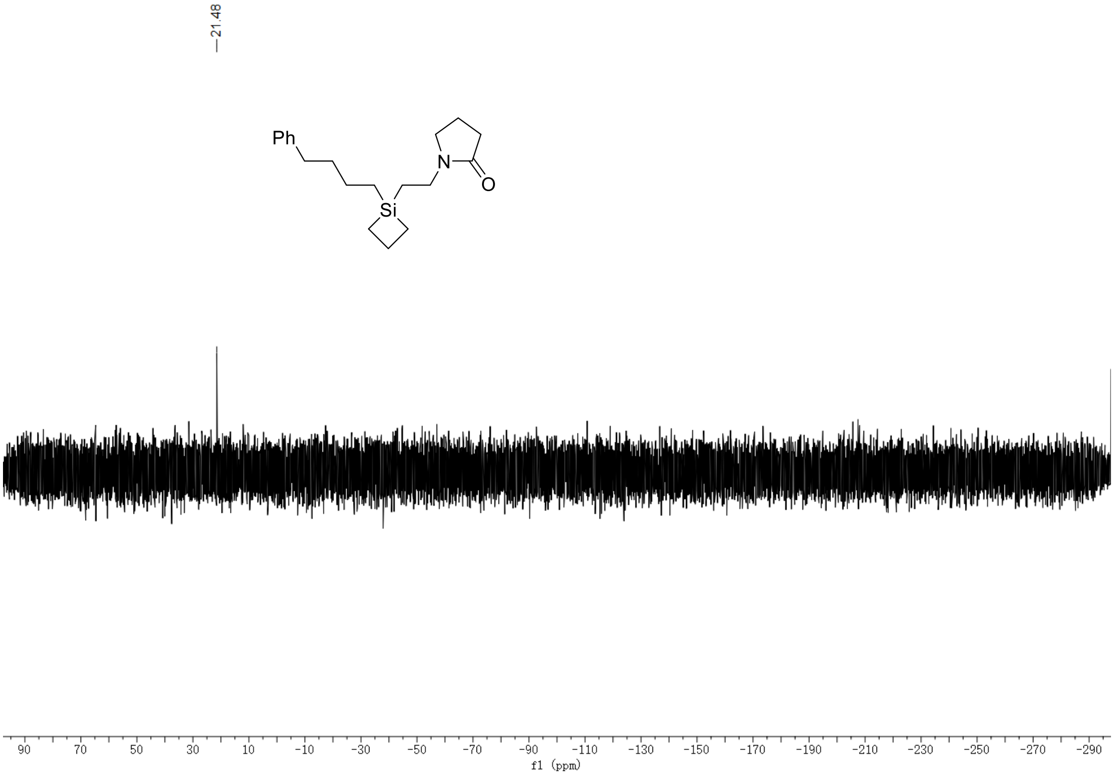

^{1}H NMR of **4ah** (600 MHz, $CDCl_3$, 25 ºC)

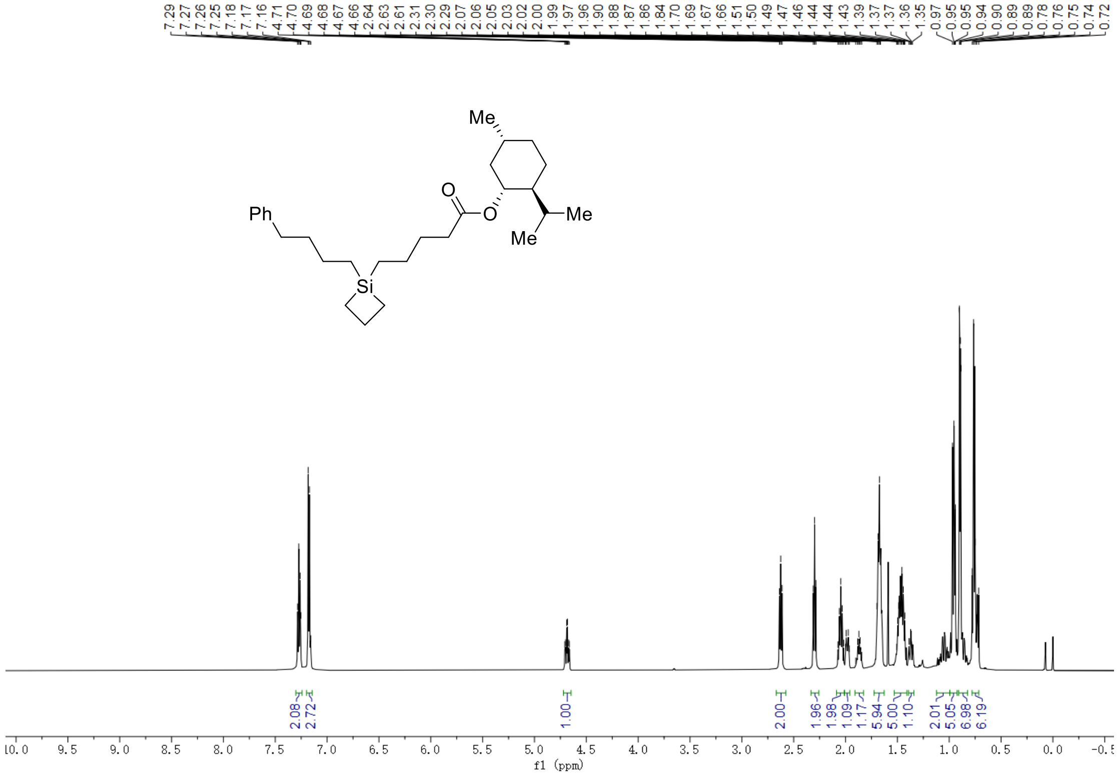

^{13}C NMR of **4ah** (151 MHz, $CDCl_3$, 25 ºC)

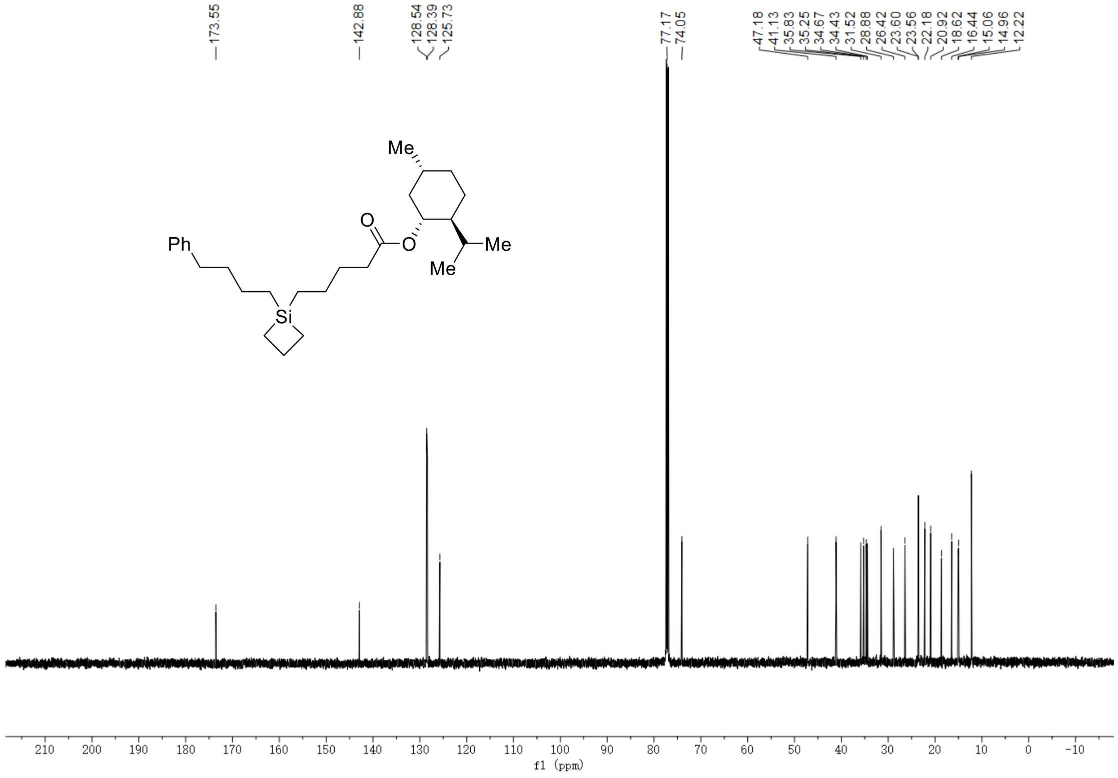

^{29}Si NMR of **4ah** (119 MHz, $CDCl_3$, 25 ºC)

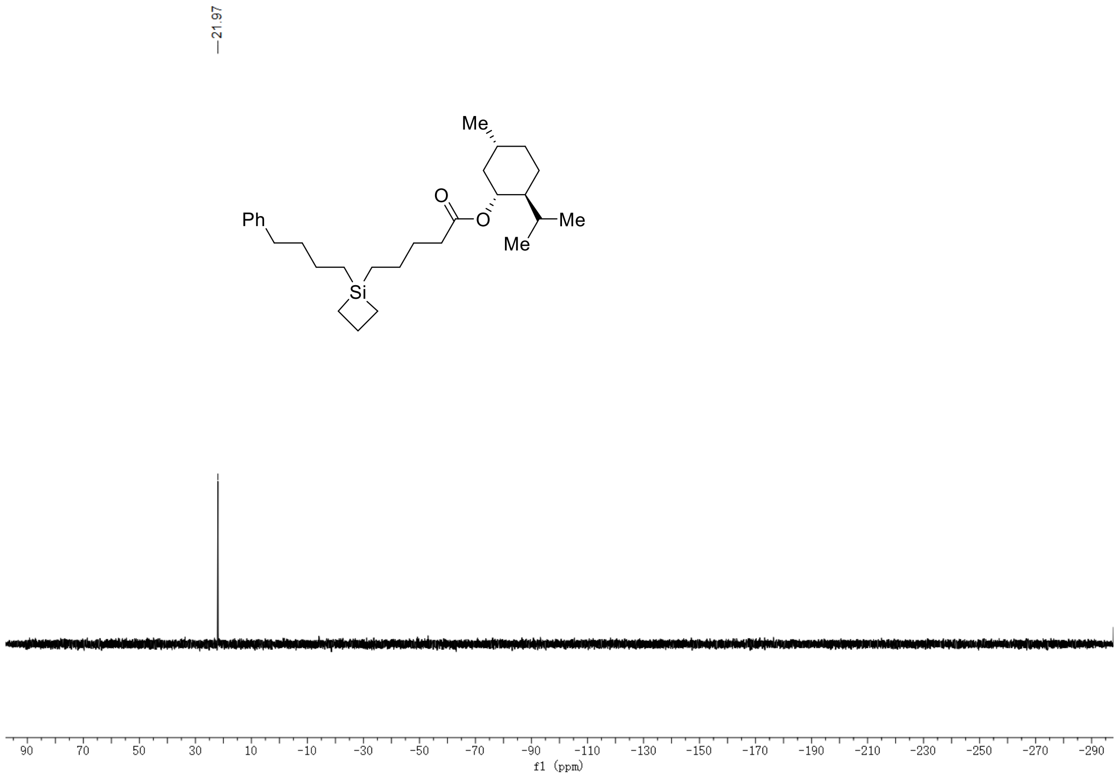

^{1}H NMR of **4ai** (600 MHz, $CDCl_3$, 25 ºC)

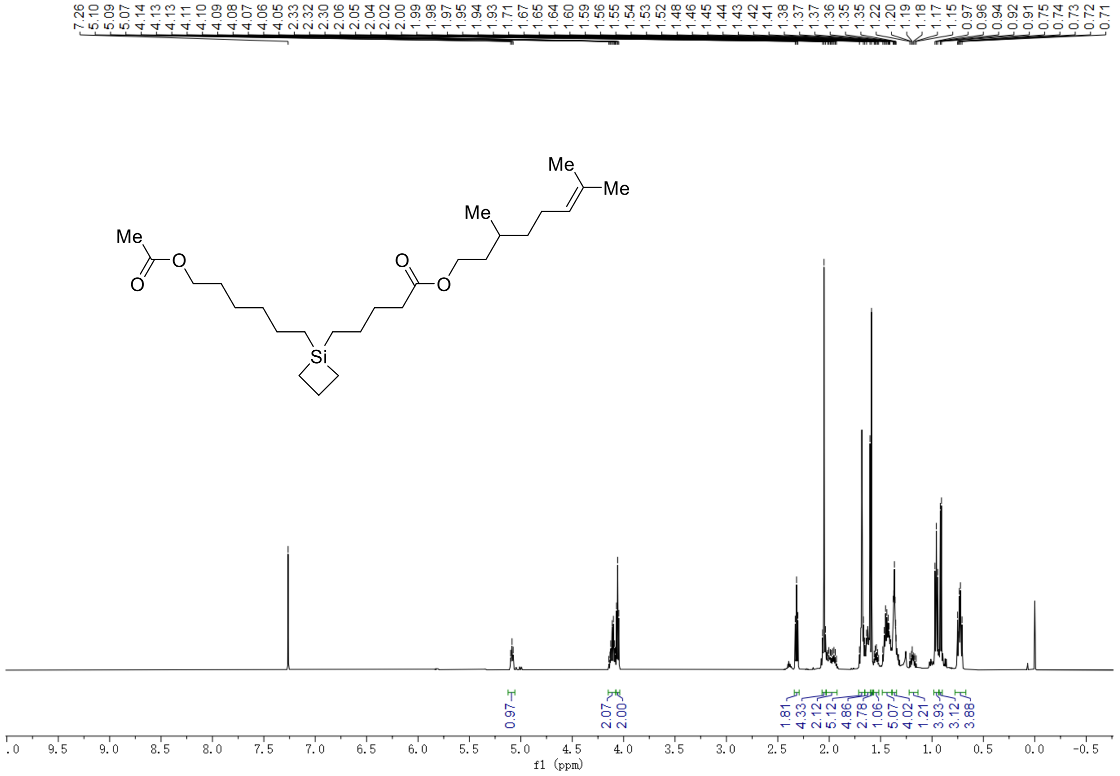

^{13}C NMR of **4ai** (151 MHz, $CDCl_3$, 25 ºC)

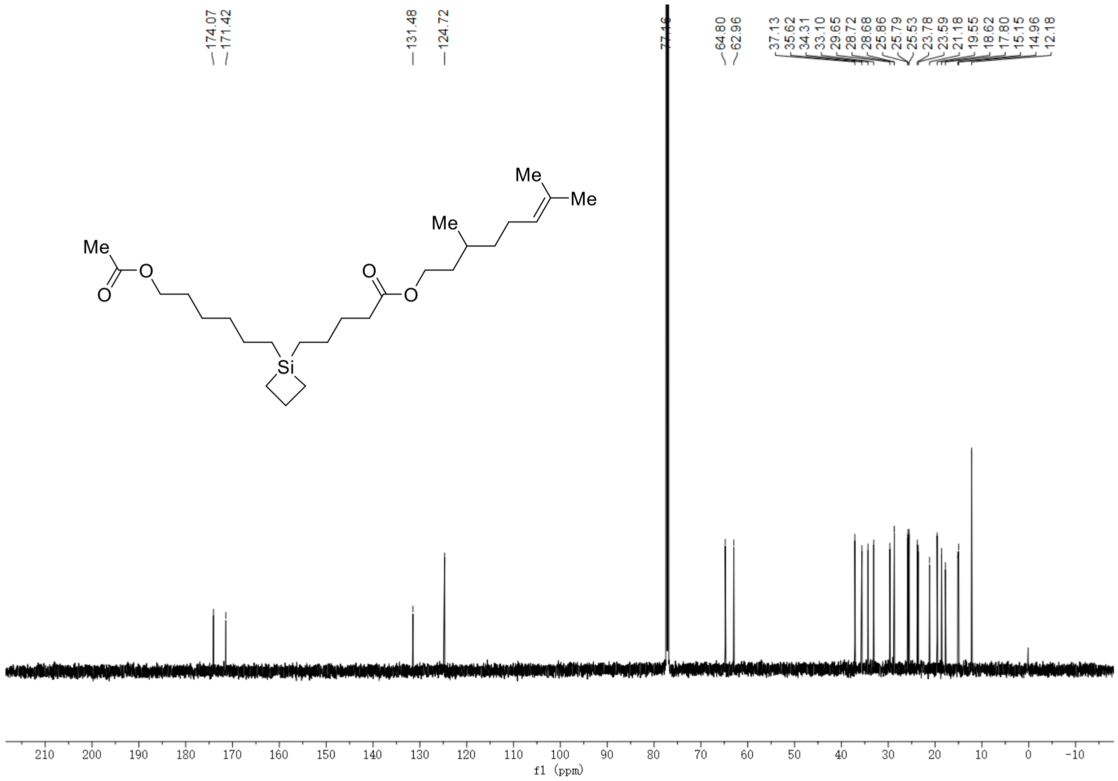

^{29}Si NMR of **4ai** (119 MHz, $CDCl_3$, 25 ºC)

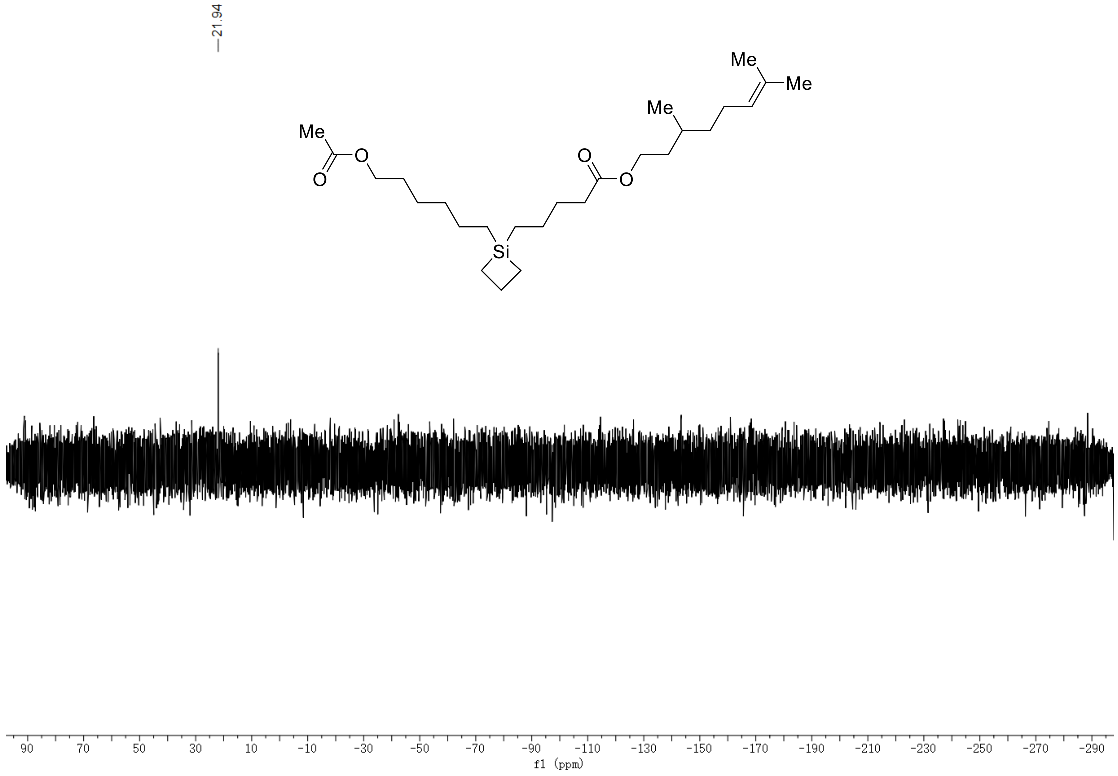

^{1}H NMR of **4aj** (600 MHz, $CDCl_3$, 25 ºC)

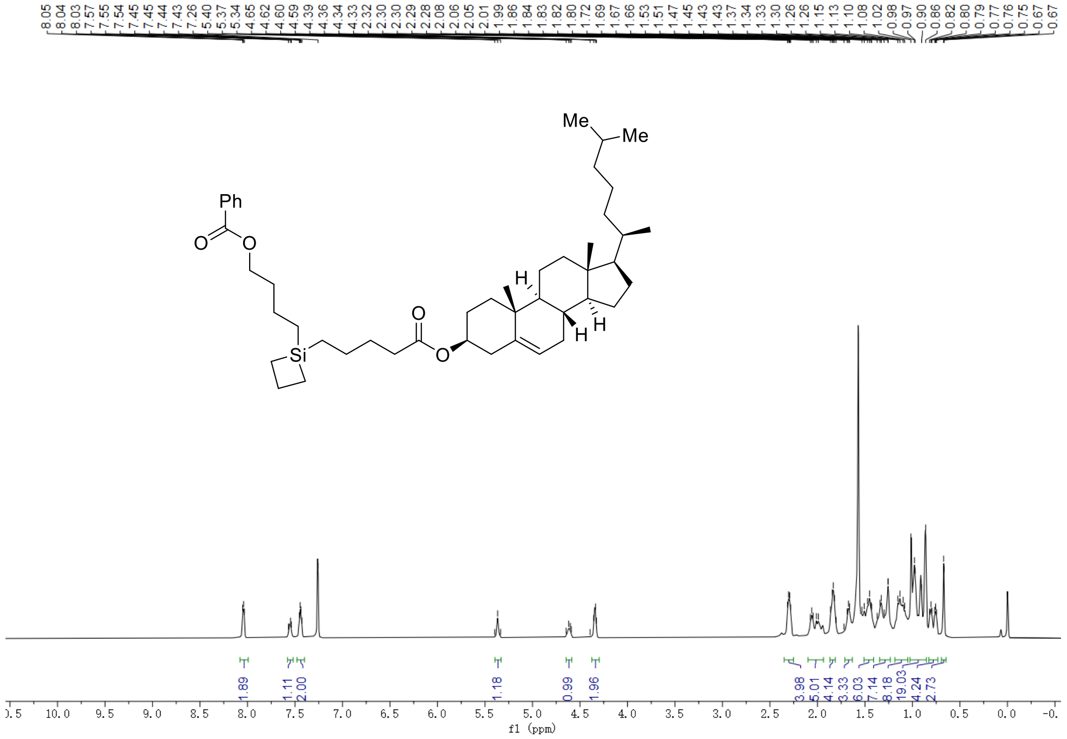

^{13}C NMR of **4aj** (151 MHz, $CDCl_3$, 25 ºC)

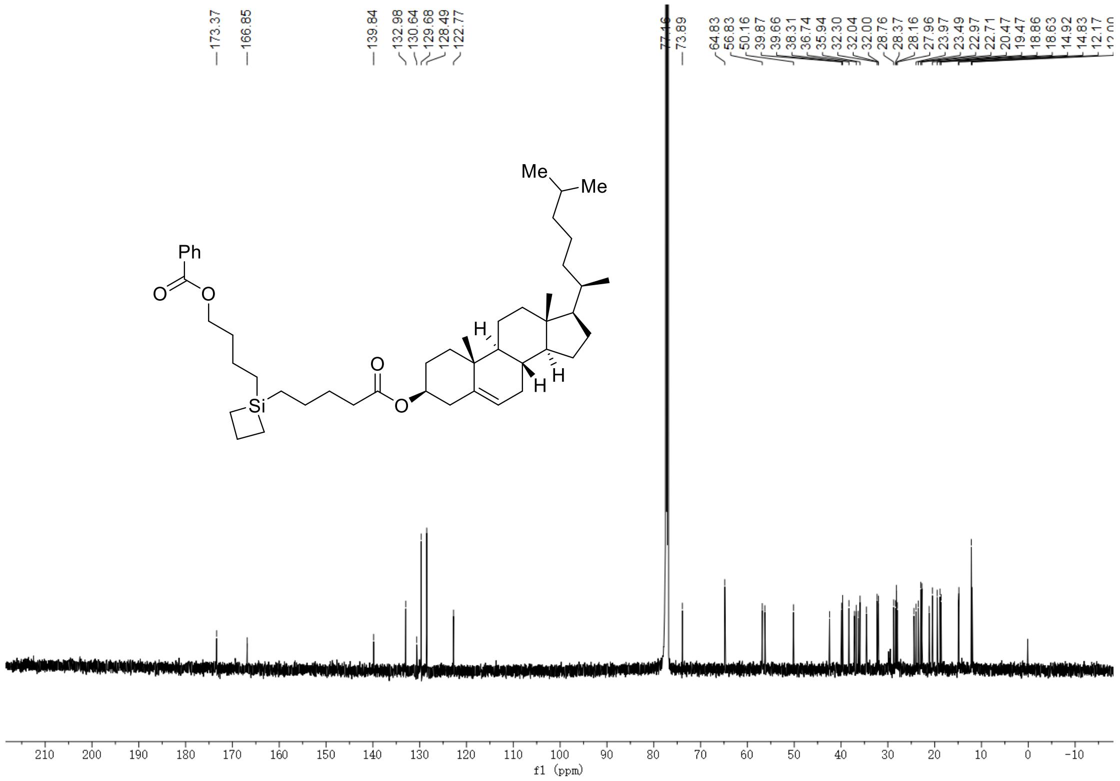

^{29}Si NMR of **4aj** (119 MHz, $CDCl_3$, 25 ºC)

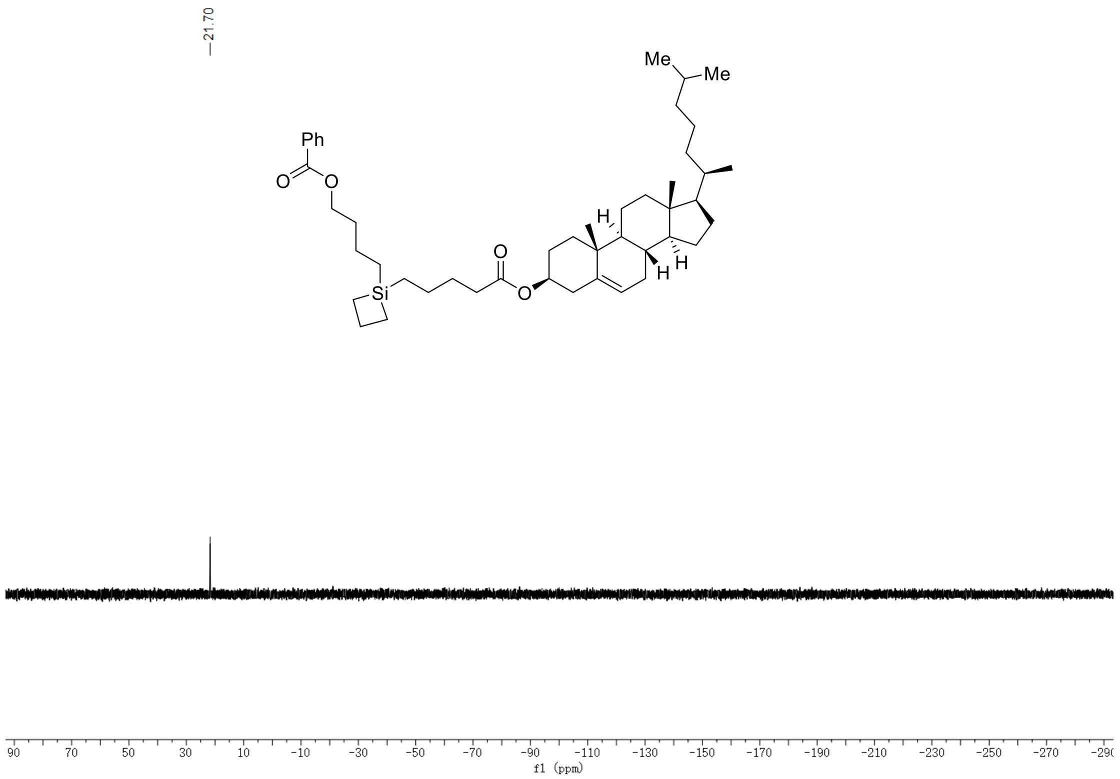

^{1}H NMR of **4ak** (600 MHz, $CDCl_3$, 25 ºC)

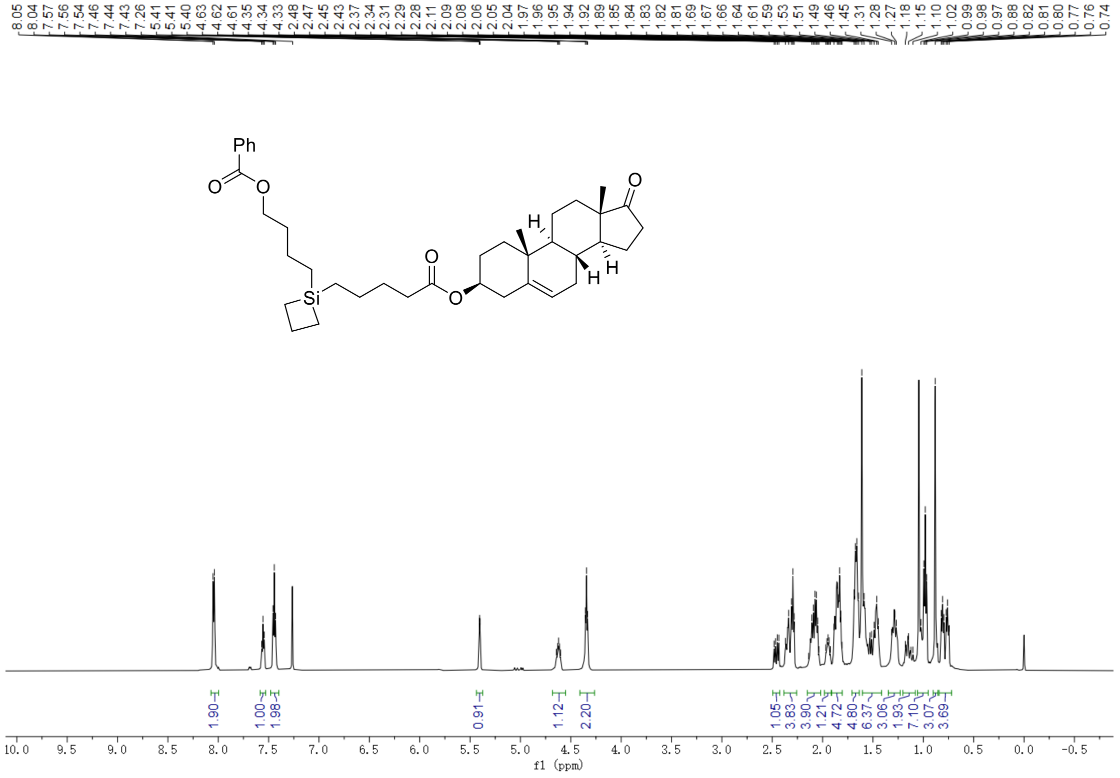

^{13}C NMR of **4ak** (151 MHz, $CDCl_3$, 25 ºC)

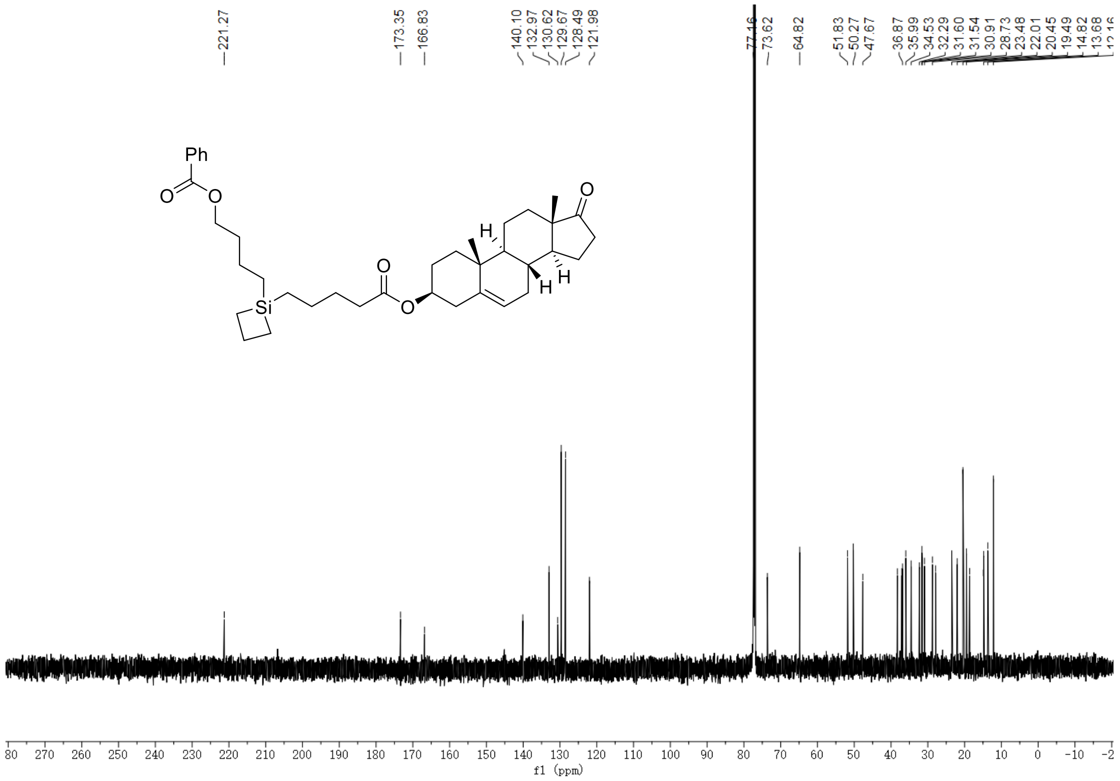

^{29}Si NMR of **4ak** (119 MHz, $CDCl_3$, 25 ºC)

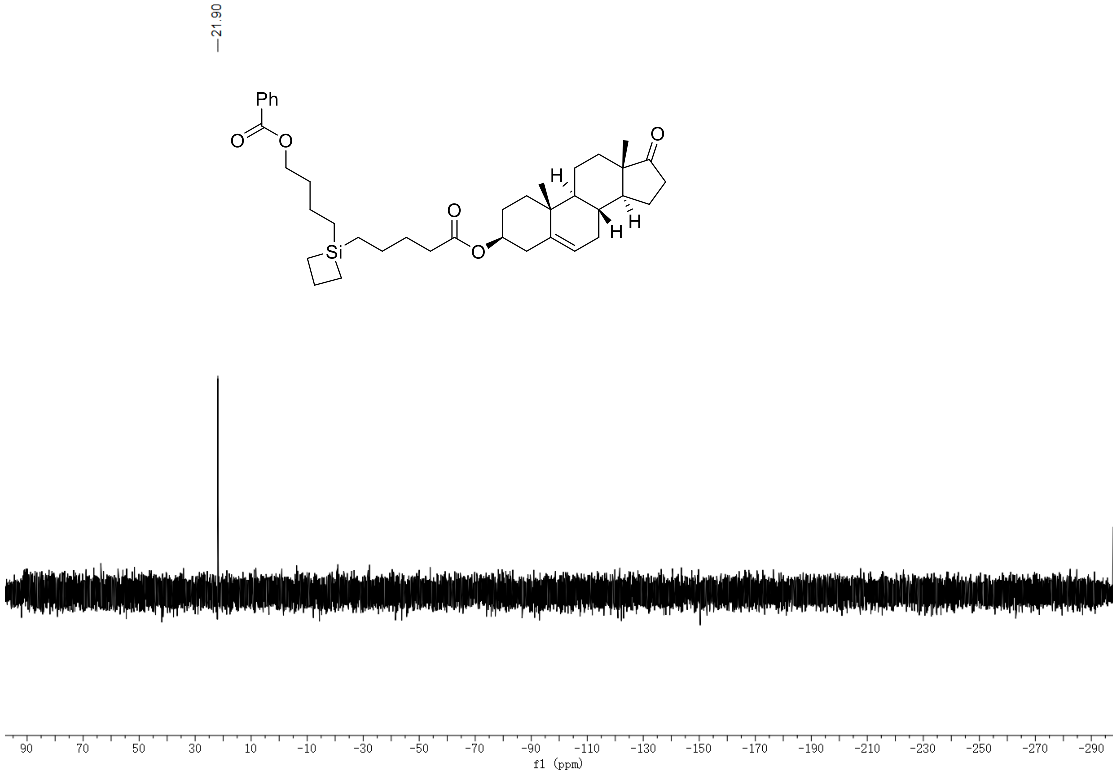

^{1}H NMR of **5** (400 MHz, $CDCl_3$, 25 ºC)

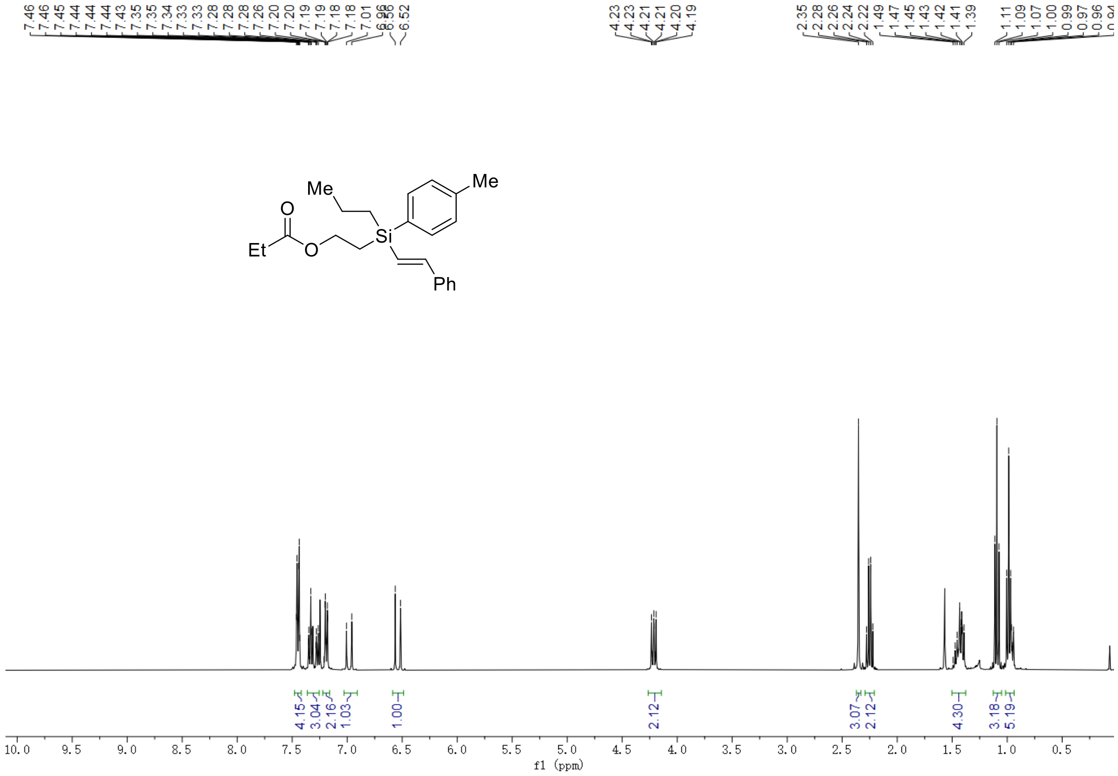

^{13}C NMR of **5** (151 MHz, $CDCl_3$, 25 ºC)

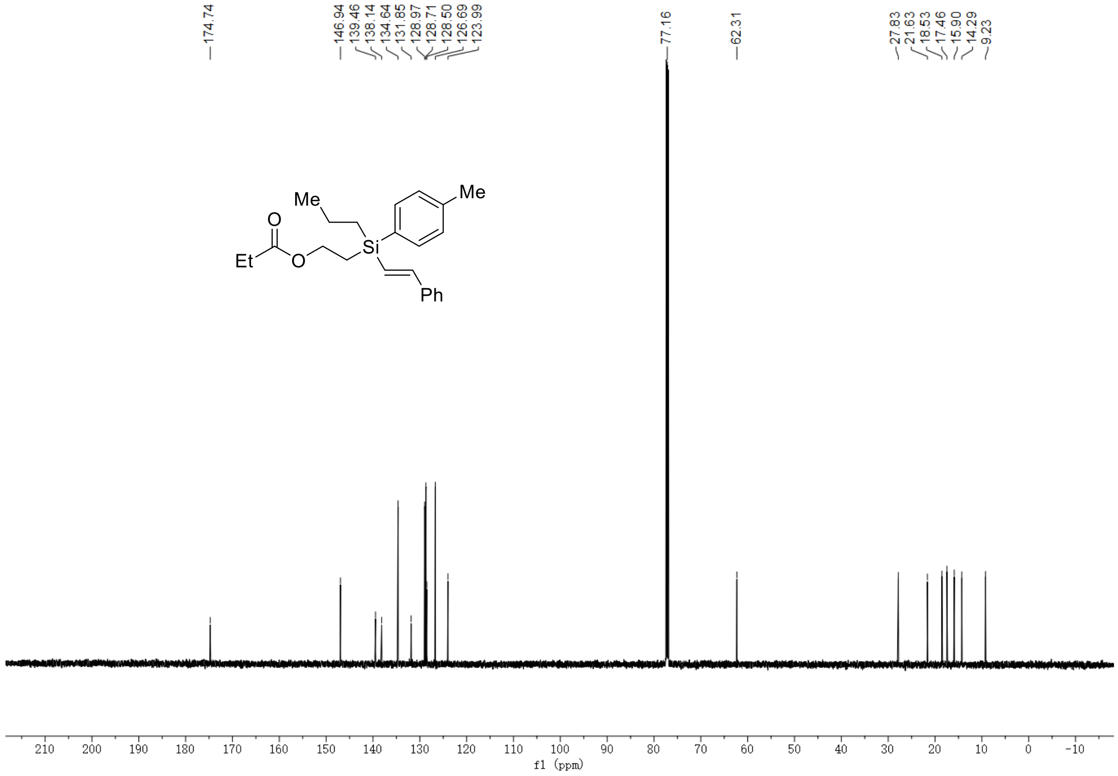

^{29}Si NMR of **5** (119 MHz, CDCl$_3$, 25 ºC)

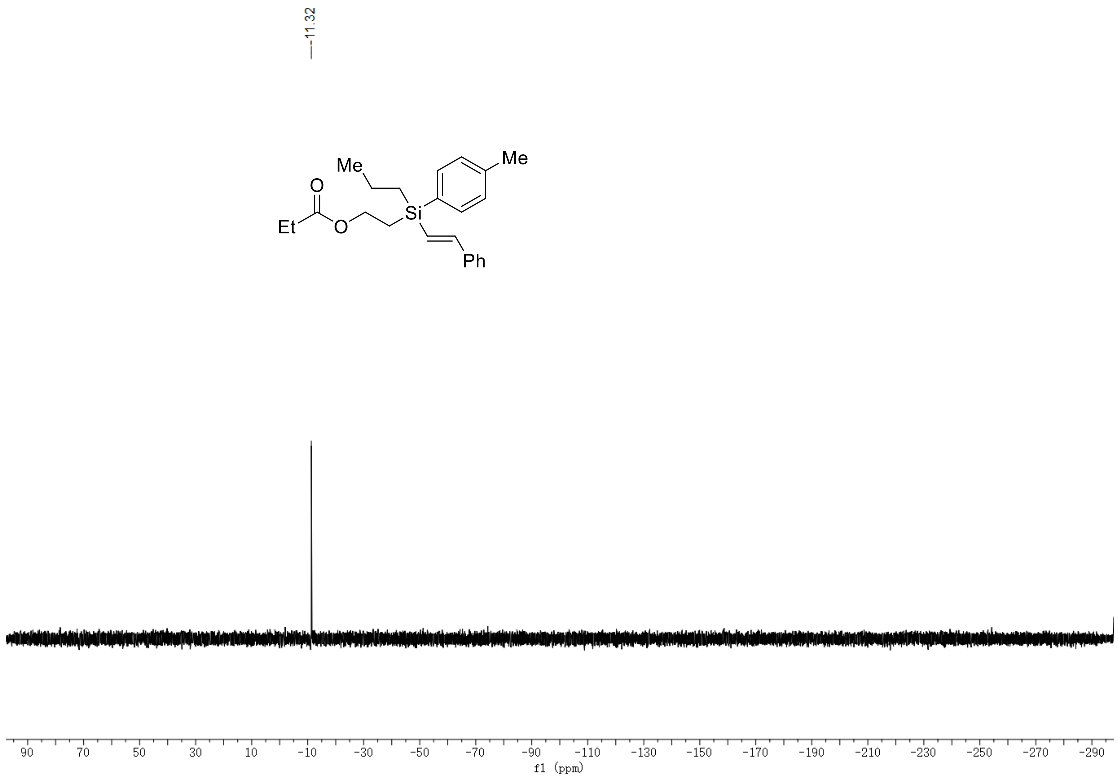

^{1}H NMR of **6** (400 MHz, CDCl$_3$, 25 ºC)

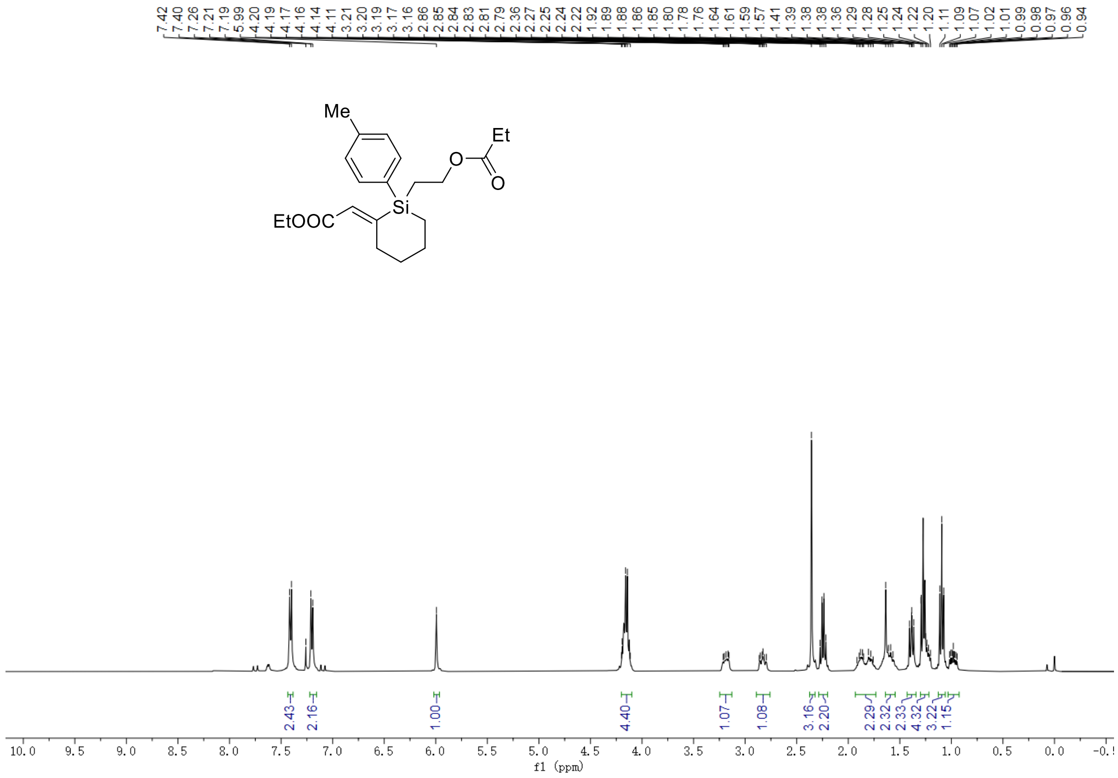

^{13}C NMR of **6** (151 MHz, $CDCl_3$, 25 ºC)

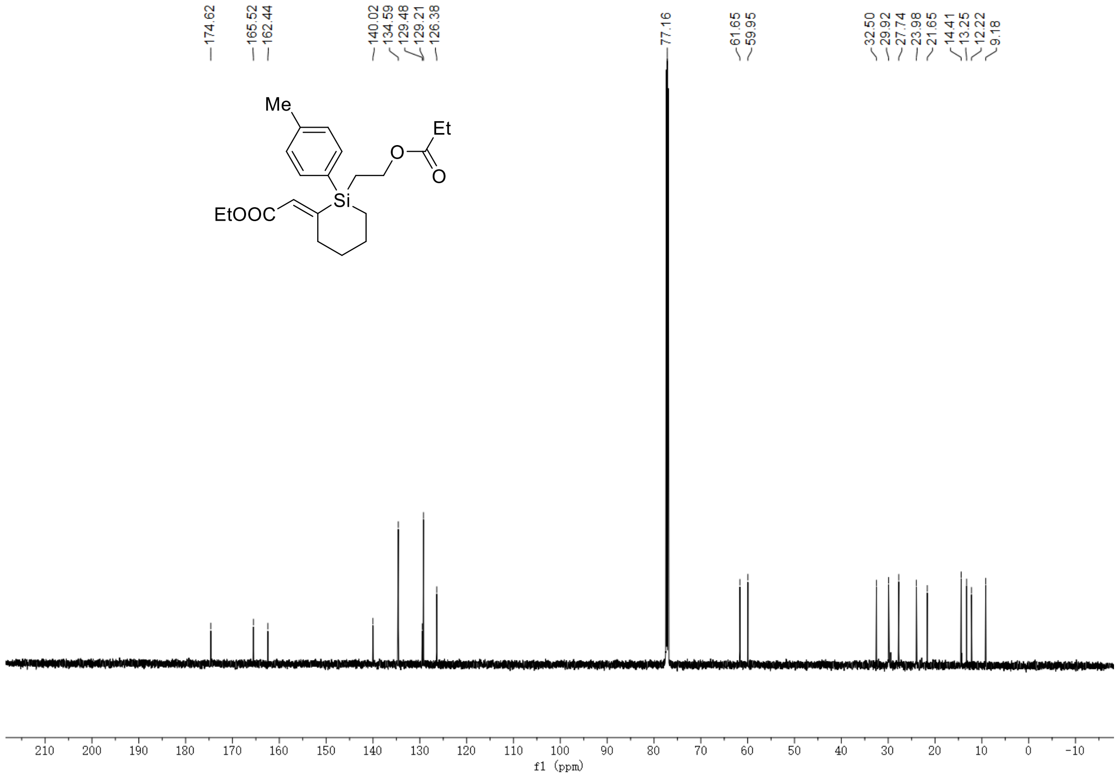

^{29}Si NMR of **6** (119 MHz, $CDCl_3$, 25 ºC)

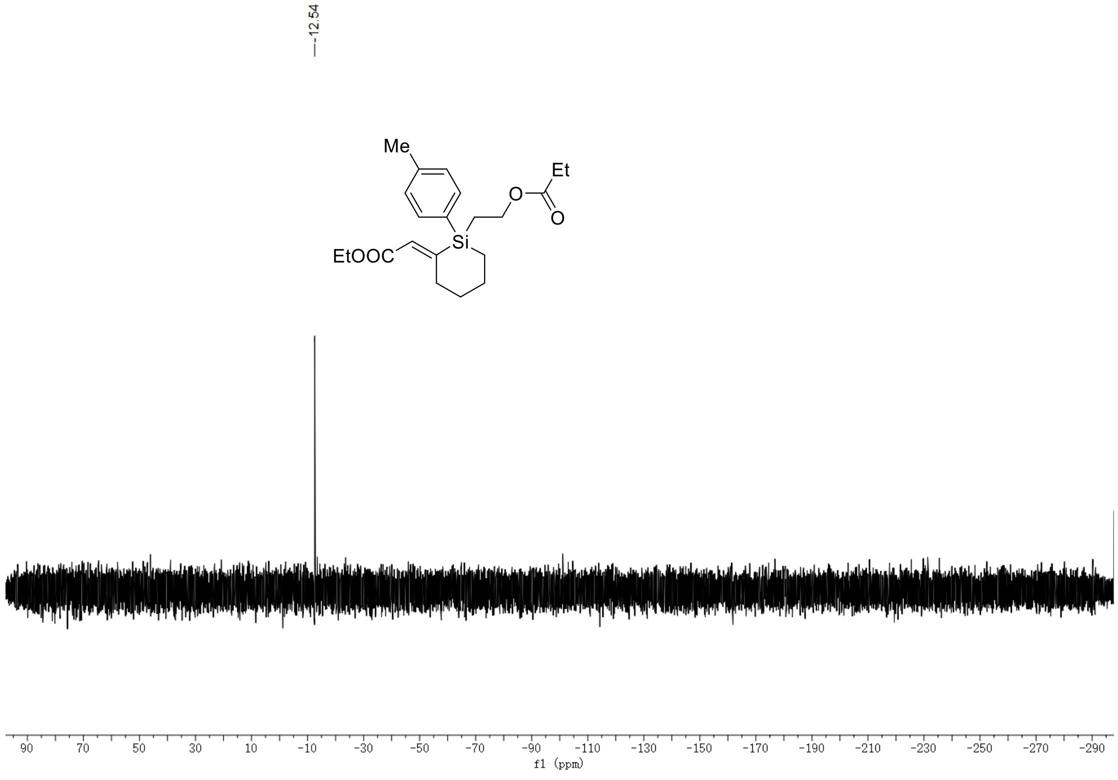

^{1}H NMR of **7** (400 MHz, $CDCl_3$, 25 ºC)

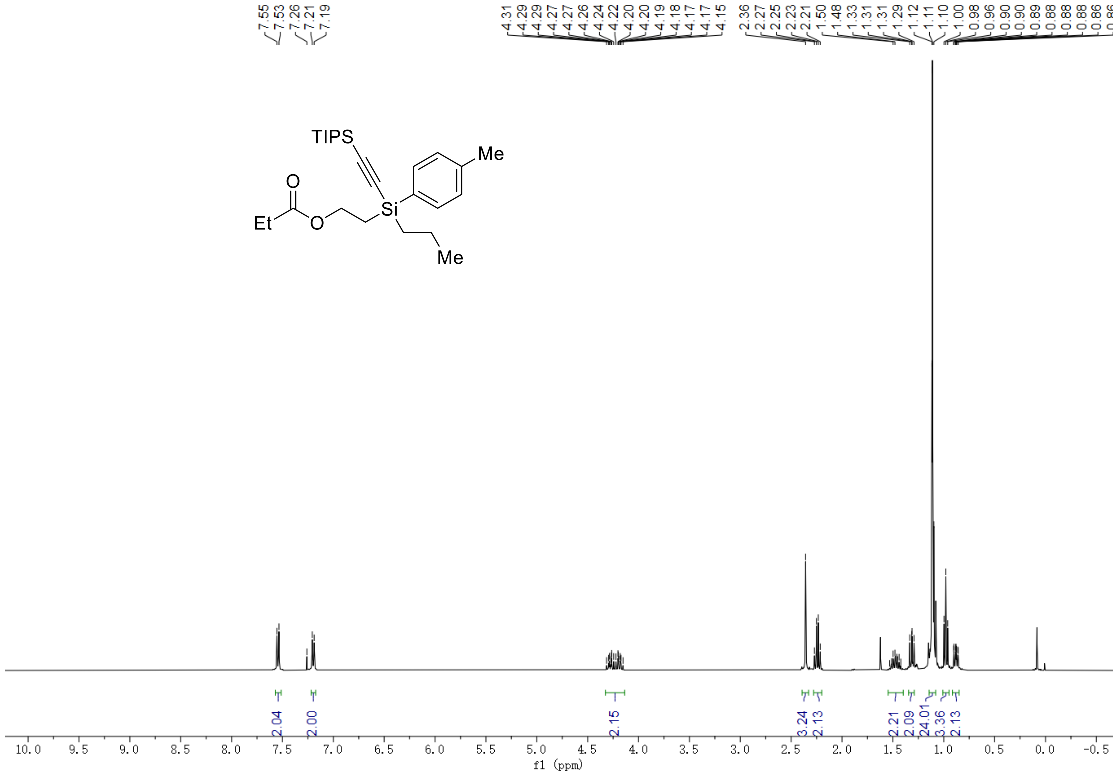

^{13}C NMR of **7** (151 MHz, $CDCl_3$, 25 ºC)

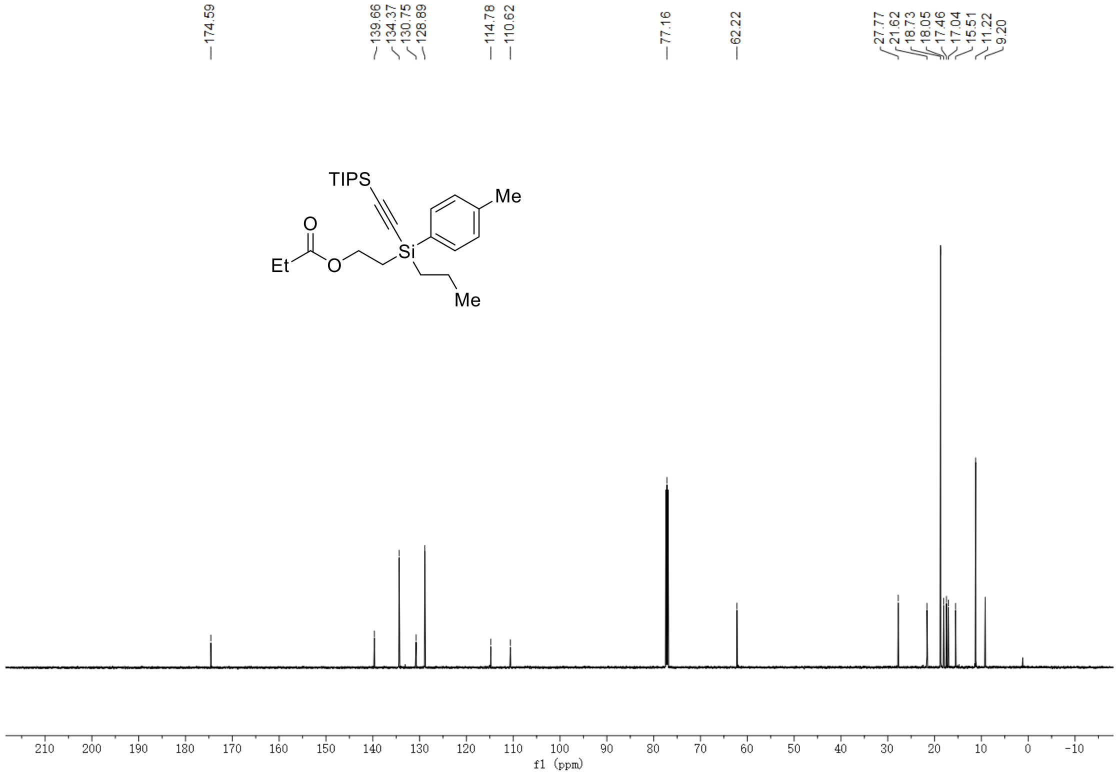

^{29}Si NMR of **7** (119 MHz, $CDCl_3$, 25 ºC)

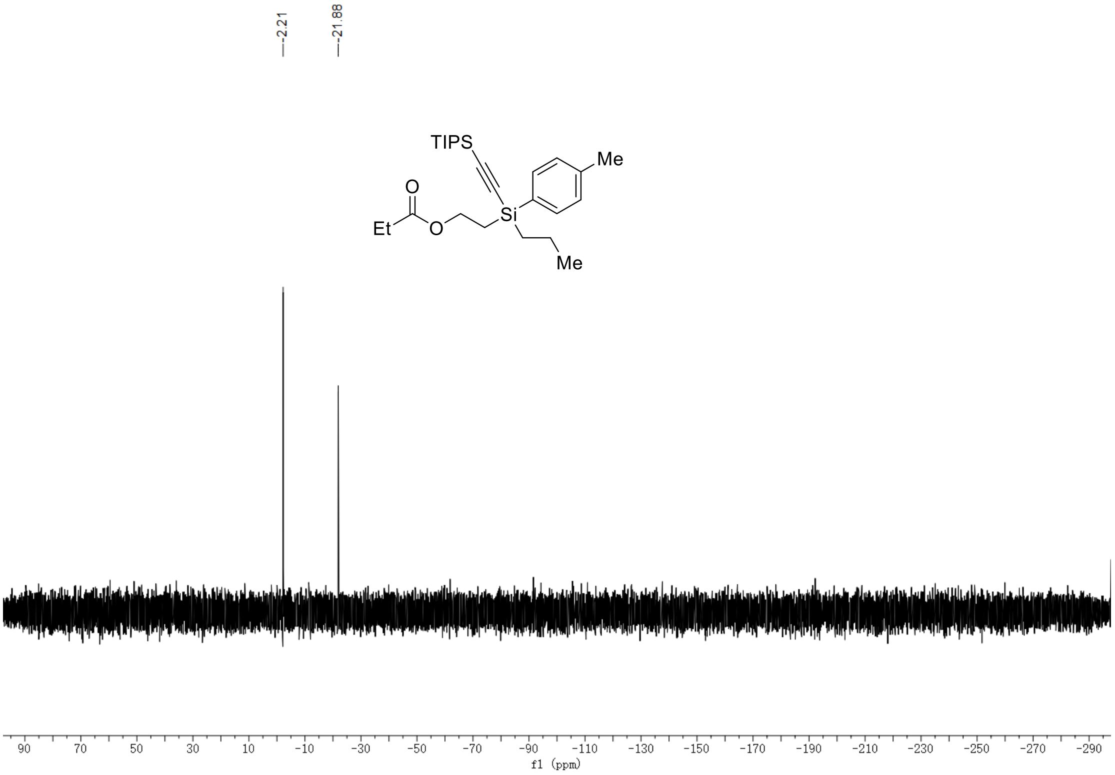

^{1}H NMR of **8** (600 MHz, $CDCl_3$, 25 ºC)

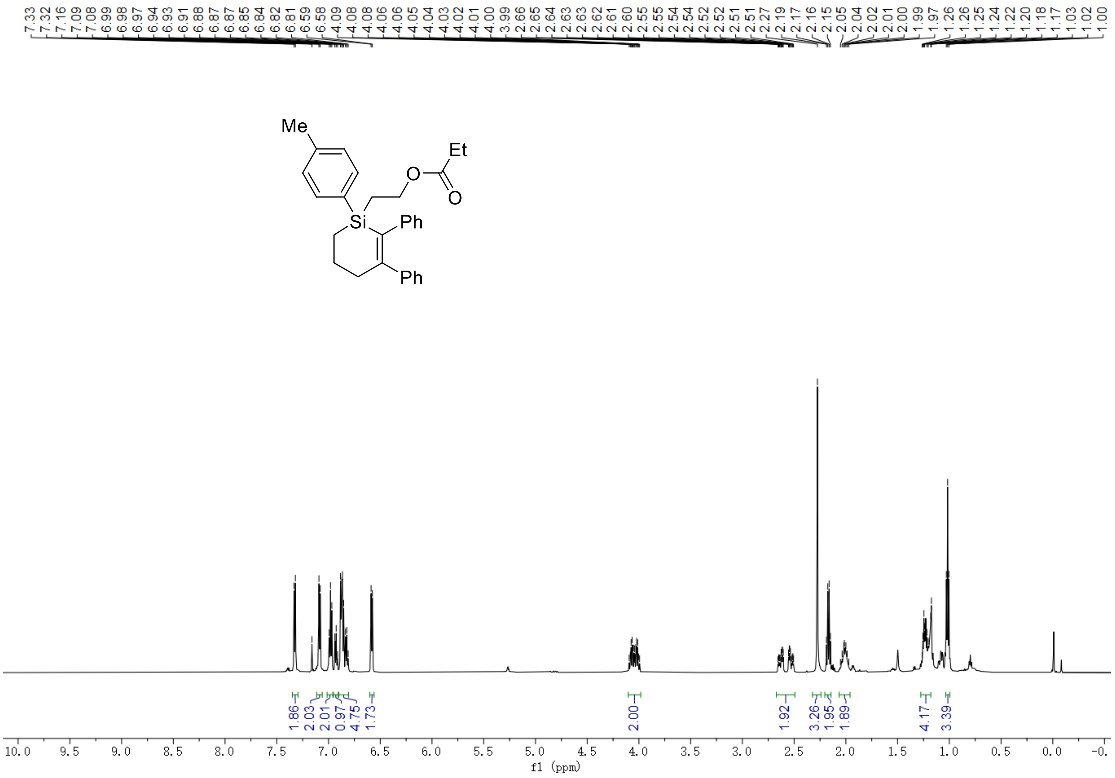

^{13}C NMR of **8** (151 MHz, $CDCl_3$, 25 ºC)

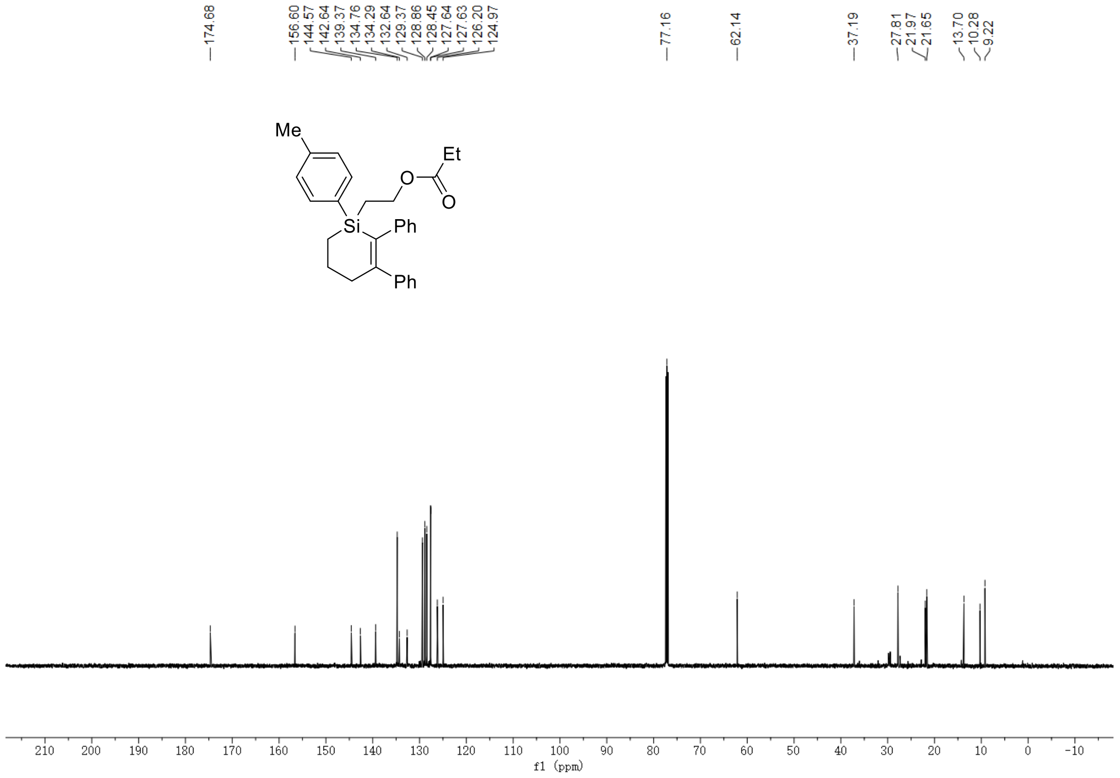

^{29}Si NMR of **8** (119 MHz, $CDCl_3$, 25 ºC)

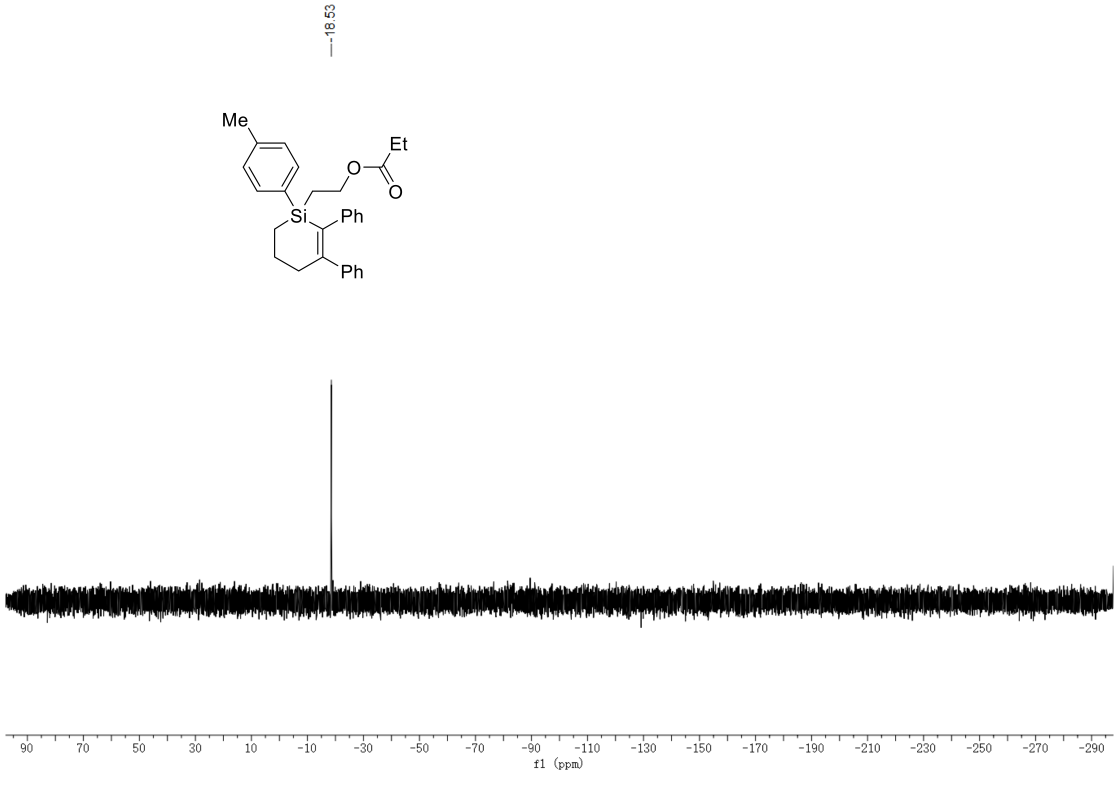

^{1}H NMR of **9** (400 MHz, $CDCl_3$, 25 ºC)

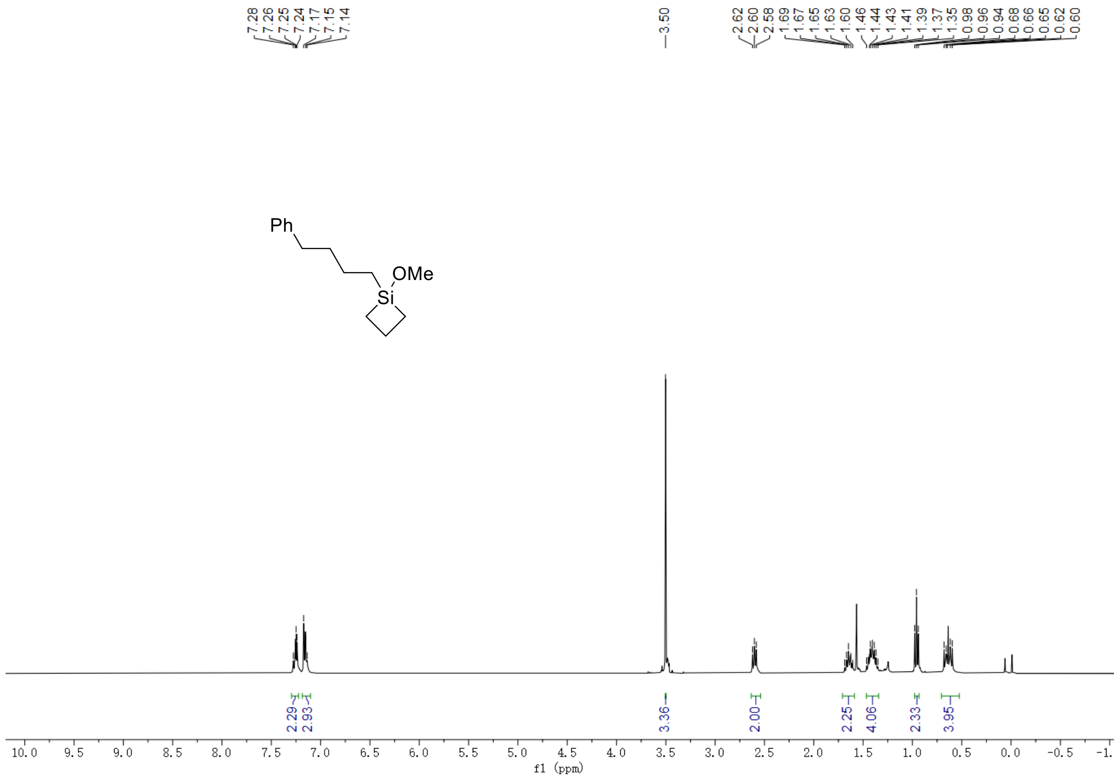

^{13}C NMR of **9** (151 MHz, $CDCl_3$, 25 ºC)

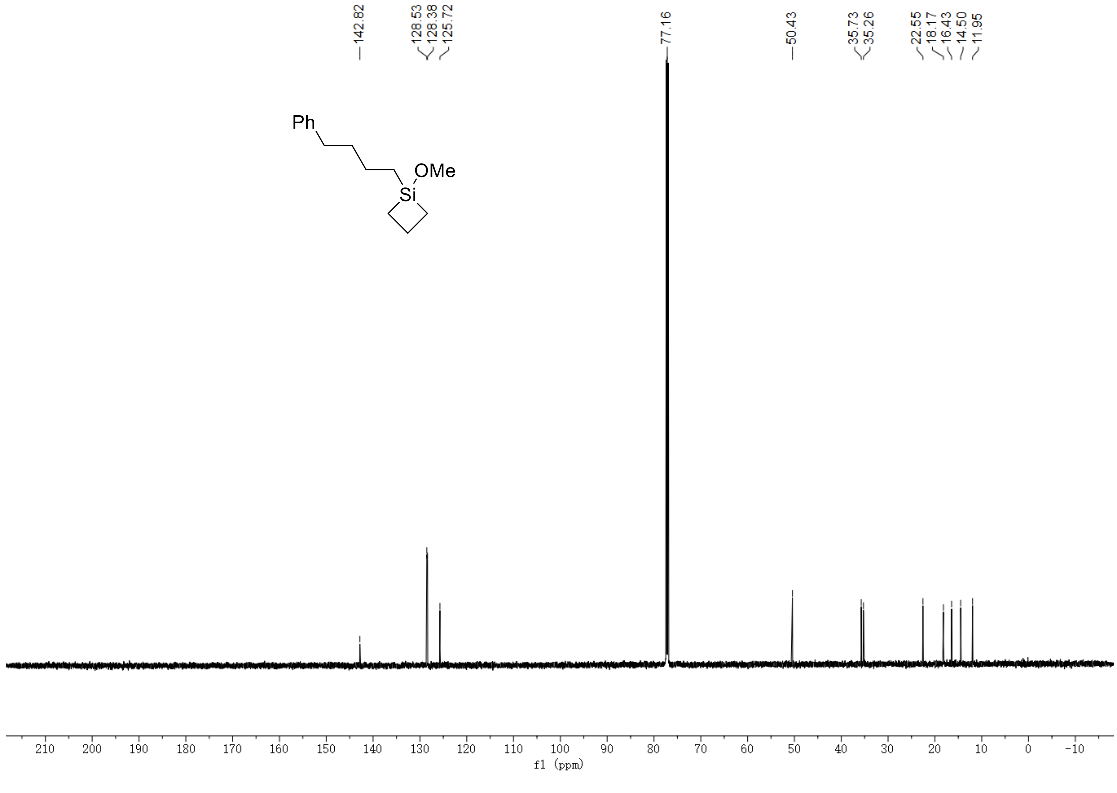

^{29}Si NMR of **9** (119 MHz, $CDCl_3$, 25 ºC)

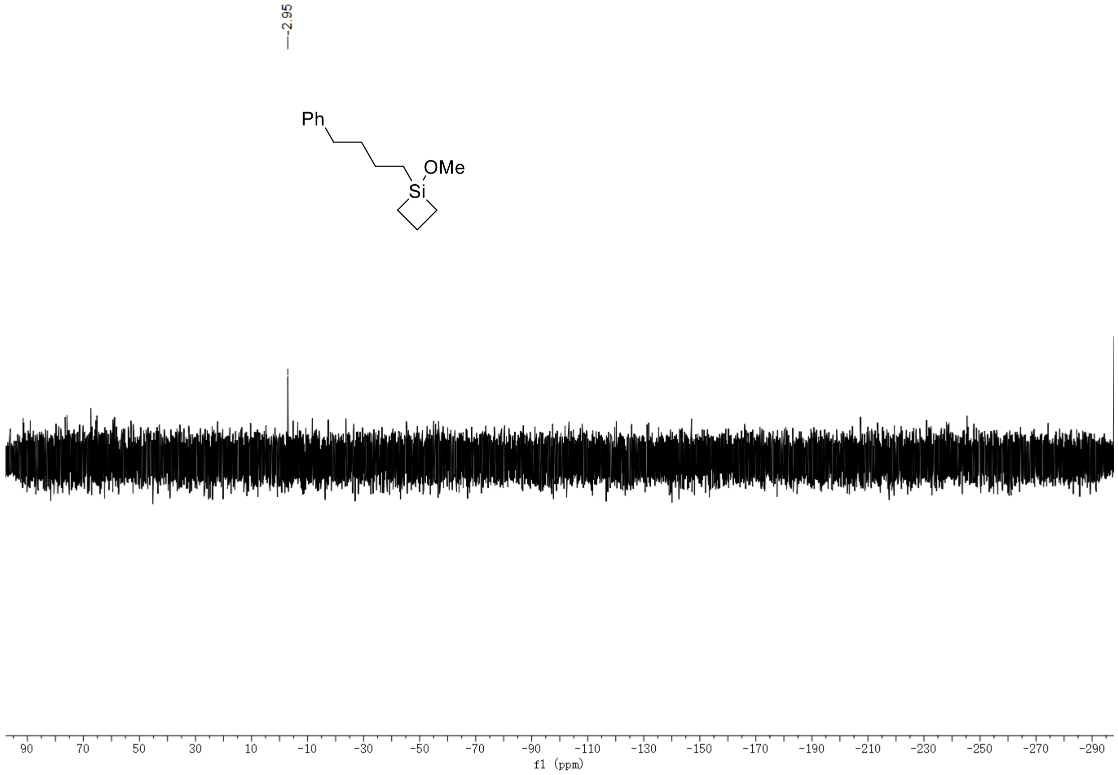

^{1}H NMR of **10a** (400 MHz, $CDCl_3$, 25 ºC)

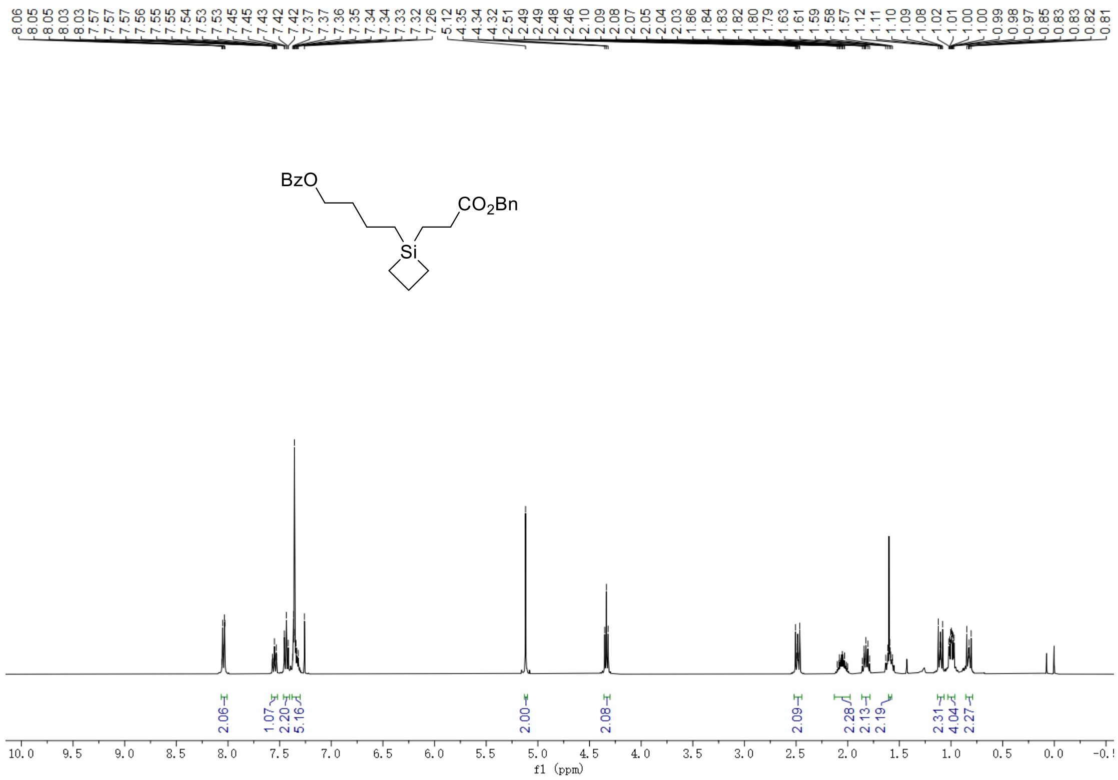

^{13}C NMR of **10a** (151 MHz, $CDCl_3$, 25 ºC)

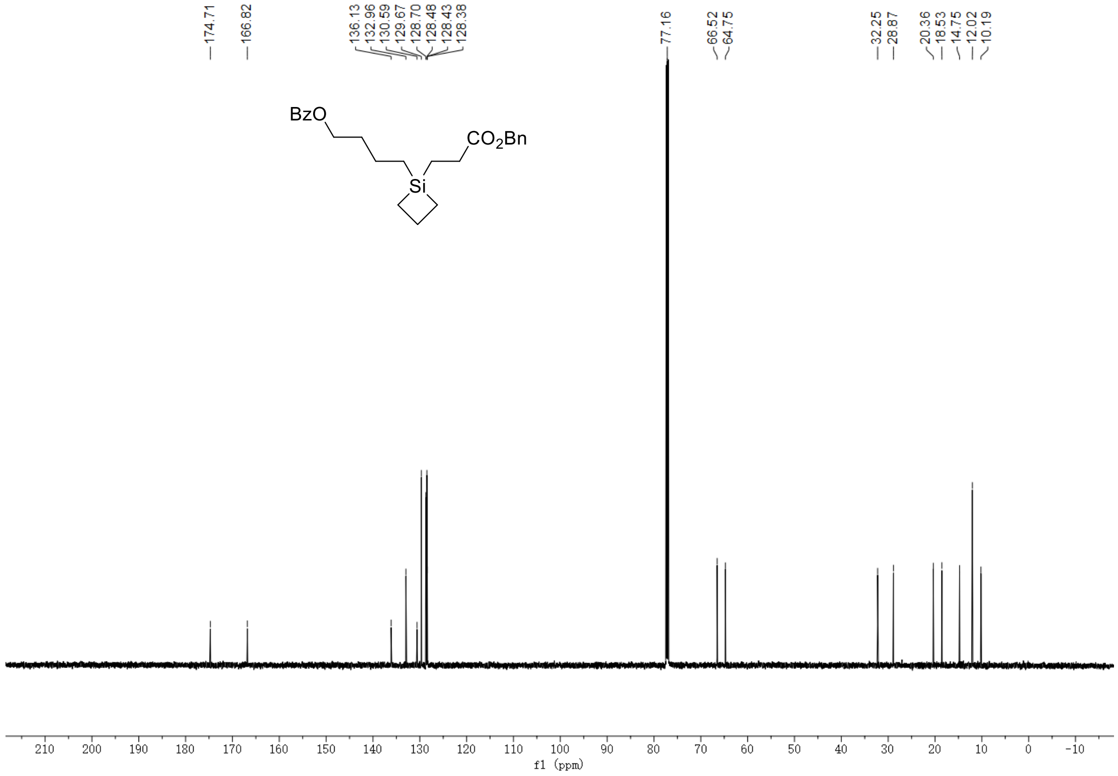

^{29}Si NMR of **10a** (119 MHz, $CDCl_3$, 25 ºC)

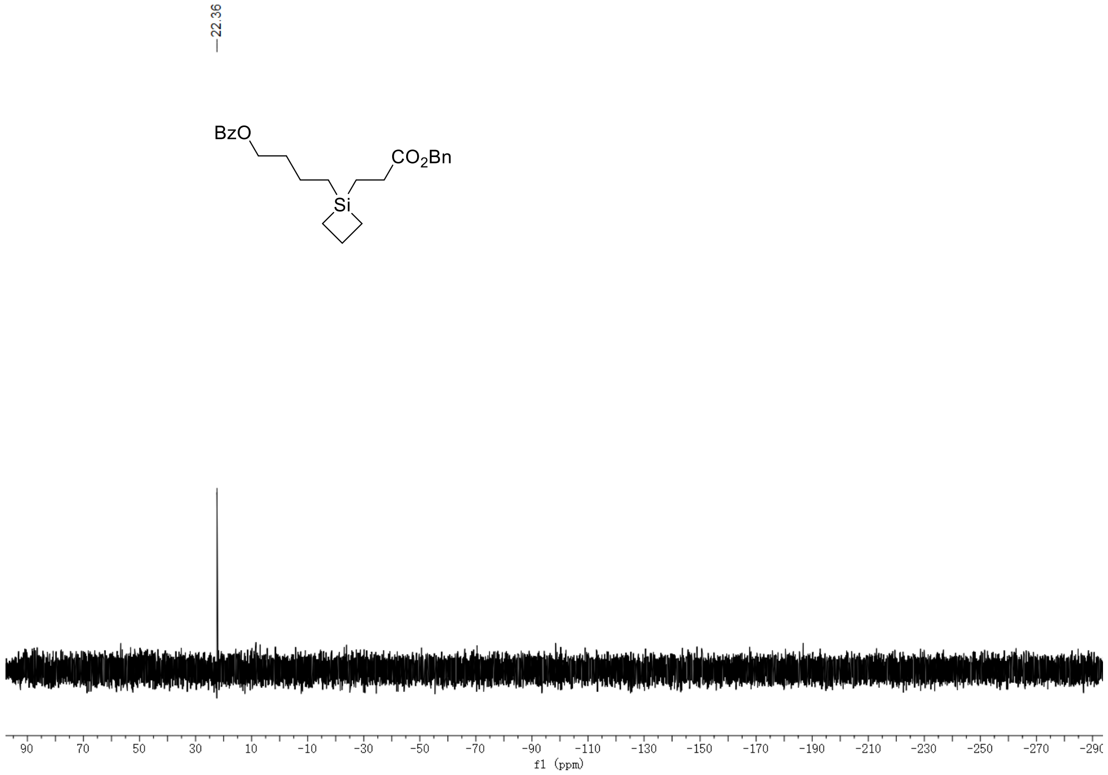

^{1}H NMR of **10b** (600 MHz, $CDCl_3$, 25 ºC)

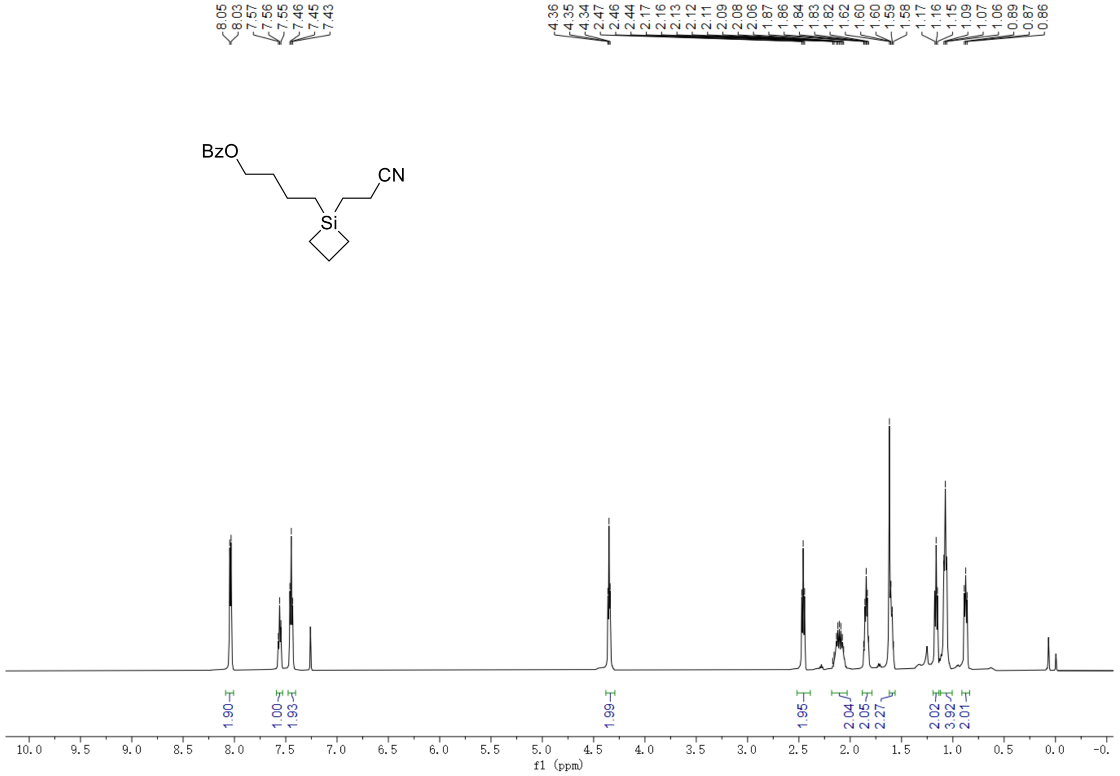

^{13}C NMR of **10b** (151 MHz, $CDCl_3$, 25 ºC)

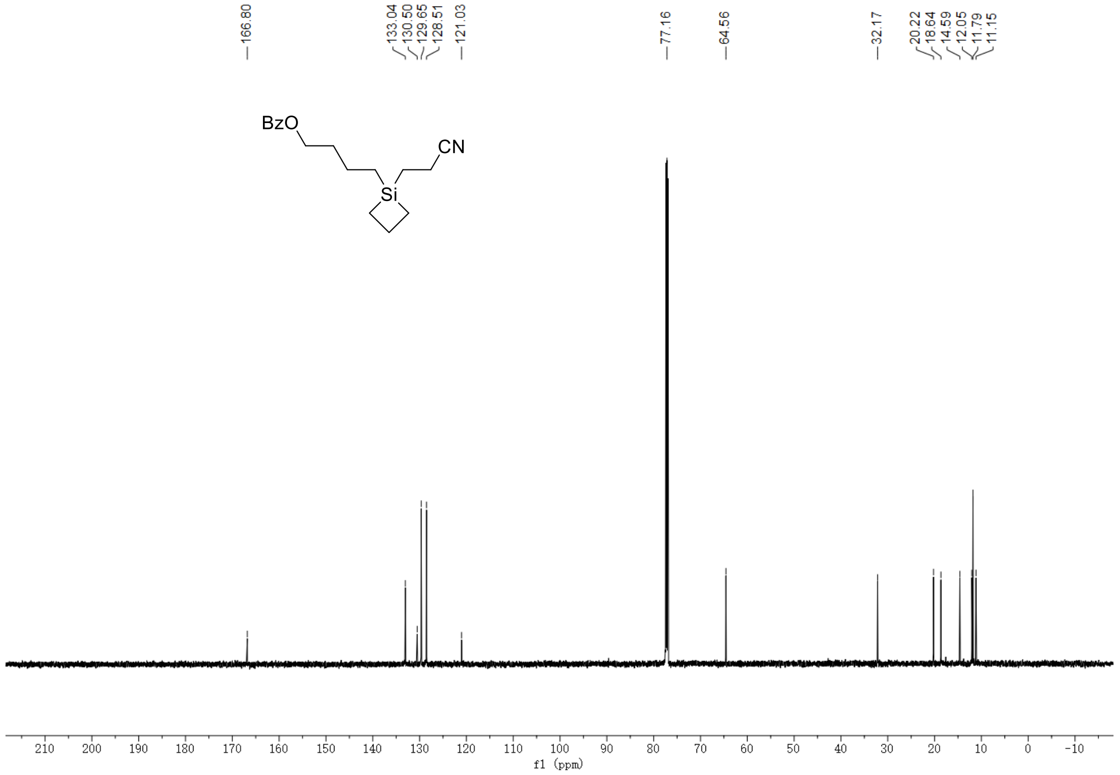

^{29}Si NMR of **10b** (119 MHz, $CDCl_3$, 25 ºC)

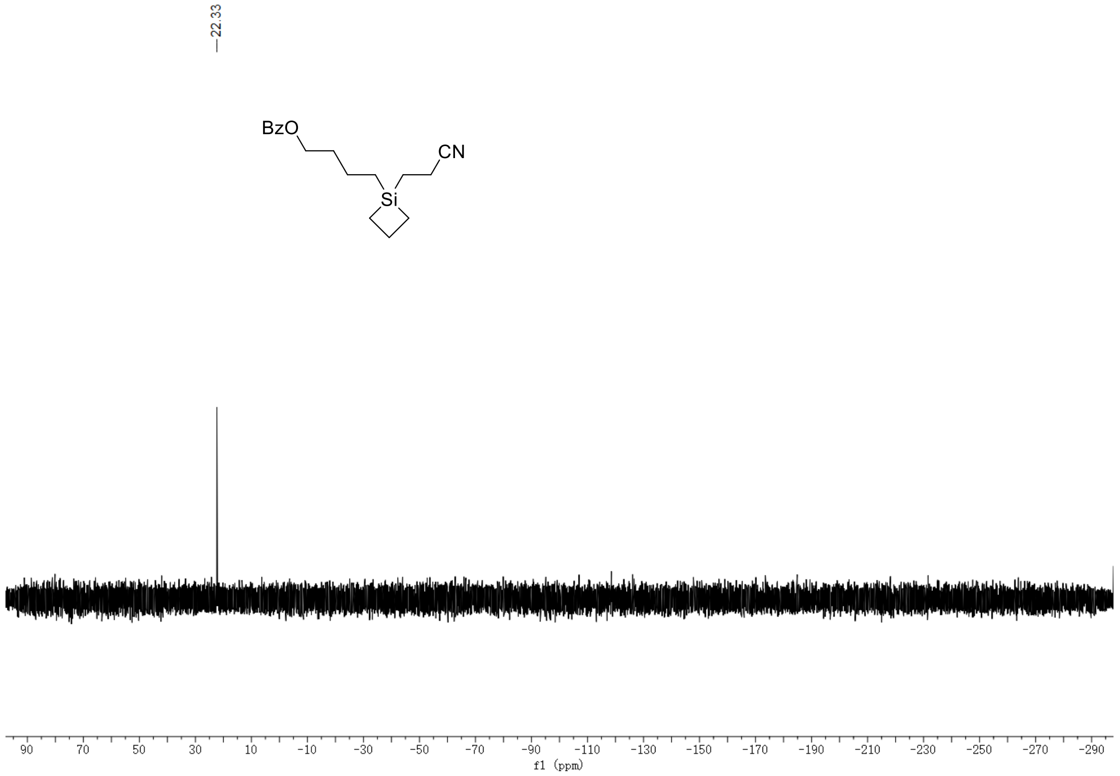

^{1}H NMR of **10c** (600 MHz, $CDCl_3$, 25 ºC)

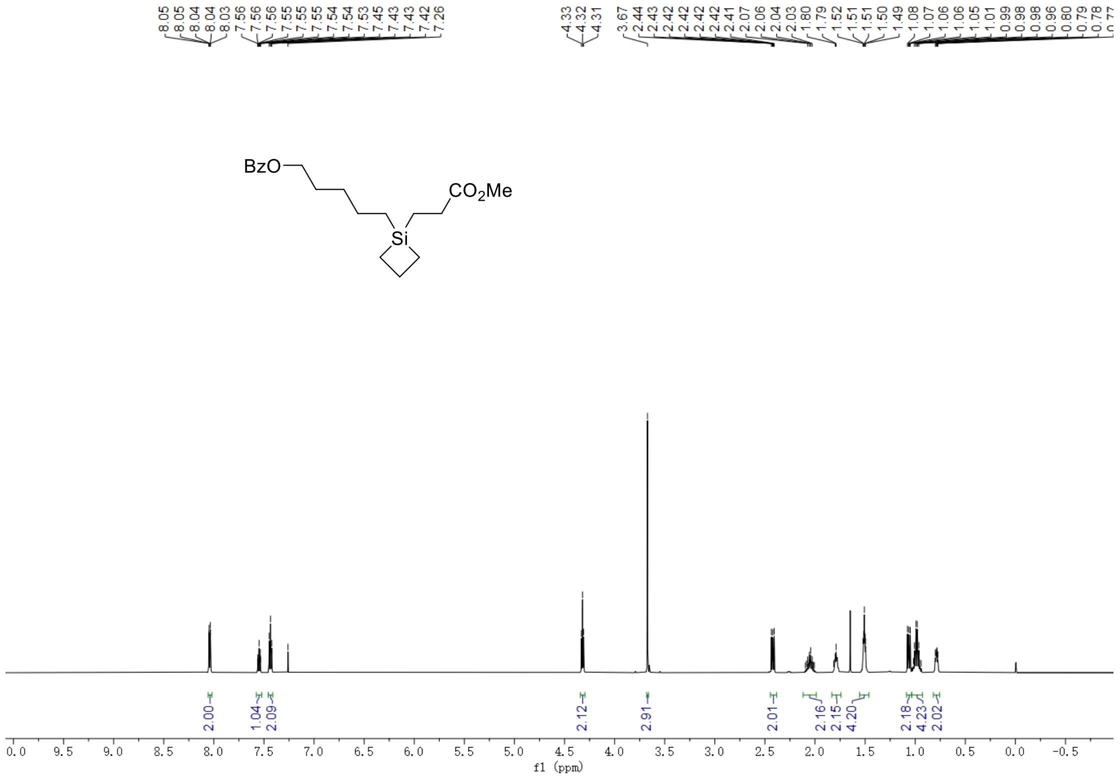

^{13}C NMR of **10c** (151 MHz, $CDCl_3$, 25 ºC)

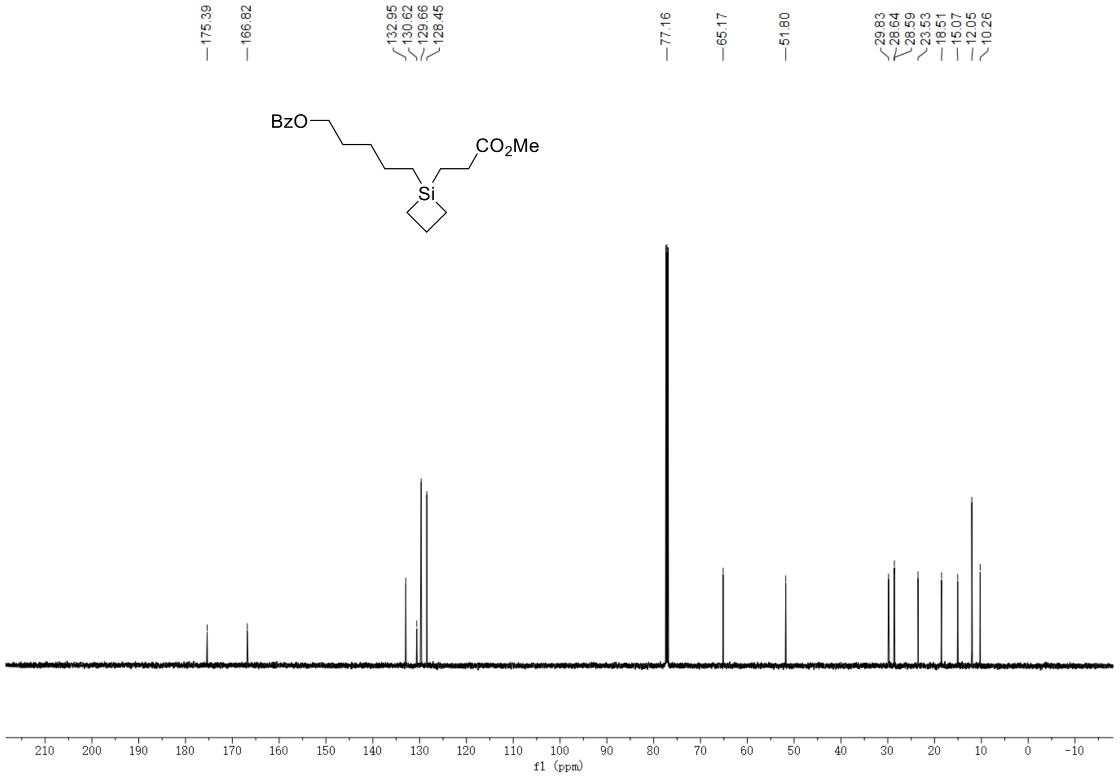

^{29}Si NMR of **10c** (119 MHz, $CDCl_3$, 25 ºC)

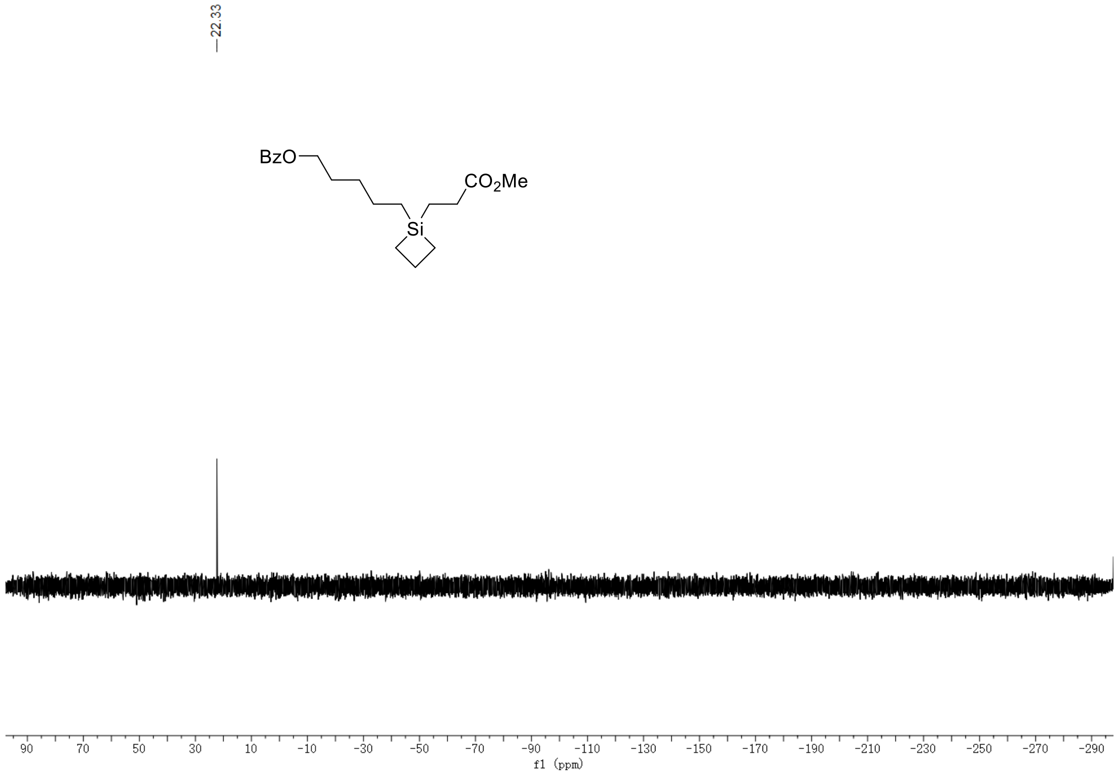

^{1}H NMR of **11** (600 MHz, $CDCl_3$, 25 ºC)

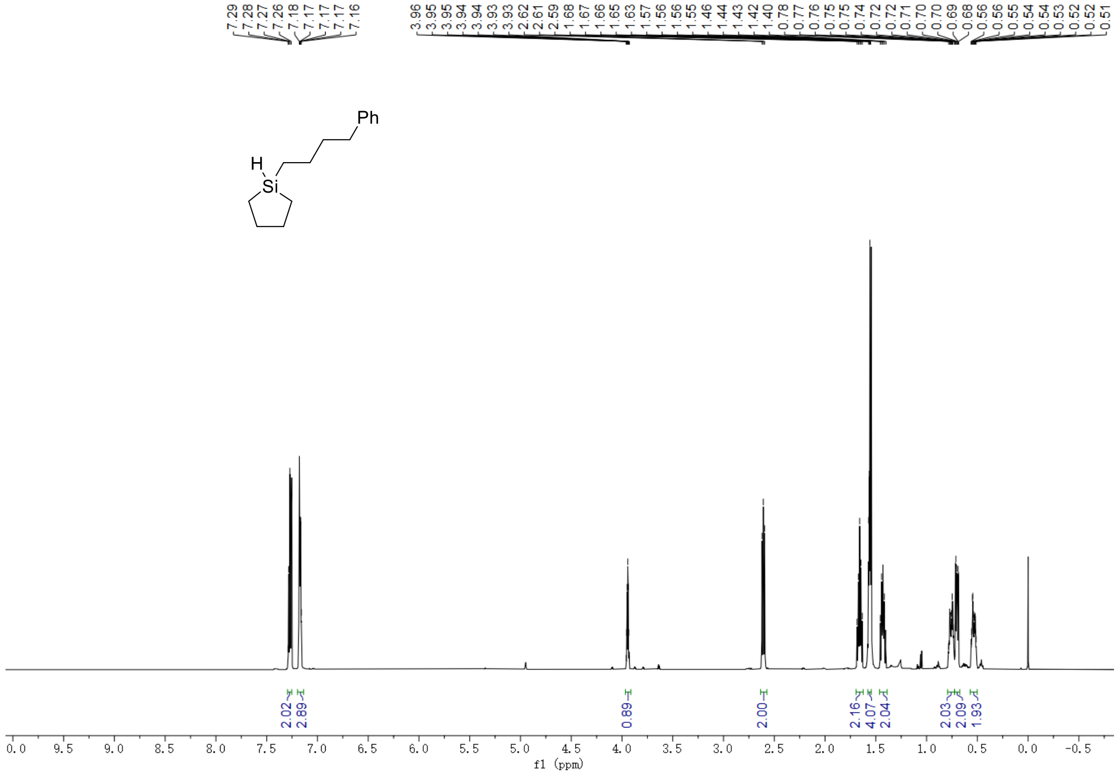

^{13}C NMR of **11** (151 MHz, $CDCl_3$, 25 ºC)

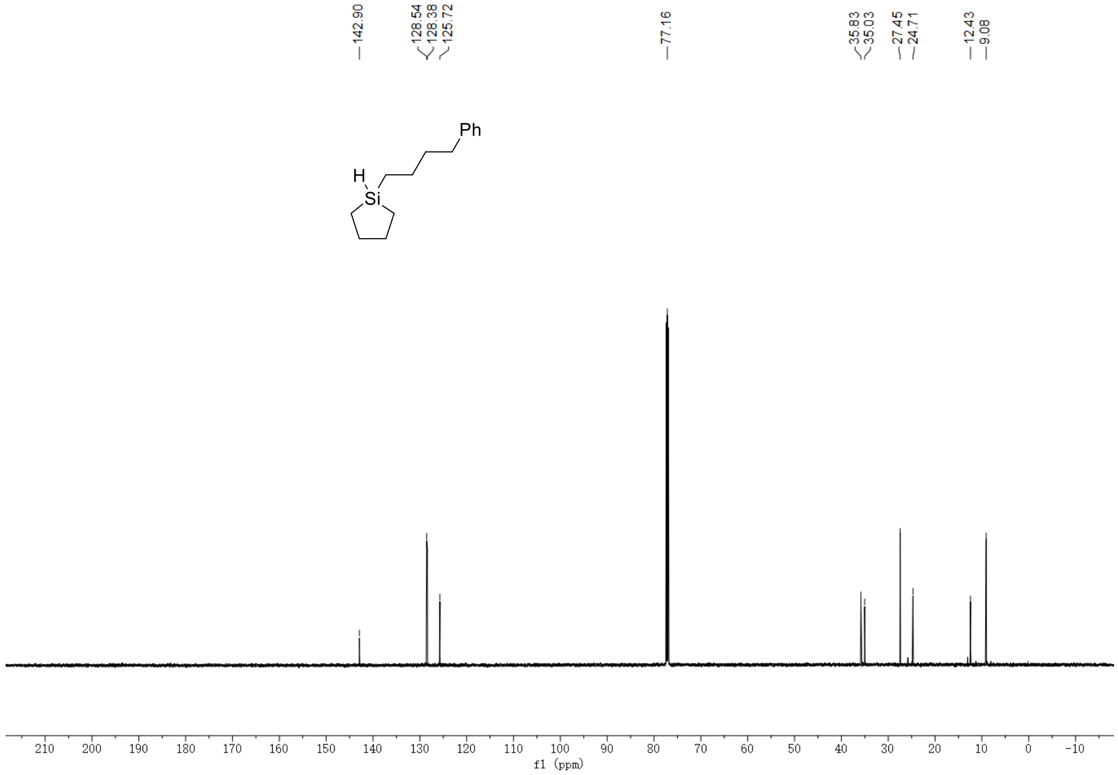

^{29}Si NMR of **11** (119 MHz, $CDCl_3$, 25 ºC)

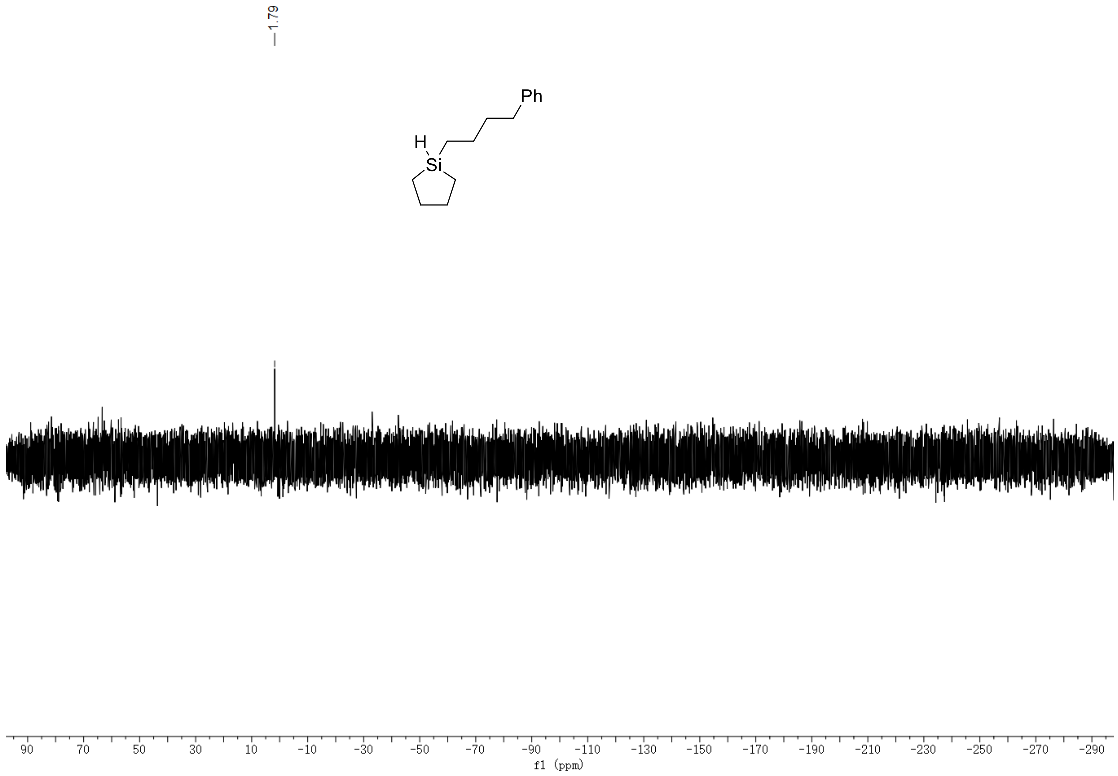

^{1}H NMR of **12** (400 MHz, $CDCl_3$, 25 ºC)

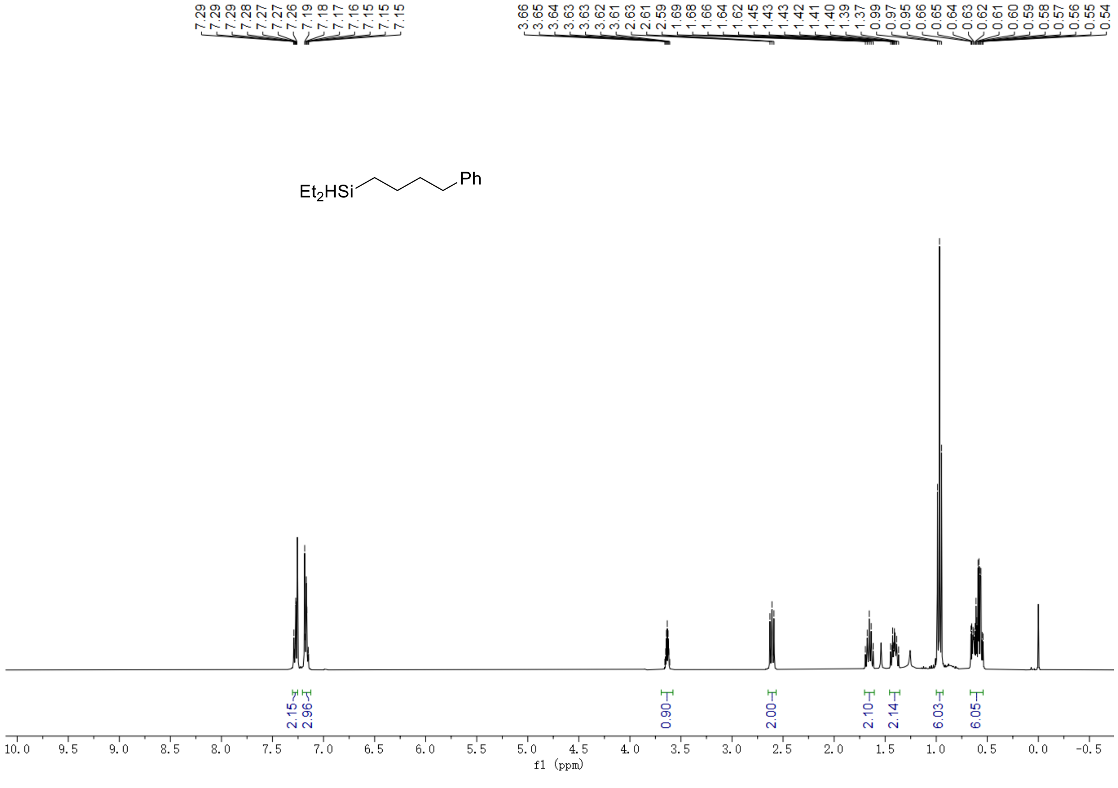

^{13}C NMR of **12** (151 MHz, $CDCl_3$, 25 ºC)

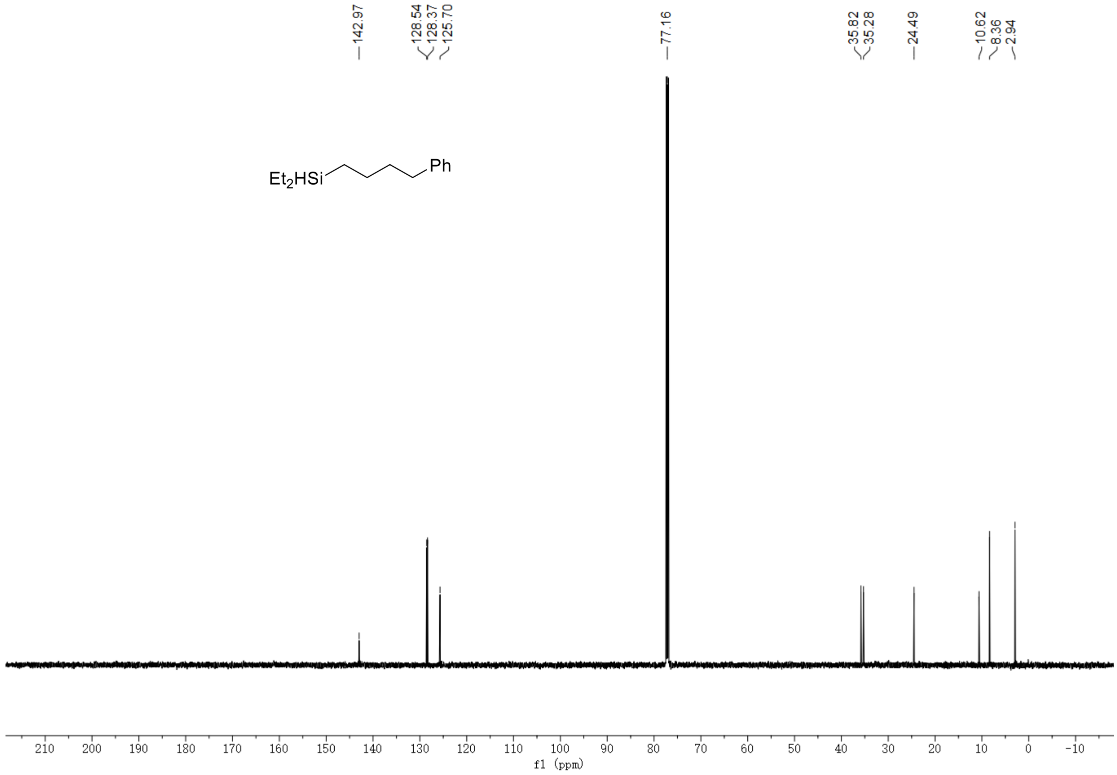

^{29}Si NMR of **12** (119 MHz, $CDCl_3$, 25 ºC)

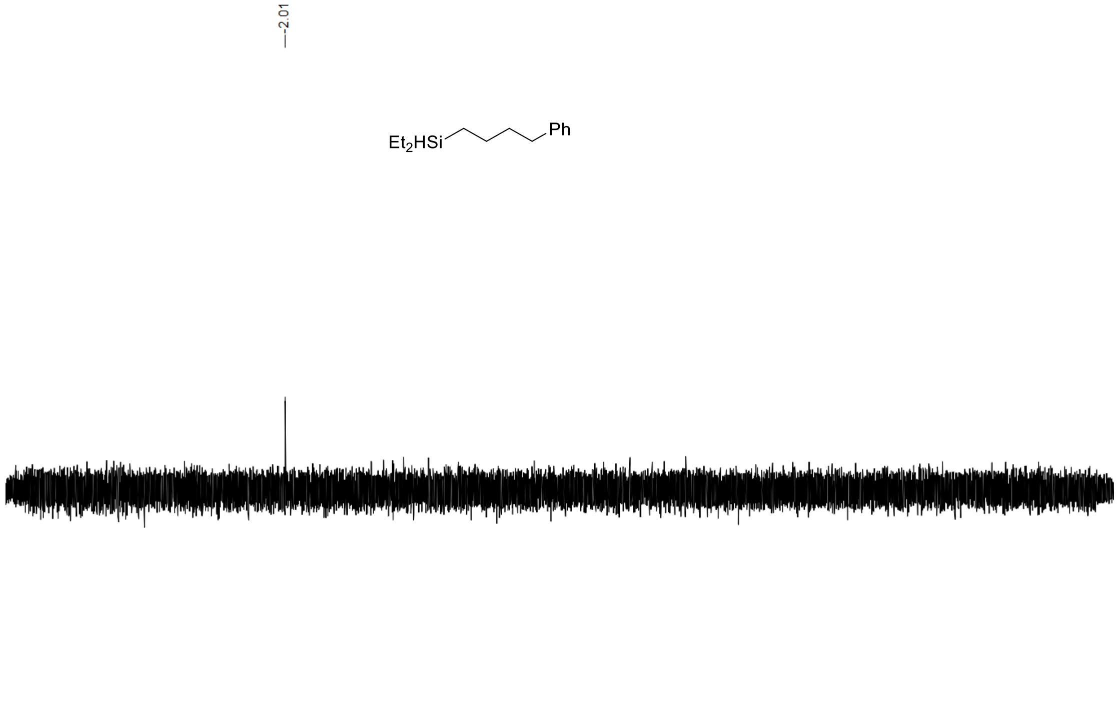

^{1}H NMR of **16** (600 MHz, $CDCl_3$, 25 ºC)

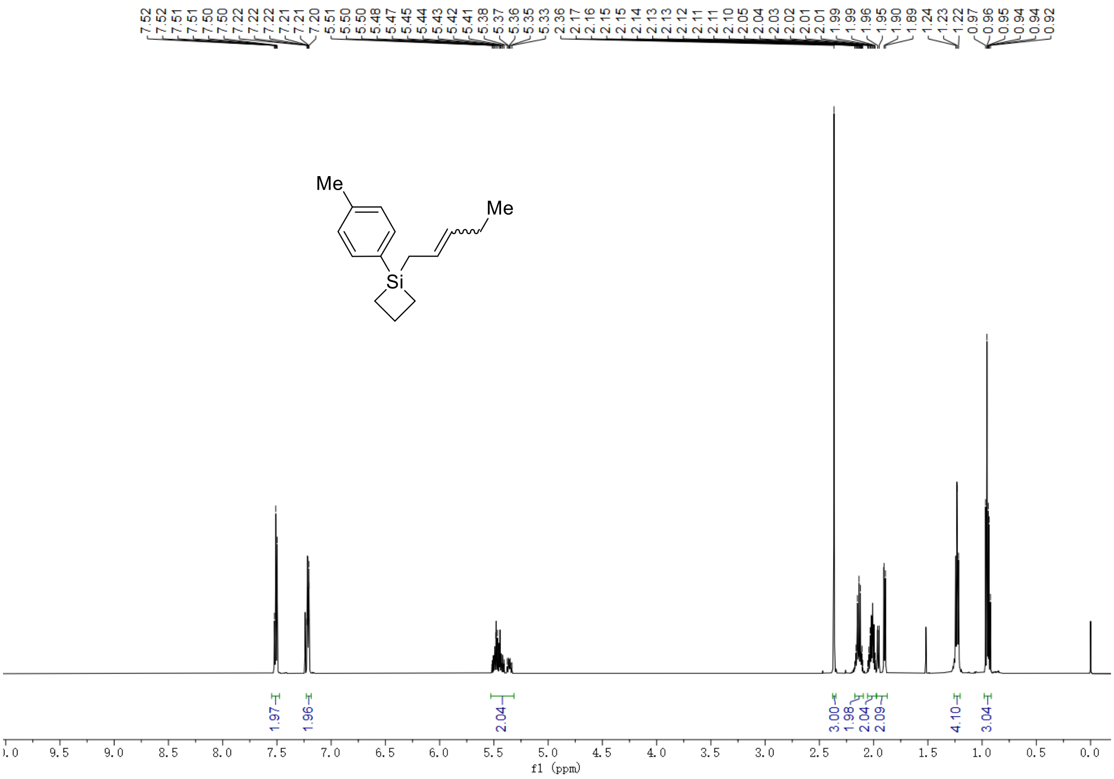

^{13}C NMR of **16** (151 MHz, $CDCl_3$, 25 ºC)

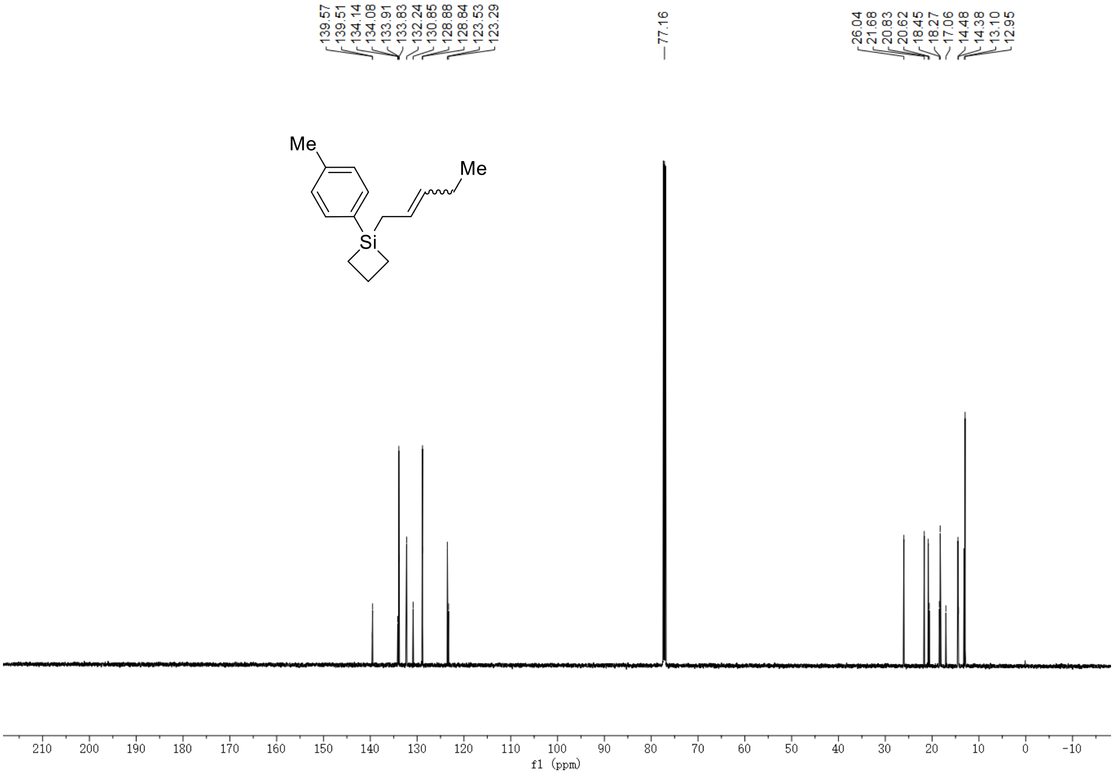

^{29}Si NMR of **16** (119 MHz, $CDCl_3$, 25 ºC)

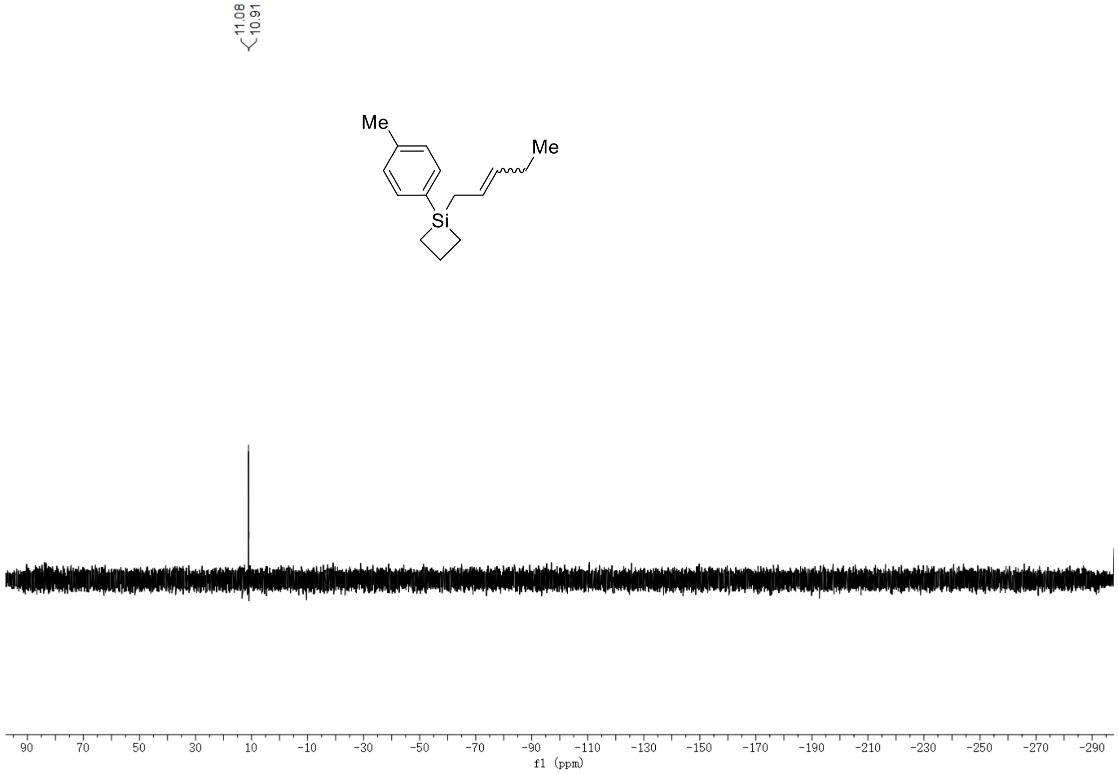

^{1}H NMR of **17** (600 MHz, $CDCl_3$, 25 ºC)

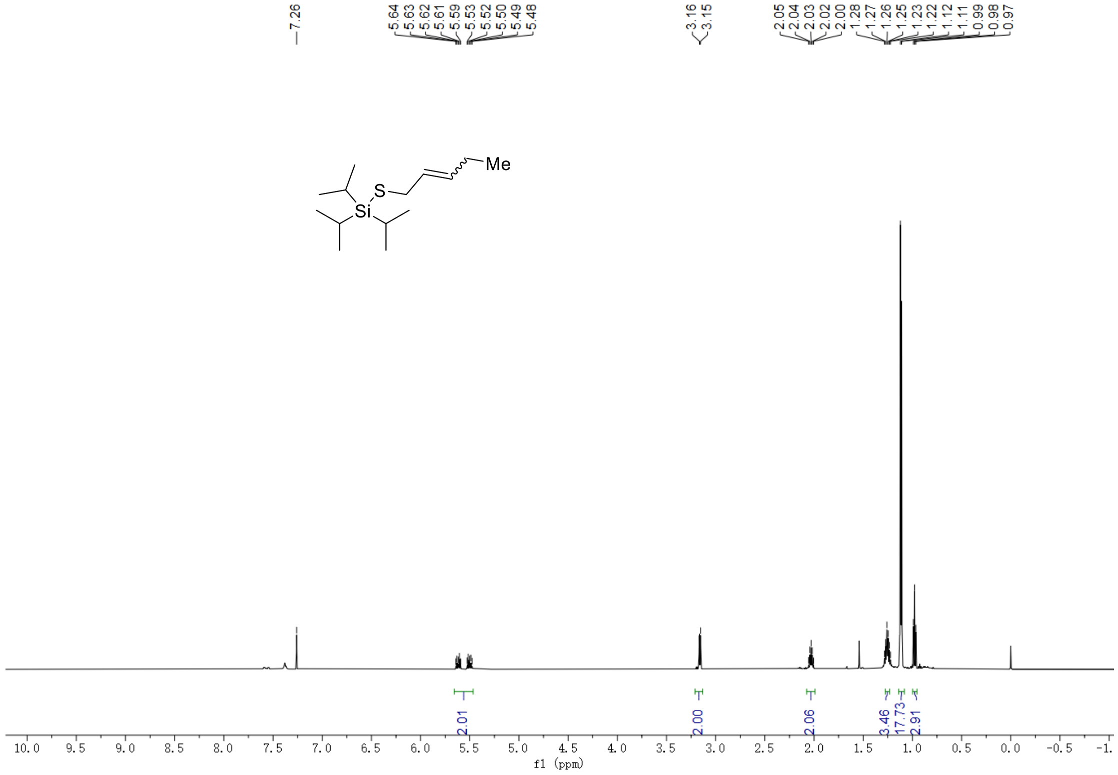

^{13}C NMR of **17** (151 MHz, $CDCl_3$, 25 ºC)

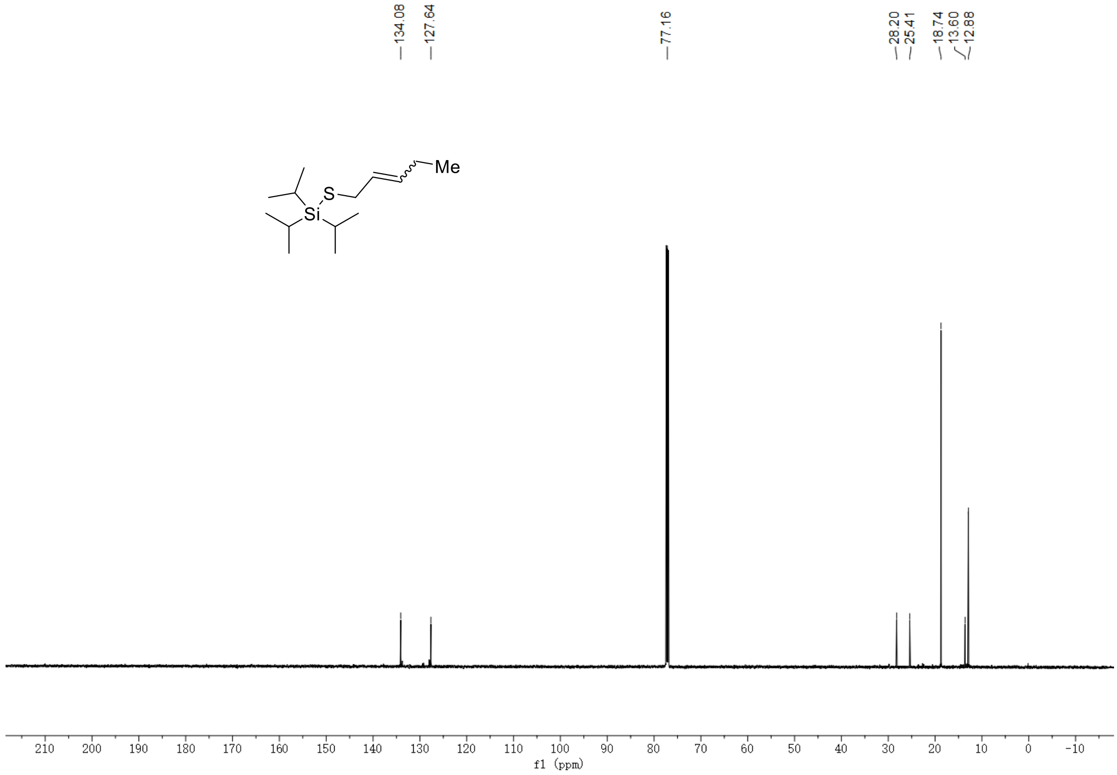

11. References

1. Yang, L.-Y.; Qin, Y.; Zhao, Z.; Zhao, D. Nickel-Catalyzed Reductive Protocol to Access Silacyclobutanes with Unprecedented Functional Group Tolerance. *Angew. Chem. Int. Ed.* **2024**, e202407773.

2. (a) Wang, Y.; Wang, Q.; Wu, L.; Jia, K.; Wang, M.; Qiu, Y. Electroreduction of Unactivated Alkenes using Water as Hydrogen Source. *Nat. Commun.* **2024**, *15*, 2780. (b) Douglass, F. T.; James, F. B.; Timothy. J. M. Convenient Synthetic Route to an Enantiomerically Pure FMOCr-Amino Acid. J. Org. Chem. **2008**, *73*, 9334–9339. (c) Liu, X.; Ye, B.; Wu, Y.; Nan, J.; Lin, Z.; Piao, H. Synthesis and Antitumor Activity of Dehydroepiandrosterone Derivatives on Es-2, A549, and HepG2 Cells in vitro. *Chem. Biol. Drug Des.* **2012**, *79*, 523–529.

3. Chen, H.; Chen, Y.; Tang, X.; Liu, S.; Wang, R.; Hu, T.; Gao, L.; Song, Z. Rhodium-Catalyzed Reaction of Silacyclobutanes with Unactivated Alkynes to Afford Silacyclohexenes. *Angew. Chem. Int. Ed.* **2019**, *58,* 4695–4699.

4. Liu, W.; Lu, W.; Yang, L.; Wu, X.; Zhang, Z. Rhodium-Catalyzed Anti-Markovnikov Hydrosilylation of Alkenes. *Tetrahedron* **2022**, *109,* 132632.

5. Buettner, G, R. Spin Trapping: ESR Parameters of Spin Adducts. *Free Radical Bio. Med.* **1987**, *3*, 259–303.

6. Gaussian 16, Revision A.03, Frisch, M. J.; Trucks, G. W.; Schlegel, H. B.; Scuseria, G. E.; Robb, M. A.; Cheeseman, J. R.; Scalmani, G.; Barone, V.; Mennucci, B.; Petersson, G. A.; Nakatsuji, H.; Caricato, M.; Li, X.; Hratchian, H. P.; Izmaylov, A. F.; Bloino, J.; Zheng, G.; Sonnenberg, J. L.; Hada, M.; Ehara, M.; Toyota, K.; Fukuda, R.; Hasegawa, J.; Ishida, M.; Nakajima, T.; Honda, Y.; Kitao, O.; Nakai, H.; Vreven, T.; Montgomery, J. A.; Peralta, Jr., J. E.; Ogliaro, F.; Bearpark, M.; Heyd, J. J.; Brothers, E.; Kudin, K. N.; Staroverov, V. N.; Kobayashi, R.; Normand, J.; Raghavachari, K.; Rendell, A.; Burant, J. C.; Iyengar, S. S.; Tomasi, J.; Cossi, M.; Rega, N.; Millam, J. M.; Klene, M.; Knox, J. E.; Cross, J. B.; Bakken, V.; Adamo, C.; Jaramillo, J.; Gomperts, R.; Stratmann, R. E.; Yazyev, O.; Austin, A. J.; Cammi, R.; Pomelli, C.; Ochterski, J. W.; Martin, R. L.; Morokuma, K.; Zakrzewski, V. G.; Voth, G. A.; Salvador, P.; Dannenberg, J. J.; Dapprich, S.; Daniels, A. D.;Farkas, O.; Foresman, J. B.; Ortiz, J. V.; Cioslowski, J.; Fox, D. J. Gaussian 16, Revision A.03, Gaussian, Inc., Wallingford CT, **2016**.

7. Zhao, Y.; Truhlar, D. G. The M06 suite of density functionals for main group thermochemistry, thermochemical kinetics, noncovalent interactions, excited states, and transition elements: two new functionals and systematic testing of four M06-class functionals and 12 other functionals. *Theor. Chem. Acc.* **2008**, *120*, 215–241.

8. Krishnan, R.; Binkley, J. S.; Seeger, R.; Pople, J. A. Self-consistent molecular orbital methods. XX. A basis set for correlated wave functions. *J. Chem. Phys.* **1980**, *72*, 650–654.

9. Marenich, A. V.; Cramer, C. J.; Truhlar, D. G. Universal Solvation Model Based on Solute Electron Density and on a Continuum Model of the Solvent Defined by the Bulk Dielectric Constant and Atomic Surface Tensions. *J. Phys. Chem. B.* **2009**, *113*, 6378–6396.
